# Further analyses of the safety of verubecestat in the phase 3 EPOCH trial of mild-to-moderate Alzheimer’s disease

**DOI:** 10.1186/s13195-019-0520-1

**Published:** 2019-08-07

**Authors:** Michael F. Egan, Yuki Mukai, Tiffini Voss, James Kost, Julie Stone, Christine Furtek, Erin Mahoney, Jeffrey L. Cummings, Pierre N. Tariot, Paul S. Aisen, Bruno Vellas, Christopher Lines, David Michelson

**Affiliations:** 1 Merck & Co., Inc., Kenilworth, NJ USA; 2 University of Nevada Las Vegas Department of Brain Health, Cleveland Clinic Lou Ruvo Center for Brain Health, Las Vegas, NV USA; 3 Banner Alzheimer’s Institute, Phoenix, AZ USA; 4 University of Southern California, San Diego, CA USA; 5 Gerontopole, INSERM U 1027, Alzheimer’s Disease Research and Clinical Center, Toulouse University Hospital, Toulouse, France; 6 Merck & Co., Inc., UG 4C-06, P.O. Box 1000, North Wales, PA 19454-1099 USA

**Keywords:** Verubecestat, BACE inhibitor, Amyloid, Alzheimer’s disease, Clinical trial, Safety

## Abstract

**Background:**

Verubecestat, a BACE1 inhibitor that reduces Aβ levels in the cerebrospinal fluid of humans, was not effective in a phase 3 trial (EPOCH) of mild-to-moderate AD and was associated with adverse events. To assist in the development of BACE1 inhibitors, we report detailed safety findings from EPOCH.

**Methods:**

EPOCH was a randomized, double-blind, placebo-controlled 78-week trial evaluating verubecestat 12 mg and 40 mg in participants with mild-to-moderate AD diagnosed clinically. The trial was terminated due to futility close to its scheduled completion. Of 1957 participants who were randomized and took treatment, 652 were assigned to verubecestat 12 mg, 652 to verubecestat 40 mg, and 653 to placebo. Adverse events and relevant laboratory, vital sign, and ECG findings were assessed.

**Results:**

Verubecestat 12 mg and 40 mg were associated with an increase in the percentage of participants reporting adverse events versus placebo (89 and 92% vs. 82%), although relatively few participants discontinued treatment due to adverse events (8 and 9% vs. 6%). Adverse events that were increased versus placebo included falls and injuries, suicidal ideation, weight loss, sleep disturbance, rash, and hair color change. Most were mild to moderate in severity. Treatment differences in suicidal ideation emerged within the first 3 months but did not appear to increase after 6 months. In contrast, treatment differences in falls and injuries continued to increase over time.

**Conclusions:**

Verubecestat was associated with increased risk for several types of adverse events. Falls and injuries were notable for progressive increases over time. While the mechanisms underlying the increased adverse events are unclear, they may be due to BACE inhibition and should be considered in future clinical development programs of BACE1 inhibitors.

**Trial registration:**

ClinicalTrials.gov NCT01739348, registered on 29 November 2012.

**Electronic supplementary material:**

The online version of this article (10.1186/s13195-019-0520-1) contains supplementary material, which is available to authorized users.

## Background

The amyloid hypothesis of Alzheimer’s disease (AD) proposes that amyloid-β (Aβ) peptide aggregates trigger the spreading of tau-related neurofibrillary tangles and subsequent, downstream, neuronal degeneration [1–3]. Aβ is the result of sequential cleavage of amyloid precursor protein (APP) by β-site APP cleaving enzyme 1 (BACE1; also known as β-secretase) and γ-secretase. Inhibition of BACE1 is a potential therapeutic strategy for disease modification in AD by decreasing amyloid production [4]. Previous approaches targeting reduction of amyloid have been associated with adverse effects including amyloid-related imaging abnormalities (ARIAs) with edema or hemorrhage in the case of monoclonal antibodies and worsening of cognition and function along with suspected notch-related symptoms in the case of γ-secretase inhibitors [5–9]. Safety concerns about BACE1 inhibition have arisen on the basis of BACE1’s diverse physiological functions and wide range of substrates, as well as the results of studies of BACE1 inhibition in animals [10, 11]. These studies have suggested that BACE inhibition could, among other effects, potentially impair cognition, myelination, and muscle spindle formation and maintenance, and result in psychosis, skin hypopigmentation, and retinal degeneration.

Verubecestat was the first BACE1 inhibitor to progress to late-stage clinical trials in AD patients. Its chemical, preclinical, and clinical pharmacological profile has been previously described [12, 13]. It is an inhibitor of BACE2 in addition to BACE1. In a phase 3 clinical trial (EPOCH), verubecestat doses of 12 mg and 40 mg were ineffective at slowing the rate of cognitive or functional decline over 78 weeks in participants with mild-to-moderate AD, despite reducing cerebrospinal fluid levels of Aβ by 63 to 81% [14]. Although the outcome of EPOCH was disappointing, BACE1 inhibition requires more study; administration earlier in the AD process, altering dosing strategies, and use in combination therapies are all possible future application of BACE1 inhibitors. Future use of BACE1 inhibitors requires a clear understanding of the benefit/risk profile associated with an experimental treatment.

We previously reported that in EPOCH, chronic treatment for 78 weeks with verubecestat 12 mg and 40 mg was associated with an increase in the percentage of participants reporting adverse events versus placebo (89 and 92% vs. 82%), although relatively few participants discontinued treatment due to adverse events (8 and 9% vs. 6%) [14]. Specific adverse events for verubecestat versus placebo included falls and injuries (20 and 23% vs. 16%), rash (12 and 10% vs. 6%), sleep disturbance (10 and 8% vs. 5%), suicidal ideation (6 and 6% vs. 3%), weight loss (6 and 6% vs. 3%), and hair color change (2 and 3% vs. 0%).

Other EPOCH findings of potential relevance to the safety of BACE1 inhibitors include the observation that there was a modest initial worsening in mean cognition scores for verubecestat versus placebo at week 13 that was not maintained at week 78 [14] and the observation in a magnetic resonance imaging (MRI) substudy of a greater decline in hippocampal volume for verubecestat versus placebo that was apparent from week 13 but did not increase thereafter [15].

Given that EPOCH constitutes the most comprehensive safety database currently available for a BACE1 inhibitor (nearly 2000 randomized participants), we report here on detailed analyses of the safety profile of verubecestat in the mild-to-moderate AD population to help inform further development of BACE1 inhibitors.

## Methods

Full details of the trial methods have been previously reported [14]. The trial protocol is included as Additional file 1. Relevant details for the present paper are summarized below.

### Participants

Eligible participants were aged 55–85 years inclusive and met the standard research and clinical criteria for probable AD dementia [16, 17]. All participants had brain MRI, or computerized tomography if MRI was contraindicated, to exclude alternative causes of dementia and a score of 15–26 on the Mini-Mental State Examination (MMSE) [18]. Participants could be taking an acetylcholinesterase inhibitor and/or memantine provided they were on stable doses prior to screening. Other than the diagnosis of AD, participants were required to be in good general health with no evidence of a current clinically significant neurological, psychiatric, or general medical condition.

### Design and treatment

The trial was conducted at 238 centers in 21 countries from November 2012 to April 2017. The trial consisted of a randomized, double-blind, placebo-controlled, parallel group, 78-week treatment period (part I) followed by an optional extension period with a planned duration of up to 5 years (part II). In part I, participants were randomized to verubecestat 12 mg, 40 mg, or placebo. Participants who completed the part I 78-week treatment period could enter the part II extension in which placebo participants were switched to 40 mg while those on 12 mg or 40 mg remained on the same dose (all in a blinded fashion).

A decision to stop the trial was made in February 2017 at the independent external Data Monitoring Committee’s recommendation, based on futility. At the time of the decision, all participants had been enrolled and part I was 5 months from its scheduled completion. None of the participants who entered part II (the extension) completed this phase of the trial due to the trial termination.

### Safety assessments

Safety was assessed by adverse event reports and by routine laboratory analyses, electrocardiograms (ECGs), and physical examinations. Routine MRI was initially performed to assess possible ARIAs but was discontinued partway through the trial based on external Data Monitoring Committee and regulatory feedback that it was no longer required. Comprehensive dermatological and ophthalmological exams were also performed at baseline and selected follow-up clinic visits. The ophthalmological findings will be the subject of a separate report, but there were no significant treatment effects. Dermatology exams were performed by a dermatologist at baseline and week 26 of treatment and by the site physician using a directed skin examination at weeks 13, 52, and 78 of treatment. Suicidality was assessed by the Columbia Suicide Severity Rating Scale (C-SSRS) [19] at every clinic visit. The Neuropsychiatric Inventory (NPI) [20] was administered at baseline and weeks 8, 13, 26, 52, and 78. Certain adverse events were prespecified as events of clinical interest (ECI), based on the signals observed in preclinical or phase 1 studies with verubecestat or signals observed for other amyloid-lowering treatments, and were subject to additional reporting requirements as detailed in the trial protocol. These included ARIAs, suicidal ideation, rash, and hypopigmentation.

### Statistical analysis

The present analyses focused on data from the part I placebo-controlled 78-week treatment phase. All treated participants were used in safety analyses. Adverse events within 14 days postdose were collected verbatim and mapped to Medical Dictionary for Regulatory Activities (MedDRA) version 20.0 lower-level terms which were then mapped to preferred terms and system organ classes. In addition, selected adverse event terms were clustered in an unblinded and post hoc manner to form “composite” adverse event groupings of related adverse events. The composite adverse events included rash/dermatitis/urticaria, delirium-like events, psychotic symptoms (hallucinations and delusions), injuries and falls, overactive bladder events, sleep disturbance events, and syncopal-like events. Exposure-response relationships were calculated for select preferred or composite adverse event terms. The numbers of participants exceeding predefined limits of change for laboratory, ECG, and vital sign measures were calculated. Additional analyses were performed as detailed in the relevant sections.

All statistical analyses were performed using SAS Versions 9.3 and 9.4 (SAS Institute, Cary, NC, USA).

### Exposure-response analysis

A previously established population pharmacokinetic model was used to estimate the verubecestat exposure (area under the curve over the dosing interval at steady-state, AUC_0–24 h_) for each participant (*n* = 1465) receiving treatment with verubecestat based on concentrations collected during 4–7 visits. Time-weighted exposure was determined based on the average exposure over the total number of days on active treatment. The PROC LOGISTIC procedure in SAS was used to develop logistic regression models to relate drug exposure to dichotomous endpoints (absence or presence of an adverse event). Conclusions with respect to whether an exposure-response relationship existed for a given endpoint was based on testing for significance (*P* < 0.05) that the slope of the linear AUC_0–24 h_ relationship was different from zero based on a fit of the active treated participants only.

## Results

### Participants

Participant disposition and demographics have been described previously [14]. Of the 1957 randomized participants who were treated with verubecestat 12 mg, 40 mg, or placebo, 1389 (70–72% in each group) completed part I. The main reasons for discontinuation were trial termination and adverse events. At baseline, the mean age of participants was 72 years (range 55 to 86), 55% were women, 52% were classified as AD of moderate severity based on MMSE score of ≤ 20 (48% had dementia of mild severity based on MMSE score of ≥ 21), and 89% were taking an acetylcholinesterase inhibitor and/or memantine.

### Safety overview

Adverse events by system organ class and preferred term are summarized in Table 1. System organ classes with a preferred term that showed an increase in at least one verubecestat group versus placebo (defined as the lower bound of the 95% confidence interval [CI] for the difference vs. placebo being > 0) were gastrointestinal (diarrhea, gastritis), infections/infestations (conjunctivitis), injury (head injury, skin abrasion), investigations (weight decreased), musculoskeletal (muscle spasms, pain in extremity), nervous system (dizziness), psychiatric (anxiety, insomnia, sleep disorder, suicidal ideation), renal/urinary (hypertonic bladder), and skin/subcutaneous (alopecia, hair color changes, urticaria). Composite adverse events that showed an increase in at least one verubecestat group versus placebo (defined as above) were injury or fall, overactive bladder symptoms, psychotic symptoms, rash/dermatitis/urticaria, and sleep disturbance (Table 2). Although numerically the results in Tables 1 and 2 suggested a dose-response trend (more adverse events in the 40 mg group compared to 12 mg), the results of exposure-response modeling suggested no significant exposure-response effects for the adverse events evaluated (Table 3).

**Table 1:** Summary of adverse events by system organ class

System organ class	Number (%)			Treatment difference (95% CI^a^)	
	12 mg (*N* = 652)	40 mg (*N* = 652)	Placebo (*N* = 653)	12 mg vs. placebo	40 mg vs. placebo
Any					
≥ 1 adverse event	582 (89.3)	601 (92.2)	533 (81.6)	7.64 (3.84, 11.48)	10.55 (6.96, 14.23)
Gastrointestinal					
Diarrhea	53 (8.1)	57 (8.7)	38 (5.8)	2.31 (− 0.46, 5.14)	2.92 (0.10, 5.81)
Gastritis	11 (1.7)	9 (1.4)	3 (0.5)	1.23 (0.13, 2.59)	0.92 (− 0.14, 2.20)
Infections					
Conjunctivitis	5 (0.8)	13 (2.0)	4 (0.6)	0.15	1.38 (0.17, 2.84)
Injury					
Head injury	3 (0.5)	9 (1.4)	2 (0.3)	0.15	1.07 (0.10, 2.33)
Skin abrasion	9 (1.4)	15 (2.3)	6 (0.9)	0.46 (− 0.78, 1.79)	1.38 (0.02, 2.95)
Investigations					
Weight decreased	42 (6.4)	42 (6.4)	20 (3.1)	3.38 (1.10, 5.81)	3.38 (1.10, 5.81)
Metabolism/nutrition					
Decreased appetite	16 (2.5)	29 (4.4)	16 (2.5)	0.00 (− 1.76, 1.77)	2.00 (0.02, 4.11)
Musculoskeletal and connective tissue					
Muscle spasms	9 (1.4)	16 (2.5)	6 (0.9)	0.46 (− 0.78, 1.79)	1.54 (0.15, 3.13)
Pain in extremity	14 (2.1)	20 (3.1)	8 (1.2	0.92 (− 0.52, 2.49)	1.84 (0.29, 3.60)
Nervous system					
Dizziness	31 (4.8)	53 (8.1)	32 (4.9)	− 0.15 (− 2.53, 2.23)	3.23 (0.56, 5.99)
Psychiatric					
Anxiety	39 (6.0)	46 (7.1)	24 (3.7)	2.31 (− 0.02, 4.73)	3.38 (0.96, 5.93)
Insomnia	35 (5.4)	29 (4.4)	20 (3.1)	2.31 (0.13, 4.60)	1.39 (− 0.70, 3.55)
Sleep disorder	18 (2.8)	8 (1.2)	3 (0.5)	2.30 (1.04, 3.91)	0.77 (− 0.27, 2.00)
Suicidal ideation	39 (6.0)	38 (5.8)	21 (3.2)	2.77 (0.51, 5.15)	2.61 (0.37, 4.98)
Renal and urinary					
Hypertonic bladder	2 (0.3)	7 (1.1)	1 (0.2)	0.15	0.92 (0.10, 2.06)
Skin and subcutaneous tissue					
Alopecia	1 (0.2)	7 (1.1)	0 (0.0)	0.15	1.07 (0.49, 2.20)
Hair color changes	12 (1.8)	16 (2.5)	0 (0.0)	1.84 (1.06, 3.19)	2.45 (1.52, 3.95)
Urticaria	14 (2.1)	12 (1.8)	3 (0.5)	1.69 (0.52, 3.16)	1.38 (0.26, 2.78)

Data shown are for adverse events with incidence of ≥ 1% in one or more verubecestat groups and where the lower bound of the 95% confidence interval for the difference versus placebo is > 0 in at least one verubecestat group

^a^Confidence intervals only produced for those comparisons for which at least one of the treatment groups (verubecestat or placebo) had an incidence ≥ 1%

**Table 2:** Number (%) of participants with composite adverse events and treatment differences

Composite adverse event	Number (%)			Treatment difference (95% CI)	
	12 mg (*N* = 652)	40 mg (*N* = 652)	Placebo (*N* = 653)	12 mg vs. placebo	40 mg vs. placebo
Delirium-like events	13 (2.0)	31 (4.8)	22 (3.4)	− 1.38 (− 3.26, 0.40)	1.39 (− 0.78, 3.63)
Injury or fall	132 (20.2)	151 (23.2)	103 (15.8)	4.47 (0.30, 8.65)	7.39 (3.10, 11.68)
Overactive bladder symptoms	12 (1.8)	27 (4.1)	12 (1.8)	0.00 (− 1.56, 1.57)	2.30 (0.48, 4.31)
Psychotic symptoms	30 (4.6)	36 (5.5)	20 (3.1)	1.54 (− 0.56, 3.73)	2.46 (0.27, 4.77)
Rash, dermatitis, urticaria	79 (12.1)	66 (10.1)	38 (5.8)	6.30 (3.24, 9.47)	4.30 (1.38, 7.32)
Sleep disturbance	67 (10.3)	55 (8.4)	31 (4.7)	5.53 (2.71, 8.48)	3.69 (1.01, 6.47)
Syncopal like events	26 (4.0)	27 (4.1)	17 (2.6)	1.38 (− 0.58, 3.44)	1.54 (− 0.44, 3.62)

**Table 3:** Linear logistic regression models of time weighted AUC_0–24 h_ by adverse event (*N* = 1465 participants)

Adverse event	Estimate (standard error)	Odds ratio (95% CI)	*P* value
Insomnia/sleep disorders	− 0.0919 (0.0479)	0.912 (0.830, 1.002)	0.0549
Serious adverse events	0.0470 (0.0285)	1.048 (0.991, 1.108)	0.0989
Psychotic symptoms	0.2671 (0.1959)	1.306 (0.890, 1.917)	0.1728
Muscle spasm	0.1047 (0.0810)	1.110 (0.947, 1.301)	0.1963
Anxiety	0.0611 (0.0475)	1.063 (0.968, 1.167)	0.1987
Rash/dermatitis/urticaria	− 0.0363 (0.0466)	0.964 (0.880, 1.057)	0.4356
Diarrhea	0.0267 (0.0441)	1.027 (0.942, 1.120)	0.5444
Falls and injuries	− 0.0171 (0.0353)	0.983 (0.917, 1.053)	0.6274
Pain in the extremity	0.0270 (0.0733)	1.027 (0.890, 1.186)	0.7128
Syncope-like (with loss of consciousness)	0.0188 (0.0557)	1.019 (0.914, 1.137)	0.7355
Rash event of clinical interest	0.0176 (0.0585)	1.018 (0.907, 1.141)	0.7639
Weight decreased	− 0.0106 (0.0483)	0.989 (0.900, 1.088)	0.8260
Suicidal ideation	− 0.0041 (0.0498)	0.996 (0.903, 1.098)	0.9342

AUC_0-24 h_, area under the concentration time curve within a 24-h dosing interval at steady state

With regard to the percentage of participants exceeding predefined limits of change in the vital signs, more participants on verubecestat 12 mg and 40 mg showed a ≥ 7% decrease in weight versus placebo (23.7 and 29.4% vs. 13.1%) and fewer participants on verubecestat 12 mg and 40 mg showed a ≥ 7% increase in weight versus placebo (7.1 and 8.0% vs. 15.5%) (Additional file 2: Table S1). No treatment differences were seen in the percentages of participants exceeding predefined limits of change for other vital signs (Additional file 2: Table S1), ECG measures (Additional file 2: Table S1), liver function tests (Additional file 3: Table S2), or other laboratory tests (Additional file 4: Table S3). There were no cases of drug-induced liver injury.

### Falls and injuries

**Fig. 1:**
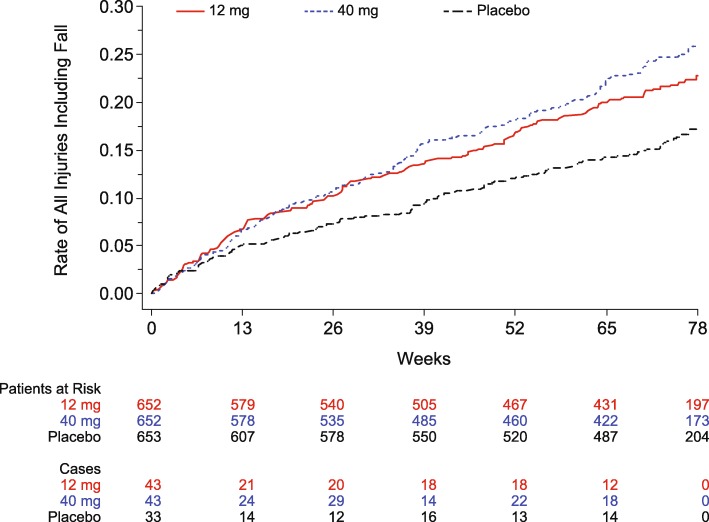
Kaplan-Meier plot of time to first event of any injury including fall. “Patients at Risk” shows the number of evaluable patients at each time point by treatment group. “Cases” shows the number of new cases of fall/injury occurring between the corresponding time point and the next time point (e.g., for 12 mg, there were 43 new cases of fall/injury between week 0 and week 13). A difference in the risk of fall/injury between verubecestat and placebo became apparent after 13 weeks and then increased over time

Fall and injury adverse event terms were combined into a composite term because on review of the narratives, many injury adverse events were noted to be due to a fall; per data entry conventions, the injury adverse event caused by the fall, and not necessarily the fall itself, was entered as the adverse event. Hip and femur fractures were the most common type of injury. Both verubecestat 12 mg and 40 mg showed an increase versus placebo in the injury or fall composite term (20.2 and 23.3% vs. 15.8%, Table 2). A Kaplan-Meier plot of the fall/injury composite term suggested that the treatment difference became apparent after 13 weeks and then increased over time (Fig. 1). No significant exposure-response effect was seen for the composite term (Table 3). When comparing participants with fall/injury versus those without, there were no marked differences in vital signs (including weight change) or related adverse events such as syncope, dizziness, or cardiac events. Participants with falls/injuries reported numerically higher rates of pre-existing medical conditions from a wide range of system organ class categories, but none appeared higher only in the verubecestat treated groups. Similarly, participants with falls/injuries were treated more frequently with concomitant medications from a wide variety of medication classes, but none appeared substantially higher only in the verubecestat groups. Greater age and higher MMSE scores were associated with increased risk of fall/injury in all groups including placebo. In the placebo group, falls/injuries were reported in 18.4% of women versus 12.7% of men; in the verubecestat groups, the rates of falls/injuries were similar between men and women. The percentage of participants with specific adverse events of head injury or skin abrasion adverse events was increased for the 40 mg dose of verubecestat versus placebo: the overall percentage of participants reporting these adverse events was < 2% (Table 1). Of the participants who experienced a fall/injury adverse event, the numbers (percentages) considered serious were 17/132 (12.9%), 18/151 (11.9%), and 9/103 (8.7%), in the 12 mg, 40 mg, and placebo groups, respectively.

Syncope-like events were reported for 4.0% and 4.1% of participants taking verubecestat 12 mg and 40 mg, respectively, versus 2.6% for placebo although the lower bounds of the 95% CIs for the differences were less than zero (Table 2). No significant exposure-response effect was seen (Table 3).

### Suicidal ideation

An ECI of suicidal ideation and behavior was defined as any positive response on the C-SSRS. Therefore, some events reported may not be new or worsening of a pre-existing condition. The incidence of ECI of suicidal ideation was higher in the verubecestat 12 mg and 40 mg groups than the placebo group (6.0% and 5.8% vs. 3.2%; treatment difference [95% CI] = 2.77 [0.51, 5.15] for 12 mg vs. placebo and 2.61 [0.37, 4.98] for 40 mg vs. placebo) (Table 1). There did not appear to be an exposure-response relationship for suicidal ideation (Table 3).

The incidence of treatment-emergent suicidal ideation or behavior events (i.e., a new or worsening of suicidal ideation or behavior after the initiation of treatment in comparison with the period between the screening and baseline clinic visits) per the C-SSRS was numerically higher in the verubecestat groups compared with placebo (Table 4). Treatment-emergent suicidal behavior was reported by three (0.5%) participants, each in the 12 mg and 40 mg groups, and one (0.2%) participant in the placebo group. Two (0.3%) participants in the 40 mg group had a suicide attempt and two (0.3%) participants in the 12 mg group completed suicide. Of the seven participants with treatment-emergent suicidal behavior, six (85.7%) had a history of depression or anxiety and all were on antidepressants. In addition, six of the seven participants with suicidal behavior (85.7%) had moderate dementia at baseline, and six of the seven (85.7%) reported mild-to-moderate psychiatric symptoms (i.e., depression, anxiety, or psychotic symptoms) prior to the behavior (based on the NPI).

**Table 4:** Summary of participants with worsening any time postdose on individual domains of the Neuropsychiatric Inventory

Domain	12 mg, n/m (%)	40 mg, n/m (%)	Placebo, n/m (%)
Delusions	135/633 (21.3)	124/632 (19.6)	153/639 (23.9)
Hallucinations	84/633 (13.3)	89/632 (14.1)	79/639 (12.4)
Agitation/aggression	245/633 (38.7)	258/632 (40.8)	259/639 (40.5)
Depression/dysphoria	278/633 (43.9)	333/632 (52.7)	256/639 (40.1)
Anxiety	269/633 (42.5)	270/632 (42.7)	250/639 (39.1)
Elation/euphoria	66/633 (10.4)	60/632 (9.5)	62/639 (9.7)
Apathy/indifference	294/633 (46.4)	297/632 (47.0)	306/639 (47.9)
Disinhibition	159/633 (25.1)	155/632 (24.5)	128/639 (20.0)
Irritability/lability	244/633 (38.5)	284/632 (44.9)	244/639 (38.2)
Aberrant motor behavior	177/633 (28.0)	188/632 (29.7)	198/639 (31.0)
Sleep and nighttime behavior disorders	191/633 (30.2)	225/632 (35.6)	182/639 (28.5)
Appetite and eating changes	252/632 (39.9)	255/631 (40.4)	225/639 (35.2)

Worsening was determined by comparing the postdose score to the baseline score

n/m = number of participants with the given item present at that time point/number of participants measured at that time point

**Fig. 2:**
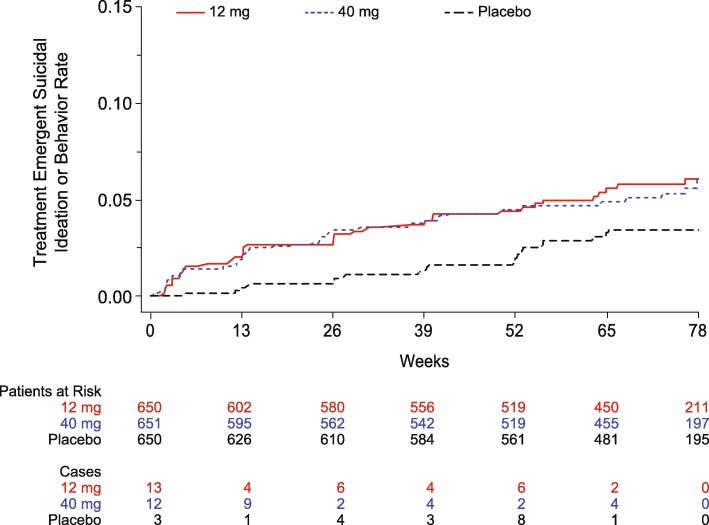
Kaplan-Meier plot of time to first treatment-emergent suicidal ideation/behavior. This analysis uses the time frame between the screening and baseline clinic visits as the reference period for determining “treatment-emergent.” “Patients at Risk” shows the number of evaluable patients at each time point by treatment group. “Cases” shows the number of new cases of suicidal ideation/behavior occurring between the corresponding time point and the next time point (e.g., for 12 mg, there were 13 new cases of suicidal ideation/behavior between week 0 and week 13). A difference in the risk of suicidal ideation/behavior between verubecestat and placebo occurred within the first 6 months of treatment but did not progress further through the remainder of the trial

The majority of suicidal ideation events were either passive or non-specific active thoughts. Regarding more severe suicidal ideation (active with method or worse), the majority was reported only at a single clinic visit and resolved by the next scheduled visit. Two participants in the 12 mg group and one each in the 40 mg group and placebo had more than one occurrence of more severe suicidal ideation. Four participants discontinued treatment due to suicidal ideation (three on verubecestat, one on placebo). The majority of participants (67–80%) who reported any treatment-emergent suicidal ideation or behavior had a medical history of psychiatric disorders including depression, anxiety, and/or suicidal ideation, which was higher than the overall trial population (45–48%). Treatment-emergent differences between verubecestat and placebo occurred within the first 6 months of treatment but did not progress further through the remainder of the trial (Fig. 2).

### Neuropsychiatric symptoms

Verubecestat was associated with increases in the percentages of participants with adverse events broadly categorized as “neuropsychiatric” in nature including dizziness, anxiety, psychotic-like symptoms (delusions and hallucinations), and sleep disturbance (Tables 1 and 2). There was no evidence of exposure-response relationships for these adverse events (Table 3). Regarding the NPI total scores, in the overall population, there were no nominally significant differences between the groups at any time point (Additional file 5: Table S4). The following individual items showed a numerical increase in the percentages of participants with worsening any time postdose for both the verubecestat 12 mg and 40 mg dose groups versus placebo (Table 5): hallucinations (13.3 and 14.1% vs. 12.4%), depression/dysphoria (43.9 and 52.7% vs. 40.1%), anxiety (42.5 and 42.7% vs. 39.1%), disinhibition (25.1 and 24.5% vs. 20.0%), irritability/lability (38.5 and 44.9% vs. 38.2%), sleep and nighttime behavior disorder (30.2 and 35.6% vs. 28.5%), and appetite and eating changes (39.9 and 40.4% vs. 35.2%).

**Table 5:** Number (%) of participants with dermatological endpoints and treatment differences

Adverse event category	Number (%)			Treatment difference (95% CI)	
	12 mg (*N* = 652)	40 mg (*N* = 652)	Placebo (*N* = 653)	12 mg vs. placebo	40 mg vs. placebo
Hypopigmentation composite endpoint^a^	16 (2.5)	16 (2.5)	14 (2.1)	0.31 (− 1.39, 2.04)	0.31 (− 1.39, 2.04)
Hypopigmentation event of clinical interest	10 (1.5)	9 (1.4)	11 (1.7)	− 0.15 (− 1.64, 1.32)	− 0.3 (− 1.77, 1.12)
Rash event of clinical interest	30 (4.6)	28 (4.3)	8 (1.2)	3.38 (1.63, 5.39)	3.07 (1.36, 5.04)
Severe rash^b^	0 (0.0)	0 (0.0)	0 (0.0)	0	0

^a^Comprised of the following terms: skin hypopigmentation, skin depigmentation, vitiligo, leukoderma, hypopigmentation of the eyelid, and idiopathic guttate hypomelanosis

^b^Stevens-Johnson syndrome, toxic epidermal necrolysis, or drug reaction with eosinophilia and systemic symptoms

### Weight loss

More participants on verubecestat 12 mg and 40 mg had an adverse event of weight loss versus placebo (6.4 and 6.4% vs. 3.1%, Table 1), and there were more participants who exceeded the predefined limit of change of a ≥ 7% decrease versus baseline (23.7 and 29.4% vs. 13.1%, Additional file 2, Table S1). No exposure-response relationship was seen for weight loss adverse events (Table 3). The verubecestat 40 mg group showed an increase in the percentage of participants with diarrhea and decreased appetite adverse events versus placebo (Table 1). The percentage of participants who showed a worsening on the “Appetite and eating changes” item of the NPI was numerically higher for verubecestat 12 mg and 40 mg versus placebo (39.9% and 40.4% vs. 35.2%, Table 5). Several deaths were attributed to metabolism and nutrition disorders associated with weight loss: failure to thrive (one participant on 12 mg), hypophagia (two participants on 40 mg), and starvation (one participant on placebo).

### Dermatological adverse events and hair color change

Dermatological adverse events are summarized in Table 6. The incidence of rash ECI was higher for participants in both the verubecestat dose groups versus placebo. There were no cases of Stevens-Johnson syndrome, toxic epidermal necrolysis, or drug-related eosinophilia with systemic symptoms (DRESS). Most cases of rash were mild to moderate in severity and did not require discontinuation of study medication. In addition, there was no relationship between the dose of study medication and the duration of rash. At the time of the last follow-up, all rashes resolved or were resolving either with or without concomitant treatment (e.g., antihistamine or corticosteroids for most participants) except one participant on 40 mg who had transient mild acantholytic dermatosis that was biopsied by a dermatologist. The biopsy results were consistent with Grover’s disease. In addition to rash ECIs, other adverse events similar to rash were also reported, including dermatitis and urticaria. When these terms were combined together, the incidence was greater for both the verubecestat groups than for placebo (Table 2).

**Table 6:** Most severe treatment-emergent suicidal ideation/behavior event summary

Category	12 mg, n/m (%)	40 mg, n/m (%)	Placebo, n/m (%)
With one or more ideation or behavior events	35/651 (5.4)	35/651 (5.4)	21/651 (3.2)
Suicidal ideation	33/651 (5.1)	35/651 (5.4)	21/651 (3.2)
Passive-wish to be dead	18/639 (2.8)	17/642 (2.6)	16/637 (2.5)
Active-non-specific (without regard to method, intent, or plan)	9/645 (1.4)	7/650 (1.1)	1/647 (0.2)
Active-method (without regard to intent or plan)	3/647 (0.5)	5/650 (0.8)	3/649 (0.5)
Active-method and intent (without regard to plan)	2/651 (0.3)	3/651 (0.5)	0/651 (0.0)
Active-method, intent, and plan	1/651 (0.2)	3/651 (0.5)	1/651 (0.2)
Suicidal behavior	3/651 (0.5)	3/651 (0.5)	1/651 (0.2)
Preparatory actions or behaviors	1/651 (0.2)	0/651 (0.0)	0/651 (0.0)
Aborted attempt	0/651 (0.0)	0/651 (0.0)	1/651 (0.2)
Interrupted attempt	0/651 (0.0)	1/651 (0.2)	0/651 (0.0)
Suicide attempt	0/651 (0.0)	2/651 (0.3)	0/651 (0.0)
Completed suicide	2/651 (0.3)	0/651 (0.0)	0/651 (0.0)
Non-suicidal self-injurious behavior	1/651 (0.2)	1/651 (0.2)	0/651 (0.0)

This analysis uses the time frame between the screening and baseline clinic visits as the reference period for determining “treatment-emergent.” For each category, the population (= m) only includes treated participants for whom worsening from the reference period was possible. For suicidal ideation categories, worsening is defined as an increasing progression from one category to another along the spectrum (from passive down to active—method, intent, and plan). For suicidal behavior categories, worsening is defined as an increasing progression from one category to another along the spectrum (from preparatory actions or behaviors down to completed suicide)

The incidence of angioedema (standardized MedDRA query) was 2.3 to 3.2% for verubecestat versus 0.5% for placebo. In addition, there were six events of swollen face in five participants taking verubecestat; the participants recovered while continuing verubecestat or, in one case, with a brief interruption of treatment. There was one event of anaphylaxis (standardized MedDRA query) in a participant taking verubecestat 40 mg. The event was described as “circulatory collapse” of moderate severity and lasted for 12 h; verubecestast was interrupted, and the event did not recur after the treatment initiation.

There were no differences between the groups in the incidence of skin hypopigmentation events of clinical interest or in a prespecified composite endpoint which included all observed adverse event terms related to reduced skin pigment (Table 6).

Hair color change was reported in 1.8% and 2.5% of participants on verubecestat 12 mg and 40 mg, respectively, versus no participants on placebo (Table 1). Most instances were mild in severity, and none led to treatment discontinuation.

### Deaths

As previously reported, there were nine deaths in the 12 mg group, 12 deaths in the 40 mg group, and five deaths in the placebo group [14]. Deaths related to suicides and weight loss are described in the relevant sections above. In addition, there were three deaths due to drowning in the verubecestat groups (one in the 12 mg group and two in the 40 mg group) versus none in the placebo group. The drownings occurred while participants were bathing unsupervised, and it is uncertain whether they were associated with syncope or suicidal ideation. However, none of the participants who drowned had a prior history of syncope or suicidal ideation. The drownings all occurred in Japan, where bathtubs are often deeper than in other countries. Future trials of AD treatments should consider recommending that participants be supervised while bathing, particularly in Japan.

## Discussion

The results from the EPOCH trial indicated that BACE1 inhibition by verubecestat was generally well-tolerated over 78 weeks in participants with mild-to-moderate AD. Relatively few participants discontinued verubecestat due to adverse events (~ 9% vs. 6% for placebo). Nevertheless, verubecestat was associated with adverse events which should be considered in evaluating the benefit-risk profile of BACE inhibitors.

Falls and injuries are common in the elderly, but verubecestat was associated with an increase versus placebo. The reason for the apparent increase is uncertain but could be multifactorial given that verubecestat was also associated with numerically higher incidences of adverse events that could contribute to falls such as dizziness, sleep disturbance, and syncope-like events. There were no marked differences between verubecestat and placebo in vital signs (other than weight) or ECG parameters and no obvious risk factors that contributed to the difference. There was an increase in syncopal-like adverse events in the verubecestat groups versus placebo but no clear relationship to falls/injuries. Falls are a leading cause of morbidity and mortality in the elderly [21]. While causes appear to be multifactorial, medication use is thought to play a role [22]. An increased risk for falls/injuries has been observed for other central nervous system (CNS) medications in the elderly [23, 24]. Specific interventions that may reduce falls such as risk management education for the caregiver, and medication management, should be considered in the context of future trials of BACE inhibitors [21].

Prior reports have suggested that the incidence of suicidal ideation in AD may be low [25]. Possible increase in suicidal ideation is a concern for all CNS drugs [26]. Accordingly, in the EPOCH trial suicidal ideation was assessed at every visit, and treatment-emergent changes were considered ECI. Three percent of those in the placebo group reported suicidal ideation compared to 5.4% in those taking verubecestat. The majority of these events were passive or non-specific active thoughts. However, there were two completed suicides and one attempted suicide, all of which occurred on verubecestat. There was also an increase in the percentages of participants who reported neuropsychiatric symptoms such as sleep disturbance, psychotic-like symptoms (delusions and hallucinations), and anxiety with verubecestat treatment. Future trials of BACE inhibitors should monitor participants for the changes in behavior and psychiatric signs or symptoms.

Verubecestat was associated with weight loss that amounted to a mean decrease of − 1.4 and − 1.7 kg in the verubecestat 12 mg and 40 mg groups versus a mean 0.1 kg increase in the placebo group at week 78 [14]. There was a doubling in the percentage of participants with weight loss adverse events and who exceeded the prespecified ≥ 7% weight decrease limit for verubecestat versus placebo. In addition, an increased percentage of participants on verubecestat showed worsening on the appetite item of the NPI and had an increase in gastrointestinal adverse events such as diarrhea. There were three deaths in the verubecestat groups attributable to disorders associated with weight loss. It is not known if weight loss is a class effect of BACE1 inhibitors or specific to verubecestat.

There was an increase in rash-related adverse events for verubecestat versus placebo. Most of these were treatable with antihistamines and/or corticosteroids and generally did not result in study discontinuation. It is unclear whether this is a mechanism-based effect or specific to verubecestat. However, development of another BACE inhibitor, BI 1181181, was reportedly terminated following the observation of rash in participants in an early phase dose-ranging trial [27].

Verubecestat also resulted in hair color change in a small percentage (~ 3%) of participants. Given that hair color change was not reported in any participants in the placebo group, BACE2 (and possibly also BACE1) has been shown to contribute to fur pigmentation in animals [28, 29], and verubecestat is an inhibitor of BACE2 as well as BACE1 [13], it is reasonable to assume that this is an effect related to BACE2 and/or BACE1 inhibition. There were no treatment-related differences in the incidence of skin hypopigmentation or related events. From a participant perspective, hair color change was not troublesome and no participants discontinued treatment because of it. In a clinical trial setting, the occurrence of hair color change raises the possibility of unblinding for a small number of participants.

Aside from BACE2 inhibition, the only other significant off-target (non-BACE1) activity of verubecestat is the inhibition of the hERG channel which is a common cause of QT_C_ prolongation and cardiac arrhythmias [13]. However, at therapeutic doses, verubecestat has a relatively little hERG activity [13] and no significant QT_C_ prolongation was observed in EPOCH. Small increases in QT_C_ were observed in phase 1 trials at doses of ≥ 300 mg [13], much higher than the maximum dose evaluated in EPOCH.

The clinical development of several other BACE1 inhibitors was discontinued due to liver toxicity/elevated liver enzymes [30, 31]. There were no clinically significant liver enzyme elevations in the EPOCH trial, suggesting that the observation with other BACE inhibitors is not a class effect. ARIAs have been reported for monoclonal antibodies [6–9], but there were no differences in the proportions of patients with ARIAs between verubecestat and placebo in EPOCH [14]. The clinical significance of ARIAs is unclear, but in some cases, they have been associated with clinical manifestations [9, 32].

As noted in the introduction, in an imaging substudy of EPOCH, verubecestat reduced MRI hippocampal volume from week 13 to week 78 compared to placebo (reduction of ~ 5.7% vs. 5.0%) [14, 33]. Similar reductions have been reported with other anti-amyloid therapies, and the clinical significance is unknown [34, 35]. In previous cases, it has been postulated that the increased reduction reflected detrimental (e.g., neurodegeneration) or beneficial (e.g., reduced inflammation, reduced plaques) effects [34, 35]. Further analyses to understand this finding are being performed and will be the subject of a separate paper.

We also noted in the introduction that in EPOCH, there was a modest initial worsening in mean cognition scores for verubecestat versus placebo at week 13 that was not maintained at week 78 [14]. For the 40 mg group, this amounted to a 0.8-point difference from placebo in ADAS-Cog_11_ score at week 13. Adverse event reports of cognitive impairment were rare, suggesting that the clinical impact of any treatment-related cognitive worsening was not obvious to clinicians and patients/caregivers, although this might have been influenced by the expectation of cognitive decline in this population. Results from the APECS trial of verubecestat in prodromal AD also showed an early-onset worsening in cognition (and additionally function) associated with verubecestat treatment [36]. Given that these are post hoc findings of relatively small magnitude, they should be treated with caution. However, there have been preliminary reports of cognitive worsening for two other BACE inhibitors [37, 38]. Speculatively, the cognitive worsening could be due to several different factors that might, for example, include effects on other (non-APP) BACE substrates or off-target (e.g., BACE2) effects [10, 11]. Regarding the time course of cognitive worsening in EPOCH, one possible explanation is that the AD pathology-driven cognitive decline may be larger than the BACE effect and make it more difficult to detect as the disease progresses in later stages. Further analyses to understand the effects of verubecestat on cognition are being performed and will be the subject of a separate paper.

## Conclusions

In conclusion, although verubecestat was generally well tolerated over 78 weeks of treatment in patients with mild-to-moderate AD, its safety profile was not as favorable as anticipated on the basis of preclinical and phase 1 findings. The increased risk of falls/injuries is notable as no clear set of risk factors or premonitory signs or symptoms were found. Increased risk of suicidal ideation was also observed, particularly within the first 6 months, and appeared to be predominantly mild and of short duration. The increased risk was associated with a prior history of psychiatric disorders. It is not possible to determine which of the increased adverse events are specific to verubecestat and which represent a class effect. Future trials of other BACE inhibitors may shed light on this issue. Differences between selective BACE1 inhibitors and combined BACE1/BACE2 inhibitors such as verubecestat may emerge. Although there were some dose-related numerical differences in the incidence rate of some adverse events, no statistically significant exposure dependencies were observed in the exposure-response analysis, suggesting that the adverse event profile is similar across exposures from 12 to 40 mg. Data from the APECS trial of verubecestat in prodromal AD showed a broadly similar safety and tolerability profile to that reported here [36].

## Additional files

Additional file 1: Trial protocol. (PDF 10847 kb)

Additional file 2: **Table S1.** Number (%) of Participants Exceeding the Predefined Limits of Change in Vital Signs and ECG Measurements. (DOCX 21 kb)

Additional file 3: **Table S2.** Number of Participants with Liver Function Laboratory Findings that Met Predetermined Criteria. (DOCX 21 kb)

Additional file 4: **Table S3.** Number (%) of Participants Exceeding the Predefined Limits of Change in Laboratory Parameters. (DOCX 36 kb)

Additional file 5: **Table S4.** Mixed-Model Analyses for Neuropsychiatric Inventory Total Score. (DOCX 19 kb)

## Data Availability

MSD’s data sharing policy, including restrictions, is available at http://engagezone.msd.com/ds_documentation.php. Requests for access to the clinical trial data can be submitted through the EngageZone site or via email to dataaccess@merck.com.

## Abbreviations

AD: Alzheimer’s disease
APP: Amyloid precursor protein
ARIA: Amyloid-related imaging abnormality
AUC: Area under the curve
Aβ: Amyloid-β
BACE1: β-Site amyloid precursor protein cleaving enzyme 1
BACE2: β-Site amyloid precursor protein cleaving enzyme 2
C-SSRS: Columbia Suicide Severity Rating Scale
ECG: Electrocardiogram
ECI: Event of clinical interest
MedDRA: Medical Dictionary for Regulatory Activities
MMSE: Mini-Mental State Exam
NPI: Neuropsychiatric Inventory
