# Supplementary material for: Further analyses of the safety of verubecestat in the phase 3 EPOCH trial of mild-to-moderate Alzheimer’s disease
Source: Alzheimers Res Ther. 2019 Aug 7;11:68. doi: 10.1186/s13195-019-0520-1 (PMC6685277; doi:10.1186/s13195-019-0520-1)
Supplement: Supplementary file 1 — Trial protocol. (PDF 10847 kb) [file 13195_2019_520_MOESM1_ESM.pdf]

Merck & Co., Inc. policy on posting of redacted study protocols on journal websites is described in Guidelines for Publication of Clinical Trials in the Scientific Literature on the www.merck.com website.

For publicly posted protocols, the Company redacts content that contains proprietary and private information.

This report may include approved and non-approved uses, formulations, or treatment regimens. The results reported may not reflect the overall profile of a product. Before prescribing any product mentioned in this report, healthcare professionals should consult local prescribing information for the product approved in their country.

Copyright © 2019 Merck Sharp & Dohme Corp., a subsidiary of Merck & Co., Inc.

All Rights Reserved. Not for regulatory or commercial use.

1.0 TITLE PAGE

Abbreviated Title	An Efficacy and Safety Trial of MK-8931 in Mild to Moderate AD (EPOCH)
Title	A Randomized, Placebo Controlled, Parallel-Group, Double Blind Efficacy and Safety Trial of MK-8931 with a Long Term Double-Blind Extension in Subjects with Mild to Moderate Alzheimer's Disease. (Protocol No. MK-8931-017-13) (also known as SCH 900931, P07738)
Sponsor	Merck Sharp & Dohme Corp., a subsidiary of Merck & Co., Inc.
Sponsor's Address	2000 Galloping Hill Road<br>Kenilworth, New Jersey 07033, U.S.A.
IND No.	110,186
EudraCT No.	2011-003151-20
Trial Physician/Director	PPD [redacted]
Phase	2/3
Date of Finalization of This Current Version of the Protocol	28-JUN-2016 – Amendment No.17
Previous Versions of the Protocol	**11-SEP-2015**--Amendment 017-16 (Country Specific)<br>**26-JUN-2015**--Amendment 017-15 (Country Specific)<br>**24-APR-2015**--Amendment 017-14 (Country Specific)<br>**09-APR-2015** – Amendment 017-13<br>**05-MAR-2015** – Amendment 017-12 (Country Specific)<br>**26-FEB2015** – Amendment 017-11 (Country Specific)<br>**18 DEC 2014** – Amendment 017-10<br>**08 AUG 2014** - Amendment 017-09<br>**11 FEB 2014** – Amendment #5 (017-08)<br>**14 AUG 2013** – Amendment #4 (017-07)<br>**08 NOV 2012** – Amendment #3 Brazil Version 1 (017-06)<br>**26 SEP 2012** – Amendment #3 (017-05)<br>**23 JUL 2012** – Amendment #2 Brazil Version 2 (017-04)<br>**23 JUL 2012** – Amendment #2 Brazil Version 1 (017-03)<br>**20 JUN 2012** - Amendment #2 (017-02)<br>**31 MAY 2012** - Amendment #1 (017-01)<br>**19 JAN 2012** - Initial Protocol (017-00)
Protocol Template Approval Date	28 MAR 2012

CONFIDENTIAL

TRIAL PROTOCOL

THIS CONFIDENTIAL INFORMATION ABOUT AN INVESTIGATIONAL DRUG OR PRODUCT IS PROVIDED FOR THE EXCLUSIVE USE OF INVESTIGATORS OF THIS DRUG OR PRODUCT AND IS SUBJECT TO RECALL AT ANY TIME. THE INFORMATION IN THIS DOCUMENT MAY NOT BE DISCLOSED UNLESS SUCH DISCLOSURE IS REQUIRED BY APPLICABLE LAW OR REGULATIONS. SUBJECT TO THE FOREGOING, THIS INFORMATION MAY BE DISCLOSED ONLY TO THOSE PERSONS INVOLVED IN THE TRIAL WHO HAVE A NEED TO KNOW, WITH THE OBLIGATION NOT TO FURTHER DISSEMINATE THIS INFORMATION. THESE RESTRICTIONS ON DISCLOSURE WILL APPLY EQUALLY TO ALL FUTURE ORAL OR WRITTEN INFORMATION SUPPLIED TO YOU BY THE SPONSOR OR ITS AFFILIATES OR REPRESENTATIVES THAT IS DESIGNATED AS "PRIVILEGED" OR "CONFIDENTIAL".

THIS PROTOCOL AMENDMENT AND ALL OF THE INFORMATION RELATING TO IT ARE CONFIDENTIAL AND PROPRIETARY PROPERTY OF MERCK SHARP & DOHME CORP., A SUBSIDIARY OF MERCK & CO., INC., WHITEHOUSE STATION, NJ, U.S.A.

SUMMARY OF CHANGES

PRIMARY REASON(S) FOR THIS AMENDMENT:

Section Number(s)	Section Title(s)	Description of Change(s)	Rationale
2.1.2<br><br>2.2.2<br><br>11.1.2<br><br>11.6.5	Trial Diagram for Main Cohort (Part I)<br><br>Trial Flow Chart for Main Cohort (Part I)<br><br>Extension Trial Flow Chart<br><br>Extension Procedures	Reinstated the collection of plasma pharmacokinetic (PK) samples in addition to dried blood spot (DBS) PK samples and added collections at Visits 9 and 10 (Part I) and 11 and 12 (Part II).	Both plasma and DBS samples were collected initially for assessment of PK, but plasma sampling was dropped when preliminary analyses indicated that DBS samples were sufficient. However, due to quality issues identified with some DBS samples, plasma sampling is now added back in. Furthermore, two additional PK samples are added in both Part I and Part II to substitute for some of the collected DBS samples with quality issues. These additional samples will facilitate PK and PK/PD modeling.
8.2.9	Interim Analysis	Modified criteria for futility interim analysis	Protocol PN017 is the first, large Phase 3 trial to evaluate an agent that has been shown to robustly reduce Aβ production. Therefore, it will be important to obtain as complete a data set as possible to fully evaluate the possibility that specific subgroups may respond to treatment. Emergent evidence from other trials in the AD field suggests, for example, that anti-amyloid therapy may provide increased efficacy in milder AD sub-populations, as compared to the more general mild-moderate AD population being studied in PN017.

Section Number(s)	Section Title(s)	Description of Change(s)	Rationale
			This amendment will change the existing protocol-specified futility criteria to make the probability of stopping the trial for futility in all study participants less likely. Provided the safety profile is acceptable, the modified criteria will facilitate the collection of a more complete data set for examining efficacy in study participant subgroups (e.g. mild AD, ApoE4 carriers, etc).
8.2.10	Accounting for Missing Data	For the Tipping Point sensitivity analysis, stated intention to calibrate between the primary ANCOVA model and the multiple imputation model, if needed.	The tipping point sensitivity analysis is intended to report the value *c* that would need to be applied to all multiply-imputed values from the active arms, to turn a significant result, non-significant. However, it is possible that a slight difference may exist between the primary ANCOVA model and the multiple imputation (MI) model (absent any adjustment to the active arms) for the final observed dataset. Such a difference would bias the intended interpretation of *c* (i.e. some small fraction of c would represent the difference between the models, as opposed to the entirety of *c* representing the detrimental effect needed to be applied to the active arms to tip the significant result to non-significant.)

Section Number(s)	Section Title(s)	Description of Change(s)	Rationale
11.2.1	Rationale for Trial Extension	Added note that Sponsor will be unblinded following the completion of Part I but some Sponsor personnel will remain blinded during Part II, as necessary, to support the conduct of the trial and the collection of efficacy data.	Maintaining Sponsor blinding for efficacy data collected at 24 months in Part II is needed to support the primary efficacy objective for Part II .

ADDITIONAL CHANGE(S) FOR THIS AMENDMENT:

Section Number(s)	Section Title(s)	Description of Change(s)	Rationale
2.2.2	Trial Flow Chart for Main Cohort (Part I)	Added footnote "i" to Ophthalmology Visit at Visit 10 column.	Editorial change
7.7.2.2.3	Events of Clinical Interest	Added clarification that the ECI events listed in this section are same for main study and extension study.	Clarification
7.7.2.2.3	Events of Clinical Interest	Since routine MRI monitoring for safety has been discontinued, the central Reading Center is not automatically performing central reading for safety. Therefore, during the main study and the extension, the site investigator or radiologist should perform a local reading, as necessary. In some cases, central reads for safety may be performed. In the event that an ARIA is detected, then the Sponsor may request that the MRI be submitted for central reading.	Clarification

2.0 SYNOPSIS

TITLE OF TRIAL: A Randomized, Placebo Controlled, Parallel-Group, Double Blind Efficacy and Safety Trial of MK-8931 with a Long Term Double-Blind Extension in Subjects with Mild to Moderate Alzheimer's Disease. (Protocol No. MK-8931-017-13) (also known as SCH 900931, P07738)

ABBREVIATED TITLE: An Efficacy and Safety Trial of MK-8931 in Mild to Moderate AD (EPOCH)

OBJECTIVES:

Part I: Primary Trial Objectives:

1. To assess the efficacy of two doses of MK-8931 on cognition in subjects with mild to moderate AD.
2. To assess the efficacy of two doses of MK-8931 on functional ability in activities of daily living in subjects with mild to moderate AD.
3. To assess the safety and tolerability of three doses of MK-8931 in the treatment of subjects with mild to moderate AD.

Part I: Secondary Trial Objective:

To assess the overall clinical response, as reflected by global assessment, of two doses of MK-8931 in subjects with mild to moderate AD.

Part II: Primary Extension Trial Objectives

1. To evaluate the safety and tolerability of MK-8931 in the long term treatment of mild to moderate Alzheimer's Disease.
2. To compare the efficacy of MK-8931 on cognition and functional ability in activities of daily living in subjects with mild to moderate AD in subjects administered MK-8931 for 24 months to that of subjects administered placebo for 18 months followed by MK-8931 for 6 months.

Part II: Exploratory Extension Trial Objective:

To compare the efficacy of MK-8931 administered to subjects for 18 months to that of subjects administered placebo for 18 months in Part I followed by long term treatment of MK-8931 in Part II on cognition, function, disease progression, and health economic burden at multiple time points.

Trial Design

Overview:
This study is comprised of two parts. Part I refers to the initial 78-week treatment period. Part II refers to the extension period (up to approximately 260 weeks) which will be available to subjects who complete Part I. Details for Part I are provided in Sections 5 to 8 of this protocol. Details for Part II are provided in Section 11 of this protocol.

NOTE subject participation in Part II is optional and Part II will only be conducted in areas where approved by local authorities.

Part I is a 78-week double blind, placebo controlled trial to evaluate the efficacy of β-site amyloid precursor protein (APP) cleaving enzyme (BACE) inhibitor MK-8931 as a potential disease-modifying therapy in subjects with mild to moderate AD. The trial is powered to detect a clinically significant change in the two coprimary outcome measures (the Alzheimer's Disease Assessment Scale Cognitive subscale [ADAS-Cog] and the Alzheimer's Disease Cooperative Study Activities of Daily Living Inventory [ADCS-ADL] change-from-baseline scores) at Week 78.

At the end of the 78-week Treatment Period of Part I, subjects who have completed treatment may choose to participate in the extension trial (Part II), during which all subjects who received placebo in Part I will receive active drug. Part II will start with enrollment of the first subject who completes Part I and chooses to participate in the extension. Part II of the study will end when the drug either becomes commercially available or when the MK-8931 program is terminated. It is expected that Part II will have a duration of up to approximately 260 weeks (5 years) for the first subject enrolled.

Safety Cohort (Part I): To provide a further assessment of the safety and tolerability of MK-8931 during chronic treatment prior to allowing unrestricted enrollment, the first 200 subjects (~50 subjects randomized to one of three doses of MK-8931 [12, 40 and 60 mg] or placebo) will be monitored more frequently during their first 13 weeks of treatment. A limited number of sites (approximately 75-125) are expected to recruit these subjects during the initial 6 to 8 months of the trial. When these 200 subjects have had the opportunity to complete 13 weeks of treatment, the first formal interim safety analysis will be performed by an external data

TITLE OF TRIAL: A Randomized, Placebo Controlled, Parallel-Group, Double Blind Efficacy and Safety Trial of MK-8931 with a Long Term Double-Blind Extension in Subjects with Mild to Moderate Alzheimer's Disease. (Protocol No. MK-8931-017-13) (also known as SCH 900931, P07738)

monitoring committee (**eDMC**), which may recommend dropping the more frequent safety monitoring (see Section 2.2).

Enrollment will continue at those initial sites after the first 200 subjects have been randomized but prior to the first formal interim safety analysis. These additional subjects will also undergo the more frequent safety monitoring schedule. This continued enrollment will yield data of less than 13 weeks of treatment on approximately 200 additional subjects which will supplement the safety data for the interim analysis. The two groups enrolled prior to the first formal interim safety analysis (approximately 400 in all) will be designated as the leading **Safety Cohort**.

The remainder of the sites (~85-115) will begin screening subjects for the **Main Cohort** (which includes all subjects enrolled after the first formal safety interim analysis) while awaiting a favorable recommendation of the eDMC to continue the trial (~9 to 12 months after the study has started). Initial sites for the Safety Cohort can also screen subjects for the Main Cohort around the same time. Main Cohort subjects may only be randomized after the eDMC's review and approval.

Trial Governance: An independent eDMC will have the primary responsibility for monitoring safety throughout both parts of the trial. In addition, at the first formal safety interim analysis, a standing internal DMC (**siDMC**) will recommend whether or not to continue the 12 and 40 mg doses into the Main Cohort (Part I). At this first interim analysis, both the siDMC and the eDMC will assess unblinded 3-month data from Subjects 1-200, with the eDMC also having access to data from the additional ~200 Safety Cohort subjects (Subjects 201-400). The 60 mg dose will not continue into the Main Cohort as both statistical analyses as well as modeling and simulation analyses (based on biomarker results in AD subjects external to this trial data) suggest that the 60mg dose offers negligible advantages in amyloid β (Aβ) lowering. The 12 mg and/or the 40 mg doses may move into the Main Cohort depending on a review of the Safety Cohort data by the governance committees. Based on the siDMC recommendation, as well as on their own assessment of safety, the eDMC will inform the blinded Merck Executive Oversight Committee (EOC) as to whether the 12 and 40 mg doses should continue on into the Main Cohort. Since the first 200 subjects will be excluded from the primary efficacy and safety analyses, the siDMC will periodically review analyses pertaining to these first 200 subjects throughout the trial. Additional details pertaining to the specific roles of the eDMC and siDMC will be specified in the respective committee charters.

Treatment Arms:

Part I: Initially, three dose arms and placebo will be included in the Safety Cohort. The 60 mg dose arm will not continue in the Main Cohort. Approval from the siDMC and eDMC is required for the 12 and 40 mg arms to continue in the Main Cohort; it is possible that the siDMC and eDMC could select only one dose, depending on the review of the safety data at the first formal interim safety analysis. If so, the sample size requirements will be updated as described in Section 8.2.9. **For clarity and simplicity, the protocol is written assuming that the 12 and 40 mg dose arms will be included in the Main Cohort.**

Part II: All active doses from Part I will be carried forward into Part II, with all subjects on active treatment continuing on their same treatment arm for Part II. Subjects originally on placebo will be assigned to the 40 mg dose. Sites and subjects will remain blinded during Part II. The Sponsor will be unblinded following the completion of Part I. Some Sponsor personnel will remain blinded during Part II, as necessary, to support the conduct of the trial and the collection of efficacy data.

Sample Size:

Part I: Factoring in the expected 18-month dropout rate, approximately 1710 subjects (570/arm) need to be enrolled in each of the three treatment groups to maintain an overall study power of 90%. The siDMC will review safety data (in addition to ADAS-Cog results to evaluate cognitive worsening) from the first 200 subjects. Due to this internal unblinding, these first 200 subjects will be excluded from the primary efficacy and safety analyses. Thus the total number of subjects that need to be enrolled in the trial will be approximately 1960 (1710 to be included in for the efficacy analysis, plus ~100 for the dropped dose arm, and the first 150 from the retained dose arms of the Safety Cohort). Both the percent of mild AD subjects and the percent of moderate AD subjects, respectively, will be no more than 60% of the total. Subjects in the Main Cohort (defined as all subjects enrolled after the first formal safety interim analysis) will be randomized to one of the three remaining treatment groups (the 12 and 40 mg dose arms or placebo). All Safety Cohort subjects in the 12 mg and 40 mg dose arms enrolled after the first 200 subjects will be included in the efficacy and safety analyses (since the siDMC will not have access to any of the data arising from these latter Safety Cohort subjects).

**TITLE OF TRIAL:** A Randomized, Placebo Controlled, Parallel-Group, Double Blind Efficacy and Safety Trial of MK-8931 with a Long Term Double-Blind Extension in Subjects with Mild to Moderate Alzheimer's Disease. (Protocol No. MK-8931-017-13) (also known as SCH 900931, P07738)
Subjects from the Safety Cohort who were originally assigned to the dropped MK-8931 dose arm will be switched to the higher remaining MK-8931 dose level for the remainder of the trial (approximately 100 subjects). These subjects will not be included in the primary efficacy or safety analyses, though they will be included in supportive safety analyses.<br><br>Part II: There are no sample size calculations for Part II, as there are no formal Part II hypotheses. Subjects who complete Part I will be eligible for Part II as described in Section 11.5. It is expected that 70% of subjects will complete Part I, and it is estimated that 90% of those will enter Part II, with (1960* 0.70*0.90) 1235 subjects projected to enter Part II.<br><br>**Substudies**: Two separate substudies will be conducted during Part I. The first will include evaluation of cerebrospinal fluid (CSF) biomarkers (eg, tau and Aβ) in both the Safety Cohort and the Main Cohort. All subjects enrolled at sites that are qualified and willing to perform a lumbar puncture will be eligible to participate in the CSF biomarkers substudy. The second will use positron emission tomography (PET) imaging to assess brain amyloid load in the Main Cohort only. Up to 100 subjects per arm will be enrolled in the PET substudy. Participation in these substudies is not required for participation in the trial.
**Number of Trial Centers:** Approximately 190-210.
**Duration of Participation:**<br><br>Part I: Each subject will participate in the initial 78-week Treatment Period for approximately 20 months from the time the Informed Consent Form (ICF) is signed through the final contact. After a screening phase of up to 4 weeks, each subject will receive treatment for 78 weeks (18 months).<br><br>Part II: Subjects who have completed the 78 weeks of treatment of Part I and tolerated study medication may choose to participate in the trial extension (Part II) which has an expected maximum duration of approximately 260 weeks, with the duration of individual subject participation dependent on the timing of enrollment in Part II. Subjects who did not complete Part I of the study or who repeatedly deviated from the protocol will not be permitted to continue in Part II. In addition, subjects who are less than 75% compliant with trial medication in Part I will require Sponsor approval to participate in Part II.
**Duration of Trial:**<br><br>Part I: The duration of the initial 78-week trial period is expected to be approximately four years from the beginning to the end of the overall trial (first subject signing informed consent to last contact with last subject).<br><br>Part II: The duration of the trial extension is expected to be up to approximately 5 years (first subject signing informed consent to last contact with last subject).
Key Inclusion Criteria:<br><br>Part I: Each subject must fulfill ALL the criteria listed below for entry<br>Each subject must be ≥ 55 to ≤ 85 years of age at the first visit.<br><br>Each subject must meet the criteria for a diagnosis of probable AD based on both a) the National Institute of Neurological and Communicative Diseases and Stroke/Alzheimer's Disease and Related Disorders Association (NINCDS-ADRDA) criteria and b) the Diagnostic and Statistical Manual of Mental Disorders, 4th Edition, Text Revision (DSM-IV-TR) criteria for AD.<br><br>Each subject must have a Mini-Mental State Examination (MMSE) score ≥ 15 and ≤ 26 at Screening.<br><br>Each subject must have a clear history of cognitive and functional decline over at least 1 year that is either a) documented in medical records or b) documented by history from an informant who knows the subject well.<br><br>Each subject must have a Magnetic Resonance Imaging (MRI) scan at the Screening Visit that is consistent with a diagnosis of AD. MRI scans or head CT (with Sponsor approval) obtained within 12 months of screening are acceptable alternatives.<br><br>Each subject must be able to read at a 6th grade level or equivalent, as determined by the investigator, and must have a history of academic achievement and/or employment sufficient to exclude mental retardation.<br><br>If a subject is receiving an acetylcholinesterase inhibitor, memantine, medical food/supplement (eg Vitamin E), and/or herbal medications for AD, the dose must have been stable for at least 3 months before Screening, and the subject must be willing to remain on the same dose for the duration of the trial. Subjects may need to be on AD treatments in accordance with local requirements. (The treatment and dose at Screening must not be

TITLE OF TRIAL: A Randomized, Placebo Controlled, Parallel-Group, Double Blind Efficacy and Safety Trial of MK-8931 with a Long Term Double-Blind Extension in Subjects with Mild to Moderate Alzheimer's Disease. (Protocol No. MK-8931-017-13) (also known as SCH 900931, P07738)

changed during the trial unless medically necessary. Additional treatments [including herbal medications] for AD that are not specified in the protocol must not be initiated during the trial.)

Each subject must have a trial partner who is reliable and competent. The trial partner must have a close relationship with the subject, have face to face contact at least 3 days/wk for a minimum of 6 waking hours/wk (or more, based on local requirements), be willing to accompany the subject to all required trial visits, and be willing to monitor compliance of the administration of the trial medication. The trial partner should understand the nature of the trial and adhere to trial requirements (eg, dose, visit schedules, and evaluations). It is recommended that the trial partner accompany the subject to all trial visits.

Each subject must have results of clinical laboratory tests (complete blood count [CBC], blood chemistries, thyroid stimulating hormone [TSH], and urinalysis) within normal limits or clinically acceptable to the investigator at Screening.

Each subject must have results of a physical examination, vital signs, and electrocardiogram (ECG) within normal limits or clinically acceptable to the investigator at Screening.

Part II:

Each subject must have tolerated study medication and completed the initial 78-week period of the trial. Subjects who did not complete the initial 78 weeks of treatment but continued in the trial may be permitted to continue in the extension at the discretion of the Sponsor.

Each subject must have a trial partner who is reliable, competent, and meets the same criteria outlined for Part I.

Key Exclusion Criteria:

Part I:

A subject meeting any of the exclusion criteria listed below must be excluded from participating in the trial:

The subject has a Rosen-modified Hachinski Ischemia Score > 4 at Screening (ie, evidence of vascular dementia).

The subject has a known history of stroke or evidence from screening MRI or CT scan that is clinically important in the investigator's opinion.

The subject has evidence of a clinically relevant neurological disorder other than the disease being studied (ie, probable AD) at Screening, including but not limited to: vascular dementia, parkinsonism, frontotemporal dementia, Huntington's disease, amyotrophic lateral sclerosis, multiple sclerosis, progressive supranuclear palsy, neurosyphilis, dementia with Lewy bodies, posterior cortical atrophy, logopenic primary progressive aphasia, other types of dementia, mental retardation, hypoxic cerebral damage, cognitive impairment due to other disorders, or head trauma with loss of consciousness that led to persistent cognitive deficits.

The subject has evidence of a clinically relevant or unstable psychiatric disorder, based on DSM-IV-TR criteria, including schizophrenia or other psychotic disorder, bipolar disorder, major depression, or delirium. Major depression in remission is not exclusionary.

The subject has evidence of a current episode of major depression based on investigator's judgment. A score on the 15-item Geriatric Depression Scale of 5 or more requires an assessment by an appropriate health care professional to evaluate for the presence of major depression. Subjects with a score of 5 or more who are not diagnosed with major depression following such an assessment may be included in the trial.

The subject's MRI scan obtained at Screening shows evidence of a neurological disorder other than probable AD or:

- evidence of a prior macrohemorrhage,
- Symptomatic vasogenic edema in the investigator's judgment,
- >3 lacunar infarcts over 10 mm each, or
- any other clinically significant finding that may account for their cognitive impairment, including but not limited to brain tumor, large or strategically located cortical or subcortical infarct, or severe white matter disease equaling a rating of 3 on the age-related white matter changes (ARWMC) scale,

TITLE OF TRIAL: A Randomized, Placebo Controlled, Parallel-Group, Double Blind Efficacy and Safety Trial of MK-8931 with a Long Term Double-Blind Extension in Subjects with Mild to Moderate Alzheimer's Disease. (Protocol No. MK-8931-017-13) (also known as SCH 900931, P07738)

An MRI that was done within 12 months before the Screening visit that is available for review by the central MRI reading vendor is also acceptable for evaluation of the eligibility criteria. A head CT scan may be accepted instead of MRI on a case-by-case basis, as approved by the Sponsor (e.g., when MRI is contraindicated for the subject).

The subject has a history of hepatitis or liver disease that, in the opinion of the investigator, has been active within the 6 months prior to Screening.

The subject has a recent or ongoing, uncontrolled, clinically significant medical condition within 3 months of the Screening Visit (such as, but not limited to, diabetes, hypertension, thyroid or endocrine disease, congestive heart failure, angina, cardiac or gastrointestinal disease, dialysis, or abnormal renal function with estimated creatinine clearance < 30 mL/min) other than the condition being studied such that, in the judgment of the investigator, participation in the trial would pose a significant medical risk to the subject. Controlled co-morbid conditions (including diabetes, hypertension, heart disease, etc) are not exclusionary, if stable within 3 months of the Screening Visit. All concomitant medications, supplements (eg Vitamin E),, or other substances must be kept as stable as medically possible during the trial.

The subject has a history or current evidence of long QT syndrome, QTC interval ≥ 470 milliseconds (for male subjects) or ≥ 480 milliseconds (for female subjects), or torsades de pointes. (Note: Determination of QTc interval at Screening will be based on the average of three measurements, using the Fridericia formula for correction.) Subjects with stable bundle branch block who exceed these limits for QTc interval are eligible for the trial if judged by an expert in cardiology not to be at increased risk for Torsades.

The subject has a history of malignancy occurring within the 5 years immediately before Screening, except for a subject who has been adequately treated for

1. basal cell or squamous cell skin cancer,
2. in situ cervical cancer, or
3. localized prostate carcinoma; or
4. who has undergone potentially curative therapy with no evidence of recurrence for ≥ 3 years post-therapy, and who is deemed at low risk for recurrence by her/his treating physician.

The subject has

1. a history of clinically significant vitamin B12 or folate deficiency in the 6 months immediately before Screening, or
2. vitamin B12 or folate deficiency in addition to increased serum homocysteine or methylmelonic acid levels at Screening as determined by central laboratory normal values.

The subject has received any of the treatments listed in Table 1 more recently than the indicated period before Screening. See Section 7.3.2.

Part II:

The subject is at imminent risk of self-harm, based on clinical interview or on the Columbia Suicidality Severity Rating Scale (C-SSRS), or of harm to others in the opinion of the investigator. Subjects must be excluded if they report suicidal ideation with intent, with or without a plan (eg, suicidal ideation item 4 or 5 on the C-SSRS) in the past 1 month or suicidal behavior in the past 6 months.

The subject has developed a recent or ongoing, uncontrolled, clinically significant medical condition (such as, but not limited to, diabetes, hypertension, thyroid or endocrine disease, congestive heart failure, angina, cardiac or gastrointestinal disease, dialysis, or abnormal renal function with estimated creatinine clearance < 30 mL/min) other than Alzheimer's disease such that, in the judgment of the investigator, participation in the trial would pose a significant medical risk to the subject. Controlled co-morbid conditions (including diabetes, hypertension, heart disease, etc) are not exclusionary if stable. All concomitant medications, supplements (eg Vitamin E), or other substances must be kept as stable as medically possible during the trial.

Note: urinary tract infections at Visit 10B are not exclusionary if adequately treated (as documented by repeat urinalysis).

The subject has a history of, or has developed during Part I evidence of long QT syndrome, QTC interval ≥ 470

TITLE OF TRIAL: A Randomized, Placebo Controlled, Parallel-Group, Double Blind Efficacy and Safety Trial of MK-8931 with a Long Term Double-Blind Extension in Subjects with Mild to Moderate Alzheimer's Disease. (Protocol No. MK-8931-017-13) (also known as SCH 900931, P07738)

milliseconds (for male subjects) or ≥ 480 milliseconds (for female subjects), or torsades de pointes. Subjects with stable bundle branch block who exceed these limits for QTc interval are eligible for the trial if judged by an expert in cardiology not to be at increased risk for Torsades.

The subject anticipates receiving any of the treatments listed in Table 13 during Part II.

The subject has developed a form of dementia that is not Alzheimer's disease, including but not limited to, dementia due to HIV infection, head trauma, vascular disease, Parkinson's disease, frontotemporal dementia, or Huntington's disease, as determined by the investigator.

INVESTIGATIONAL PRODUCT, DOSE, MODE OF ADMINISTRATION

Investigational Product:

Part I :

- MK-8931 will be supplied as tablets of 12 mg, 40 mg and 60 mg. As assigned per the randomization, subjects enrolled in the Safety Cohort will receive one of the following dosing regimens:

1. MK-8931 12 mg orally once daily (QD),
2. MK-8931 40 mg orally QD, or
3. MK-8931 60 mg orally QD.

Subjects enrolled in the Main Cohort will receive, per the randomization, either the 12 or 40 mg dose. Subjects in the Safety Cohort who initially received the 60 mg dose prior to the first formal interim safety analysis will be switched to the remaining higher dose for the remainder of the trial.

Part II:

MK-8931 will be supplied as tablets of 12 mg and 40 mg . All active doses from Part I will be carried forward into Part II, with all subjects on active treatment continuing on their same treatment arm for Part II. Subjects originally on placebo will be assigned to 40 mg:

1. MK-8931 12 mg orally once daily (QD) - 12/12 treatment group:
2. MK-8931 40 mg orally once daily (QD) - 40/40 and placebo/40 treatment groups:

Reference Product: Matching placebo tablets: As assigned per the randomization, subjects will receive matching placebo tablets orally QD throughout the trial.

STATISTICAL METHODS:

Data Sets to be Analyzed:

- The Full Analysis Set (FAS) population will serve as the primary population for the analysis of efficacy data in this trial. In order to better adhere to the intent-to-treat principle, subjects will be encouraged to continue in the trial even if they discontinue treatment (often referred to as "retrieved dropout").
- The All-Patients-as-Treated (APaT) population will be used for the analysis of safety data in this trial. The APaT population consists of all randomized subjects who received at least one dose of trial treatment, with subjects included in the treatment group corresponding to the trial treatment they actually received.
- It is noted that the first 200 subjects enrolled will be excluded from the primary efficacy and safety analyses, since the siDMC will have access to unblinded data from these subjects in advance of a dose decision.

Sample Size (Part I):

Sample sizes were calculated to achieve 90% overall power to demonstrate significance of both Coprimary Efficacy Endpoints for at least one dose. The required number of randomized subjects, after accounting for a 5.8% dropout rate every 13 weeks, (ie, a 30% cumulative dropout at 78 weeks), and assuming a drug effect of 35% for both of the 12 and 40 mg doses (corresponding to a roughly 2-point difference for ADAS-Cog and a 3.4-point difference for ADCS-ADL with 18-month placebo progression rates estimated primarily from a manuscript from Schneider and Sano [2009]) is 570 subjects per arm (1710 in total). The total number of subjects to be randomized in the study is approximately 1960 (1710 contributing to the analysis, the first 150 Safety Cohort subjects from the retained dose arms, and approximately 100 subjects from the dropped dose arm).

**TITLE OF TRIAL:** A Randomized, Placebo Controlled, Parallel-Group, Double Blind Efficacy and Safety Trial of MK-8931 with a Long Term Double-Blind Extension in Subjects with Mild to Moderate Alzheimer's Disease. (Protocol No. MK-8931-017-13) (also known as SCH 900931, P07738)
**Efficacy Analysis:**<br>Part I: The Primary Hypotheses will be tested on the basis of the two Coprimary Endpoints, the 78-week change from Baseline in ADAS-Cog score and the 78-week change from Baseline in ADCS-ADL score.<br>The primary analysis approach will be conducted separately on each of the Coprimary Endpoints and will utilize a longitudinal ANCOVA model. Time is treated as a categorical variable so that no restriction is imposed on the trajectory of the means over time. The analysis model will also adjust for the categorical factors of geographic region, treatment, gender, baseline use of Vitamin E (0-400 IU/day, > 400 IU /day), Apolipoprotein E (APOE) genotype (APOE 4 positive, APOE 4 negative), Baseline AD medication (use of acetylcholinesterase inhibitor [AChEI] and/or memantine, vs. no use), study cohort (Safety Cohort, Main Cohort) and the interaction of time-by-treatment, with the Baseline values of MMSE and age included as continuous covariates. The baseline value of the dependent variable, as well as the baseline-by-time interaction term will also be included. The Week-78 change-from-Baseline mean treatment differences (MK-8931 – placebo), corresponding 95% confidence intervals (CIs), and p-values will be estimated from this model. An unstructured covariance matrix will be used to model the correlation among repeated measurements.<br>The analysis of the Key Secondary Endpoint, Clinical Dementia Rating Sum of Boxes (CDR-SB), will be conducted in a manner similar to that used for the Coprimary Endpoints. Disease modification will be assessed using an analysis of clinical endpoints at different time points and using *in vivo* biomarkers that assess key features to AD pathology.<br>Part II: There are no formal hypotheses in Part II. All efficacy measurements analyzed in Part I will continue to be collected and analyzed in Part II. The primary Part II endpoint is the 24-Month (6 months after the primary timepoint from Part I) change-from-baseline treatment difference on ADAS-Cog and ADCS-ADL.
**Safety Analysis (Part I and Part II):** Safety and tolerability will be assessed by a clinical review of all relevant parameters including adverse events (AEs), laboratory tests, vital signs, and ECG measurements.<br>The analysis of safety results will follow a tiered approach. The tiers differ with respect to the analyses that will be performed. Safety parameters or AEs of special interest that are identified a priori constitute "Tier 1" Safety Endpoints that will be subject to inferential testing for statistical significance with p-values and 95% CIs provided for between-group comparisons. These include: 1) microhemorrhage, superficial siderosis or macrohemorrhage; 2) vasogenic edema; 3) delirium; and 4) rash Events of Clinical Interest (ECI; see **Section 7.7.2.2.3**). All other safety parameters will be considered Tier 2 or Tier 3 Safety Endpoints. Tier 2 Safety Endpoints will be assessed via point estimates with 95% CIs provided for between-group comparisons. Tier 3 Safety Endpoints will be summarized using only point estimates by treatment group.
**Interim Analyses:** Three types of interim analyses (IAs) will be performed during this trial: 1) the first formal analysis for safety and dose selection on the Safety Cohort, 2) multiple ongoing safety analyses and 3) one futility analysis. Trial enrollment will continue during all IAs. The eDMC will be involved in all three types of interim analyses and will review unblinded safety data from all IAs. The primary responsibilities of the siDMC will be to review safety results from Subjects 1-200 at the first formal analysis for safety and approve the use of the 12 and 40 mg doses for the Main Cohort. At the time of the first formal safety IA both the siDMC and the eDMC will review limited unblinded analysis on the ADAS-Cog for the first 200 subjects only to evaluate the potential for cognitive worsening. For the subsequent ongoing safety analyses, ADAS-Cog analyses based on data from all available subjects will be presented to the eDMC only to monitor potential cognitive worsening. The siDMC will also periodically review unblinded results from Subjects 1-200 to evaluate safety. These reviews will include clinical endpoints from the first 200 subjects only. The siDMC's dose selection will be based on in-study safety data. Please refer to **Section 8.2.9** for a summary of the conduct, endpoints, timing, and purpose of the IAs.
**Pharmacogenetics:** The relationship between 1) APOE genotype and the response to therapy and 2) human leukocyte antigen (HLA) region genetic markers and rash will be evaluated as an exploratory outcome. Additional exploratory pharmacogenetic studies using other genetic markers may be performed, eg, if significant pharmacokinetic/pharmacodynamic (PK/PD) relationships are observed or AEs are identified. Pharmacogenetic studies will be conducted with PK results and clinical and biomarker measures.

2.1 Trial Design Diagram

2.1.1 Trial Diagram for Safety Cohort (Part I)

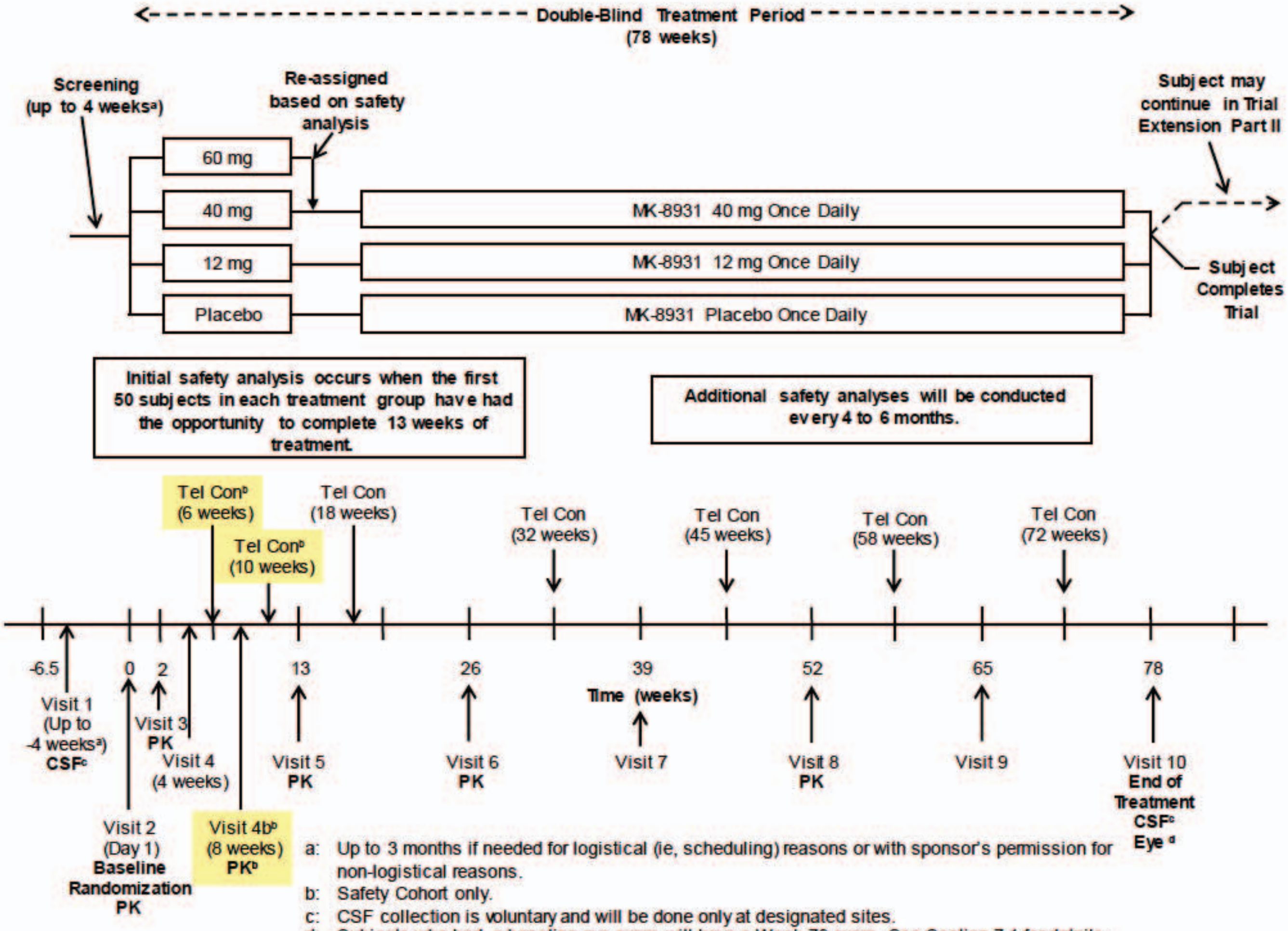

a: Up to 3 months if needed for logistical (ie, scheduling) reasons or with sponsor's permission for non-logistical reasons.
b: Safety Cohort only.
c: CSF collection is voluntary and will be done only at designated sites.
d: Subjects who had a baseline eye exam will have a Week 78 exam. See Section 7.1 for details.

CSF=Cerebrospinal Fluid; PK=Pharmacokinetics; Tel Con=Telephone Contact

2.1.2 Trial Diagram for Main Cohort (Part I)

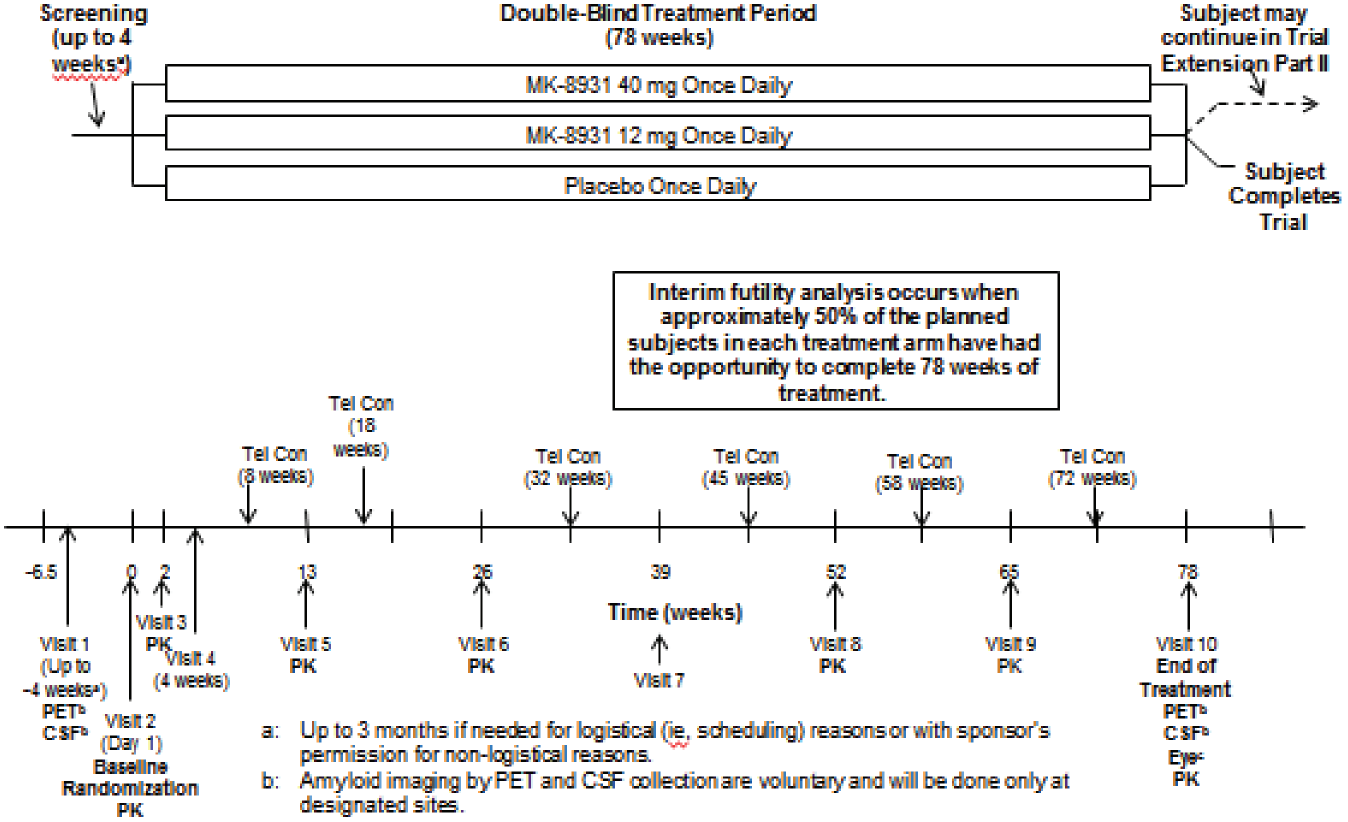

a: Up to 3 months if needed for logistical (ie, scheduling) reasons or with sponsor's permission for non-logistical reasons.

b: Amyloid imaging by PET and CSF collection are voluntary and will be done only at designated sites.

c: Subjects who had a baseline eye exam will have a Week 78 exam. See Section 7.1 for details

.

CSF=Cerebrospinal fluid; PET=Positron Emission Tomography; PK=Phamacokinetics; Tel Con=Telephone Contact

2.1.3 Trial Diagram for Trial Extension (Part II)

Refer to Section 11.1.1 for the Trial Diagram for Trial Extension Part II.

2.2 Trial Flow Charts

2.2.1 Trial Flow Chart for Safety Cohort (Part I)

Columns and cells highlighted in yellow and green refer to differences between the Safety Cohort and the Main Cohort.

NOTE: Blood and CSF samples should NOT be collected immediately prior to testing of clinical outcomes.

Safety Cohort Trial Period	Screen Period[a]	Double-Blind Treatment Period																			
		Baseline/ Randomization			Safety Cohort Only													EOT			
Visit Number	1	2	3	4	TC[b]	4b	TC[b]	5	TC[b]	6	TC[b]	7	TC[b]	8	TC[b]	9	TC[b]	10	TC[b,c]	UV	ETV
Scheduled Week	Up to – 4	(Study Day 1[d])	2	4	6	8	10	13	18	26	32	39	45	52	58	65	72	78	80		
Visit Window (Weeks)			±1	±1	±1	±1	±1	±1	±4	±4	±4	±4	±4	±4	±4	±4	±4	±4	+1		
Informed Consent	X																				
Informed Consents for Substudies[e]	X																				
Pharmacogenetic Testing Informed Consent[f]	X																				
Issue/Collect Subject Identification Card	X																	X			X
Medical History	X																				
Review Prior Medications	X																				
Record Concomitant Medications	X	X	X	X	X	X	X	X	X	X	X	X	X	X	X	X	X	X	X	X	X

Safety Cohort Trial Period	Screen Period[a]	Double-Blind Treatment Period																			
		Baseline/ Randomization			Safety Cohort Only													EOT			
Visit Number	1	2	3	4	TC[b]	4b	TC[b]	5	TC[b]	6	TC[b]	7	TC[b]	8	TC[b]	9	TC[b]	10	TC[b,c]	UV	ETV
Scheduled Week	Up to – 4	(Study Day 1[d])	2	4	6	8	10	13	18	26	32	39	45	52	58	65	72	78	80		
Visit Window (Weeks)			±1	±1	±1	±1	±1	±1	±4	±4	±4	±4	±4	±4	±4	±4	±4	±4	+1		
Structural MRI	X[g,h]																	X[h,i]		X[j]	X[h,i]
Vital Signs	X	X	X	X		X		X		X		X		X		X		X		X	X
Body Weight	X					X		X		X				X				X			X
Height	X																				
Physical & Neurological Examinations	X					X						X						X		X[j]	X
Full Body Skin Examination by Dermatologist[y]			X							X											
Directed Skin Examination by Site Physician								X						X				X			X
12-Lead Electrocardiogram[k]	X		X											X						X[j]	X
Hematology and Chemistry Blood Samples	X[l]		X			X		X		X		X		X				X		X[j]	X
Serum Pregnancy Test (βhCG) Blood Samples	X[m]																				
FSH Blood Samples	X[n]																				
APOE and HLA Genotyping Blood Samples		X[o]																			
Pharmacogenetic Blood Sample		X[f,o p]																			
PK/PD Blood Samples[w]		X[p]	X			X		X		X[t]				X						X[j]	X
Urinalysis	X		X					X		X		X		X				X		X[j]	X

Safety Cohort Trial Period	Screen Period[a]	Double-Blind Treatment Period																			
		Baseline/ Randomization			Safety Cohort Only													EOT			
**Visit Number**	1	2	3	4	TC[b]	4b	TC[b]	5	TC[b]	6	TC[b]	7	TC[b]	8	TC[b]	9	TC[b]	10	TC[b,c]	UV	ETV
**Scheduled Week**	Up to – 4	(Study Day 1[d])	2	4	6	8	10	13	18	26	32	39	45	52	58	65	72	78	80		
**Visit Window (Weeks)**			±1	±1	±1	±1	±1	±1	±4	±4	±4	±4	±4	±4	±4	±4	±4	±4	+1		
Modified Hachinski Ischemia Score	X																				
15-item Geriatric Depression Scale	X																				
Inclusion/Exclusion Criteria	X	X																			
ADAS-Cog	X[q]	X						X		X		X		X		X		X			X
ADCS-ADL		X						X		X		X		X		X		X			X
CDR-SB	X[r]	X[r]								X				X				X			X
Mini-Mental State Examination (MMSE)	X					X				X				X				X			X
Diagnostic Assessment and Narrative Summary[s]	X																				
Neuropsychiatric Inventory (NPI)		X				X		X		X				X				X			X
Modified Resource Utilization in Dementia (RUD) Lite[u]		X								X				X				X			X
Health Economic Assessment (HEA)		X								X				X				X			X
EuroQol Five Dimension Questionnaire (EQ-5D)		X								X				X				X			X
Columbia Suicide Severity Rating Scale (C-SSRS)[v]	X	X	X	X		X		X		X		X		X		X		X		X[j]	X

Safety Cohort Trial Period	Screen Period[a]	Double-Blind Treatment Period																			
		Baseline/ Randomization			Safety Cohort Only													EOT			
Visit Number	1	2	3	4	TC[b]	4b	TC[b]	5	TC[b]	6	TC[b]	7	TC[b]	8	TC[b]	9	TC[b]	10	TC[b,c]	UV	ETV
Scheduled Week	Up to – 4	(Study Day 1[d])	2	4	6	8	10	13	18	26	32	39	45	52	58	65	72	78	80		
Visit Window (Weeks)			±1	±1	±1	±1	±1	±1	±4	±4	±4	±4	±4	±4	±4	±4	±4	±4	+1		
CSF Collection[e]	X[g]																	X[i]			X[i]
Record Adverse Events	X	X	X	X	X	X	X	X	X	X	X	X	X	X	X	X	X	X	X	X	X
Dispense Trial Medication		X		X				X		X		X		X		X					
Administration of Trial Medication on Site		X								X[t]											
Medication Compliance			X	X	X	X	X	X	X	X	X	X	X	X	X	X	X	X		X	X
Drug Accountability Assessment			X	X		X		X		X		X		X		X		X		X	X
Ophthalmology Visit (details below)																		X[z]			
Visual Acuity																		X[z]			
Posterior Eye Exams																		X[z]			
Fundus Photography																		X[z]			
Fundus Autofluorescence[x]																		X[z]			
SD-OCT																		X[z]			
CAM		X --------------------To be completed only for adverse events of delirium (see 7.7.2.2.3).----------------X																			

βhCG=β-Human Chorionic Gonadotropin; ADAS-Cog=Alzheimer's Disease Assessment Scale Cognitive Subscale; ADCS-ADL=Alzheimer's Disease Cooperative Study Activities of Daily Living Inventory; APOE=Apolipoprotein E; CAM=Confusion Assessment Method; CDR-SB=Clinical Dementia Rating Sum of Boxes; CSF=Cerebrospinal Fluid; EOT=End of Treatment; ETV=Early Termination Visit; FSH=Follicle Stimulating Hormone; HLA= human leukocyte antigen; MRI=Magnetic Resonance Imaging; PK/PD=Pharmacokinetic/Pharmacodynamic; Screen=Screening; SD-OCT=Spectral-Domain Optical Coherence Tomography; TC=Telephone Contact; UV=Unscheduled Visit

a The order in which evaluations are completed is at the discretion of the investigator in order to best accommodate the needs of the subject/caregiver as well as the logistical considerations of the coordinator/staff. Screening procedures may be conducted over several visits during the Screening Period provided that the results are available to evaluate inclusion and exclusion criteria before Randomization. Sites will be permitted to initiate screening activities before trial

Safety Cohort Trial Period	Screen Period[a]	Double-Blind Treatment Period																			
		Baseline/ Randomization			Safety Cohort Only													EOT			
Visit Number	1	2	3	4	TC[b]	4b	TC[b]	5	TC[b]	6	TC[b]	7	TC[b]	8	TC[b]	9	TC[b]	10	TC[b,c]	UV	ETV
Scheduled Week	Up to – 4	(Study Day 1[d])	2	4	6	8	10	13	18	26	32	39	45	52	58	65	72	78	80		
Visit Window (Weeks)			±1	±1	±1	±1	±1	±1	±4	±4	±4	±4	±4	±4	±4	±4	±4	±4	+1		

medication is available on-site. The Screening Period may last up to 3 months if needed for logistical (ie, scheduling) reasons or with sponsor's permission for non-logistical reasons. Subjects with screening values/findings outside ranges described in the protocol may, at the discretion of the investigator, be re-screened at a later date. If subjects satisfy the inclusion/exclusion criterion at the re-screening visit, they may continue in the screening process. **NOTE: One re-screen per subject will be allowed without sponsor approval. Additional rescreens may be approved by the Sponsor on a case by case basis.** Re-screening refers to restarting the full screening process beyond the 3-month screening window. Repeat of individual tests during 3-month screening period does not count as a full rescreening. Screening longer than 3 months may be approved by the Sponsor on a case-by-case basis. For approval, the Sponsor should be contacted prior to the subject's randomization visit. Depending on the time that has elapsed since the subject's initial visit, the Sponsor may require repeating some of the Screening assessments prior to randomization.

b Telephone contact with subject and trial partner/caregiver by site to assess safety, AEs, medication compliance, and any other issues. Any telephone contact may be conducted as an in-person unscheduled visit if the subject or caregiver expresses a preference for this or if the site has significant safety/tolerability concerns.

c The telephone contact can have a window from 14 days to 21 days after Visit 10 or ETV.

d The timing of all visits after the Study Day 1 visit (Visit 2) must be based on the day the first dose of trial medication was administered to the subject. **NOTE: If trial medication is not available on-site for the baseline/randomization visit (Visit 2-Study Day 1) then the visit must be postponed until trial medication is available.**

e At designated sites, subjects will be asked to undergo lumbar puncture to provide CSF samples **after** the subject has met all other screening inclusion and exclusion criteria. An informed consent for the CSF substudy must be obtained before the procedure is performed. The CSF substudy consent form includes consent to keep four CSF samples in long term storage for future unspecified biomedical research.

f Informed consent for pharmacogenetic samples must be obtained before the DNA sample is collected. DNA sample for analysis should be obtained predose, on Day 1 (or with the next scheduled blood draw), as the last sample drawn, on randomized subjects only, or at a later date as soon as the informed consent is obtained. Subjects who are unwilling to provide written informed consent for pharmacogenetic testing may be included in the trial.

g To evaluate the eligibility criteria, each must have either: an MRI scan performed during the Screening Period or a previous MRI performed within 12 months before the Screening visit that is reviewed by the central MRI reading vendor. A head CT scan may be accepted instead of MRI on a case-by-case basis, as approved by the Sponsor (e.g., when MRI is contraindicated for the subject). If a new MRI needs to be performed for screening, it is preferable to be scheduled to occur after the subject has met all the inclusion and exclusion criteria that do not require an MRI. The radiologist review by the imaging vendor of the MRI scan must be available before randomization to evaluate the inclusion/exclusion criteria..

h Structural MRI data acquisition for volumetric analyses will be performed at the visits indicated. In addition an MRI may be performed at any visit for safety monitoring if clinically indicated as determined by the investigator (e.g., in follow-up to an AE).To limit variability and operator errors, all MRI sequences should be run at each scan, even if performed for safety monitoring. MRI scans may be acquired at either 1.5 Tesla or 3 Tesla field strength, and for a given subject all scans should be acquired at the same field strength.

Safety Cohort Trial Period	Screen Period[a]	Double-Blind Treatment Period																			
		Baseline/ Randomization			Safety Cohort Only													EOT			
Visit Number	1	2	3	4	TC[b]	4b	TC[b]	5	TC[b]	6	TC[b]	7	TC[b]	8	TC[b]	9	TC[b]	10	TC[b,c]	UV	ETV
Scheduled Week	Up to – 4	(Study Day 1[d])	2	4	6	8	10	13	18	26	32	39	45	52	58	65	72	78	80		
Visit Window (Weeks)			±1	±1	±1	±1	±1	±1	±4	±4	±4	±4	±4	±4	±4	±4	±4	±4	+1		

i Only subjects who have a new MRI scan performed during the screening period will also have an end of treatment MRI scan for purposes of the biomarker analyses. Subjects who were assessed based on an MRI scan performed prior to the study or based on a head CT scan will not undergo an end of treatment MRI scan. For End of Treatment visits, MRI, PET scans, ophthalmic assessments (for applicable subjects), and CSF collection should occur within 4 weeks before the end of treatment clinical visit (V10) and before trial medication is discontinued.

j Procedure should only be done if clinically indicated as determined by the investigator. **NOTE: PK blood samples should be collected at the investigator's discretion when an AE considered possibly related to study medication is associated with the Unscheduled Visit.**

k **NOTE: Triplicate measurements of ECG will be required at the Screening Visit and if there is an observation of QTc prolongation at any post-Screening visit.**

l See **Table 3** for additional lab tests at Screening.

m Each female subject must have a serum pregnancy test (βhCG) at Screening if cessation of menses was < 12 months before Screening and there are no other indications that the subject is not of childbearing potential as defined in the inclusion criterion in Section 7.3.1. The serum pregnancy test must be negative before the first administration of trial medication.

n A blood sample for determining serum FSH should be collected only if serum FSH levels are required to determine childbearing potential as defined in Section 7.3.1.

o Blood samples for APOE and HLA genotyping are required and should be collected in a separate tube from the blood sample taken for the pharmacogenetic substudy. For subjects who were screened prior to Amendment #4 and developed a rash ECI post-randomization, a blood sample for HLA genotyping can be collected retrospectively if they have re-consented for Amendment #4.

p **At Visit 2 only,** blood samples for PK/PD, and pharmacogenetic analyses must be collected before the administration of trial medication and after administration of the ADAS-Cog, CDR, ADCS-ADL, and NPI.

q The ADAS-Cog evaluation at Screening is included so that subjects can become familiar with the assessment, and is not used to evaluate inclusion/exclusion criteria.

r The CDR is performed at the Screening Visit and will be used as the Baseline score unless the Screening Visit is more than 6 weeks before the Baseline Visit, in which case the CDR must be repeated. Repeat CDR can be performed at the Baseline Visit.

s Diagnosis and narrative summary (completed by the PI or qualified designee) will be reviewed by external expert, whose concurrence is required prior to randomization.

t **At Visit 6 only, subjects should not take their trial medication prior to the visit.** Trial medication should be administered on site after the PK/PD blood sample is collected. Safety Cohort subjects who have completed this procedure correctly at the 2-Week Visit (Visit 3) do not need to perform this procedure again at Visit 6.

u The RUD-Lite-Baseline form should be completed at Visit 2. The RUD-Lite-Follow-Up form should be completed at Visits 6, 8, 10 and, if needed, ETV.

v The C-SSRS-Baseline form should be completed at Visit 1. The C-SSRS-Since Last Visit form should be completed at Visits 2, 3, 4, 4b, 5, 6, 7, 8, 9, 10 and, if

Safety Cohort Trial Period	Screen Period[a]	Double-Blind Treatment Period																			
		Baseline/ Randomization			Safety Cohort Only													EOT			
Visit Number	1	2	3	4	TC[b]	4b	TC[b]	5	TC[b]	6	TC[b]	7	TC[b]	8	TC[b]	9	TC[b]	10	TC[b,c]	UV	ETV
Scheduled Week	Up to – 4	(Study Day 1[d])	2	4	6	8	10	13	18	26	32	39	45	52	58	65	72	78	80		
Visit Window (Weeks)			±1	±1	±1	±1	±1	±1	±4	±4	±4	±4	±4	±4	±4	±4	±4	±4	+1		

needed, ETV.

w Dried blood spot (DBS) PK samples will be taken at each timepoint. The site will record the date and time of each PK sample and the date and time of the last two doses of trial medication before each PK sample. PK samples can be taken at the same time when blood samples for hematology and chemistry are taken.

x Best efforts will be made to accommodate the fundus autofluorescence (FAF) assessment. FAF images will be read by a central Reading Center designated by the Sponsor.

y All trial sites will make best efforts to arrange for a dermatologist to perform these scheduled assessments. Where this is operationally infeasible (eg, excessive travel distance to a dermatologist), a site physician may perform the skin assessments at these visits with sponsor approval. The site should document all reasonable efforts made to identify a dermatologist. In the case that a site's dermatologist has not yet completed all site ready activities (e.g., regulatory documentation, training), the site physician should perform the Full Body Skin Examinations at Visits 1 and 6 after notifying the Sponsor. For convenience, the Visit 1 skin examination may be performed after the MRI scan but prior to the baseline Visit 2.

z Only subjects with a baseline ophthalmology exam will have a Week 78 ophthalmology exam completed as described in Section 7.1. Subjects are NOT required to discontinue from the trial if for some reason the ophthalmic procedures cannot be obtained at Week 78.

2.2.2 Trial Flow Chart for Main Cohort (Part I)

The cells highlighted in blue refer to differences between the Safety Cohort and the Main Cohort.

NOTE: Blood and CSF samples should NOT be collected immediately prior to testing of clinical outcomes.

Main Cohort Trial Period	Screening Period[a]	Double-Blind Treatment Period																	
		Baseline/ Randomization														EOT			
Visit Number	1	2	3	4	TC[b]	5	TC[b]	6	TC[b]	7	TC[b]	8	TC[b]	9	TC[b]	10	TC[b,c]	UV	ETV
Scheduled Week	Up to – 4	(Study Day 1[d])	2	4	8	13	18	26	32	39	45	52	58	65	72	78	80		
Visit Window (Weeks)			±1	±1	±1	±1	±4	±4	±4	±4	±4	±4	±4	±4	±4	±4	+1		
Informed Consent	X																		
Informed Consents for Substudies[e]	X																		
Pharmacogenetic Testing Informed Consent[f]	X																		
Issue/Collect Subject Identification Card	X															X			X
Medical History	X																		
Review Prior Medications	X																		
Record Concomitant Medications	X	X	X	X	X	X	X	X	X	X	X	X	X	X	X	X	X	X	X
Structural MRI	X[g,h,i]															X[h,i]		X[j]	X[h,i]
Vital Signs	X	X	X	X		X		X		X		X		X		X		X	X
Body Weight	X					X		X				X				X			X
Height	X																		
Physical & Neurological Examinations	X									X						X		X[j]	X
12-Lead Electrocardiogram[k]	X		X									X						X[j]	X

Main Cohort Trial Period	Screening Period[a]	Double-Blind Treatment Period																	
		Baseline/ Randomization														EOT			
Visit Number	1	2	3	4	TC[b]	5	TC[b]	6	TC[b]	7	TC[b]	8	TC[b]	9	TC[b]	10	TC[b,c]	UV	ETV
Scheduled Week	Up to – 4	(Study Day 1[d])	2	4	8	13	18	26	32	39	45	52	58	65	72	78	80		
Visit Window (Weeks)			±1	±1	±1	±1	±4	±4	±4	±4	±4	±4	±4	±4	±4	±4	+1		
Full Body Skin Examination by Dermatologist[y]	X							X											
Directed Skin Examination by Site Physician						X						X				X			X
Hematology and Chemistry Blood Samples	X[l]		X			X		X		X		X				X		X[j]	X
Serum Pregnancy Test (βhCG) Blood Samples	X[m]																		
FSH Blood Samples	X[n]																		
APOE and HLA Genotyping Blood Samples	X[o]																		
Pharmacogenetic Blood Sample		X[f,o,p]																	
PK/PD Blood Samples[w]		X[p]	X			X		X[t]				X		X		X		X[j]	X
Urinalysis	X		X			X		X		X		X				X		X[j]	X
Modified Hachinski Ischemia Score	X																		
15-item Geriatric Depression Scale	X																		
Inclusion/Exclusion Criteria	X	X																	
ADAS-Cog	X[q]	X				X		X		X		X		X		X			X
ADCS-ADL		X				X		X		X		X		X		X			X
CDR-SB	X[r]	X[r]						X				X				X			X
Mini-Mental State Examination (MMSE)	X							X				X				X			X

Main Cohort Trial Period	Screening Period[a]	Double-Blind Treatment Period																	
		Baseline/ Randomization														EOT			
Visit Number	1	2	3	4	TC[b]	5	TC[b]	6	TC[b]	7	TC[b]	8	TC[b]	9	TC[b]	10	TC[b,c]	UV	ETV
Scheduled Week	Up to – 4	(Study Day 1[d])	2	4	8	13	18	26	32	39	45	52	58	65	72	78	80		
Visit Window (Weeks)			±1	±1	±1	±1	±4	±4	±4	±4	±4	±4	±4	±4	±4	±4	+1		
Diagnostic Assessment and Narrative Summary[s]	X																		
Neuropsychiatric Inventory (NPI)		X				X		X				X				X			X
Modified Resource Utilization in Dementia (RUD) Lite[u]		X						X				X				X			X
Health Economic Assessment (HEA)		X						X				X				X			X
EuroQol Five Dimension Questionnaire (EQ-5D)		X						X				X				X			X
Columbia Suicide Severity Rating Scale (C-SSRS)[v]	X	X	X	X		X		X		X		X		X		X		X[j]	X
CSF Collection[e]	X[g]															X[i]			X[i]
Amyloid Imaging by PET[e]	X[g]															X[i]			X[i]
Record Adverse Events	X	X	X	X	X	X	X	X	X	X	X	X	X	X	X	X	X	X	X
Dispense Trial Medication		X		X		X		X		X		X		X					
Administration of Trial Medication on Site		X						X[t]											
Medication Compliance			X	X	X	X	X	X	X	X	X	X	X	X	X	X		X	X
Drug Accountability Assessment			X	X		X		X		X		X		X		X		X	X
Ophthalmology Visit (details below)																X[z, i]			
Visual Acuity																X[z, i]			
Posterior Eye Exams																X[z, i]			

Main Cohort Trial Period	Screening Period[a]	Double-Blind Treatment Period																	
		Baseline/ Randomization														EOT			
Visit Number	1	2	3	4	TC[b]	5	TC[b]	6	TC[b]	7	TC[b]	8	TC[b]	9	TC[b]	10	TC[b,c]	UV	ETV
Scheduled Week	Up to – 4	(Study Day 1[d])	2	4	8	13	18	26	32	39	45	52	58	65	72	78	80		
Visit Window (Weeks)			±1	±1	±1	±1	±4	±4	±4	±4	±4	±4	±4	±4	±4	±4	+1		
Fundus Photography																X[z, i]			
Fundus Autofluorescence [x]																X[z, i]			
SD-OCT																X[z, i]			
CAM		X -------- To be completed only for adverse events of delirium (see 7.7.2.2.3).------------X																	

βhCG=β-Human Chorionic Gonadotropin; ADAS-Cog=Alzheimer's Disease Assessment Scale Cognitive Subscale; ADCS-ADL=Alzheimer's Disease Cooperative Study Activities of Daily Living Inventory; APOE=Apolipoprotein E; CAM=Confusion Assessment Method; CDR-SB=Clinical Dementia Rating Sum of Boxes; CSF=Cerebrospinal Fluid; EOT=End of Treatment; ETV=Early Termination Visit; FSH=Follicle Stimulating Hormone; HLA= human leukocyte antigen; MRI=Magnetic Resonance Imaging; PK/PD=Pharmacokinetic/Pharmacodynamic; PET=Positron Emission Tomography; Screen=Screening; SD-OCT=Spectral-Domain Optical Coherence Tomography; TC=Telephone Contact; UV=Unscheduled Visit

a The order in which evaluations are completed is at the discretion of the investigator in order to best accommodate the needs of the subject/caregiver as well as the logistical considerations of the coordinator/staff. Screening procedures may be conducted over several visits during the Screening Period provided that the results are available to evaluate inclusion and exclusion criteria before Randomization. Sites will be permitted to initiate screening activities before trial medication is available on-site. The Screening Period may last up to 3 months if needed for logistical (ie, scheduling) reasons or with sponsor's permission for non-logistical reasons. Subjects with screening values/findings outside ranges described in the protocol may, at the discretion of the investigator, be re-screened at a later date. If subjects satisfy the inclusion/exclusion criterion at the re-screening visit, they may continue in the screening process. **NOTE: One re-screen per subject will be allowed without Sposor approval. Additional rescreens may be approved by the Sponsor on a case by case basis.**. Re-screening refers to restarting the full screening process beyond the 3-month screening window. Repeat of individual tests during 3-month screening period does not count as a full rescreening. Screening longer than 3 months may be approved by the Sponsor on a case-by-case basis. For approval, the Sponsor should be contacted prior to the subject's randomization visit. Depending on the time that has elapsed since the subject's initial visit, the Sponsor may require repeating some of the Screening assessments prior to randomization.

b Telephone contact by site with subject and trial partner/caregiver to assess safety, AEs, medication compliance, and any other issues. Any telephone contact may be conducted as an in-person unscheduled visit if the subject or caregiver expresses a preference for this or if the site has significant safety/tolerability concerns.

c The telephone contact can have a window from 14 days to 21 days after Visit 10 or ETV.

d The timing of all visits after the Study Day 1 visit (Visit 2) must be based on the day the first dose of trial medication was administered to the subject. **NOTE: If trial medication is not available on-site for the baseline/randomization visit (Visit 2-Study Day 1) then the <u>visit must be postponed</u> until trial medication is available.**

e At designated sites, subjects will be asked to undergo amyloid imaging using PET, and/or lumbar puncture to provide CSF samples after the subject has met all

Main Cohort Trial Period	Screening Period[a]	Double-Blind Treatment Period																		
		Baseline/ Randomization															EOT			
Visit Number	1	2	3	4	TC[b]	5	TC[b]	6	TC[b]	7	TC[b]	8	TC[b]	9	TC[b]	10	TC[b,c]	UV	ETV
Scheduled Week	Up to – 4	(Study Day 1[d])	2	4	8	13	18	26	32	39	45	52	58	65	72	78	80		
Visit Window (Weeks)			±1	±1	±1	±1	±4	±4	±4	±4	±4	±4	±4	±4	±4	±4	+1		

other screening inclusion and exclusion criteria. Informed consent for the substudies must be obtained before the procedures are performed. The CSF substudy consent form includes consent to keep four CSF samples in long term storage for future unspecified biomedical research.

f Informed consent for pharmacogenetic samples must be obtained before the DNA sample is collected. DNA sample for analysis should be obtained predose, on Day 1 (or with the next scheduled blood draw), as the last sample drawn, on randomized subjects only, or at a later date as soon as the informed consent is obtained. Subjects who are unwilling to provide written informed consent for pharmacogenetic testing may be included in the trial.

g To evaluate the eligibility criteria, each must have either: a MRI scan performed during the Screening Period or a previous MRI performed within 12 months before the Screening visit that is reviewed by the central MRI reading vendor. A head CT scan may be accepted instead of MRI on a case-by-case basis, as approved by the Sponsor (e.g., when MRI is contraindicated for the subject). If a new MRI needs to be performed for screening, it is preferable to be scheduled to occur after the subject has met all the inclusion and exclusion criteria that do not require an MRI. The radiologist review by the imaging vendor of the MRI scan must be available before randomization to evaluate the inclusion/exclusion criteria.

h Structural MRI data acquisition for volumetric analyses will be performed at the visits indicated. In addition an MRI may be performed at any visit for safety monitoring if clinically indicate as determined by the investigator (e.g., in follow-up to an AE).To limit variability and operator errors, all MRI sequences should be run at each scan, even if performed for safety monitoring. MRI scans may be acquired at either 1.5 Tesla or 3 Tesla field strength, and for a given subject all scans should be acquired at the same field strength.

i Only subjects who have a new MRI scan performed during the screening period will also have an end of treatment MRI scan for purposes of the biomarker analyses. Subjects who were assessed based on an MRI scan performed prior to the study or based on a head CT scan will not undergo an end of treatment MRI scan. For End of Treatment visits, MRI, PET scans, ophthalmic assessments (for applicable subjects) and CSF collection should occur within 4 weeks before the end of treatment clinical visit (V10) and before trial medication is discontinued.

j Procedure should only be done if clinically indicated as determined by the investigator. **NOTE: PK blood samples should be collected at the investigator's discretion when an AE considered possibly related to study medication is associated with the Unscheduled Visit.**

k **NOTE: Triplicate measurements of ECG will be required at the Screening Visit and if there is an observation of QTc prolongation at any post-Screening visit.**

l See Table 3 for additional lab tests at Screening.

m Each female subject must have a serum pregnancy test (βhCG) at Screening if cessation of menses was < 12 months before Screening and there are no other indications that the subject is not of childbearing potential as defined in the inclusion criterion in Section 7.3.1. The serum pregnancy test must be negative before the first administration of trial medication.

n A blood sample for determining serum FSH should be collected only if serum FSH levels are required to determine childbearing potential as defined in Section 7.3.1.

o Blood samples for APOE and HLA genotyping are required and should be collected in a separate tube from the blood sample taken for the pharmacogenetic substudy. For subjects who were screened prior to Amendment #4 and developed a rash ECI post-randomization, a blood sample for HLA genotyping can be

**Main Cohort Trial Period**	**Screening Period[a]**	**Double-Blind Treatment Period**																	
		**Baseline/ Randomization**														**EOT**			
**Visit Number**	**1**	**2**	**3**	**4**	**TC[b]**	**5**	**TC[b]**	**6**	**TC[b]**	**7**	**TC[b]**	**8**	**TC[b]**	**9**	**TC[b]**	**10**	**TC[b,c]**	**UV**	**ETV**
**Scheduled Week**	**Up to – 4**	**(Study Day 1[d])**	**2**	**4**	**8**	**13**	**18**	**26**	**32**	**39**	**45**	**52**	**58**	**65**	**72**	**78**	**80**		
**Visit Window (Weeks)**			**±1**	**±1**	**±1**	**±1**	**±4**	**±4**	**±4**	**±4**	**±4**	**±4**	**±4**	**±4**	**±4**	**±4**	**+1**		

collected retrospectively if they have re-consented for Amendment #4.

p **At Visit 2 only,** blood samples for PK/PD, and pharmacogenetic analyses must be collected before the administration of trial medication and after administration of the ADAS-Cog, CDR, ADCS-ADL, and NPI.

q The ADAS-Cog evaluation at Screening is included so that subjects can become familiar with the assessment, and is not used to evaluate inclusion/exclusion criteria.

r The CDR is performed at the Screening Visit and will be used as the Baseline score unless the Screening Visit is more than 6 weeks before the Baseline Visit, in which case the CDR must be repeated. Repeat CDR can be performed at the Baseline Visit.

s Diagnosis and narrative summary (completed by the PI or qualified designee) will be reviewed by external expert, whose concurrence is required prior to randomization.

t **At Visit 6 only, subjects should not take their trial medication prior to the visit.** Trial medication should be administered on site after the PK/PD blood sample is collected.

u The RUD-Lite-Baseline form should be completed at Visit 2. The RUD-Lite-Follow-Up form should be completed at Visits 6, 8, 10 and, if needed, ETV.

v The C-SSRS-Baseline form should be completed at Visit 1. The C-SSRS-Since Last Visit form should be completed at Visits 2, 3, 4, 5, 6, 7, 8, 9, 10 and, if needed, ETV.

w Both plasma and dried blood spot (DBS) PK samples will be taken at each timepoint. The site will record the date and time of each PK sample and the date and time of the last two doses of trial medication before each PK sample. PK samples can be taken at the same time when blood samples for hematology and chemistry are taken.

x Best efforts will be made to accommodate the FAF assessment. FAF images will be read by a central Reading Center designated by the Sponsor.

y All trial sites will make best efforts to arrange for a dermatologist to perform these scheduled assessments. Where this is operationally infeasible (eg, excessive travel distance to a dermatologist), a site physician may perform the skin assessments at these visits with sponsor approval. The site should document all reasonable efforts made to identify a dermatologist. In the case that a site's dermatologist has not yet completed all site ready activities (e.g., regulatory documentation, training), the site physician should perform the Full Body Skin Examinations at Visits 1 and 6 after notifying the Sponsor. For convenience, the Visit 1 skin examination may be performed after the MRI scan but prior to the baseline Visit 2.

z Only subjects with a baseline ophthalmology exam will have a Week 78 ophthalmology exam completed as described in Section 7.1. Subjects are NOT required to discontinue from the trial if for some reason the ophthalmic procedures cannot be obtained at Week 78.

2.2.3 Trial Flow Chart for Trial Extension (Part II)

Refer to Section 11.1.2 for the Trial Flow Chart for Trial Extension Part II.

3.0 TABLE OF CONTENTS

1.0 TITLE PAGE 1
SUMMARY OF CHANGES 2
2.0 SYNOPSIS 6
2.1 Trial Design Diagram 13
2.1.1 Trial Diagram for Safety Cohort (Part I) 13
2.1.2 Trial Diagram for Main Cohort (Part I) 14
2.1.3 Trial Diagram for Trial Extension (Part II) 15
2.2 Trial Flow Charts 15
2.2.1 Trial Flow Chart for Safety Cohort (Part I) 15
2.2.2 Trial Flow Chart for Main Cohort (Part I) 22
2.2.3 Trial Flow Chart for Trial Extension (Part II) 28
3.0 TABLE OF CONTENTS 29
3.1 List of Tables 35
3.2 List of Appendices 36
4.0 LIST OF ABBREVIATIONS AND DEFINITIONS OF TERMS 37
5.0 INTRODUCTION 40
5.1 Therapeutic Rationale 40
5.2 Subject Population Rationale 41
5.3 Trial Design Rationale 42
5.4 Outcome Measures Rationale 44
5.5 Dose and Administration Rationale 46
6.0 TRIAL OBJECTIVES AND HYPOTHESES (PART I) 47
6.1 Primary Trial Objectives and Hypotheses 47
6.2 Key Secondary Trial Objective and Hypothesis 47
6.3 Other Secondary Trial Objectives and Hypotheses 47
6.4 Exploratory Objectives 48
7.0 INVESTIGATIONAL AND ANALYSIS PLAN 49
7.1 Overall Trial Design 49
7.2 Beginning and End of the Trial (Part I) 56
7.3 Trial Population 57
7.3.1 Subject Inclusion Criteria 58
7.3.2 Subject Exclusion Criteria 60
7.3.3 Subject Discontinuation Criteria 65
7.3.4 Replacement of Subjects 67
7.4 Treatments 67
7.4.1 Trial Treatments 67
7.4.1.1 Treatments Administered 67

7.4.1.2 Method of Treatment Assignment, Randomization, and/or Stratification 68
7.4.1.3 Selection and Timing of Dose for Each Subject 69
7.4.1.3.1 Selecting the Dose for Each Subject 69
7.4.1.3.2 Determining the Timing of Dose Administration for Each Subject 69
7.4.1.4 Blinding Trial Treatments 70
7.4.1.5 Investigational Medicinal Products 70
7.4.1.5.1 Identity of Investigational Medicinal Products 70
7.4.1.5.2 Source 70
7.4.1.5.3 Labeling 70
7.4.1.5.4 Packaging 70
7.4.1.5.5 Storage 71
7.4.1.5.6 Dispensing 71
7.4.1.5.7 Replacement of Investigational Product 71
7.4.1.5.8 Investigational Medicinal Product Accountability 71
7.4.2 Non-Trial Treatments 72
7.4.2.1 Prior and Concomitant Medications 72
7.4.2.1.1 Medications, Supplements, and Other Substances Prohibited Prior to Screening and During the Trial 72
7.4.2.1.2 Concomitant Medications, Supplements, and Other Substances Allowed During the Trial 73
7.4.3 Procedures for Monitoring Subject Compliance With Administration of Trial Treatments 74
7.5 Trial Schedule 74
7.6 Trial Procedures 75
7.7 Assessments 87
7.7.1 Efficacy Assessments 87
7.7.1.1 Coprimary Efficacy Endpoints 87
7.7.1.2 Key Secondary Efficacy Endpoint 87
7.7.1.3 Other Efficacy Endpoints 87
7.7.1.3.1 Other Secondary Efficacy Endpoints 87
7.7.1.3.2 Exploratory Efficacy Endpoints 88
7.7.2 Safety Monitoring and Assessments 88
7.7.2.1 Safety Endpoints 88
7.7.2.2 Definition of Terms 88
7.7.2.2.1 Adverse Event 88
7.7.2.2.2 Serious Adverse Event 89
7.7.2.2.3 Events of Clinical Interest 90

7.7.2.2.4 Overdose 92
7.7.2.2.5 Clinical Supply Complaint 92
7.7.2.2.6 Planned Hospitalization.. 93
7.7.2.3 Monitoring 93
7.7.2.3.1 Monitoring Adverse Events 93
7.7.2.3.2 Monitoring Laboratory Assessments............................. 94
7.7.2.4 Assessment of Adverse Events 94
7.7.2.4.1 Reference Safety Information (RSI) for the Assessment of Expectedness of Adverse Events 97
7.7.2.4.2 Potential Toxicities of Investigational Products 97
7.7.2.5 Reporting Safety Observations by the Investigator to the Sponsor.. 97
7.7.2.5.1 Expedited Reporting.. 97
7.7.2.5.2 Expedited Reporting by the Sponsor to a Regulatory Health Authority 98
7.7.2.5.3 Unblinding Treatment for a Subject During the Trial 98
7.7.3 Pharmacogenetics 99
7.7.3.1 Pharmacogenetics Endpoints and Analyses 99
7.7.4 Other Endpoints 99
7.7.4.1 Pharmacodynamic Endpoints 99
7.7.4.2 Pharmacoeconomic and Quality of Life Endpoints................ 99
7.8 Criteria for Early Termination of the Trial 100
8.0 STATISTICAL AND ANALYTICAL PLAN 100
8.1 Subject Populations to be Analyzed 101
8.2 Efficacy Analyses 102
8.2.1 Primary Efficacy Analysis... 104
8.2.2 Key Secondary Efficacy Analysis... 107
8.2.3 Multiplicity 107
8.2.4 Other Secondary Efficacy Analyses... 109
8.2.5 Exploratory Analyses 110
8.2.6 Dropping Dose Arms... 111
8.2.7 Subgroup Analyses.. 112
8.2.8 Justification of Sample Size 113
8.2.8.1 Parameter Estimates for Coprimary Endpoints and Key Secondary Endpoints... 113
8.2.8.2 Parameter Estimates for Secondary Biomarkers / Substudy Endpoints.. 115
8.2.8.3 Assumptions for the Effect of MK-8931............................... 116
8.2.8.4 Determining Sample Size / Power 117

8.2.8.4.1 Primary and Key Secondary Endpoints 117
8.2.8.4.2 Secondary Biomarkers and Substudy Endpoints 118
8.2.9 Interim Analysis 119
8.2.10 Accounting for Missing Data 124
8.3 Safety 127
8.3.1 Analysis of Prespecified Safety Endpoints 130
8.3.2 Analysis of Commonly Occurring Safety Endpoints 130
8.3.3 Analysis of Descriptive Safety Endpoints 130
8.4 Trial Medication Compliance and Exposure 131
8.4.1 Compliance 131
8.4.2 Exposure 131
8.5 Demography 131
9.0 ADHERENCE TO ETHICAL, REGULATORY, AND ADMINISTRATIVE CONSIDERATIONS 132
9.1 Ethical Conduct of the Trial 132
9.1.1 Independent Ethics Committee or Institutional Review Board 132
9.1.2 Subject Information and Consent 132
9.1.3 Subject Identification Card 133
9.1.4 Registration of the Trial 134
9.2 Reporting Trial Data to the Sponsor 134
9.2.1 Data Collection Forms 134
9.2.2 Preparing Case Report Forms for All Subjects 134
9.2.3 Preparing Case Report Forms for Subjects Who Fail Screening 135
9.3 Publications and Other Rights 135
9.3.1 Rights to Publish by the Investigator 135
9.3.2 Use of Proprietary or Confidential Information in a Publication 136
9.3.3 Use of Trial Information in a Publication 136
9.3.4 Authorship of Publications 137
9.4 Trial Documents and Records Retention 137
10.0 INVESTIGATORS AND TRIAL ADMINISTRATIVE STRUCTURE 138
10.1 Sponsor 138
10.2 Investigators 138
10.2.1 Selecting Investigators 138
10.2.2 Financial Disclosure Requirement 138
10.2.3 Clinical Study Report Coordinator Investigator 139
10.3 Central Organizations 139
10.3.1 Scientific Advisory Committee 139
10.3.2 Executive Oversight Committee 139

10.3.3 Data Monitoring Committee 139

11.0 TRIAL EXTENSION (PART II) 140

11.1 EXTENSION DESIGN 140

11.1.1 Extension Diagram........ 142

11.1.2 Extension Trial Flow Chart 143

11.2 EXTENSION RATIONALE 145

11.2.1 Rationale for Trial Extension 145

11.2.2 Dose and Administration Rationale for Extension 145

11.3 EXTENSION OBJECTIVE (S) 146

11.3.1 Extension Primary Objectives 146

11.3.2 Extension Exploratory Objective 146

11.4 BEGINNING AND END OF TRIAL EXTENSION (PART II) 146

11.5 EXTENSION POPULATION........ 147

11.5.1 Extension Inclusion Criteria 147

11.5.2 Extension Exclusion Criteria 148

11.5.3 Extension Discontinuation Criteria 149

11.5.4 Replacement of Extension Subjects 150

11.6 Treatment in the Extension........ 151

11.6.1 Treatment in the Extension 151

11.6.2 Investigational Medicinal Product........ 151

11.6.2.1 Source 151

11.6.2.2 Packaging 152

11.6.2.3 Labelling........ 152

11.6.2.3.1.1 Calendar Sleeves for Study Medication Bottles . 152

11.6.3 Prior and Concomitant Medications 152

11.6.3.1 Medications, Supplements and Other Substances Prohibited Prior to Screening and During the Extension 152

11.6.3.2 Concomitant Medications, Supplements, and Other Substances Allowed During the Extension 153

11.6.4 Extension Schedule 154

11.6.5 Extension Procedures........ 155

11.6.6 Extension Assessments........ 159

11.6.6.1 Efficacy Assessments 159

11.6.6.1.1 Extension Primary Efficacy Endpoints........ 159

11.6.6.1.2 Extension Exploratory Efficacy Endpoints........ 159

11.6.7 Safety Assessments 160

11.6.7.1 Extension Safety Endpoints 160

11.7 STATISTICAL AND ANALYTIC PLAN FOR EXTENSION POPULATION 160

11.7.1 Subjects to be Analyzed 160
11.7.2 Efficacy Analyses 161
11.7.2.1 Primary Efficacy Analysis 162
11.7.2.2 Exploratory Analysis 162
11.7.2.3 Multiplicity 162
11.7.2.4 Subgroup Analyses 162
11.7.2.5 Parameter Estimates for the Primary Endpoints 162
11.7.2.5.1 Assumptions for the Effect of MK-8931 162
11.7.2.5.2 Power Calculations 163
11.7.3 Dropping Dose Arms 164
11.7.4 Accounting for Missing Data 164
11.7.5 Safety Analyses 164
11.7.6 Trial Medication Compliance and Exposure 164
11.7.6.1 Compliance 164
11.7.6.2 Exposure 165
11.7.7 Demography 165
12.0 REFERENCES 165
INVESTIGATOR SIGNATURE PAGE 228

Confidential

3.1 List of Tables

Table 1 Prohibited Medications, Supplements, and Other Substances 63
Table 2 Medications, Supplements, and Other Substances Allowed During the Trial 74
Table 3 Laboratory Tests 81
Table 4 Evaluating Adverse Events 95
Table 5 Analysis Strategy for Efficacy Variables 103
Table 6 Parameter Assumptions for Progression Rate on Placebo, Standard Deviations, and Within-Endpoint Correlations for ADAS-Cog, ADCS-ADL, and CDR-SB 115
Table 7 Parameter Assumptions for Progression Rate on Placebo, Standard Deviations, and Within-Endpoint Correlations for THV, Total Tau, and Cortical Amyloid Load 116
Table 8 Assumed Change-From-Baseline Treatment Effects (Active – Placebo) at 78 Weeks of Treatment for Various Potential Efficacy Configurations of the High and Low Doses of MK-8931 117
Table 9 Number of Randomized Subjects Required for 90% Overall Power Under Different Assumed Drug Effects[e] 118
Table 10 Summary of Interim Analysis Strategy 120
Table 11 Analysis Strategy for Safety Parameters 130
Table 12 Prohibited Medications, Supplements, and Other Substances 149
Table 13: Medications, Supplements, and Other Substances Allowed During the Trial 154
Table 14 Laboratory Tests 158
Table 15 Analysis Strategy for Primary Endpoints 161
Table 16 Parameter Assumptions for Progression Rate on Placebo, Standard Deviations, and Within Endpoint Correlations for ADAS-Cog, ADCS-ADL, and CDR-SB 163
Table 17 Power Calculations (MK-8931 40 mg / MK-8931 40 mg vs. Placebo / MK-8931 40 mg) 163

3.2 List of Appendices

Appendix 1 Code of Conduct for Clinical Trials 168
Appendix 2 DNA Sampling and Pharmacogenetic Analysis Procedures 172
Appendix 3 The National Institute of Neurological and Communicative Diseases and Stroke/Alzheimer’s Disease and Related Disorders Association (NINCDS-ADRDA) Criteria for Probable AD[18] 178
Appendix 4 Comparison of the Constrained Longitudinal Data Analysis Model and the Longitudinal ANCOVA Model and Guidance on Longitudinal Data Analysis: *Efficient Use of Baseline Information in Longitudinal Data Analyses* 181
Appendix 5 Predefined Limits of Change Criteria 219
Appendix 6 The use of [18F]Flutemetamol, a Positron Emission Tomography (PET) tracer 222

Confidential

4.0 LIST OF ABBREVIATIONS AND DEFINITIONS OF TERMS

Term	Definition
Aβ	amyloid β
AChE	Acetylcholinesterase
AChEI	Acetylcholinesterase Inhibitor
AD	Alzheimer’s Disease
ADAS-Cog	Alzheimer’s Disease Assessment Scale Cognitive Subscale
ADCS-ADL	Alzheimer’s Disease Cooperative Study Activities of Daily Living Inventory
ADL	Activities of Daily Living
ADNI	Alzheimer's Disease Neuroimaging Initiative
AE	Adverse Event
ALT	Alanine aminotransferase (SGPT)
ANCOVA	Analysis of Covariance
APaT	All-Patients-as-Treated
APOE	Apolipoprotein E
APP	Amyloid Precursor Protein
AREDS	Age-Related Eye Disease Study
AST	Aspartate aminotransferase (SGOT)
BACE	β-site APP cleaving enzyme
BP	Blood Pressure
βhCG	β-Human Chorionic Gonadotropin
BSA	Body Surface Area
BUN	Blood Urea Nitrogen
CAM	Confusion Assessment Method
CBC	Complete Blood Count
CDR-SB	Clinical Dementia Rating Sum of Boxes
CDT	Counterfeit, Diversion and Tampering
CFR	Code of Federal Regulations
CI	Confidence Interval
cLDA	Constrained Longitudinal Data Analysis
CMH	Cochran-Mantel-Haenzel
CMV	Cytomegaloviruse
CRF	Case Report Form
CRO	Clinical Research Organization
CSF	Cerebrospinal Fluid
CSR	Clinical Study Report
CTD	Clinical Trial Directive
CYP	Cytochrome P450
DBS	Dried Blood Spot
DNA	Deoxyribonucleic Acid
DMC	Data Monitoring Committee
DRESS	Drug Reaction with Eosinophilia and Systemic Symptoms
DSM-IV-TR	Diagnostic and Statistical Manual of Mental Disorders, 4th Edition, Text Revision
EBV	Epstein-Barr Virus
ECG	Electrocardiogram

Term	Definition
ECI	Events of Clinical Interest
eCRF	Electronic Case Report Form
EDC	Electronic Data Capture
eDMC	External (to Sponsor) Data Monitoring Committee
EDTA	Ethylenediamine Tetraacetic Acid
EIA	Enzyme immunoassay
EM	Exposure Multiples
EMA	European Medicines Agency
EQ-5D	EuroQol Five Dimension Questionnaire
EOC	Executive Oversight Committee
ETDRS	Early Treatment Diabetic Retinopathy Study
EU	European Union
FAF	Fundus Autofluorescence
FAS	Full Analysis Set
FDA	Food and Drug Administration, USA
FSH	Follicle Stimulating Hormone
FTA-ABS	Fluorescent treponemal antibody absorption
GCP	Good Clinical Practice
GMP	Good Manufacturing Practice
HEA	Health Economic Assessment
HLA	Human Leukocyte Antigen
ICF	Informed Consent Form
ICH	International Conference on Harmonisation of Technical Requirements for Registration of Pharmaceuticals for Human Use
ICMJE	International Committee of Medical Journal Editors
ID	Identification
IEC	Independent Ethics Committee
IMP	Investigational Medicinal Product
IND	Investigational New Drug Application; legal instrument in the USA that allows trial of unapproved, investigational new drugs in human subjects
Investigational Product	The drug, biologic, and/or device being investigated in the current trial
IOP	Intraocular pressure
IPR	Indirect Pharmacologic Response
IRB	Institutional Review Board
IU	International Units
IVRS	Interactive Voice Response System
K_i	Equilibrium Inhibition Constant
K-M	Kaplan-Meier
LDH	Lactate Dehydrogenase
LFT	Liver Function Test
LSM	Least Squares Mean
MAR	Missing at Random
MCAR	Missing Completely at Random
MFAS	Modified Full Analysis Set
MMSE	Mini-Mental State Examination
MNAR	Missing Not at Random

Term	Definition
MRI	Magnetic Resonance Imaging
mSv	millisievert
NINCDS-ADRDA	National Institute of Neurological and Communicative Diseases and Stroke/Alzheimer's Disease and Related Disorders Association
NMDA	N-Methyl-D-Aspartate
NPI	Neuropsychiatric Inventory
OCT	Optical Coherence Tomography
PD	Pharmacodynamic
PDLC	Pre-Defined Limit of Change
PET	Positron Emission Tomography
PiB	Pittsburgh compound B
P-gp	P-glycoprotein
PK	Pharmacokinetic
PK/PD	Pharmacokinetic/Pharmacodynamic
p-tau	Phosphorylated Microtubule-Associated Protein Tau
RBC	Red Blood Cell
RNA	Ribonucleic Acid
RPE	Retinal Pigment Epithelium
RSD	Rising Single Dose
RSI	Reference Safety Information
RUD Lite	Resource Utilization in Dementia Lite Questionnaire
SAE	Serious Adverse Event
(S)AE	All adverse events, including serious adverse events
SAP	Statistical Analysis Plan
sAPPβ	N-terminal fragment secreted after β-secretase cleavage of APP
SD-OCT	Spectral-Domain Optical Coherence Tomography
SGOT	Serum Glutamic Oxaloacetic Transaminase (AST)
SGPT	Serum Glutamic Pyruvic Transaminase (ALT)
siDMC	Standing Internal (to Sponsor) Data Monitoring Committee
SOC	System Organ Class
T-BIL	Total Bilirubin
TC	Telephone Contact
Term	Termination
THV	Total hippocampal volume
TSH	Thyroid Stimulating Hormone
Tx	Treatment
UGT1A1	Uridine diphosphate glucuronosyltransferase 1A1
ULN	Upper Limit of Normal
Unsched	Unscheduled
USA	United States of America
WBC	White Blood Cell

5.0 INTRODUCTION

This study is comprised of two parts. Part I refers to the initial 78-week treatment period. Part II refers to the extension period (up to approximately 260 weeks). Details for Part I are provided in Sections 5 to 8 of this protocol and details for Part II are provided in Section 11.0.

NOTE subject participation in Part II is optional and Part II will only be conducted in areas where approved by local authorities._

5.1 Therapeutic Rationale

Alzheimer's disease (AD) is a slowly developing neurodegenerative disease that is the leading cause of dementia world-wide. Currently available treatments for AD are limited, and include cholinesterase inhibitors (eg, donepezil) and the low affinity NMDA receptor antagonist (memantine) which modestly improve symptoms but do not alter disease progression. Therefore, novel pharmacological agents that slow or halt the progression of AD are needed.

Alzheimer's disease is characterized by specific histopathological features including amyloid deposits (plaques), neurofibrillary tangles, and neuronal degeneration. The "amyloid hypothesis" posits that amyloid-β (Aβ) peptides aggregate into complexes, such as fibrils and plaques, which subsequently trigger the development of tau-related neurofibrillary tangles. These tangles are thought to be the more proximal cause of neuronal degeneration. Aβ pathology appears to begin years before the onset of AD and is thought at some point to trigger tau pathology, neural degeneration, and the subsequent gradual emergence of clinical symptoms. As amyloid plaques continue to accumulate, tangle pathology spreads to a variety of brain regions, leading to progressive neuronal degeneration, brain atrophy, and cognitive decline.

Aβ peptides are produced when amyloid precursor protein (APP) is cleaved by three distinct proteases: α-secretase, BACE1 (β site APP cleaving enzyme 1; also known as β-secretase), and γ-secretase. Most APP is processed by α and γ-secretases to generate nonamyloidogenic peptides. However, 5-10% of APP is cleaved by BACE1 and γ-secretase to generate pathogenic Aβ peptides (Aβ40 and Aβ42). Deletion of BACE1 in mice eliminates Aβ in both the plasma and the brain. Thus, inhibition of BACE1 is a potential therapeutic strategy for slowing or halting progression of AD.

Confidential

MK-8931 is a potent BACE1 inhibitor being developed for the treatment of AD. It has been shown to reduce Aβ levels in the cerebrospinal fluid (CSF) and brain of rodents and primates. MK-8931 also reduces Aβ in human CSF. In Phase 1 trials, MK-8931 has been generally safe and well tolerated (see Investigator's Brochure). These results suggest that MK-8931 may reduce Aβ production in humans and could potentially slow progression in subjects with mild to moderate AD. Detailed information about MK-8931 including preclinical studies, pharmacokinetics and other relevant information is provided in the Investigator's Brochure.

5.2 Subject Population Rationale

Alzheimer's disease is clinically staged as mild, moderate, or severe. Subjects with mild to moderate AD have less amyloid deposition and neuronal loss than subjects with severe AD. Many experts have suggested that treatment early in the disease may be more likely to show clinical efficacy. Therefore, subjects with mild to moderate AD (MMSE 15-26, inclusive) rather than severe AD, will be enrolled in this trial. Over the 18-month treatment duration, placebo-treated subjects are expected to decline, on average, 3 points on the MMSE, with some subjects progressing more rapidly (eg, up to 5-6 point drop) from moderate AD to severe AD (MMSE scores<10). Since accurate testing with the trial outcome measures (eg, ADAS-Cog) becomes more challenging in subjects with severe AD, this protocol will not include those moderate AD subjects who are relatively close (MMSE 10-14) to a diagnosis of severe AD.

Subjects in the trial must meet criteria for a diagnosis of probable AD based on both the National Institute of Neurological and Communicative Diseases and Stroke/Alzheimer's Disease and Related Disorders Association (NINCDS-ADRDA) criteria and the Diagnostic and Statistical Manual of Mental Disorders, 4th Edition, Text Revision (DSM-IV-TR) criteria. Subjects with atypical variants of AD, such as posterior cortical atrophy and logopenic primary progressive aphasia, do not meet these criteria and will not be included. Disease severity and progression in such variants are not adequately captured with the outcome measures used in this trial.

Subjects who are receiving cholinesterase inhibitors and/or memantine for AD may be enrolled in this trial, although concomitant administration of these medications is not a requirement. If subjects are receiving cholinesterase inhibitors and/or memantine, then the dose must have been stable for at least the three months prior to Screening. The dose must not be changed, nor should medication be discontinued, unless medically necessary to ensure subject safety. **Subjects must not be started on, or switched to another cholinesterase inhibitor or memantine, after randomization into Part I of the trial.** During Part II, subjects may start or switch to another cholinesterase inhibitor or memantine. Dose alterations during the trial increase the difficulty in determining whether changes in efficacy or safety are due to trial medication or to the change in treatment. Investigators should first discuss with subjects and family members during the screening period, whether such therapy should be initiated prior to participation in

this trial. Other additional treatments must not be initiated during the trial except as otherwise specified in this protocol.

Medical foods/supplements (eg Vitamin E), and/or herbal medications for AD (eg, Axona®, Souvenaid®) should also be stable for at least three months prior to screening and should not be changed during Part I of the trial unless medically necessary.

Details about specific benefits and risks for subjects participating in this clinical trial can be found in the accompanying Investigator's Brochure and Informed Consent documents.

5.3 Trial Design Rationale

Part I of this trial is a 78-week double blind, placebo-controlled trial to evaluate the efficacy of BACE inhibitor MK-8931 as a potential disease-modifying therapy in subjects with mild to moderate AD. Part I is powered to detect a clinically significant change in the two coprimary outcome measures (ADAS-Cog and ADCS-ADL change-from-baseline scores at Week 78).

This initial efficacy trial includes a large sample size and long treatment duration compared with many Phase 2 trials, which, given the slowly progressive nature of Alzheimer's disease, is necessary in order to adequately assess efficacy and safety/tolerability. Therefore this trial is also designed as a pivotal Phase 3 trial. Key considerations for size and duration include the requirement to demonstrate clinically relevant progression of disease in the placebo group and slowing of progression in the treated group. Other designs involving shorter treatment duration and fewer subjects were evaluated. In particular, biomarkers have been proposed as potential predictors of disease progression and markers of treatment efficacy. However, no biomarker has yet been validated as surrogate marker for efficacy. Other options, such as trials with smaller sample sizes or shorter durations, would not be sufficiently statistically powered to demonstrate efficacy at clinically relevant effect sizes. Overall, given that AD is a fatal condition with no known cure, testing the efficacy of BACE inhibition in slowing or halting AD progression in a robust, efficient, and timely manner is a matter of critical importance to patients and clinicians.

To provide a further assessment of the safety and tolerability of MK-8931 during chronic treatment prior to allowing unrestricted enrollment, the first 200 subjects (~50 subjects randomized to one of three doses of MK-8931 or placebo) will be monitored more frequently during their first 13 weeks of treatment. A limited number of sites (approximately 75-125) are expected to recruit these subjects during the initial 6 to 8 months of the trial. When these 200 subjects have had the opportunity to complete 13 weeks of treatment, the first formal interim safety analysis will be performed by an external data monitoring committee (**eDMC**), which may recommend dropping the more frequent safety monitoring (see **Section 2.2**).

Enrollment will continue at those initial sites after the first 200 subjects have been randomized but prior to the first formal interim safety analysis. These additional subjects will also undergo the more frequent safety monitoring schedule. This continued enrollment will yield safety data of less than 13 weeks of treatment on approximately 200 additional subjects which will supplement the interim safety data provided to the eDMC. The two groups enrolled prior to the first formal interim safety analysis (approximately 400 in all) will be designated as the leading **Safety Cohort**.

The remainder of the sites (~85-115) will begin screening subjects for the **Main Cohort** (which includes all subjects enrolled after the first formal safety interim analysis) while awaiting a favorable recommendation of the eDMC to continue the trial (~9 to 12 months after the trial has started). Initial sites for the Safety Cohort can also screen subjects for the Main Cohort around the same time. Main Cohort subjects may only be randomized after the eDMC's review and approval.

The current trial incorporates a staggered enrollment approach that assesses safety and tolerability in a restricted cohort for an extended period prior to allowing a less restricted enrollment into the larger trial. We have considered the use of a separate trial of similar duration (ie, three months) conducted independently of this trial in lieu of the restricted cohort approach, but believe the planned approach offers several advantages. These include 1) the leading cohort can be followed for safety not only for the initial three months as would be typical in an exploratory separately conducted trial, but in an ongoing fashion over the entire 18 month period of the trial, providing a longer and more comprehensive assessment as the trial proceeds; 2) by using a single trial design with common sites and enrollment criteria, the comparability of data from the leading cohort is expected to be more similar to and hence predictive of the safety/tolerability profile for the overall study population than could be expected of data from a separately conducted trial; 3) the safety and tolerability data will also be able to be combined with that of later enrolled subjects in an ongoing way as well as at trial end, providing a more powerful means to assess the safety and tolerability of the drug for trial participants.

Details of safety monitoring including cognitive safety are provided below as well as in the eDMC charter. Briefly, the Safety Cohort in the current protocol has a 95% probability of detecting a serious adverse event if the true frequency is 1/150. Regarding cognitive adverse events, a recently completed Phase 3 trial of a gamma secretase inhibitor found evidence for cognitive worsening in AD patients on active treatment[(5)]. These adverse effects were not seen in a Phase 2 safety trial of this gamma secretase inhibitor. In the current trial, the independent eDMC will use periodic unblinded interim analyses on the ADAS-Cog in an effort to detect early signs of cognitive deterioration (see **Section 8.2.9** below for details) as well as other deleterious medical effects due to active treatment.

Subjects who complete the 78 weeks of treatment of Part I and tolerate study medication may choose to participate in the trial extension (Part II) which has an expected maximum duration of approximately 260 weeks, with the duration of individual subject participation dependent on the timing of enrollment in Part II. Subjects who did not complete Part I of the study will not be permitted to continue in Part II. In addition, subjects who are less than 75% compliant with trial medication in Part I or who repeatedly deviate from protocol requirements will not be permitted to continue in Part II, except in special circumstances, which will require Sponsor approval . Additional details pertaining to the trial extension can be found in Section 11.0.

5.4 Outcome Measures Rationale

The primary clinical measures in this trial are the Alzheimer's Disease Assessment Scale Cognitive subscale (ADAS-Cog) and the Alzheimer's Disease Cooperative Study Activities of Daily Living Inventory (ADCS-ADL). The ADAS-Cog is a measure of cognition while the ADCS-ADL is a measure of functional ability in activities of daily living. A key secondary endpoint will be the Clinical Dementia Rating Sum of Boxes (CDR-SB) scores, a measure of global function, including daily activities and cognitive abilities. All three measures are validated instruments that are acceptable to regulatory agencies, have been used previously in drug trials in this patient population, and can reliably detect clinically relevant changes in AD.

This trial will also assess the effects of MK-8931 on biomarkers related to AD pathology. The Sponsor hypothesizes that BACE inhibition, in addition to slowing clinical deterioration, will slow the progression of AD pathological changes. It is further hypothesized that clinical changes are correlated with biomarker changes. Three types of biomarkers appear to reflect various aspects of AD biology. First, brain volumetric measures assess brain tissue loss, presumably due to neuronal loss from the neurotoxic effects of amyloid. Therefore, volumetric measures that correlate with disease progression (including hippocampal, lateral ventricular, and total brain volumes) will be assessed using magnetic resonance imaging (MRI). Second, the inhibition of BACE by MK-8931 should reduce synthesis of Aβ. Based on modeling of amyloid dynamics, reduced synthesis may reduce total amyloid load. Thus, the amount of cortical amyloid deposition will be measured using positron emission tomography (PET) and [^{18}F]Flutemetamol as the amyloid-imaging PET ligand. Finally, CSF Aβ and tau measures are included as biomarkers since both are abnormal in AD and have been associated with different aspects of AD biology. CSF levels of tau, in particular, are thought to reflect brain tau pathology, which is correlated with cognitive decline(6). BACE inhibition, by reducing Aβ, may limit downstream tau pathology and, thereby, reduce CSF tau levels. Therefore, CSF total tau levels will be included as a key biomarker. CSF levels of Aβ, in contrast, may be impacted more directly by BACE inhibition and will be used as a biomarker of target engagement.

Confidential

After the initiation of P017, the FDA issued guidance to sponsors indicating that all trials with BACE1 inhibitors that are longer than two weeks in duration must include routine monitoring of skin. There have been observations in the literature that mice lacking the gene for BACE2 have reduced pigment [30]. In recently completed studies of MK-8931 in two pigmented species (mouse and rabbit), reduced hair pigment was observed within four weeks of treatment in all animals, which was reversible with cessation of treatment. On microscopic examination, normal skin histology was observed except for loss of pigmentation in the hair follicle/shaft. There was no loss of melanocytes. All phases of the hair cycle were present, and no inflammatory or degenerative changes in hair follicles, sebaceous glands, epidermis, or dermis were observed. In contrast to these observations in mouse and rabbit, no change in hair pigment or skin and retinal histology have been observed in monkeys treated for up to 9 months with MK-8931 at exposures 36-fold above those achieved at the highest dose of MK-8931 tested in P017 (60 mg). Furthermore, no adverse events of skin hypo- or depigmentation have been reported thus far in prior human trials of MK-8931.

A review of preclinical data and the FDA request with a panel of expert dermatologists indicated the overall risk to patients is minimal. The risk of vitiligo, with loss of dermal melanocytes, was seen as low. If vitiligo were observed, medical management was recommended without discontinuation of study medication, given that vitiligo is not associated with significant morbidity or mortality. Hypopigmentation was viewed as being a more likely finding with BACE inhibitor treatment, rather than vitiligo. Hypopigmentation by itself was also not seen as requiring discontinuation of study medication. Therefore, baseline and post-treatment dermatology assessments were added to this protocol to document the incidence of hypopigmentation, not to detect a significant safety risk. Patients will be informed of the risk of hypopigmentation and instructed to use sun screen in affected areas if exposed to sunlight. A long-term risk may be increased skin cancer due to sun damage to the skin. Incident cases of hypopigmentation should be referred to a specialist for appropriate diagnosis and treatment, if clinically indicated. If no difference is seen in the occurrence of hypopigmentation between the placebo and MK-8931 groups after 300 subjects/group are evaluated at six months in the MK-8931 program (P017 and 019), the eDMC may recommend the discontinuation of the routine skin examinations in a future amendment. Sites would continue to collect skin-related adverse events and refer subjects for further dermatologic evaluation as needed.

5.5 Dose and Administration Rationale

The amount of Aβ reduction required to slow disease progression is unknown. The strategy for dose selection in this protocol is to use a dose that maximally inhibits CSF Aβ that is also thought to be safe and well tolerated. Initially, three doses will be evaluated in the leading Safety Cohort. Using PK/PD modeling and simulation, the 12 mg and 40 mg doses are projected to reduce CSF Aβ by at least 50% and 75%, respectively, in more than 90% subjects. The 60 mg dose will provide an exposure margin 1.5 times that of the 40 mg dose mean exposure (for AUC and Cmax). The 60mg dose also provides an exposure margin of approximately one times (AUC and Cmax) for subjects treated with the 40 mg dose who are in the top 5% in terms of exposure. Thus, the 60 mg dose arm in the Safety Cohort will yield safety and tolerability data relevant to longer term exposure with 40 mg to the subset of subjects with the highest drug exposures.

It is unknown how much Aβ lowering is optimal from the perspectives of both safety/tolerability as well as efficacy. Therefore, following the review of data from the leading Safety Cohort, two doses will be used for the Main Cohort. These include the 40 mg dose, which reduces CSF Aβ by >75%, and the 12 mg dose that produces a more moderate (>50%) reduction in CSF Aβ. Approval of these two doses by the siDMC and eDMC is required at the first formal safety interim analysis. A detailed description of the eDMC and the siDMC roles is included in their respective charters.

It is possible that the siDMC could select only one dose for the Main Cohort due to safety or other emerging data. It is also possible that if the 12 and 40 mg doses are taken forward, one dose could be discontinued later by the eDMC due to emerging safety or tolerability concerns. For clarity and simplicity, the protocol is written assuming that the 12 and 40 mg doses are advanced for dosing of the Main Cohort and that these two doses continue on until the end of the trial. Details about changes that would occur if only one dose is advanced for dosing in the Main Cohort are described in **Section 8.2.9**.

6.0 TRIAL OBJECTIVES AND HYPOTHESES (PART I)

6.1 Primary Trial Objectives and Hypotheses

Objectives:

1. To assess the efficacy of two doses of MK-8931 on cognition in subjects with mild to moderate AD.
2. To assess the efficacy of two doses of MK-8931 on functional ability in activities of daily living in subjects with mild to moderate AD.
3. To assess the safety and tolerability of three doses of MK-8931 in the treatment of subjects with mild to moderate AD.

Hypotheses:

1. At least one MK-8931 dose is superior to placebo at 78 weeks of treatment with respect to change from Baseline in ADAS-Cog score.
2. At least one MK-8931 dose is superior to placebo at 78 weeks of treatment with respect to change from Baseline in ADCS-ADL score.

6.2 Key Secondary Trial Objective and Hypothesis

Objective:

To assess the overall clinical response, as reflected by global assessment, of two doses of MK-8931 in subjects with mild to moderate AD.

Hypothesis:

At least one MK-8931 dose is superior to placebo at 78 weeks of treatment with respect to the change-from-Baseline in CDR-SB score.

6.3 Other Secondary Trial Objectives and Hypotheses

Objectives:

1. To assess the effect of two doses of MK-8931 on total hippocampal volume in subjects with mild to moderate AD.
2. To assess the effect of two doses of MK-8931 on CSF total tau in subjects with mild to moderate AD included in the CSF substudy.
3. To assess the effect of two doses of MK-8931 on cortical amyloid load assessed using [^{18}F]Flutemetamol and PET imaging in subjects with mild to moderate AD included in the PET substudy.
4. To assess the effect of two doses of MK-8931 on responder rates in subjects with mild to moderate AD.
5. To assess the efficacy of two doses of MK-8931 on neuropsychiatric and neuropsychological measures in subjects with mild to moderate AD.

Hypotheses:

1. At least one MK-8931 dose is superior to placebo at 78 weeks of treatment with respect to change-from-Baseline in total hippocampal volume.
2. At least one MK-8931 dose is superior to placebo at 78 weeks of treatment with respect to change-from-Baseline on CSF total tau.
3. At least one MK-8931 dose is superior to placebo at 78 weeks of treatment with respect to change-from-Baseline on cortical amyloid assessed with [^{18}F]Flutemetamol binding using PET.
4. At least one MK-8931 dose is superior to placebo at 78 weeks of treatment with respect to the proportion of responders based on a combination of ADAS-Cog and ADCS-ADL results. (see **Section 8.2.4** for the definition of a responder).

6.4 Exploratory Objectives

Other objectives of this trial are to assess the effect of MK-8931 on 1) other biomarkers (ie, regional [^{18}F]Flutemetamol binding using PET, other brain volumetric measures using structural MRI, and concentrations of CSF Aβ peptides and p-tau, 2) the correlation between biomarkers (eg, MRI measures) and disease progression (eg, ADAS-Cog scores), 3) HLA association with rash and 4) health economic outcomes.

7.0 INVESTIGATIONAL AND ANALYSIS PLAN

7.1 Overall Trial Design

Overview

This is a randomized, placebo-controlled, parallel-group, multi-site, double-blind trial of MK-8931 in subjects with mild to moderate AD.

Trial Governance Committees

There will be three committees (see **Section 10.3**) charged with interpreting and acting on the results of the various interim analyses (see **Section 8.2.9**). The first is the eDMC, which will receive unblinded reports from all interim analyses. Based primarily on safety considerations, the eDMC may make recommendations as to the future conduct of the trial (including, potentially, a recommendation to stop the trial). The second committee is the siDMC of the Sponsor, which will only review unblinded data on the first 200 subjects enrolled. The siDMC will be primarily responsible for the recommendations to the eDMC to continue the trial at the first formal interim analysis, thus opening up enrollment to the remainder of sites and subjects (Main Cohort). The siDMC will also recommend which active dose group(s) should be used for the Main Cohort. Since the first 200 subjects will be excluded from the primary efficacy and safety analyses, the siDMC will also periodically review the accruing data of these first 200 subjects as the trial progresses. The third committee is an Executive Oversight Committee (**EOC**) of the sponsor which will receive recommendations throughout the trial from the eDMC and is responsible for acting upon the recommendations of the eDMC. The composition, activities, and responsibilities of these trial governance committees will be described in the eDMC and siDMC charters.

The eDMC and the siDMC will review data in a manner such that the trial team, investigators, subjects, and vendors will not have access to the unblinded data.

The EOC will not have access to unblinded data or reports unless it is deemed necessary by the eDMC in order to act upon an eDMC recommendation. Both the siDMC and the EOC will be completely independent of, and separate from, the trial team performing the medical monitoring and supervising the operational aspects of the protocol.

Summary

Following a Screening Period of up to four weeks (up to three months with sponsor's permission when needed), approximately 400 eligible subjects will be randomized in a 1:1:1:1 ratio to one of four double-blind treatment arms (three doses of MK-8931, or placebo) for the Safety Cohort. For the Main Cohort, eligible subjects will be randomized in a 1:1:1 ratio to one of the remaining three double-blind treatment arms (12 and 40 mg MK-8931, or placebo). Throughout Part I of the trial, subjects must remain on the AD treatment and dose (if any) that they are receiving at Screening and additional AD treatments must not be initiated or stopped during the trial. During Part II, subjects will be permitted to change or start AD treatments.

During the 78-week double-blind Treatment Period, all subjects will return for visits at the end of Weeks 2, 4, 13, 26, 39, 52, 65, and 78, and will be contacted by telephone at the end of Weeks 18, 32, 45, 58, and 72 to assess AEs, trial medication compliance and concomitant medications. Subjects in the Safety Cohort will have an additional visit at the end of Week 8 and will be contacted by telephone at the end of Week 6 and Week 10. At the end of Week 8, subjects in the Main Cohort will have a telephone contact rather than a visit.

At the end of the 78-week Treatment Period (Part I), subjects who have completed the treatment and tolerated study medication may be eligible for enrollment in the trial extension (Part II), during which all subjects who received placebo during Part I will receive active drug. Subjects who did not complete Part I of the study or who repeatedly deviate from protocol requirements will not be permitted to continue in Part II. In addition, subjects who are less than 75% compliant with trial medication in Part I will not be permitted to continue in Part II, except in special circumstances, which will require Sponsor approval. Additional details pertaining to Part II can be found in **Section 11.0**.

The two primary efficacy endpoints are the ADAS-Cog and ADCS-ADL scores. This trial is powered to detect a slowing of disease progression of approximately 2 points for ADAS-Cog and 3.4 points for ADCS-ADL at the end of 78 weeks (18 months), which, based on prior studies, is a clinically significant effect. Assuming an 18-month placebo-progression rate of approximately 6.1 points for the ADAS-Cog, and -9.6 points for the ADCS-ADL, these changes correspond to roughly a 35% slowing of the disease progression. The Key Secondary Efficacy Endpoint is the CDR-SB score. Additional Secondary and Exploratory Efficacy Endpoints include the percentage of responders based on ADAS-Cog and ADCS-ADL scores, hippocampal volume as measured using structural MRI, the NPI and MMSE scores; [^{18}F]Flutemetamol binding assessed using PET; brain volumetric measures (including ventricular and whole brain volume) assessed using structural MRI; CSF biomarker concentrations; and health economic and quality of life outcomes a modified Resource Utilization in Dementia [RUD] Lite Questionnaire, the Health Economic Assessment [HEA]) and the EuroQol Five Dimension Questionnaire (EQ-5D).

Safety and tolerability will be assessed by a clinical review of all relevant parameters including AEs, laboratory tests, vital signs, and ECG measurements. Safety Endpoints of Special Interest based on a general concern about compounds expected to reduce Aβ in the brain include 1) microhemorrhage, superficial siderosis or macrohemorrhage; and 2) vasogenic edema; and Safety Endpoints of Special Interest based on safety findings from clinical pharmacology studies include 3) delirium; and 4) rash Events of Clinical Interest (ECI; see **Section 7.7.2.2.3**). Details of related safety findings are provided in the Investigator's Brochure and described below.

Regarding MRI monitoring, the US FDA had noted the occurrence of imaging abnormalities believed to represent cerebral vasogenic edema in association with the investigational use of compounds that are intended to treat AD by reducing β-amyloid in the brain. These imaging abnormalities, described by Salloway et al.[(1)], have, in the majority of instances, been asymptomatic and their presence has been detected by routine MRI scans. Symptoms, when present in association with such imaging abnormalities, have been reported to include headache, worsening cognitive function, alteration of consciousness, seizures, unsteadiness, and vomiting. In most instances, the occurrence of such imaging abnormalities, even when symptomatic, has not required treatment beyond discontinuation of the investigational compound, with imaging abnormalities then resolving; infrequently, high-dose steroid therapy has been administered in the presence of prominent symptoms.

Previous regulatory guidance required that all subjects have an MRI scan during Screening in order to qualify for the trial and, subsequently, routine MRI scans for safety monitoring. Following a comprehensive review of the existing data, the FDA updated their guidance stating that serial clinical and MRI monitoring was no longer required as a matter of course in clinical trials of small molecule drugs that may affect β-amyloid. As a result routine MRI safety monitoring was removed from this study. MRI scans will continue to be included at Screening for eligibility and brain structure volumetric assessment and at the end of treatment visit for evaluation of the volumetric outcome measures. MRIs may also be performed at a post-treatment visit for safety monitoring if clinically indicated as determined by the investigator (e.g., in follow-up to an AE). Amyloid-related imaging abnormalities that arise during the trial should be handled as follows.

1) Trial medication should be discontinued if an imaging abnormality consistent with macrohemorrhage appears or a **clinically symptomatic** incident vasogenic edema, microhemorrhage, or superficial siderosis is seen.

2) If symptomatic cerebral vasogenic edema occurs, MRI scans should be repeated within three to four weeks to assess stability and then performed every four to six weeks (or as clinically indicated) until the vasogenic edema resolves. Treatment with high dose dexamethasone can be considered, as suggested by the US FDA, if associated symptoms are severe.

3) For subjects who present with new clinically symptomatic microhemorrhages or superficial siderosis, an MRI re-scan at three to four weeks should be performed in order to evaluate their stability.

4) Re-dosing can be considered with Sponsor approval based on investigators' clinical judgment if clinical symptoms associated with symptomatic vasogenic edema, microhemorrhage, or superficial siderosis have resolved.

5) For macrohemorrhage (symptomatic or asymptomatic), subjects must be discontinued from trial medication and cannot resume dosing. They may continue with study visits as part of the retrieved drop out aspect of the trial.

In 3- and 6-month toxicity studies in rats, histomorphologic evaluation of the eyes revealed changes in the retina. These findings were characterized by bilateral hypertrophy of the retinal pigment epithelium (RPE) and areas of focal cellular accumulations consisting of large round cells that appeared to originate from the hypertrophied RPE layer. In some areas, the cellular accumulations resulted in secondary focal disruption of the overlying retina. Importantly, there was no evidence of neuronal necrosis in any animal (see Investigator's Brochure for details). In Phase 1 clinical trials of up to 28 days treatment with MK-8931, incidence and severity of AEs related to eye disorders were unremarkable (see Investigator's Brochure for details), though the effects of treatment for longer periods is uncertain. Nevertheless, retinal monitoring with appropriate ophthalmologic examinations was initially included in this trial to provide long-term data for ocular safety.

Ophthalmologic evaluation of subjects with AD can be challenging. Experts have noted that assessment of visual acuity can be confounded by cognitive deficits[20] which could limit its usefulness for assessing drug effects. On the other hand, SD-OCT is a sensitive method for evaluating retinal structure with micrometer resolution. Therefore, SD-OCT measurements of RPE thickness were initially included to assess MK-8931's effect on the RPE layer. Additional ophthalmologic assessments included visual acuity test, SD-OCT measurements of other retinal layers, posterior eye exam including dilated funduscopy, and fundus photography. Fundus autofluorescence (FAF) was also included at centers with the appropriate capabilities. Ophthalmologic assessments were initially conducted at ophthalmology centers at Screening, Week 13, 26, 52 and 78, and Early Termination Visit.

Confidential

Based on the recommendation of the eDMC in March 2015, after reviewing results from this trial and available data from an ongoing trial in prodromal AD patients (MK-8931-019) the following modifications will be applied to ophthalmologic monitoring

- New subjects screened under Amendment 13 will not have any ophthalmic assessments performed in the trial. In addition, subjects who are in screening when Amendment 13 is approved and have not yet undergone ophthalmic assessments will not have any ophthalmic assessments performed.

- Subjects enrolled prior to Amendment 13 who had ophthalmic procedures completed at screening will have the following procedures performed at the Week 78 Visit: visual acuity, posterior eye exams, fundus photography, and SD-OCT. Fundus autofluorescence will also continue to be performed at Week 78 at sites who have this capability. Sites will obtain these Week 78 assessments, but in cases where this is operationally infeasible (e.g., subject uncooperative), these assessments may be omitted with Sponsor approval. The rationale for the continued ophthalmic assessments in this cohort is to collect further longitudinal data; it is not required for routine safety monitoring. Therefore, subjects are NOT required to discontinue from the trial if for some reason the ophthalmic procedures cannot be obtained at Week 78.

- Subjects enrolled prior to Amendment 13 who had ophthalmic procedures completed at screening and discontinue prior to Week 78 will not have further ophthalmic examinations completed.

Preclinical studies initially noted increased thickness in the retinal pigment epithelium in rodents and slight reduction of iris pigment in rabbits, as described in the IB. More recent studies failed to replicate the effect on iris pigment (see IB).

To account for potentially different rates of cognitive decline and treatment response, subjects will be stratified at Randomization by the severity of their disease as measured by MMSE score at Screening (mild AD: $20 < \text{MMSE} \leq 26$; moderate AD: $15 \leq \text{MMSE} \leq 20$) and AD treatment at Screening (use of AChEI or memantine; no use of AChEI or memantine). Randomization will also be stratified by geographic region (US/Canada, Europe/Australia/New Zealand, Japan, Rest of the World).

Both the percent of mild AD subjects and the percent of moderate AD subjects, respectively, will be no more than 60% of the total.

All subjects will be required to provide blood samples for apolipoprotein E (APOE; a risk factor gene for AD) genotyping. The effects of APOE genotype on endpoints and response to treatment will be evaluated in exploratory analyses.

Subjects will be asked to provide blood samples for pharmacokinetic (PK) analyses to determine plasma concentrations of MK-8931. This information will be used to develop a population PK model and to explore the exposure-response relationship in the trial population. Pharmacokinetic blood samples will be collected at the following visits:

- Baseline/Randomization Visit
- Week 2
- Week 8 (Safety Cohort Only)
- Week 13
- Week 26
- Week 52
- Week 65
- Week 78
- Early Termination Visit (Part I only)
- Unscheduled Visit (Part I only, PK blood samples should be collected at the investigators discretion when an AE considered possibly related to study medication is associated with the unscheduled visit)
- Week 82
- Week 91

Under Amendment 17, both plasma and dried blood spot (DBS) PK samples will be collected at each timepoint as noted in the Trial Flow Charts. Details regarding use of samples for PK and PK/PD modeling will be specified in the modeling analysis plan document.

For subjects who sign a separate consent form for pharmacogenetic analysis, blood samples for pharmacogenetic analysis will also be collected.

Three types of interim analyses (IAs) will be performed during this trial: 1) the first formal analysis for safety and dose selection, 2) multiple ongoing safety analyses and 3) one futility analysis. Trial enrollment will continue during all interim analyses. The siDMC will only review results from the first 200 subjects enrolled. Additional details pertaining to the endpoints, timing, and purpose of the interim analysis are summarized in **Section 8.2.9**, with further details to be included in the siDMC and eDMC charters.

Quality control is an essential part of all clinical trials. For AD trials, it is particularly important to monitor a) whether subjects selected for randomization meet entry criteria and b) clinical ratings. This trial will include a review by outside expert(s) of each subject prior to randomization. This review may include all available medically relevant data, a narrative summary of the subject's history, and a review of video/audio recordings of key clinical interviews performed at the Screening Visit. These interviews may include the MMSE, the CDR, the ADCS-ADL, and the ADAS-Cog assessments. For the Main Cohort, only audio recordings will be used for the review. Safety Cohort recordings will be switched to only audio before the Main Cohort is initiated.

Raters of clinical assessments will undergo the applicable training prior toconducting assessments in the trial. Further, rater performance on these assessments will be carefully evaluated and monitored to ensure and maintain adequate reliability throughout the trial. Details will be specified in the **Manual of Assessments**. In order to qualify for the trial, raters must be approved by the sponsor, which will typically require successful completion of a trial specific rater training program. A recorded practice interview of the ADAS-Cog may also be required for some raters. This recording may be performed with a colleague who simulates an AD patient. Details will be specified by the sponsor prior to site initiation. To ensure the continued quality of the assessments (MMSE, CDR, ADAS-Cog, and ADCS-ADL), raters will be asked to video/audio record interviews and ratings at some or all visits. Some or all of these recorded interviews will be reviewed by outside experts. For the Main Cohort, only audio recordings will be used for the review. Safety Cohort recordings will be switched to only audio before the Main Cohort is initiated. Raters will be provided feedback on the quality of their interviews and ratings by the outside experts by e-mail, telephone or in meetings in order to develop and maintain good rater reliability. Based on this feedback, raters may change their initially recorded scores if errors are identified. Routine rater meetings may be conducted to assess and maintain reliability for the duration of the trial. Raters who do not perform adequately may be required to undergo additional remediation or may be replaced.

While concerns have been raised that video/audio recordings could theoretically compromise subjects' privacy, this issue must be balanced with the needs to conduct methodologically adequate and scientifically rigorous trials that are capable of testing the key hypotheses. Given that the key endpoints in this trial involve subjective judgments, monitoring the adequacy of subject interviews and ratings is essential and part of good research methodology. Prior studies have clearly demonstrated that the failure to adequately monitor such ratings can substantially increase the risks of failed trials[(7,8)]. Recorded interviews will be encrypted using state of the art methods to ensure privacy. Recordings will only be reviewed by approved trial personnel for quality control purposes and will be destroyed within two years of the completion of the trial unless local regulatory authorities or Institutional Review Boards or Independent Ethics Committees (IRBs/IECs) have different requirements for storage.

Subjects may participate in this protocol and continue to participate in certain observational studies initiated prior to Protocol 017, if approved by the Sponsor. These studies must involve only limited cognitive testing (eg, annual) and subjects will not be permitted to undergo any non-protocol cognitive testing within 2 months prior to the Baseline and End of Treatment visits.

Substudies

Two substudies are planned for Part I of this trial: 1) an amyloid imaging substudy to evaluate changes in amyloid load using PET and [^{18}F]Flutemetamol in the Main Cohort only; and 2) a CSF biomarker substudy to evaluate changes in CSF concentrations of Aβ40, Aβ42, sAPPβ, total tau, and phosphorylated tau (p-tau) in both the Safety Cohort and the Main Cohort. These biomarkers may be useful for assessing the pharmacodynamic and/or disease modifying effects of MK-8931. They may also be useful for assessing endogenous amyloid load and diagnostic accuracy. At designated sites, all subjects will be asked to participate in the relevant substudy (up to 100 subjects/arm for the PET substudy). Subjects may be asked to participate in one or both of the substudies depending on the substudies designated for that site and the stage of the trial (Safety Cohort vs. Main Cohort). Subjects are not required to participate in a substudy to participate in the larger trial.

Subjects who choose to participate in the amyloid imaging substudy must sign an informed consent form and undergo a PET scan following an i.v. injection of [^{18}F]Flutemetamol at the Screening and Week 78 visits.

Subjects who choose to participate in the CSF biomarker substudy must sign an informed consent form. A lumbar puncture for the collection of CSF will be performed at the Screening and Week 78 visits.

7.2 Beginning and End of the Trial (Part I)

Each subject is considered to be enrolled in the trial when the subject (or the subject's legal representative) has provided written informed consent **in accordance with local requirements**.

Each subject is considered to have ended participation in the trial when he/she has completed the last protocol-specified contact (eg, visits or telephone contacts) or has prematurely discontinued from the trial. A subject will be considered a completer of Part I if he/she has completed the Week 78 visit and did not discontinue trial medication during the trial.

A subject is considered to have discontinued after he/she has withdrawn consent or has been discontinued under the conditions specified in Section 7.3.3. In order to better adhere to the intent-to-treat analysis principle, subjects who discontinue trial medication prior to the end of the initial 78-week treatment period but are willing to return to the trial site should continue with scheduled visits as part of the retrieved dropout component of the trial. All applicable activities scheduled for the final trial

Confidential

visit should be performed at the time of treatment discontinuation. At subsequent scheduled visits, sites should perform the ADAS-Cog, ADCS-ADL, CDR-SB, MMSE, NPI, C-SSRS, concomitant medication review and continue to monitor for AEs. The HEA, modified RUD Lite, and EQ-5D should also be performed, if possible. The following procedures may be conducted, but are not required at these subsequent visits: vital signs, body weight, physical and neurological exams, ECG, structural MRI, PET, CSF collection, blood and urine safety labs, and PK specimens, except as needed to follow-up on AEs.

A subject is considered to have been lost to follow-up if he/she is unable to be contacted by the investigator. The end of participation for a subject lost to follow-up is the last known contact (eg, visit or telephone contact).

The overall trial begins when the first subject is enrolled (ie, signs the informed consent form). The overall trial ends when the last remaining subject has ended participation in the trial, by completing the trial, by discontinuing from the trial, or by being declared as lost to follow-up. At the end of the initial 78-week treatment period (Part I), subjects who have completed treatment and tolerated study medication may be eligible to participate in the trial extension (Part II). Subjects who did not complete Part I of the study will not be permitted to continue in Part II. In addition, subjects who are less than 75% compliant with trial medication or who repeatedly deviate from protocol requirements in Part I will not be permitted to continue in Part II, except in special circumstances, which will require Sponsor approval . Details pertaining to Part II can be found in Section 11.0.

Each subject will be monitored for the occurrence of AEs beginning immediately after the subject has signed informed consent through 14 days after the last protocol-specified visit. Follow-up procedures related to pregnancy or existing SAEs may continue beyond the end of the clinical trial.

Each subject will participate in Part I for approximately 20 months from the time the subject signs the Informed Consent Form (ICF) through the final protocol-specified contact for Part I.

7.3 Trial Population

The trial population is adult subjects with a diagnosis of mild to moderate AD.

7.3.1 Subject Inclusion Criteria

A subject must meet all the criteria listed below to participate in the trial.

1. Each subject must be ≥ 55 to ≤ 85 years of age at the first visit.
2. Each subject must meet the criteria for a diagnosis of probable AD based on both a) the National Institute of Neurological and Communicative Diseases and Stroke/Alzheimer's Disease and Related Disorders Association (NINCDS-ADRDA) criteria (see **Appendix 3**) and b) the Diagnostic and Statistical Manual of Mental Disorders, 4th Edition, Text Revision (DSM-IV-TR) criteria for AD.
3. Each subject must have an MMSE score ≥ 15 and ≤ 26 at Screening
4. Each subject must have a clear history of cognitive and functional decline over at least one year that is either a) documented in medical records or b) documented by history from an informant who knows the subject well.
5. Each subject must have an MRI scan at the Screening Visit that is consistent with a diagnosis of AD. MRI scans or head CT obtained at Screening or within 12 months before Screening (with Sponsor approval) are acceptable alternatives and must be submitted for central review.
6. Each subject must be able to read at a 6th grade level or equivalent, as determined by the investigator, and must have a history of academic achievement and/or employment sufficient to exclude mental retardation.
7. If a subject is receiving an acetylcholinesterase inhibitor, memantine, medical food/supplement (eg Vitamin E), and/or herbal medications for AD, the dose must have been stable for at least three months before Screening, and the subject must be willing to remain on the same dose for the duration of the trial. Subjects may need to be on AD treatments **in accordance with local requirements**. (The treatment and dose at Screening must not be changed during the trial unless medically necessary to ensure subject safety. Additional treatments [including herbal medications] for AD that are not specified in the protocol must not be initiated during the trial.) The subject and caregiver must agree that they do not plan to discontinue treatment or initiate additional AD treatments during the trial unless medically necessary.
8. Each subject must have a reliable and competent trial partner/caregiver who must have a close relationship with the subject, have face to face contact at least three days a week for a minimum of six waking hours a week (or more in accordance with local requirements), be willing to accompany the subject to all required trial visits, and be willing to monitor compliance of the administration of the trial medication. The trial partner/caregiver should understand the nature of the trial and adhere to trial requirements (eg, dose, visit schedules, and evaluations).
9. Each subject must have results of clinical laboratory tests (complete blood count [CBC], blood chemistries, thyroid stimulating hormone [TSH], and urinalysis) within normal limits or clinically acceptable to the investigator at Screening.

10. Each subject must have results of a physical examination, vital signs, and ECG within normal limits or clinically acceptable to the investigator at Screening.
11. Each female subject is not of childbearing potential as indicated by one of the following
    1. has reached natural menopause (defined as $\geq$ 46 years of age with either
        a. $\geq$ 12 months of spontaneous amenorrhea or
        b. $\geq$ 6 months of spontaneous amenorrhea with serum follicle stimulating hormone (FSH) levels $>$ 40 IU/L as determined by the central laboratory). Pregnancy is to be ruled out by a negative serum βhCG before the first administration of trial medication.
    2. has had a hysterectomy;
    3. has had a bilateral tubal ligation; or
    4. has had a bilateral oophorectomy (with or without a hysterectomy) and greater than 6 weeks have passed since the surgery.
12. Each subject must be willing to provide a blood sample for APOE and HLA genotyping.
13. Based on the investigator's judgment, each subject is able to speak, read, hear, and understand the language of the trial staff and the informed consent form, and possess the ability to respond verbally to questions, follow instructions, and complete questionnaires. Each subject must also be able and willing to adhere to dose and visit schedules and to consent to video/audio recording of selected interviews.
14. Each subject (or legal representative) must sign the informed consent form **in accordance with local requirements**, after the scope and nature of the investigation have been explained to them, and before Screening assessments.

    Subjects who agree to participate in pharmacogenetic testing must give written informed consent for pharmacogenetic testing. Subjects who are unwilling to sign the informed consent for pharmacogenetic testing may be included into the trial, however, pharmacogenetic samples must not be obtained.

Additional Inclusion Criterion for Safety Cohort

15. Each subject must have a reliable and competent trial partner/caregiver who has a close relationship with the subject, has face to face contact at least five days a week for a minimum of 10 waking hours a week, is willing to accompany the subject to all required trial visits, and is willing to monitor compliance of the administration of the trial medication. The trial partner/caregiver should understand the nature of the trial and adhere to trial requirements (eg, dose, visit schedules, and evaluations).

7.3.2 Subject Exclusion Criteria

A subject meeting any of the exclusion criteria listed below must be excluded from participating in the trial:

1. The subject has a Rosen-modified Hachinski Ischemia Score > 4 at Screening (ie, evidence of vascular dementia).
2. The subject has a known history of stroke or evidence from screening imaging scan (e.g. MRI scan or CT) that is clinically important in the investigator's opinion.
3. The subject has evidence of a clinically relevant neurological disorder other than the disease being studied (ie, probable AD) at Screening, including but not limited to: vascular dementia, parkinsonism, frontotemporal dementia, Huntington's disease, amyotrophic lateral sclerosis, multiple sclerosis, progressive supranuclear palsy, neurosyphilis, dementia with Lewy bodies, posterior cortical atrophy, logopenic primary progressive aphasia, other types of dementia, mental retardation, hypoxic cerebral damage, cognitive impairment due to other disorders, or head trauma with loss of consciousness that led to persistent cognitive deficits.
4. The subject has a history of seizures or epilepsy within the last 5 years before Screening.
5. The subject has evidence of a clinically relevant or unstable psychiatric disorder, based on DSM-IV-TR criteria, including schizophrenia or other psychotic disorder, bipolar disorder, major depression, or delirium. Major depression in remission is not exclusionary.
6. The subject has evidence of a current episode of major depression based on investigator's judgment. A score on the 15-item Geriatric Depression Scale of 5 or more requires an assessment by an appropriate health care professional to evaluate for the presence of major depression. Subjects with a score of 5 or more who are not diagnosed with major depression following such an assessment may be included in the trial.
7. The subject is at imminent risk of self-harm, based on clinical interview or on the Columbia Suicidality Severity Rating Scale (C-SSRS), or of harm to others in the opinion of the investigator. Subjects must be excluded if they report suicidal ideation with intent, with or without a plan or method (eg positive response to items 4 or 5 in assessment of, suicidal ideation on the C-SSRS) in the past 2 months or suicidal behavior in the past 6 months.
8. The subject has a history of alcoholism or drug dependency/abuse within the last 5 years before Screening.

9. The subject does not have an MRI scan obtained within 12 months before Screening available for central review and is unwilling or not eligible to undergo an MRI scan (eg, metal implants, obesity) at the Screening Visit (see **MRI Procedure Manual and Imaging Charter** for details). Exception: A head CT scan obtained at Screening or within 12 months before Screening may be accepted for evaluation of the eligibility criteria on a case-by-case basis, as approved by the Sponsor (e.g., when MRI is contraindicated for the subject). The head CT must be suitable for central review.
10. The subject's MRI scan obtained at Screening shows evidence of a neurological disorder other than probable AD or
    - evidence of a prior macrohemorrhage,
    - symptomatic vasogenic edema in the investigator's judgment,
    - > 3 lacunar infarcts over 10 mm each, or
    - any other clinically significant finding that may account for their cognitive impairment, including but not limited to: brain tumor, large or strategically located cortical or subcortical infarct, or severe white matter disease equaling a rating of 3 on the age-related white matter changes (ARWMC) scale.

    An MRI that was done within 12 months before the Screening visit that is available for review by the central MRI reading vendor is also acceptable for evaluation of the eligibility criteria. A head CT scan obtained at Screening or within 12 months before Screening may be accepted instead of MRI on a case-by-case basis, as approved by the Sponsor (e.g., when MRI is contraindicated for the subject).
11. The subject at Screening has
    1. alanine aminotransferase (ALT) ≥ 3 x upper limit of normal (ULN), OR
    2. aspartate aminotransferase (AST) ≥ 3 x ULN, OR
    3. total bilirubin (T-BIL) ≥ 1.5 x ULN.

    Should a liver function test (LFT) be abnormal (ALT/AST >ULN but < 3x ULN, T-BIL > ULN but < 1.5 x ULN) at Screening but not meet the specified criteria, the investigator should attempt to characterize at entry the reason(s) for the elevation (eg, alcohol abuse, metabolic syndrome with fatty liver, etc). Subjects with suspected Gilbert's Syndrome who have isolated T-BILI ≥ 1.5 x ULN may enter the trial upon genetic confirmation (eg, uridine diphosphate glucuronosyltransferase 1A1 [UGT1A1] assessment).
12. The subject has a history of hepatitis or liver disease that, in the opinion of the investigator, has been active within the six months prior to Screening.

13. The subject has a recent or ongoing, **uncontrolled, clinically significant** medical condition within **3 months** of the Screening Visit (such as, but not limited to, diabetes, hypertension, thyroid or endocrine disease, congestive heart failure, angina, cardiac or gastrointestinal disease, dialysis, or abnormal renal function with estimated creatinine clearance < 30 mL/min)) other than the condition being studied such that, in the judgment of the investigator, participation in the trial would pose a significant medical risk to the subject. Controlled co-morbid conditions (including diabetes, hypertension, heart disease, etc) are not exclusionary if stable within three months of the Screening Visit. All concomitant medications, supplements (eg Vitamin E), or other substances must be kept as stable as medically possible during the trial.

Note: urinary tract infections at screening are not exclusionary if adequately treated (as documented by repeat urinalysis) prior to baseline.

14. The subject has a history or current evidence of long QT syndrome, QT_C interval ≥ 470 milliseconds (for male subjects) or ≥ 480 milliseconds (for female subjects), or torsades de pointes. (Note: Determination of QTc interval at Screening will be based on the average of three measurements, using the Fridericia formula for correction.) Subjects with stable bundle branch block who exceed these limits for QTc interval are eligible for the trial if judged by an expert in cardiology not to be at increased risk for Torsades.
15. The subject has a history of malignancy occurring within the five years immediately before Screening, except for a subject who has been adequately treated for
    1. basal cell or squamous cell skin cancer,
    2. in situ cervical cancer, or
    3. localized prostate carcinoma; or
    4. who has undergone potentially curative therapy with no evidence of recurrence for ≥ 3 year post-therapy, and who is deemed at low risk for recurrence by her/his treating physician.
16. The subject has
    1. clinically significant vitamin B12 or folate deficiency in the six months immediately before Screening, or
    2. vitamin B12 or folate deficiency in addition to increased serum homocysteine or methylmelonic acid levels at Screening as determined by central laboratory normal values.
17. The subject is pregnant, is attempting to become pregnant, or is nursing children.
18. The subject has received any of the treatments listed in Table 1 more recently than the indicated period before Screening.
19. The subject anticipates receiving any of the treatments listed in Table 1 during the current trial.

Table 1 Prohibited Medications, Supplements, and Other Substances	Period Before Screening
Anti-amyloid agents (eg, tarenflurbil, tramiprosate)	3 months
Anti-amyloid antibodies (eg, bapineuzumab)	6 months
Anti-amyloid vaccine<br>(Subjects who received placebo in a vaccine trial may participate in this trial.)	ever
Regularly used (> 2 doses/week) anticholinergic medications of moderate potency or greater (eg benztropine, cyclobenzaprine, cyproheptadine, dicyclomine, diphenhydramine, promethazine, diphenoxylate with atropine, hydroxyzine, hyoscyamine, prochlorperazine, trihexyphenidyl, trimethobenzamide).<br>**Exception:** Daily use of anticholinergic medications for incontinence (eg, oxybutynin, tolterodine, darifenacin, solifenacin, trospium, fesoterodine), nasal spray for rhinorrhea (ipratropium) or inhalants for pulmonary disorders (eg, tiotropium) is acceptable if stable for at least one month before the Screening Visit.	4 weeks
Antidepressants that are monoamine oxidase (MAO) inhibitors or antidepressants with moderate or greater anticholinergic potency or cognitive side effects, including tricyclics (eg, amitriptyline, amoxapine, clomipramine, desipramine, imipramine, isocarboxazide, maprotiline, nortriptyline, phenelzine, protriptyline, tranylcypromine, trimipramine)<br>**Exception**: use of 50mg or less at night of nortriptyline or desipramine during the trial is acceptable.	4 weeks
Neuroleptics with moderate or greater anticholinergic potency (eg, chlorpromazine, fluphenazine, loxapine, perphenazine, thioridazine, thiothixene, trifluoperazine, clozapine)	4 weeks
Mood stabilizers and anticonvulsants (eg, lithium, valproic acid, phenytoin, levetiracetam, carbamazepine)<br>**Exception**: use of pregabalin and gabapentin for neuropathic pain is acceptable.	4 weeks
General anesthetics	3 months
Regularly used (> 2 doses/week) sedatives/benzodiazepines (eg, chlordiazepoxide, clonazepam, diazepam, flurazepam, meprobamate, triazolam).<br>**Exception:** Daily use of the following medications is acceptable if stable for at least one month before the Screening Visit: trazodone, mirtazapine, zaleplon ≤ 5 mg, zopiclone ≤ 7.5 mg, eszopiclone ≤ 3 mg, zolpidem ≤ 5 mg, or lorazepam ≤ 1.0 mg. For other medications in this category not specified here, please contact the Sponsor for guidance.	4 weeks
Regularly used (> 2 doses/week) narcotic analgesics (eg, codeine, morphine , hydromorphone, oxycodone, propoxyphene (Darvon) and its variations, & combination products that contain a narcotic)<br>**Exception:** short term use (<1 month) more than 2 doses/week is acceptable for temporary conditions	4 weeks
Anti-parkinsonian medications (eg, L-dopa, amantadine, bromocriptine, pergolide, selegiline, l-deprenyl/selegiline, rasagiline).<br>**Exception:** carbidopa/levodopa and dopamine agonists are allowed for treating restless legs syndrome. .	3 months
Stimulant medications (eg, amphetamine, methylphenidate, atomoxetine, modafinil)	4 weeks
Corticosteroids: oral, intravenous, or intramuscular use<br>**Exceptions:** Use of the following is acceptable: low dose oral treatment with the equivalent of 10 mg prednisone or less; short-term (<3 weeks) oral treatment with the equivalent of 60 mg prednisone or less; local injections into joints or bursae; topical, inhaled or nasal use	4 weeks

CYP3A4 inducers (strong) including: rifampicin and St. John's Wort, phenytoin, carbamazepine<br>**Exceptions:** Use of the following is acceptable: topical use; short-term (<2 weeks) oral treatment during the trial; use of oral St. John's Wort <300 mg three times a day during the trial, regardless of duration.	4 weeks
Drugs known to cause ocular changes or damage (eg, chloroquine, hydroxychloroquine [sometimes used for arthritis], many anti-malarial treatments, ethambutol for tuberculosis, amiodarone for ventricular arrhythmias, and tamoxifen for breast cancer)	4 weeks
Digoxin	4 weeks
**Methotrexate**	4 weeks

NOTE: This is not a complete list of excluded medications. Contact the Sponsor if there is a question about a specific medication.

20. The subject has a known allergy or sensitivity to the excipients in the investigational product(s).
21. The subject has any clinically significant condition or situation, other than the condition being studied that, in the opinion of the investigator, would interfere with the trial evaluations, required procedures or optimal participation in the trial.
22. The subject has used any investigational drugs within the 30 days or 5 half-lives, whichever is longer, immediately before Screening or has participated in studies involving repeated cognitive testing within 30 days before screening. Observational studies, such as those involving annual cognitive assessments and/or neuroimaging (including investigational PET ligands), are allowed if approved by Sponsor.
23. The subject has a history of a hypersensitivity reaction to more than three drugs.
24. The subject has a history of erythroderma (exfoliative dermatitis), DRESS syndrome (Drug Reaction with Eosinophilia and Systemic Symptoms), Stevens-Johnson Syndrome or toxic epidermal necrolysis.
25. The subject has tested positive for HIV.
26. The subject has a close family member (including the caregiver, the spouse, or any children) who is among the personnel of the investigational or sponsor staff directly involved with this trial.

Additional Exclusion Criteria for Safety Cohort

27. The subject has a history of an ongoing medical condition that has been poorly controlled within **6 months** of the Screening Visit (such as, but not limited to, hypotension, diabetes, hypertension, cerebrovascular disease, thyroid disease, endocrine disturbance, congestive heart failure, cardiac or gastrointestinal disease, dialysis, or abnormal renal function with estimated creatinine clearance < 30 mL/min),) other than the condition being studied such that, in the judgment of the investigator, a subject's participation in the trial would pose a significant medical risk.

28. The subject has history of congestive heart failure (moderate or greater severity), myocardial infarction, heart surgery, syncope, bradycardia (resting heart rate persistently < 50 beats/minute over the past 12 months), or clinically significant hypotension within one year immediately before Screening.
29. The subject has trans-illumination defects of the iris, or irregularly shaped pupil.

Additional Exclusion Criteria for the CSF Substudy

30. The subject has any of the following conditions:

- increased intracranial pressure (ICP),
- skin infection at lumbar puncture (LP) site,
- bleeding diathesis (eg, thrombocytopenia), or.
- is taking anticoagulant therapy (eg, warfarin, dabigatran, apixaban, or other blood factor or thrombin inhibitor). Note: Use of anti-platelet therapy (eg, aspirin) is permitted if deemed appropriate in the investigator's judgment.

Additional Exclusion Criteria for the Amyloid PET Substudy

31. The subject has been exposed or will be exposed to ionizing radiation in other research studies and participation in this substudy would result in an annual exposure to the subject of > 15 mSv. Note: radiation exposure from routine medical procedures or other non-research activities is not considered part of the criteria for the 15 mSv limit.
32. The subject has had any surgical or medical condition which might significantly alter the distribution, metabolism, or excretion of [^{18}F]Flutemetamol.
33. The subject is unwilling or has a contraindication to undergo PET scanning including but not limited to claustrophobia, excessive weight or girth.

7.3.3 Subject Discontinuation Criteria

A subject may discontinue from the clinical trial at any time for any reason. A subject **must** be discontinued from the trial if the subject or legal representative (such as a parent or legal guardian) withdraws consent.

The investigator or the Sponsor should stop trial medication in any case in which emerging effects are of unacceptable risk to the individual subject, or if unmanageable factors arise that may interfere significantly with the trial procedures and/or the interpretation of results.

Subjects who discontinue trial medication may nevertheless still continue to participate in the regularly scheduled activities, so that data may continue to be collected in an effort to adhere to the intent-to-treat principle.

A subject must discontinue trial medication for any of the following reasons:

1. The subject or legal representative withdraws consent;
2. Elevated ALT, AST, or T-BIL meeting any one of the following criteria:
   A. ALT or AST ≥ 8 x ULN;
   B. ALT or AST ≥ 5 x ULN for more than 2 weeks;
   C. ALT or AST ≥ 3 x ULN and T-BIL ≥ 2 x ULN at the same visit;
   D. ALT or AST ≥ 3 x ULN with the appearance of symptoms indicating hepatitis (eg, worsening fatigue, nausea, vomiting, right upper quadrant pain or tenderness, fever, rash, or eosinophilia).

   Exception: if elevations are determined to be due to some other medical condition, subject may resume trial medication with Sponsor approval;
3. An imaging abnormality consistent with macrohemorrhage appears (see details in **Section 7.1**);
4. QTc prolongation (defined as QTc interval > 500 ms or QTc change from baseline > 60 ms, based on the average of three measurements using the Fridericia formula for correction).

   Exception: if QTc change from baseline > 60 ms is determined to be due to some other medical condition, or if subject has a new onset of bundle branch block, subject may continue treatment with Sponsor approval;
5. The subject develops a form of dementia that is not Alzheimer's disease, including but not limited to, dementia due to HIV infection, head trauma, vascular disease, Parkinson's disease, frontotemporal dementia, or Huntington's disease, as determined by the investigator;
6. The subject develops a severe rash. For the purpose of this program, a "**severe rash**" is defined as one of the following:
   - A vesicular rash (ie, one with blistering lesions) that is not clearly caused by herpes simplex virus or contact allergy such as poison ivy AND has EITHER a) extensive body surface area (BSA) involvement OR b) involves oral/mucosal surfaces
   - Stevens-Johnson Syndrome, erythroderma, or toxic epidermal necrolysis
   - DRESS syndrome
7. The subject develops an uncontrolled clinically significant rash defined as follows:
   - A clinically significant rash (see **Section 7.7.2.2.3**) that is not controlled by topical medications or oral medications such as antihistamines (detailed in the Rash Guidance Document), and
   - A clinically significant rash that causes intolerable discomfort for the subject.

8. The subject's trial partner is no longer willing or able to participate in the study and a suitable replacement trial partner cannot be found in a reasonable period of time;

Subjects who stop trial medication but are willing to continue in the trial should continue to be evaluated in accordance with the Trial Flow Chart. Subjects who are unwilling to continue should proceed to a termination visit. At a minimum, the following information should be collected when a subject discontinues:

1. The reason the subject discontinued;
2. The date of the last dose of test products from the trial;
3. The date of the last assessment and/or contact. A follow-up contact (telephone or visit) will be arranged as appropriate;
4. (Serious) Adverse events;
5. Compliance with the test product administration as specified in this protocol;
6. Final Assessments:
7. Every effort should be made to ensure that all procedures and evaluations scheduled for the final trial visit are performed (**Section 2.2**, Trial Flow Chart)
8. Retrieve all investigative products and test articles from the subject.

7.3.4 Replacement of Subjects

A subject who discontinues from the trial will not be replaced.

7.4 Treatments

7.4.1 Trial Treatments

7.4.1.1 Treatments Administered

One of the following treatments will be administered to each randomized subject in the Safety Cohort for the Double-Blind Treatment Period:

- MK-8931 12 mg tablets QD orally once per day;
- MK-8931 40 mg tablets QD orally once per day;
- MK-8931 60 mg tablets QD orally once per day*;
- Placebo tablets QD orally once per day.

*When the 60 mg dose is dropped following the first formal interim safety analysis, subjects will be switched to the 40 mg arm.

Absent a decision from the eDMC to drop either the 12 mg or 40 mg dose pursuant to an unexpected safety finding, one of the following treatments will be administered to each randomized subject in the Main Cohort for the remaining Double-Blind Treatment Period:

- MK-8931 12 mg tablets QD orally once per day;
- MK-8931 40 mg tablets QD orally once per day;
- Placebo tablets QD orally once per day.

Randomization and administration of the first dose of the trial medication will occur at the trial site on Study Day 1 (Visit 2) after all Baseline assessments have been completed. Subsequent dosing will be done once daily without regard to food by the subject or his/her trial partner/caregiver (ie, unsupervised at his/her home) at approximately the same time each day.

7.4.1.2 Method of Treatment Assignment, Randomization, and/or Stratification

Randomization will be implemented through the use of a central interactive voice response system (IVRS). Subjects in the Safety Cohort will be randomized in a 1:1:1:1 ratio to one of four treatment arms (three doses of MK-8931, or placebo). Subjects in the Main Cohort will be randomized in a 1:1:1 ratio to one of three treatment arms (12 and 40 mg MK-8931 or placebo).

Though not planned, it is possible that a second active dose arm (eg, 40 mg) could be dropped depending on eDMC review. After the first formal interim analysis, subjects already receiving a dose that is dropped will remain in the trial and will be re-assigned to take the higher MK-8931 dose remaining in the trial. As noted in the Synopsis and **Section 5.5**, it is possible that one dose can be dropped for safety/tolerability reasons during treatment of the Main Cohort. In the event that only one active dose remains while enrollment is still ongoing, then all subjects yet to be enrolled will be randomized in a 1:1 ratio to the remaining active dose or placebo and subjects in the dropped dose arm will be re-assigned to receive the remaining active dose.

Randomized treatment assignment will be stratified by geographic region (US/Canada, Europe/Australia/New Zealand, Japan, Rest of the World), severity of disease at Screening (mild AD: $20 < \text{MMSE} \leq 26$; moderate AD: $15 \leq \text{MMSE} \leq 20$) and AD treatment at Screening (use of AChEI or memantine; no use of AChEI or memantine). Both the percent of mild AD subjects and the percent of moderate AD subjects, respectively, will be no more than 60% of the total.

7.4.1.3 Selection and Timing of Dose for Each Subject

7.4.1.3.1 Selecting the Dose for Each Subject

The rationale for the selection of doses to be used in this trial is presented in Section 5.3.

7.4.1.3.2 Determining the Timing of Dose Administration for Each Subject

The trial medication should be administered by the subject, by the subject's trial partner, or by a caregiver. Each subject should take one tablet at the same time every day. The tablet comprising one dose of trial medication for each of the treatment groups is summarized below:

- Placebo: placebo (1 Tablet)
- MK-8931 12 mg: MK-8931 12 mg (1 Tablet)
- MK-8931 40 mg: MK-8931 40 mg (1 Tablet)
- MK-8931 60 mg: MK-8931 60 mg (1 Tablet) (Safety Cohort only)

The first dose of trial medication must be taken at the site on Study Day 1 (Visit 2) under supervision and after all Baseline assessments have been completed. **NOTE: If trial medication is not available on-site for the baseline/randomization visit (Study Day 1) the visit must be postponed until trial medication is available.**

The date and time that the trial medication is administered should be recorded by site personnel.

If a subject misses a dose, the subject may take the dose later in the day and should continue with the regular dosing schedule by taking the next dose at the usual time the next day. Any changes in dosing schedule should be noted by the subject or subject's trial partner/caregiver and recorded by the site at the next visit. Subjects should not take more than one dose on the same calendar day.

With the exceptions of a) those subjects originally randomized to a dose that is discontinued after the first formal safety interim analysis or b) any subjects randomized to a dose that is dropped for reasons of safety or tolerability, there will be no adjustments to the dose of any subject in the trial.

7.4.1.4 Blinding Trial Treatments

A double-blind technique will be used: all doses of MK-8931 and the matching placebo will be identical in appearance and will be packaged identically so that the treatment blind is maintained. The placebo tablet is similar to the investigational product with regard to appearance, weight, smell and taste. Neither the subject nor the investigational staff (sponsor, investigator, and evaluators) will know which treatment the subject is receiving.

See **Section 7.7.2.5.3** for a description of the method of unblinding a subject during the trial, should such action be warranted.

7.4.1.5 Investigational Medicinal Products

The investigator has the responsibility for taking all steps to maintain appropriate records and to ensure appropriate supply, handling, storage, distribution, and usage of these materials in accordance with the protocol and any applicable laws and regulations.

7.4.1.5.1 Identity of Investigational Medicinal Products

The trial medication will be provided as a tablet formulation to support all subjects (from all cohorts) in the trial. Please see the Investigator's Brochure for a full description of the investigational medicinal product.

Placebo tablets will be identically matched in appearance to the active trial medication. The trial will be a double blind trial: each of the active and placebo tablets will have the same look and feel to maintain the blind.

7.4.1.5.2 Source

The sponsor will provide trial medication as follows: MK-8931 12 mg tablets; MK-8931 40 mg tablets; MK-8931 60 mg tablets; matching placebo tablets.

7.4.1.5.3 Labeling

MK-8931/placebo blister pack labels should include the following information and comply with the regulatory requirements appropriate for clinical site: Dosing directions will state, "Take 1 tablet once a day".

7.4.1.5.4 Packaging

MK-8931 12, 40 and 60 mg and matching placebo tablets will be packaged in blister packs.

7.4.1.5.5 Storage

Trial treatment supplies must be stored in a secure, limited-access location under the storage conditions specified on the supply label. Site storage conditions should be monitored by the site personnel for adherence to label specifications and reviewed during site visits.

7.4.1.5.6 Dispensing

The investigator or qualified designee(s) will dispense trial treatments at the designated site(s) to subjects who have provided written informed consent and have met the entry criteria. Clinical supplies may not be used for any purpose other than that which is stated in this protocol.

See the Trial Flow Chart in Section 2.2 for a schedule of when clinical supplies are to be dispensed to the subjects.

In order to align with regional standard medical practice, trial sites in Japan are allowed to dispense subjects' treatment kits between drug dispensing visits identified in the protocol. Study medication will continue to be dispensed using the Interactive Voice Response system, however, study sites may increase the frequency with which study medication is dispensed to study participants in order to follow institutional regulations and/or local standard of care. All trial sites using partial dispensing options must follow the Merck guidance provided in "Procedure for Investigational Medicinal Product (IMP) Management" document.

7.4.1.5.7 Replacement of Investigational Product

Replacement of trial medication will be performed only in limited cases (eg, lost, broken, or spilled treatment bottles). In the event that replacement units are needed, the IVRS help desk should be contacted. The IVRS help desk will be responsible for obtaining permission to release replacement units from the sponsor.

7.4.1.5.8 Investigational Medicinal Product Accountability

Accurate and current accounting of the dispensing and return of investigational products will be maintained on an ongoing basis by a member of the trial site staff:

- Investigational medicinal products dispensed to each subject will be recorded in the trial-specific Subject IMP Accountability Log (or equivalent document approved by the sponsor).

The Subject IMP Accountability Log will be verified by the sponsor's trial monitor. The original Subject IMP Accountability Log will be approved by the investigator and retained at the trial site and a copy supplied to the sponsor when the trial is complete.

Each subject will be instructed by the investigator or designee to return all unused and partially used test articles to the site at all protocol-specified visits.

The sponsor’s trial monitor will instruct the site on the return of all investigational products supplies. Inventory records must be readily available for inspection by the trial monitor and/or auditor, and open to government inspection at any time.

7.4.2 Non-Trial Treatments

Subjects who are provided non-trial treatments, such as day care, may continue these through the trial. The frequency should not change unless medically indicated. Day care attendance on the day before clinic visits and cognitive testing should remain stable through the protocol. For example, a subject who attends day care the day prior to baseline testing should also attend day care on the day before all subsequent visits that include ADAS-Cog and ADCS-ADL testing.

7.4.2.1 Prior and Concomitant Medications

7.4.2.1.1 Medications, Supplements, and Other Substances Prohibited Prior to Screening and During the Trial

The subject must not take the treatments listed in **Table 1** prior to Screening and during the trial after Screening.

During the trial, initiation of treatment with medications known to be associated[21,22] with substantial increased risk of Stevens-Johnson Syndrome and toxic epidermal necrolysis should be avoided when possible. Subjects who have been safely treated with at least one routine course of treatment with these medications in the past are exempted from this requirement. Examples of such medications are included below. The sponsor should be consulted for questions about specific medications.

- trimethoprim-sulfamethoxazole, azithromycin, allopurinol, phenobarbital, oxicam NSAIDS (eg, celecoxib, valdecoxib, meloxicam) carbamazepine, phenytoin, valproic acid, nevirapine, lamotrigine, and chlormezanone

7.4.2.1.2 Concomitant Medications, Supplements, and Other Substances Allowed During the Trial

Medications, supplements, and other substances allowed during the trial include, but are not limited to, those listed in **Table 2**. Medication dose for non-AD treatments should be stable for at least the one month prior to screening and should not be changed during the trial unless medically necessary to ensure subject safety.

Requirements for AD medications are listed in the **Subject Inclusion Criteria Number 7** (**Section 7.3.1**). Acetylcholinesterase inhibitors and memantine should not be initiated during the trial unless medically necessary to ensure subject safety as this could impact the primary outcome measures; nevertheless, subjects who do so may continue in the trial.

Stability criteria for other allowed medications specified in the entry criteria (see Table 1) should be met. If changes are made during the screening period, subjects should continue with screening after the specified stability period (e.g., one month).

Note that the use of any concomitant medication must relate to the documented medical history, prophylaxis, or an adverse event of the subject.

Table 2 Medications, Supplements, and Other Substances Allowed During the Trial

Acetylcholinesterase inhibitors (eg, donepezil, tacrine, rivastigmine, galantamine)
Memantine
Huperzine A
Vitamin E
Herbal supplements from *Ginkgo biloba*, ginseng, *Huperzia serrata* (Qian Ceng Ta)
Medical foods/supplement (eg, Axona®, Souvenaid®)
Estrogens and estrogen-like compounds
Antihypertensives
Nonsteroidal anti-inflammatory drugs (NSAIDs)
Cycloxygenase 2 inhibitors
**Neuroleptics:** asenapine, aripiprazole, olanzapine, quetiapine, risperidone, ziprasidone
**Analgesics/Narcotics:** Use of ≤ 2 doses/week or short-term use (<1 month) of more than 2 doses/week for temporary conditions is acceptable (eg, codeine, morphine , hydromorphone, oxycodone, propoxyphene (Darvon) and its variations, & combination products that contain a narcotic).
**Sedative/benzodiazepines:** Use of the following medications is acceptable if stable for at least one month before the Screening Visit: trazodone, mirtazapine, zaleplon ≤ 5 mg, zopiclone ≤ 7.5 mg, eszopiclone ≤ 3 mg, zolpidem ≤ 5 mg, or lorazepam ≤ 1.0 mg. For other medications in this category not specified here, please contact the Sponsor for guidance.
**Antidepressants:** bupropion, citalopram (40 mg or less), escitalopram, fluoxetine, mirtazapine, paroxetine, sertraline, venlafaxine. Use of 50mg or less at night of nortriptyline or desipramine during the trial is acceptable.
**Carbidopa/levodopa and dopamine agonists** are allowed for treating restless legs syndrome.
**Pregabalin and gabapentin:** Treatment for neuropathic pain
**Anticholinergic medications**: Daily use of anticholinergic medications for incontinence (eg, oxybutynin, tolterodine, darifenacin, solifenacin, trospium, fesoterodine), nasal spray for rhinorrhea (ipratropium) or inhalants for pulmonary disorders (eg, tiotropium) is acceptable if stable for at least one month before the Screening Visit.
**Rifampicin and St. John's Wort:** Topical use, short-term (<2 weeks) oral treatment during the trial, use of oral St. John's Wort <300 mg three times a day during the trial, regardless of duration
**Corticosteroids:** Low dose oral treatment with the equivalent of 10 mg prednisone or less, short-term (<3 weeks) oral treatment with the equivalent of 60 mg prednisone or less, if needed for management of rash, local injections into joints or bursae, topical use, inhaled or nasal use
Selective H1 blockers, selective H2 blockers, and topical anti-pruritic treatments for treatment of rash during the trial, as specified in the Rash Guidance Document

7.4.3 Procedures for Monitoring Subject Compliance With Administration of Trial Treatments

At all protocol-specified visits, the investigator or qualified designee is to record whether treatment had been taken per protocol in the preceding interval.

7.5 Trial Schedule

The visit-by-visit schedule of trial activities is provided in the Trial Flow Chart in **Section 2.2**.

The timing of each subject visit is relative to Study Day 1 of that subject, with Study Day 1 defined as the date of randomization (which should also be the date of the first administration of trial medication) (**Section 7.4.1.1**).

All visits should be performed within the windows specified in **Section 2.2**, the Trial Flow Chart. Every attempt should be made to have each subject attend each visit as scheduled. However, if a subject is unable to attend a visit within the specified windows, the visit should be scheduled as closely as possible to these windows. A subject should not miss a protocol-specified visit due to scheduling difficulties.

The trial partner is expected to accompany the subject to all trial visits. At the discretion of the investigator, exceptions are acceptable, such as visits where no clinical measures are scheduled for administration to the trial partner (e.g., Visit 3 or 4). However, in these instances, the trial partner should be contacted by phone to complete AE, concomitant medication, and trial medication compliance review. Other exceptions to trial partner attendance include visits scheduled to conduct procedures such as MRI and ophthalmology evaluations. For additional details, the Sponsor may be consulted.

7.6 Trial Procedures

The Trial Flow Chart in **Section 2.2** summarizes the trial procedures to be performed at each visit. Individual trial procedures are described below.

The order in which evaluations are completed is at the discretion of the investigator in order to best accommodate the needs of the subject/caregiver as well as the logistical considerations of the coordinator/staff.

In order to minimize variability of evaluations, it is preferred that the same individuals perform the same types of evaluations for all subjects at each trial site. In addition, blood and CSF samples for a visit should NOT be collected prior to the testing of the clinical outcomes.

1. Explain Trial and Obtain Written Informed Consent

   The investigator or qualified designee will explain the trial to the subject, answer all of his/her questions, and obtain written informed consent before performing any trial-related procedure. A copy of the informed consent will be given to the subject (see **Section 9.1.2** for further description of the Informed Consent).

   Given the trial population and the duration of the trial, it is possible that a subject's cognition may decline to a point where they no longer have capacity to provide informed consent. If this occurs, the site should obtain consent to continue in the trial from the subject's legally acceptable representative and in accordance with local standards and requirements. The subject's assent to continue should also be obtained.

2. Explain Trial and Obtain Written Informed Consent for Substudies.

   The investigator or qualified designee will explain the collection of CSF samples and amyloid plaque imaging to the subject, answer all of his/her questions, and obtain written informed consent before performing any of these procedures. A copy of the informed consent will be given to the subject.

Confidential

3. Explain Pharmacogenetic Substudy and Obtain Written Informed Consent for Pharmacogenetic Testing

   The investigator or qualified designee will explain the pharmacogenetic testing to the subject, answer all of his/her questions, and obtain written informed consent before performing any procedure related to pharmacogenetic testing (except APOE and HLA genotyping, which is explained as part of the main trial informed consent). A copy of the informed consent will be given to the subject.

4. Issue or Collect Subject Identification Card

   The investigator or qualified designee will provide the subject with a Subject Identification Card after the subject provides written informed consent. The investigator or qualified designee will retrieve the card from the subject at the last contact (see **Section 9.1.3** for further description of the Subject Identification Card).

5. Obtain Demographic Data

   Record demographic data (eg, sex, race, ethnicity, age, years of education).

6. Obtain Medical History

   A medical history will be obtained by the investigator or qualified designee. A subject's medical history should include the diagnosis date and any therapies taken for AD (see **Section 7.4.2.1**), information on family history and personal history. All relevant findings should be recorded in the medical history.

7. Review Prior Medications

   Review of appropriate prior medications, including the necessary washout times, with the subject. For non-AD prior medications, a record of prior medication taken by the subject within three months before starting the trial will be obtained. For AD medications, a record of prior medication taken by the subject up to one year before starting the trial will be obtained.

8. Record Concomitant Medications

   A record of concomitant medication taken by the subject during the trial is to be obtained.

9. Record (Serious) Adverse Events

   See **Section 7.7.2.4**, for instructions on the assessment and reporting of (Serious) Adverse Events and **Section 7.7.2.5** for instructions on the reporting of (Serious) Adverse Events to the sponsor.

10. Vital Signs

    The following vital signs will be measured and recorded: pulse (beats/minute), BP (mm Hg), temperature (°C/°F), and respiratory rate (breaths per minute). Blood pressure should be measured in the sitting position.

11. Body Weight (kg/lbs) and Height (cm/in)

    Height and body weight data will be collected and recorded. Body weight data will be collected without shoes and with heavy clothing removed. Body weight should be performed on the same scale for the same individual. Measurements should be recorded to the nearest kilogram/pounds and centimeter/inches.

12. Physical & Neurological Examinations

    A physical examination including a standard neurological examination will be performed. If the subject is discontinued for any reason during the treatment phase, every attempt should be made to perform a final physical examination.

13. Modified Hachinski Ischemia Score

    The subject's modified Hachinski Ischemia Score[9] will be determined and recorded by the principal investigator or trained designee according to the instructions in the **Manual of Assessments**.

14. 15-item Geriatric Depression Scale

    The 15-item Geriatric Depression Scale will be administered to the subject in paper form and scored and recorded by the principal investigator or trained designee according to the instructions in the **Manual of Assessments**.

15. Mini-Mental State Examination (MMSE)

    The MMSE will be administered to the subject in paper form and scored and recorded by the principal investigator or trained designee according to the instructions in the **Manual of Assessments**. The MMSE is very important for characterizing the complete patient population at baseline, so it is required for every subject who has signed the informed consent form including subjects who have screen-failed.

16. Columbia Suicide Severity Rating Scale (C-SSRS)

    The C-SSRS will be administered to the subject and scored and recorded by the principal investigator or trained designee according to the instructions in the **Manual of Assessments**.

The C-SSRS provides a detailed assessment of suicidal ideation and behaviors. The paper version of the C-SSRS will be completed at each visit as indicated in the Trial Flow Chart (and unscheduled visits as clinically indicated). At baseline, information will be collected regarding the subject's lifetime history of suicidal ideation and behavior, while information collected at subsequent visit will be based on the time interval since the previous visit. In addition, subjects who at any time during this study spontaneously report AEs of suicidal ideation or behavior with intent (with or without a plan), either as outpatient or during visit interviews, must be assessed by the Investigator and referred for further mental health evaluation as clinically indicated. Subjects who report suicidal ideation with intent, with or without a plan or method (ie, a positive response to Items 4 or 5 in the assessment of suicidal ideation on the C-SSRS) or suicidal behavior must be evaluated that day by a psychiatrist or other trained mental health professional who is a licensed psychologist, social worker or nurse practitioner (or comparable professional qualification in countries outside the United States). Only subjects whose suicidal ideation is passive, who expressly deny any intent to act, and who, after evaluation, are not judged to be at serious risk for self-harm during the course of the trial may continue with trial treatment; others must be discontinued from trial treatment and receive appropriate clinical follow-up care to assure their safety. After appropriate follow-up care, if the investigator judges that the subject can safely resume trial treatment, re-dosing can be considered with Sponsor approval. As part of site validation, sites are to indicate

which health care professionals are to be responsible for acute care on-site and to specify referral center(s) to be used for further evaluation. All reports of suicidal ideation or behavior must be recorded as an Event of Clinical Interest (ECI).

17. Neuropsychiatric Inventory (NPI)

    The NPI will be administered to the subject's trial partner/caregiver in paper form and scored and recorded by the principal investigator or trained designee according to the instructions in the **Manual of Assessments**.

18. Review Inclusion/Exclusion Criteria

    The inclusion and exclusion criteria will be reviewed by the investigator or qualified designee to ensure that the subject qualifies for the trial.

    NOTE: in addition to the Inclusion/Exclusion criteria, the investigator must have the following documents from relevant vendors prior to randomization:

    a) MRI approval document, certifying that the MRI is acceptable;

    b) diagnostic verification* certification certifying agreement with diagnosis of AD dementia.

    **Please refer to the Manual of Assessments for qualifications required of the Qualified Designee when the Qualified Designee is completing the Diagnostic Verification for study entry.*

19. Alzheimer's Disease Assessment Scale Cognitive Subscale (ADAS-Cog)

    The ADAS-Cog will be administered to the subject in paper form and scored and recorded by the principal investigator or trained designee according to the instructions in the **Manual of Assessments**.

20. Alzheimer's Disease Cooperative Study Activities of Daily Living Inventory (ADCS-ADL)

    The ADCS-ADL will be administered to the subject's trial partner/caregiver in paper form and scored and recorded by the principal investigator or trained designee according to the instructions in the **Manual of Assessments**.

21. 12-Lead Electrocardiogram (ECG)

    A 12-Lead Electrocardiogram will be performed according to the instructions in a separate **ECG Instruction Manual.**

    Note: Triplicate measurements of ECG will be required at the Screening Visit and if there is an observation of QTc prolongation at any post-Screening visit.

22. Skin ExaminationsTo monitor for hypopigmentation, skin examinations will be performed at the visits specified in **Section 2.2**, Trial Flow Chart. At the Screening and Week 26 visits, a dermatologist should perform a detailed examination of the subject's skin, including the head, neck, extremities, and torso, as well as the oral cavity. A full body exam should be performed if possible, however removal of all clothing (ie undergarments) will not be required if a subject refuses. All trial sites will make best efforts to arrange for a dermatologist to perform these scheduled assessments. Where this is operationally infeasible (eg, due to excessive travel distance to a dermatologist), the site should contact the sponsor who will determine on a case by case basis if all reasonable efforts were made to identify a dermatologist. In exceptional cases, and only with Sponsor approval, a site physician may perform the skin assessments at these visits. The site should document all reasonable efforts made to identify a dermatologist. In the case that a site's dermatologist has not yet completed all site ready activities (e.g., regulatory documentation, training), the site physician should perform the Full Body Skin Examinations at Visits 1 and 6 after notifying the Sponsor.

    At Weeks 13, 52 and 78, a site physician will perform a directed examination of the subject's skin. In addition, sites will instruct subjects to perform a self-examination of their skin between clinic visits (with the assistance of their trial partner, if needed). Subjects should report any abnormal loss of skin pigment to the site. The site physician's examination will focus on exposed skin and any areas of pigment loss noted by the subject or trial partner. If a clinically significant area of hypopigmentation is observed, the site should refer the subject to a dermatologist for further follow-up.

    At each scheduled assessment, the following information should be recorded: whether significant sites of skin hypo- or depigmentation were observed, assessor (dermatologist or site physician), and any adverse events.

    Based on the local standard of care, the investigator should refer subjects to a dermatologist or medical expert if: 1) a clinically significant area of skin develops hypopigmentation compared to baseline; or 2) if needed based on their clinical judgment. The following criteria are provided to guide the investigator but are not meant to be inclusive or to require referral in all cases.

    1) A clinically significant area of hypopigmentation may include:

        a) greater than 3x3 cm

        b) a smaller area (eg, 1x1 cm) on the face or hands

        c) a speckled pattern involving a larger amount of body surface area or

        d) a smaller area (eg, 1x1 cm) of complete pigment loss.

Hypopigmentation for criterion 1 is defined as a decrease in skin color relative to the surrounding skin and its prior baseline appearance.

2) Clinical judgment criteria include:

a) In the investigator's judgment, the hypopigmentation requires evaluation by an expert (eg, small lesions on the face), or

b) The subject or caregiver is distressed by the appearance of the skin lesion or hypo/depigmentation

Dermatologists will evaluate and treat any lesions based on local standard of care. No special procedures are required per protocol.

23. Laboratory Tests

Laboratory tests for hematology, blood chemistry, and urinalysis are specified in Table 3. Blood samples for laboratory tests are to be taken **prior** to investigational product(s) administration at visits where trial medication is administered on site.

Table 3 Laboratory Tests

Hematology	Chemistry	Urinalysis
Basophils	Albumin	Blood
Eosinophils	Alkaline phosphatase	Glucose
Hematocrit	ALT (SGPT)	Ketones
Hemoglobin	AST (SGOT)	Microscopic exam
Lymphocytes	Bicarbonate	pH
Monocytes	Blood urea nitrogen (BUN)	Protein
Neutrophils	Calcium	Specific gravity
Partial thromboplastin time[a,b]	Chloride	
Platelets	Cholesterol	
Prothrombin time[a,b]	Creatinine	
RBC	Folate[a]	
WBC	FSH[a]	
	Glucose	
	Homocysteine and methylmelonic acid[a,c]	
	Inorganic phosphorus	
	LDH	
	Potassium	
	Treponemal EIA reflex to FTA-ABS or other test for syphilis [a,c]	
	Serum pregnancy test (βhCG)[a,d]	
	Sodium	
	T4[a,c]	
	Total Bilirubin	
	Total protein	
	TSH[a]<br>Vitamin B12[a]	

EIA=enzyme immunoassay; FTA-ABS=fluorescent treponemal antibody absorption

a For Screening Visit only.

b For the CSF substudy only to exclude bleeding diathesis.

c Will be measured according to the **Laboratory Manual**.

d Each female subject must have a serum pregnancy test (βhCG) at Screening if cessation of menses was < 12 months before Screening and ≥ 6 months and there are no other indications that the subject is not of childbearing potential as defined in the inclusion criterion in **Section 7.3.1**. The serum pregnancy test must be negative before the first administration of trial medication.

24. Childbearing Potential Assessment

    Assess whether a female subject is of childbearing potential with a serum FSH test if serum FSH levels are required for the determination (see **Section 7.3.1**).

25. Pregnancy Assessment

    Assess whether a female subject is pregnant with a serum βhCG test if it is required (see **Section 7.3.1**).

26. Structural MRI

    As indicated in **Section 2.2**, Trial Flow Chart, subjects will have 1.5 Tesla or 3.0 Tesla MRI scans according to a separate **MRI Procedure Manual and Imaging Charter**. For a given subject all scans should be acquired at the same field strength for the duration of the trial.

    To evaluate the eligibility criteria, each subject must have either: an MRI scan performed during the Screening Period or a previous MRI performed within 12 months before the Screening visit that is available for review by the central MRI reading vendor. A head CT scan may be accepted instead of MRI on a case-by-case basis, as approved by the Sponsor (e.g., when MRI is contraindicated for the subject) and if so the CT scan must also be available for central vendor review. If a new MRI needs to be performed for screening, it is preferable to be scheduled to occur after the subject has met all the inclusion and exclusion criteria that do not require an MRI. The radiologist review by the imaging vendor of the MRI scan must be available before randomization to evaluate the inclusion/exclusion criteria.

    All MRIs/CTs will be read by a central reader and the results reported to the site. The central reader must verify in writing to the site that the screening MRI (or CT) scan is acceptable prior to randomization. In addition, an MRI may be performed at any visit for safety monitoring if clinically indicated as determined by the investigator (e.g., in follow-up to an AE). To limit variability and operator errors, all MRI sequences should be run at each scan, even if performed for safety monitoring. Site radiologists can read MRI scans for safety monitoring and should contact the central reader for immediate consultation in the case of a safety concern, such as a possible vasogenic edema.

    All subjects who have a new MRI scan performed during the screening period will also have an End of Treatment MRI scan (Week 78 or Early Termination Visit) for purposes of the volumetric analyses. A subject's End of Treatment MRI should be scheduled within four weeks before the End of Treatment clinical visit and before trial medication is discontinued. For volumetric MRI data analyses, the Screening MRI will be used for the Baseline assessment. Note that subjects whose screening assessment was based on an MRI scan performed prior to the study or based on a CT scan will not undergo an end of treatment MRI scan and will not contribute to the biomarker analyses.

27. Clinical Dementia Rating (CDR)

    The CDR will be administered to both the subject and the subject's trial partner/caregiver in paper form, and the CDR Sum of Boxes (CDR-SB) will be scored and recorded by the principal investigator or trained designee according to the instructions in the **Manual of Assessments**.

28. Modified Resource Utilization in Dementia (RUD) Lite Questionnaire

    A modified **RUD Lite** Questionnaire will be administered to the subject's trial partner/caregiver in paper form and recorded by the principal investigator or trained designee according to the instructions in the **Manual of Assessments**.

29. Health Economic Assessment (HEA)

    The **HEA** will be administered to the subject's trial partner/caregiver in paper form and recorded by the principal investigator or trained designee according to the instructions in the **Manual of Assessments**.

30. EuroQol Five Dimension Questionnaire (EQ-5D)

    The EQ-5D will be administered to the subject's trial partner/caregiver in paper form and recorded by the principal investigator or trained designee according to the instructions in the **Manual of Assessments**.

31. Blood Sample for APOE and HLA Genotyping

    To obtain sufficient DNA for APOE and HLA genotyping, blood samples will be drawn at the specified time point indicated in **Section 2.2**, Trial Flow Chart, into the appropriate tubes provided by the sponsor (see the **Laboratory Manual** for sample acquisition, shipping and labeling instructions).

32. Pharmacokinetic/Pharmacodynamic Blood Samples

    Blood samples for PK/PD analyses should be collected, processed, stored, and packaged according to the instructions in the **Laboratory Manual**.

    Record the following information for each PK/PD blood sample collected:

    - Date and time of each PK/PD blood sample;
    - Date and time of the last two doses of trial medication before each PK/PD blood sample.

    Careful attention to the collection, handling, and storage of the PK/PD blood samples is essential to reduce the risk of hemolysis and PK/PD variability. Any deviations from the PK/PD blood collection schedule, such as a missing or breaking a sample, should be documented.

33. Cerebrospinal Fluid (CSF) Collection

At the specified time points indicated in **Section 2.2**, Study Flow Chart, at sites that are qualified and willing to perform a lumbar puncture, all subjects who have consented to provide CSF samples will have CSF collected by lumbar puncture. Lumbar puncture should NOT be performed immediately prior to testing of clinical outcomes (eg, ADAS-Cog). For the Screening Visit, CSF should be collected after inclusion and exclusion criteria have been met and the LP should occur between the Screening Visit and the Baseline Visit following an acceptable MRI or CT scan and before randomization. For End of Treatment and Early Termination visits, CSF collection should occur within 4 weeks of the clinical visit and before trial medication is discontinued.

CSF samples for PK/PD analyses should be collected, processed, stored, and packaged according to the instructions in the **Laboratory Manual**.

Record the following information for each PK/PD CSF sample collected:

Date and time of each PK/PD CSF sample;

Date and time of the last dose of trial medication before each PK/PD CSF sample.

Careful attention to the collection, handling, and storage of the PK/PD CSF samples is essential to reduce PK/PD variability. **POLYPROPYLENE tubes and liquid transfer devices will both be supplied by the Sponsor and MUST be used. Use of other tubes could substantially alter the CSF measures.** Any deviations from the PK/PD CSF collection schedule, such as a missing, broken, or damaged sample, should be documented.

The CSF samples obtained will be split into six aliquots. Two aliquots will be used for PK/PD analyses specified in this protocol, while four aliquoted CSF samples will be shipped and stored long term for future use.

Confidential

34. Amyloid Imaging by PET

At the specified time points indicated in Section 2.2, Trial Flow Chart, subjects at designated sites will have amyloid imaging by PET with [^{18}F]Flutemetamol tracer. Each subject dose per scan will contain up to 185 MBq (5 mCi) ± 10% at the time of administration and not more than 6 μg/mL flutemetamol plus related substances. The maximum administered dose volume is 10 mL with a maximum of 20 μg total flutemetamol. For further details on amyloid imaging by PET, please see Appendix 6, "The use of [18F]Flutemetamol, a Positron Emission Tomography (PET) tracer to non-invasively detect the presence or lack thereof of amyloid plaque in the brain of aMCI subjects," and the Investigator's Imaging Operations Manual for PET Imaging of the Brain (IIOM).

The subject's PET scan will be read by a central reader. A subject's initial PET scan for amyloid imaging should be scheduled to occur after inclusion and exclusion criteria have been met and between the Screening Visit and the Baseline Visit following an acceptable MRI scan and before randomization. To participate in the PET substudy, subjects must have had a new MRI at screening (i.e., subjects screened based on a CT scan or historical MRI would not be eligible for the substudy). For End of Treatment and Early Termination visits, PET scans should occur within 4 weeks of the clinical visit and before the trial treatment is discontinued.

35. Pharmacogenetic (PGt) Samples

Informed consent specific for PGt sampling, must be obtained prior to collection. To obtain sufficient DNA for pharmacogenetic studies, a single 8.5-mL blood sample will be drawn at the specified time point indicated in Section 2.2, Study Flow Chart, into the appropriate tubes provided by the sponsor (see the **Laboratory Manual** for sample acquisition, shipping, labeling instructions and additional information on PGt sample management).

36. Dispense Trial Medication

The investigator or qualified designee will dispense the subject's treatment kit (see Section 7.4.1.5.8) and instruct the subject and subject's trial partner/caregiver regarding dosing with trial medication (see Section 7.4.1.3.2).

37. Administration of Trial Medication on Site

The first dose of trial medication should be administered at the site under medical supervision on Study Day 1. The time and date of first dose of trial medication administration will be recorded.

On the day of the 26-Week Visit (Visit 6), subjects should not take their trial medication before going to the sites. Trial medication should be administered on site after the PK/PD blood sample is collected. The time and date of this trial medication administration will be recorded. Safety Cohort subjects who have completed this procedure correctly at the 2-Week Visit (Visit 3) do not need to perform this procedure again at Visit 6.

38. Medication Compliance/Drug Accountability Assessment

The investigator or qualified designee will account for trial medication as described in Section 7.4.1.5.8.

39. Visual Acuity Test[1]

Visual acuity test will be attempted under standardized conditions according to the MK-8931 BACE Program Manual of Ophthalmic Procedures. Use of the Early Treatment Diabetic Retinopathy Study (ETDRS) chart or equivalent (eg, Landolt ‘C’ ETDRS chart or Snellen chart) for the test should be attempted first. If cognitive impairment interferes with these tests, Teller Acuity Cards may be attempted to assess visual acuity during the trial. For some subjects, dementia severity may prevent accurate assessment of visual acuity.

40. Posterior Eye Exams [1]

41. Posterior eye exam with dilated funduscopy will be performed to assess retinal effects. These procedures will be performed according to the MK-8931 BACE Program Manual of Ophthalmic Procedures.

42. Fundus Photography [1]

Fundus photography will be performed at the posterior pole of each eye according to the MK-8931 BACE Program Manual of Ophthalmic Procedures. Fundus photographs will be read by a central Reading Center designated by the Sponsor.

43. Fundus Autofluorescence [1, 2]

Fundus autofluorescence will be measured with a Sponsor-approved instrument according to the MK-8931 BACE Program Manual of Ophthalmic Procedures.

Autofluorescence pattern changes with enlarged areas of hypo- and hyper-fluorescence will be explored as detailed in the MK-8931 BACE Program Manual of Ophthalmic Procedures.

Best efforts will be made to accommodate the FAF assessment. Where the FAF assessment is operationally infeasible sites can enroll subjects without the FAF assessment. FAF images will be read by a central Reading Center designated by the Sponsor.

44. Spectral-domain Optical Coherence Tomography (SD-OCT) [1]

SD-OCT images of the retina will be acquired according to the MK-8931 BACE Program Manual of Ophthalmic Procedures.

In order to achieve very high repeatability and reproducibility, only an SD-OCT system that is approved by the Sponsor will be used in the trial.

SD-OCT measurements including the thickness of RPE, outer nuclear layer and possibly other layers including the retinal nerve fiber layer will be detailed in the MK-8931 BACE Program Manual of Ophthalmic Procedures.

45. Confusion Assessment Method

The CAM is completed by the rater based on information provided by other sources (i.e. medical records, asking the subject questions). <u>It should be completed only if and when an adverse event of delirium is suspected</u> to ascertain the diagnosis of delirium.

[1] Following the approval of Amendment 13 routine ophthalmology examinations are no longer required and have been removed from the Trial Flow Chart. However, subjects who had a Visit 1 ophthalmology examination will have assessments for #39 through #44 (noted above) completed during the Week 78 Visit. If cognitive impairment interferes with the VA test, Teller Acuity Cards should be used to assess VA at ophthalmology sites that have this capability.

[2] FAF will continue to be performed at Week 78 Visits at sites who have this capability only for subjects who had a baseline assessment.

7.7 Assessments

7.7.1 Efficacy Assessments

7.7.1.1 Coprimary Efficacy Endpoints

The two Coprimary Efficacy Endpoints for the trial are the:

1. change-from-Baseline score in the ADAS-Cog at Week 78 and
2. change-from-Baseline score in the ADCS-ADL at Week 78.

7.7.1.2 Key Secondary Efficacy Endpoint

The Key Secondary Efficacy Endpoint is the change-from-Baseline score in the CDR-SB at Week 78.

7.7.1.3 Other Efficacy Endpoints

7.7.1.3.1 Other Secondary Efficacy Endpoints

Other Secondary Efficacy Endpoints are:

1. the change-from-Baseline in total hippocampal volume at Week 78;
2. the change-from-Baseline on CSF total tau concentration at Week 78;
3. the change-from-Baseline on cortical amyloid load using PET at Week 78 (assessed with specific uptake value ratio [SUVR] from composite cortical region of interest of [^{18}F]Flutemetamol binding);
4. responder status at Week 78 (see **Section 8.2.4** for the definition of a responder);
5. the change-from-Baseline in NPI score at Week 78;
6. the change-from-Baseline in MMSE score at Week 78.

7.7.1.3.2 Exploratory Efficacy Endpoints

Exploratory Endpoints include the:

Change-from-Baseline scores at 13, 26, 39, 52, and 65 weeks of treatment for the primary and secondary measurements (as applicable): ADAS-Cog, ADCS-ADL, CDR-SB, total hippocampal volume, NPI and MMSE. Additional exploratory pharmacodynamic endpoints are defined in **Section 7.7.4.1**.

7.7.2 Safety Monitoring and Assessments

7.7.2.1 Safety Endpoints

Safety Endpoints of Special Interest (Tier 1)

Safety parameters and AEs of special interest are: 1) microhemorrhage, superficial siderosis or macrohemorrhage; 2) vasogenic edema; 3) delirium; and 4) rash ECI (see **Section 7.7.2.2.3**). Some of these AEs are combined into composite endpoints for formal safety analyses (see **Table 11**).

Commonly Occurring Safety Endpoints (Tier 2)

Commonly Occurring Safety Endpoints include safety parameters and AEs not included in the Safety Endpoints of Special Interest, but observed to be “common.” For this trial, a safety parameter or AE is considered as “common” if it occurs in ≥ 1% of subjects in any one treatment group.

Descriptive Safety Endpoints (Tier 3)

Descriptive Safety Endpoints include all other safety parameters not analyzed as a Tier 1 or Tier 2 Safety Endpoint. These Tier 3 Safety Endpoints include AEs, laboratory assessments, ECG assessments, ophthalmological measures and vital signs.

7.7.2.2 Definition of Terms

7.7.2.2.1 Adverse Event

Per the International Conference on Harmonization (ICH), an adverse event (AE) is defined as any untoward medical occurrence in a patient or clinical investigation subject administered a pharmaceutical product and which does not necessarily have to have a causal relationship with this treatment. An AE can therefore be any unfavorable and unintended sign (including an abnormal laboratory finding, for example), symptom, or disease temporally associated with the use of a medicinal product, whether or not considered related to this medicinal product. Any worsening (ie, any clinically significant adverse change in frequency and/or intensity) of a preexisting condition that is temporally associated with the use of the Sponsor’s product, is also an adverse event.

Changes resulting from normal growth and development that do not vary significantly in frequency or severity from expected levels are not to be considered adverse events. Examples of this may include, but are not limited to, teething, typical crying in infants and children and onset of menses or menopause occurring at a physiologically appropriate time.

Progression of the condition under study is not considered an adverse event unless it is assessed as drug-related by the investigator, or is characterized by unusual or atypical decline for AD progression in the judgment of the investigator, or meets criteria for a serious adverse event (see Section 7.7.2.2.2).

Sponsor's product includes any pharmaceutical product, biological product, device, diagnostic agent or protocol-specified procedure, whether investigational (including placebo or active comparator medication) or marketed, manufactured by, licensed by, provided by or distributed by the Sponsor for human use.

Adverse events may occur during clinical trials, or as prescribed in clinical practice, from overdose (whether accidental or intentional), from abuse and from withdrawal.

7.7.2.2.2 Serious Adverse Event

Serious Adverse Event (SAE) is any untoward medical occurrence or effect that at any dose:

1. Results in death;
2. Is life-threatening;
3. Requires hospitalization or prolongation of existing inpatients' hospitalization;
4. Results in persistent or significant disability or incapacity;
5. Is a congenital anomaly or birth defect;
6. Is an Other Important Medical Event.

Note: In addition to the above criteria, adverse events meeting either of the below criteria, although not serious per ICH definition, are reportable to the Sponsor in the same timeframe as SAEs to meet certain local requirements. Therefore, these events are considered serious by the Sponsor for collection purposes.

7. Is a cancer;
8. Is associated with an overdose;

Refer to Table 4 for additional details regarding each of the above criteria.

Life-threatening in the definition of a serious adverse event refers to an event in which the subject was at risk of death at the time of event; it does not refer to an event which hypothetically might have caused death if it were more severe.

Medical judgment should be exercised in deciding whether an adverse event/reaction is serious in other situations. Important adverse events/ reactions that are not immediately life-threatening or do not result in death or hospitalization, but may jeopardize the subject or may require intervention to prevent one of the other outcomes listed in the definition above, should also be considered serious. These are considered “Other Important Medical Events”.

For the time period beginning when the consent form is signed until randomization, any serious adverse event, or follow up to a serious adverse event, including death due to any cause, that occurs to any subject must be reported within 24 hours to the Sponsor if it causes the subject to be excluded from the trial, or is the result of a protocol-specified intervention, including but not limited to washout or discontinuation of usual therapy, diet, placebo treatment or a procedure.

For the time period beginning at randomization through 14 days following cessation of treatment, any serious adverse event, or follow up to a serious adverse event, including death due to any cause, whether or not related to the Sponsor's product, must be reported within 24 hours to the Sponsor either by electronic media or paper. Electronic reporting procedures can be found in the EDC data entry guidelines. Paper reporting procedures can be found in the Investigator Trial File Binder (or equivalent).

Additionally, any serious adverse event, considered by an investigator who is a qualified physician to be related to the Sponsor's product that is brought to the attention of the investigator at any time outside of the time period specified in the previous paragraph also must be reported immediately to the Sponsor.

All subjects with serious adverse events must be followed up for outcome.

7.7.2.2.3 Events of Clinical Interest

An "Event of Clinical Interest" is a non-serious adverse event or occurrence that is designated to be of special interest and must be reported to the sponsor as though it were a serious adverse event – as described in **Section 7.7.2.5.1**.

For the time period beginning when the consent form is signed until randomization, any ECI, or follow up to an ECI, that occurs to any subject must be reported within 24 hours to the Sponsor if it causes the subject to be excluded from the trial, or is the result of a protocol-specified intervention, including but not limited to washout or discontinuation of usual therapy, diet, placebo treatment or a procedure.

For the time period beginning at randomization through 14 days following cessation of treatment, any ECI, or follow up to an ECI, whether or not related to the Sponsor’s product, must be reported within 24 hours to the Sponsor, either by electronic media or paper. Electronic reporting procedures can be found in the EDC data entry guidelines. Paper reporting procedures can be found in the Investigator Trial File Binder (or equivalent).

The following events are considered events of clinical interest for this trial (main study and extension study):

1. An overdose of Sponsor's product, as defined in **Section 7.7.2.2.4**, Overdose, that is not associated with clinical symptoms or abnormal laboratory results is to be reported as a non-serious ECI, using the terminology "accidental or intentional overdose without adverse effect."
2. Adverse events associated with potential for abuse (euphoric mood, mania, hypomania, or similar events; see separate guidance document for details);
3. An elevated AST or ALT lab value that is ≥3 x the upper limit of normal (ULN) and an elevated total bilirubin lab value that is ≥2 x ULN and, at the same time, an alkaline phosphatase lab value that <2 x ULN, as determined by way of protocol-specified laboratory testing or unscheduled laboratory testing is to be reported as a non-serious ECI.
4. ALT or AST ≥3 x ULN and a ≥20% increase from baseline;
5. **Incident vasogenic edema in post-treatment MRI scans;
6. **Incident macrohemorrhage in post-treatment MRI scans;
7. **Incident superficial siderosis in post-treatment MRI scans;
8. **Incident microhemorrhage in post-treatment MRI scans;
9. Suicidal ideation or behavior (see separate guidance document for details);
10. Delirium that is ascertained by investigator or a qualified designee. The Confusion Assessment Method (CAM) should be used to verify delirium whenever feasible. [10];
11. A **clinically significant rash** in the investigator's judgment (such as a duration >2 weeks OR a rash that is >10% BSA, OR a rash causes significant discomfort not relieved by topical medication) OR a **severe rash** (as defined in **Section 7.3.3**);
12. Adverse events of clinically significant skin hypo- or depigmentation (see **Section 5.4** for details)

** Since routine MRI monitoring for safety has been discontinued, the central Reading Center is not automatically performing central reading for safety. Therefore, during the main study and the extension, the site investigator or radiologist should perform a local reading, as necessary. In some cases, central reads for safety may be performed. In the event that an Amyloid Related Imaging Abnormality (ARIA) is detected, then the Sponsor may request that the MRI be submitted for central reading.

A separate guidance document will be provided to sites for follow-up care of elevated LFT. Follow-up care for clinically significant rashes and severe rashes will also be detailed in another guidance document to sites, which includes evaluation by a dermatologist, photographs of skin lesions, and biopsy if indicated. For severe rashes, an adjudication committee will be adjudicating all cases of Stevens-Johnson Syndrome, erythroderma, toxic epidermal necrolysis, or DRESS syndrome based on photographs, biopsy results, and other available clinical data. Details will be described in the adjudication committee charter.

7.7.2.2.4 Overdose

An overdose is a significant variation above the recommended/scheduled dosage for a product. In this current trial an overdose of the investigational product MK-8931 is the administration of at least three times the daily dose of trial medication in a calendar day as specified in **Section 7.4.1.3.2** of this protocol.

All reports of overdose with and without an adverse event must be reported by the investigator within 24 hours to the Sponsor either by electronic media or paper. Electronic reporting procedures can be found in the EDC data entry guidelines. Paper reporting procedures can be found in the Investigator Trial File Binder (or equivalent).

For definitions and reporting requirements for amyloid PET tracer overdose, please refer to the PET IIOM.

7.7.2.2.5 Clinical Supply Complaint

A clinical supply complaint is defined as any communication concerning manufacturing, packaging, labeling or distribution (including adverse storage at depots) of a clinical supply that describes a potential defect related to its identity, strength, quality or purity after it is released and left the control of a Merck-approved packaging facility for distribution. A clinical supply GCP inquiry is defined as any communication of an event taking place at a trial site after the product was satisfactorily received at the trial site, which puts product disposition in question. Examples include adverse storage of product at the trial site and dosing past expiration. Alleged Counterfeit, Diversion and Tampering (CDT), adverse events and trial site errors/issues which do not put product disposition in question should not be reported.

The investigator shall take responsibility for and shall take all steps to maintain appropriate records and ensure appropriate supply, storage, handling, distribution and usage of investigational product in accordance with the protocol and any applicable laws and regulations. This responsibility includes reporting of all clinical supply complaints and/or clinical supply GCP inquiries to the Sponsor.

Clinical supplies complaints and GCP inquiries, as defined above, must be reported to the Sponsor within 1 business day of first becoming aware of the issue. Sponsor contact information and related reporting details can be found in the Investigator Trial File Binder.

7.7.2.2.6 Planned Hospitalization

A hospitalization planned by the subject prior to signing the ICF is considered a therapeutic intervention and not the result of a new SAE and should be recorded as medical history. If the planned hospitalization or procedure is executed as planned, the record in the subject's medical history is considered complete. However, if the event/condition worsens during the trial, it must be reported as an AE.

7.7.2.3 Monitoring

7.7.2.3.1 Monitoring Adverse Events

All adverse events that occur after the consent form is signed but before randomization must be reported by the investigator if they cause the subject to be excluded from the trial, or are the result of a protocol-specified intervention, including but not limited to washout or discontinuation of usual therapy, diet, placebo treatment or a procedure. From the time of randomization through 14 days following cessation of treatment, all adverse events must be reported by the investigator. Such events will be recorded at each examination on the Adverse Event case report forms/worksheets. The reporting timeframe for adverse events meeting any serious criteria is described in section 7.7.2.2.2. The investigator will make every attempt to follow all subjects with non-serious adverse events for outcome.

Electronic reporting procedures can be found in the EDC data entry guidelines. Paper reporting procedures can be found in the Investigator Trial File Binder (or equivalent).

Subjects who participate in the extension trial will not be followed up by telephone 14 days after the last protocol-specified visit in Part I.

Subjects will be questioned and/or examined by the investigator or a qualified designee for evidence of AEs. The questioning of subjects with regard to the possible occurrence of adverse events will be generalized such as, "How have you been feeling since your last visit?" The presence or absence of specific AEs should not be elicited from subjects. This does not preclude use of other sources of information that may suggest potential AE's (eg. NPI, C-SSRS).

Subjects having AEs will be monitored with relevant clinical assessments and laboratory tests, as determined by the investigator.

Adverse events, actions taken as a result of AEs, and follow-up results must be recorded in the electronic Case Report Forms (eCRF; **Section 9.2**), as well as in the subject's source documentation. Follow-up laboratory results should be filed with the subject's source documentation.

For all AEs that require the subject to be discontinued from the trial and SAEs, relevant clinical assessments and laboratory tests will be repeated as clinically appropriate, until final resolution or stabilization of the event(s).

7.7.2.3.2 Monitoring Laboratory Assessments

All laboratory assessments will be performed centrally at a certified laboratory selected by the sponsor. The clinical laboratory values will be reported to the investigator by the laboratory and he/she will review them for significance and consideration as an AE.

7.7.2.4 Assessment of Adverse Events

An investigator who is a qualified physician will evaluate all adverse events with respect to the elements outlined in **Table 4**. The investigator's assessment of causality is required for each adverse event. Refer to **Table 4** for instructions in evaluating adverse events.

Table 4 Evaluating Adverse Events

**Maximum Intensity**	**Mild**	awareness of sign or symptom, but easily tolerated (for pediatric trials, awareness of symptom, but easily tolerated)
	**Moderate**	discomfort enough to cause interference with usual activity (for pediatric trials, definitely acting like something is wrong)
	**Severe**	incapacitating with inability to work or do usual activity (for pediatric trials, extremely distressed or unable to do usual activities)
**Seriousness**	A serious adverse event (AE) is any adverse event occurring at any dose or during any use of Sponsor's product that:	
	**†Results in death**; or	
	**†Is life threatening; or** places the subject, in the view of the investigator, at immediate risk of death from the event as it occurred [Note: This does not include an adverse event that, had it occurred in a more severe form, might have caused death.]; or	
	**†Results in a persistent or significant disability/incapacity** (substantial disruption of one's ability to conduct normal life functions); or	
	**†Results in or prolongs an existing inpatient hospitalization** (hospitalization is defined as an inpatient admission, regardless of length of stay, even if the hospitalization is a precautionary measure for continued observation. (Note: Hospitalization [including hospitalization for an elective procedure] for a preexisting condition which has not worsened does not constitute a serious adverse event.); or	
	**†Is a congenital anomaly/birth defect (**in offspring of subject taking the product regardless of time to diagnosis); or	
	**Is a cancer;** (although not serious per ICH definition, is reportable to the Sponsor within 24 hours to meet certain local requirements); **or**	
	**Is associated with an overdose** (whether accidental or intentional). Any adverse event associated with an overdose is considered a serious adverse event. An overdose that is not associated with an adverse event is considered a non-serious event of clinical interest and must be reported within 24 hours.	
	**Other important medical events** that may not result in death, not be life threatening, or not require hospitalization may be considered a serious adverse event when, based upon appropriate medical judgment, the event may jeopardize the subject and may require medical or surgical intervention to prevent one of the outcomes listed previously (designated above by a †).	
**Duration**	Record the start and stop dates of the adverse event. If less than 1 day, indicate the appropriate length of time and units	
**Action taken**	Did the adverse event cause the Sponsor's product to be discontinued?	
**Relationship to Sponsor's Product**	Did the Sponsor's product cause the adverse event? The determination of the likelihood that the Sponsor's product caused the adverse event will be provided by an investigator who is a qualified physician. The investigator's signed/dated initials on the source document or worksheet that supports the causality noted on the AE form, ensures that a medically qualified assessment of causality was done. This initialed document must be retained for the required regulatory time frame. The criteria below are intended as reference guidelines to assist the investigator in assessing the likelihood of a relationship between the test drug and the adverse event based upon the available information.<br>**The following components are to be used to assess the relationship between the Sponsor's product and the AE**; the greater the correlation with the components and their respective elements (in number and/or intensity), the more likely the Sponsor's product caused the adverse event:	
	**Exposure**	Is there evidence that the subject was actually exposed to the Sponsor's product such as: reliable history, acceptable compliance assessment (pill count, diary, etc.), expected pharmacologic effect, or measurement of drug/metabolite in bodily specimen?
	**Time Course**	Did the AE follow in a reasonable temporal sequence from administration of the Sponsor's product?<br>Is the time of onset of the AE compatible with a drug-induced effect (applies to trials with investigational medicinal product)?
	**Likely Cause**	Is the AE not reasonably explained by another etiology such as underlying disease, other drug(s)/vaccine(s), or other host or environmental factors

**Relationship to Sponsor's Product (continued)**	**The following components are to be used to assess the relationship between the Sponsor's product and the AE: (continued)**	
	**Dechallenge**	Was the Sponsor's product discontinued or dose/exposure/frequency reduced?<br>If yes, did the AE resolve or improve?<br>If yes, this is a positive dechallenge. If no, this is a negative dechallenge.<br>(Note: This criterion is not applicable if: (1) the AE resulted in death or permanent disability; (2) the AE resolved/improved despite continuation of the Sponsor's product; (3) the trial is a single-dose drug trial); or (4) Sponsor's product(s) is/are only used one time.)
	**Rechallenge**	Was the subject re-exposed to the Sponsor's product in this trial?<br>If yes, did the AE recur or worsen?<br>If yes, this is a positive rechallenge. If no, this is a negative rechallenge.<br>(Note: This criterion is not applicable if: (1) the initial AE resulted in death or permanent disability, or (2) the trial is a single-dose drug trial); or (3) Sponsor's product(s) is/are used only one time.)<br>NOTE: IF A RECHALLENGE IS PLANNED FOR AN ADVERSE EVENT WHICH WAS SERIOUS AND WHICH MAY HAVE BEEN CAUSED BY THE SPONSOR'S PRODUCT, OR IF RE-EXPOSURE TO THE SPONSOR'S PRODUCT POSES ADDITIONAL POTENTIAL SIGNIFICANT RISK TO THE SUBJECT THEN THE RECHALLENGE MUST BE APPROVED IN ADVANCE BY THE U.S. CLINICAL MONITOR AND THE INSTITUTIONAL REVIEW BOARD/INDEPENDENT ETHICS COMMITTEE.<br><br>WHERE RESTRICTED LOCALLY BY IRBs, (INCLUDING SITES IN FRANCE): FOR SAEs JUDGED TO BE RELATED TO TRIAL DRUG, SUBJECTS CANNOT BE RECHALLENGED WITH TRIAL DRUG. SUBJECTS MUST BE DISCONTINUED FROM TRIAL DRUG.
	**Consistency with Trial Treatment Profile**	Is the clinical/pathological presentation of the AE consistent with previous knowledge regarding the Sponsor's product or drug class pharmacology or toxicology?
The assessment of relationship will be reported on the case report forms /worksheets by an investigator who is a qualified physician according to his/her best clinical judgment, including consideration of the above elements.		
**Record one of the following**:		**Use the following scale of criteria as guidance (not all criteria must be present to be indicative of a Sponsor's product relationship).**
**Yes, there is a reasonable possibility of Sponsor's product relationship.**		There is evidence of exposure to the Sponsor's product. The temporal sequence of the AE onset relative to the administration of the Sponsor's product is reasonable. The AE is more likely explained by the Sponsor's product than by another cause.
**No, there is not a reasonable possibility of Sponsor's product relationship**		Subject did not receive the Sponsor's product OR temporal sequence of the AE onset relative to administration of the Sponsor's product is not reasonable OR there is another obvious cause of the AE. (Also entered for a subject with overdose without an associated AE.)

Confidential

7.7.2.4.1 Reference Safety Information (RSI) for the Assessment of Expectedness of Adverse Events

The Reference Safety Information (RSI) for assessing the expectedness of an adverse event for the investigational product MK-8931 in this current trial is to be the most recent Investigator's Brochure for MK-8931.

7.7.2.4.2 Potential Toxicities of Investigational Products

Refer to the Investigator's Brochure for additional information on AEs related to toxicities observed to date.

7.7.2.5 Reporting Safety Observations by the Investigator to the Sponsor

7.7.2.5.1 Expedited Reporting

Any occurrence of the following events or outcomes in a subject in the trial must be reported expeditiously by the investigator or qualified designee to the sponsor's Global Safety representative or designee by entering all information relevant to the event in the appropriate eCRFs within **24 hours of learning of the event**. The Global Safety Intake Form – or a sponsor-approved equivalent form – should be used in the event that the EDC system is not functioning.

1. SAE (including SAEs associated with overdose, pregnancy, exposure during pregnancy or lactation);
2. Death;
3. Planned hospitalizations (not previously reported in the medical history);
4. Events of Clinical Interest (ECI);
5. Cancer.

Although pregnancy and lactation are not considered adverse events, it is the responsibility of investigators or their designees to report any pregnancy or lactation in a subject (spontaneously reported to them) that occurs during the trial.

Pregnancies and lactations that occur after the consent form is signed but before randomization must be reported by the investigator if they cause the subject to be excluded from the trial, or are the result of a protocol-specified intervention, including but not limited to washout or discontinuation of usual therapy, diet, placebo treatment or a procedure. Pregnancies and lactations that occur from the time of randomization through 14 days following cessation of Sponsor's product must be reported by the investigator. All reported pregnancies must be followed to the completion/termination of the pregnancy. Pregnancy outcomes of spontaneous abortion, missed abortion, benign hydatidiform mole, blighted ovum, fetal death, intrauterine death, miscarriage and stillbirth must be reported as serious events (Important Medical Events). If the pregnancy continues to term, the outcome (health of infant) must also be reported.

Such events must be reported within 24 hours to the Sponsor either by electronic media or paper. Electronic reporting procedures can be found in the EDC data entry guidelines. Paper reporting procedures can be found in the Investigator Trial File Binder (or equivalent).

If the investigator is unsure about when to report an observation from the lists above, the event or outcome should be reported to the sponsor or designee by entering all information relevant to the event in the appropriate eCRFs within 24 hours of learning of the event. The Global Safety Intake Form – or a sponsor-approved equivalent form – should be used in the event that the EDC system is not functioning.

Any observation reported to the sponsor or designee that is also an AE, is to be recorded in the eCRF (**Section 9.2**), as well as in the subject's source documentation, along with any actions taken as a result of AE and follow-up results.

If an autopsy is performed, available results should be entered into the EDC screens.

The investigator must assess causality of the event as relative to the investigational product administered in the trial as described in **Section 7.7.2.4.2**.

7.7.2.5.2 Expedited Reporting by the Sponsor to a Regulatory Health Authority

Global Safety will monitor data for safety. The Sponsor will manage the expedited reporting of relevant safety information to concerned health authorities, competent authorities, and IRBs/IECs in accordance with local laws and regulations.

7.7.2.5.3 Unblinding Treatment for a Subject During the Trial

To assess an occurrence of a safety observation, Global Safety may unblind the treatment of any subject for whom a safety observation was reported by the investigator to the sponsor as described in **Section 7.7.2.5.1**.

When the investigator or sub-investigator needs to identify the drug used by a subject and the dosage administered in case of emergency eg, the occurrence of serious adverse experiences, he/she will contact the emergency unblinding call center by telephone and make a request for emergency unblinding. As requested by the investigator or sub-investigator the emergency unblinding call center will provide the information to him/her promptly and report unblinding to the sponsor. The emergency unblinding call-center will make a record promptly however, the investigator or sub-investigator must enter the intensity of the adverse experiences observed, their relation to study drug, the reason thereof, etc., in the medical chart etc., before unblinding is performed.

Additionally, the investigator must go into the IVRS system and perform the unblind in the IVRS system to update drug disposition. In the event that the emergency unblinding call center is not available for a given site in this trial, IVRS/IWRS should be used for emergency unblinding in the event that this is required for subject safety.

In the event that unblinding has occurred, the circumstances around the unblinding (eg, date and reason) must be documented promptly, and the Sponsor Clinical Director notified as soon as possible. Only the principal investigator or delegate and the respective subject's code should be unblinded. Trial site personnel and Sponsor personnel directly associated with the conduct of the trial should not be unblinded.

7.7.3 Pharmacogenetics

7.7.3.1 Pharmacogenetics Endpoints and Analyses

The relationship between APOE genotype and the response to therapy will be evaluated as an exploratory outcome. HLA genotype and risk for rash will also be evaluated as an exploratory outcome. Additional exploratory pharmacogenetics (PGt) studies may be performed if significant Pharmacokinetic/Pharmacodynamic (PK/PD) relationships are observed or adverse events are identified. Genomic markers of disease may also be investigated. Pharmacogenetic studies will be conducted and the subsequent analysis will be compared to PK/PD results or clinical outcomes. Any significant PGt relationships to outcome will require validation in future clinical trials.

7.7.4 Other Endpoints

7.7.4.1 Pharmacodynamic Endpoints

The following pharmacodynamic endpoints will be assessed in addition to those defined in **Section 7.7.1.3.1**:

1. Ventricular brain volume, left hippocampal volume, right hippocampal volume, and whole brain volume changes-from-Baseline at Weeks 52 and 78 as determined by structural MRI.
2. Changes-from-Baseline at 78 weeks in the CSF concentrations of Aβ40, Aβ42, sAPPβ, and p-tau.
3. Change-from-Baseline in regional amyloid SUVR of [^{18}F]Flutemetamol at 78 weeks.

7.7.4.2 Pharmacoeconomic and Quality of Life Endpoints

1. Modified RUD Lite at Baseline and at Weeks 26, 52, and 78,
2. HEA at Baseline and at Weeks 26, 52, and 78; and the
3. EQ-5D at Baseline and at Weeks 26, 52, and 78.

7.8 Criteria for Early Termination of the Trial

The trial may be terminated early by the eDMC for safety concerns or based on the results of the interim efficacy analysis for futility (see **Section 8.2.9**).

8.0 STATISTICAL AND ANALYTICAL PLAN

This section outlines the statistical analysis strategy and procedures for Part I of the trial (see **Section 11.0** for the statistical analysis strategy and procedures pertaining to Part II). If changes are made to the Primary and/or Key Secondary Hypotheses or the statistical methods related to those hypotheses after the trial has begun, but before any unblinding, then the protocol will be amended (consistent with ICH Guideline E-9). Changes to non-key secondary, exploratory, or other non-confirmatory analysis plans made after the protocol has been finalized, along with an explanation as to when and why they occurred, will be listed in the Clinical Study Report (CSR) for the trial. Post hoc exploratory analyses will be clearly identified in the CSR. No separate Statistical Analysis Plan (SAP) will be issued for this trial. The statistical analysis of the data obtained from this trial will be the responsibility of the designee from the Clinical Biostatistics Department of the sponsor.

The official, final database will not be unblinded until a medical/scientific review has been performed, protocol violators have been identified, and the data have been declared final and complete.

The exact timing of the predose efficacy assessments (screening or baseline) is provided in the Trial Flowchart (**Section 2.2.2**). Without loss of generality, and within **Section 8** only, a distinction will not be made between the Screening Visit and the Baseline Visit; all predose assessments will be referred to generally as "baseline" assessments. In the event that an assessment was taken at both the Screening Visit and at the Baseline Visit, the data from the Baseline Visit will be used in the analysis.

8.1 Subject Populations to be Analyzed

The analysis populations are the Full Analysis Set population, the Modified Full Analysis Set population and the All-Patients-as-Treated population.

- The Full Analysis Set (FAS) population will serve as the primary population for the analysis of efficacy data in this trial. The FAS population consists of all randomized subjects who have a baseline observation and at least one within-analysis-window (± 6 weeks) post-Randomization observation for the analysis endpoint subsequent to at least one dose of trial treatment. Subjects will be included in the treatment group to which they were randomized. Since the Sponsor's standing internal DMC (siDMC) reviewed unblinded analyses (including the ADAS-Cog) from the first 200 subjects enrolled during Part I, these subjects will be excluded from the FAS population, and hence all efficacy analyses.

- The Modified Full Analysis Set (MFAS) population will be utilized for sensitivity analyses and is defined identically to the FAS population with one exception: subjects will be required to have either 1) a baseline measurement for the analysis endpoint or 2) at least one within-analysis-window (± 6 weeks) post-dose, post-randomization observation for the analysis endpoint, but not necessarily both.
- The All-Patients-as-Treated (APaT) population will be used for the analysis of safety data in this trial. The APaT population consists of all randomized subjects who received at least one dose of trial treatment, with subjects included in the treatment group corresponding to the trial treatment they actually received. For most subjects this will be the treatment group to which they were randomized. Subjects who take incorrect trial treatment for the entire treatment period will be included in the treatment group corresponding to the trial treatment actually received. Since the siDMC reviewed unblinded analyses from the first 200 subjects enrolled during Part I, these subjects will be excluded from the primary safety analyses.
- For laboratory, vital sign, and ECG endpoints, at least one postdose measurement is required for inclusion in the analysis of each specific parameter, and a Baseline measurement is required for change-from-Baseline analyses (safety only).

Though no Per-Protocol analyses are planned, a list of protocol violators (eg, subjects misdiagnosed with AD, lack of trial medication compliance, change in background therapy during the trial) will be finalized prior to the final Part 1 database lock and subsequent unblinding.

8.2 Efficacy Analyses

Details pertaining to the analysis of all endpoints are discussed below. Table 5 provides a summary of the Primary and Secondary Endpoints to be analyzed, as well as the analysis approaches that will be used.

The primary analysis approach will include all efficacy observations in the FAS population, including those obtained after the discontinuation of study medication. .

Subjects from both cohorts will be included in the efficacy analyses (though subjects enrolled in any dose that is dropped during their participation in the study will not be included in the primary efficacy analyses of the remaining doses). However, since the siDMC will be reviewing unblinded data from the first 200 subjects enrolled, the first 200 subjects enrolled will be excluded from all efficacy analyses.

As noted in the efficacy sections below, nominal 95% confidence intervals will be computed for many treatment comparisons. However, all confidence intervals corresponding to formal hypothesis tests under the prespecified multiplicity strategy (Section 8.2.3) will be constructed at the 97.51% level.

Table 5 Analysis Strategy for Efficacy Variables

Endpoint/Variable (Description, Time Point)	Primary vs. Supportive Approach	Statistical Method†	Analysis Population	Missing Data Approach
**Primary Endpoints**				
**Coprimary Endpoints:**				
CFB at Week 78 in ADAS-Cog score	P	Longitudinal ANCOVA	FAS	Model-based
	S	Longitudinal ANCOVA	FAS	Pattern-Mixture Model (Tipping Point)
	S	Longitudinal ANCOVA (Exclude data collected after the introduction of AChEIs and/or study medication discontinuation)	FAS	Model-based
	S	cLDA	MFAS	Model-Based
CFB at Week 78 in ADCS-ADL score	P	Longitudinal ANCOVA	FAS	Model-based
	S	Longitudinal ANCOVA	FAS	Pattern-Mixture Model (Tipping Point)
	S	Longitudinal ANCOVA (Exclude data collected after the introduction of AChEIs and/or study medication discontinuation)	FAS	Model-based
	S	cLDA	MFAS	Model-Based
**Key Secondary Endpoint**				
CFB at Week 78 in CDR-SB score	P	Longitudinal ANCOVA	FAS	Model-based
	S	Longitudinal ANCOVA	FAS	Pattern-Mixture Model (Tipping Point)
	S	Longitudinal ANCOVA (Exclude data collected after the introduction of AChEIs and/or study medication discontinuation)	FAS	Model-based
	S	cLDA	MFAS	Model-Based
**Other Secondary Endpoints**				
CFB at Week 78 in total hippocampal volume	P	Longitudinal ANCOVA	FAS	Model-based

Table 5 Analysis Strategy for Efficacy Variables

Endpoint/Variable (Description, Time Point)	Primary vs. Supportive Approach	Statistical Method†	Analysis Population	Missing Data Approach
CFB at Week 78 CSF total tau	P	Longitudinal ANCOVA	FAS	NA
CFB at Week 78 on brain amyloid load	P	Longitudinal ANCOVA	FAS	NA
Percentage of Responders at Week 78	P	Logistic Regression	FAS	Impute as Failure
CFB at Week 78 in NPI score	P	Longitudinal ANCOVA	FAS	Model-based
CFB at Week 78 in MMSE score	P	Longitudinal ANCOVA	FAS	Model-based

The primary model contains categorical terms for treatment, time, geographic region, gender, baseline use of Vitamin E, APOE genotype, study cohort, and the interaction of time-by-treatment, with the Baseline values of MMSE, and age included as continuous covariates. Terms for the baseline value and the baseline-by-time interaction of the dependent variable will also be included.

ADAS-Cog=Alzheimer's Disease Assessment Scale Cognitive subscale; ADCS-ADL=Alzheimer's Disease Cooperative Study Activities of Daily Living Inventory; CDR-SB=Clinical Dementia Rating Sum of Boxes; CFB=Change from Baseline; ANCOVA=Analysis of Covariance; cLDA=Constrained Longitudinal Data Analysis; FAS=Full Analysis Set; MMSE=Mini-Mental State Examination; NA=Not Applicable; NPI=Neuropsychiatric Inventory; P=Primary; S=Supportive.

8.2.1 Primary Efficacy Analysis

For the Primary Hypotheses refer to **Section 6**.

The Primary Hypotheses will be tested on the basis of the two Coprimary Endpoints, the 78-week change from Baseline in ADAS-Cog score and the 78-week change from Baseline in ADCS-ADL score.

The primary analysis approach will be conducted separately on each of the endpoints.

Confidential

A longitudinal ANCOVA model will be used on the change scores, with time treated as a categorical variable so as not to impose any restriction on the trajectory of the means over time. The analysis model will adjust for the categorical factors of geographic region (US/Canada, Europe/Australia/New Zealand, Japan, Rest of the World), treatment (one or two remaining dose[s] of MK-8931 or placebo), gender, APOE genotype (APOE 4 positive, APOE 4 negative), baseline use of Vitamin E (0-400 IU/day, > 400 IU/day), baseline AD medication (use of AChEI or memantine, no use of AChEI or memantine), study cohort (Safety Cohort, Main Cohort) and the interaction of time-by-treatment, with the baseline values of MMSE and age included as continuous covariates. The baseline value of the dependent variable, as well as the baseline-by-time interaction term will also be included. The Week-78 change-from-Baseline mean treatment differences (MK-8931 – placebo), corresponding confidence intervals (CIs), and P-values will be estimated from this model. An unstructured covariance matrix will be used to model the correlation among repeated measurements.

Three supportive analyses will be conducted on each of the Coprimary Endpoints. These analyses will be conducted to assess the effect of 1) missing data using a pattern-mixture model (“tipping-point” analysis), 2) the use of off-regimen data after the subject has permanently discontinued trial medication or after the subject has initiated AChEIs and 3) analyzing the data using the cLDA model on the MFAS population.

For the first sensitivity analyses (tipping-point), the model-based estimates of the mean treatment difference on the change scores, along with the corresponding confidence intervals and p-values will be provided for a range of *c* values which have been subtracted from the values imputed for the active arms (see 8.2.10 for more details). Of specific interest is the smallest *c* value that, for a given endpoint, transforms a statistically significant result to a non-statistically significant result.

For the second sensitivity analysis (excluding observations obtained after study medication discontinuation or after the initiation of AChEIs, memantine, or Vitamin E), the model-based estimates of the mean treatment difference on the change scores, along with the corresponding confidence intervals and p-values will be provided.

For the third sensitivity analysis (cLDA model on the MFAS population), the analysis of the primary efficacy endpoint will be conducted using a constrained longitudinal data analysis (cLDA) method proposed by Liang and Zeger [11]. This model assumes a common mean across treatment groups at baseline and a different mean for each treatment at each of the post-baseline time points. The response vector consists of baseline and the values observed at each post-baseline time point. Additional details pertaining to the cLDA model are included in Section 12.5. The model-based estimates of the mean treatment difference on the change scores, along with the corresponding confidence intervals and p-values will be provided as obtained using the cLDA model, with all of the same covariates that were included in the primary model (now excluding the terms for baseline and baseline-by-time interaction). An unstructured covariance model will be used.

Although the baseline measurement is included in the response vector, it is independent of treatment. Hence, the Baseline means are constrained to be the same for different treatment groups. Note that in the event that there are no missing data, the estimated treatment difference from the above cLDA model will be identical to that from a traditional longitudinal analysis of covariance (ANCOVA) model that uses the Baseline value as a covariate. However, unlike longitudinal ANCOVA, the cLDA model accounts for variability in the baseline values, thus providing more accurate standard errors and CIs for individual treatment effects. Additional details pertaining to the use of the cLDA model in this trial are included in **Appendix 4**.

Additional details pertaining to the handling of missing data may be found in **8.2.10**. In order to better adhere to the intent-to-treat principle, subjects will be encouraged to continue in the trial even if they discontinue treatment (often referred to as "retrieved dropout").

Analyses will also be conducted to assess the effect of MK-8931 over time, in part to investigate the possibility that MK-8931 has a disease-modifying effect. Specifically, the Week-13 change-from-Baseline mean treatment differences (MK-8931 – placebo), corresponding 95% confidence intervals (CIs), and nominal (ie, unadjusted) P-values will be estimated using the above model, as will the difference (Week 78 – Week 13) in treatment difference means. A nominally (unadjusted p-value < 0.05) significantly larger Week 78 treatment difference, as compared to Week 13, coupled with a statistically significant difference in treatment at 78 weeks indicates increasing efficacy over time and hence the possibility that MK-8931 has a disease modifying effect.

Confidential

Additionally, the relationship between the primary endpoints and the Change-from-Baseline in Total Hippocampal Volume will be examined both graphically and through the computation of correlations. Although no formal hypotheses testing will be performed on the correlation analyses, a positive relationship between this biomarker and the primary endpoints, in conjunction with statistically significant results on both primary endpoints and this biomarker, would provide additional evidence that MK-8931 has a disease modifying effect. The relationships between the primary endpoints and the other biomarkers (change-from-baseline in whole brain volume, left hippocampal volume, right hippocampal volume, ventricular volume, CSF total tau, CSF p-tau, Aβ42, Aβ40, and cortical amyloid load) will be explored in a similar fashion.

8.2.2 Key Secondary Efficacy Analysis

For the Key Secondary Hypotheses refer to **Section 6**.

CDR-SB

The analysis of the Key Secondary Endpoint, CDR-SB, will be conducted in a manner similar to that used for the Coprimary Endpoints.

8.2.3 Multiplicity

Strategies for controlling Type 1 error and for determining formal statistical significance on the Primary and Key Secondary Hypotheses are described below (assuming that two doses are brought into the Main Cohort; please see **Section 8.2.6** should only one dose remain at the end of the trial). With the exception of the Health Economic Endpoints, p-values (active vs. placebo) will be provided for all primary, secondary, and exploratory endpoints, regardless of the outcome of the multiplicity-controlled testing of the primary and key secondary hypotheses. These nominal p-values should be regarded as a measure of strength of association between the endpoint and the treatment effect rather than as formal tests of hypotheses.

As noted in **Section 5.3**, the eDMC will be monitoring for potential cognitive worsening throughout the trial as part of their charge to ensure subject safety. Subsequent to the first formal safety IA, the eDMC will be provided with ADAS-Cog analyses at their periodic safety reviews, unblinded at the treatment group level, solely for this purpose. There is no intention of stopping the trial due to positive efficacy at any of these safety reviews. Nevertheless, since unblinded analyses on one of the primary efficacy endpoints is being periodically reviewed, a small amount of alpha ($\alpha = 0.0001$) will be allocated for each of these looks, purely for statistical rigor. An allowance will be made such that a total of up to nine of these unblinded reports on ADAS-Cog may be presented throughout the duration of this trial at these periodic safety reviews.

As detailed in **Section 8.2.9**, one prespecifed futility analysis will be conducted, for which unblinded analyses for the ADAS-Cog, the ADCS-ADL, and the CDR-SB will be presented to the eDMC. Though there is no intention for stopping for overwhelming efficacy at this analysis, a small amount of alpha (α = 0.0001) will be allocated, again primarily for statistical rigor.

Since there are two active doses (generally referred to as the low and high dose within this section) and six endpoints on which strong control is desired, there are twelve total hypotheses on which strong Type 1-error control must be maintained at a familywise α = 0.0498 level (with a total of α = 0.0002 allocated to the [up to] nine unblinded safety reviews of ADAS-Cog and the one prespecifed interim efficacy analysis via the Haybittle-Peto method[23,24]). This control will be achieved by utilizing a Bonferroni approach in conjunction with a closed-testing sequential approach. Specifically, a separate hypothesis family will be created for each of the two active doses (with the one active dose within each family to be compared to placebo). Each family will be comprised of six hypotheses corresponding to the two Coprimary Endpoints, the one Key Secondary Endpoint, and the three biomarker endpoints (total hippocampal volume, total tau, and cortical amyloid load using PET imaging). Using a Bonferroni approach, each family will be tested *independently* at the α = 0.0249 level.

Within each dose family, the two Primary Hypotheses will be sequentially tested (ADAS-Cog followed by ADCS-ADL) at the α = 0.0249 level. If both primary null hypotheses are rejected, then testing may proceed to the Key Secondary Hypothesis (still at α = 0.0249), and in a similar fashion on to sequentially test the CFB in total hippocampal volume, the CFB in total tau, and the CFB in cortical amyloid load. This testing strategy is diagrammed in **Figure 1**.

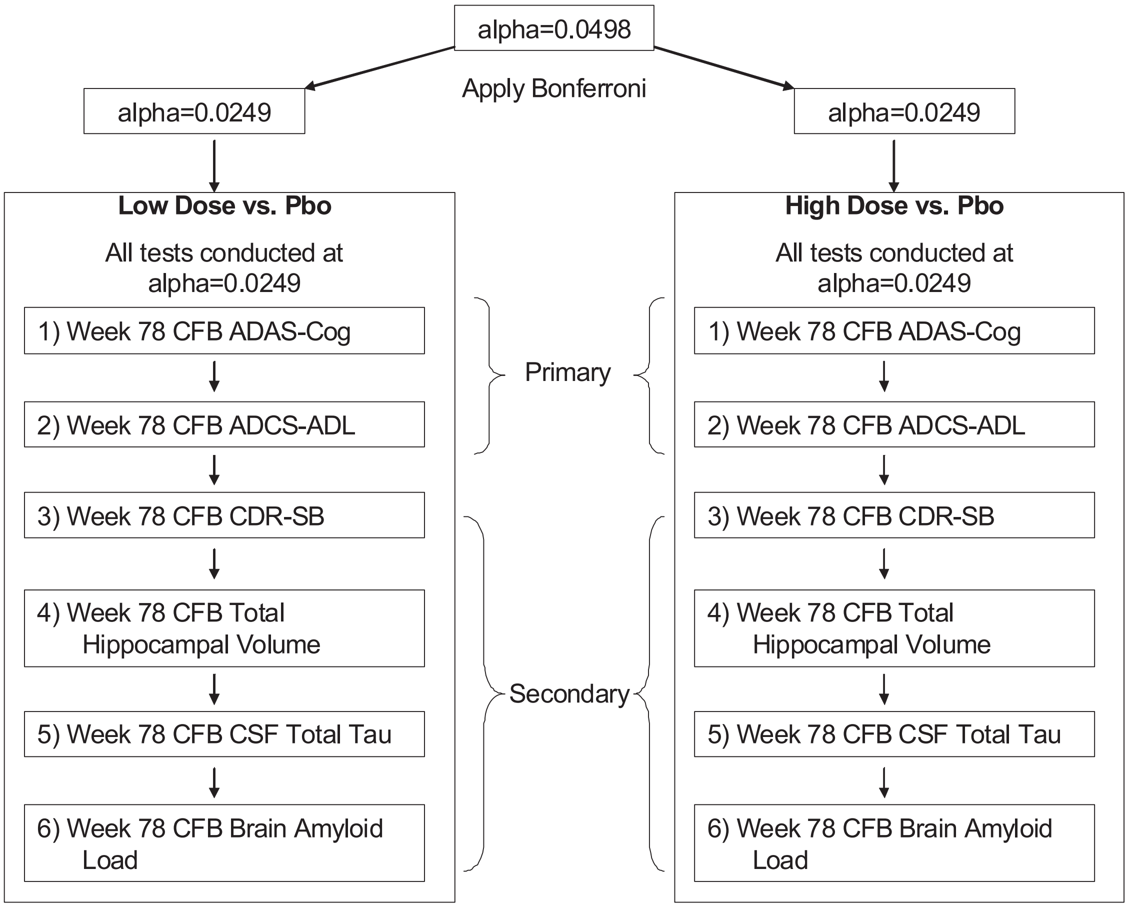

ADAS-Cog=Alzheimer's Disease Assessment Scale Cognitive subscale; ADCS-ADL=Alzheimer's Disease Cooperative Study Activities of Daily Living Inventory; CDR-SB=Clinical Dementia Rating Sum of Boxes; CFB=Change-from-Baseline; CSF=Cerebrospinal Fluid; Low Dose=12 mg; High Dose=40 mg

Figure 1 Testing Strategy to Address Multiplicity

8.2.4 Other Secondary Efficacy Analyses

Only the primary analysis approach will be used (ie, no supportive analysis will be conducted) for the other secondary efficacy endpoints.

The testing of the secondary hypotheses associated with the CFB in total hippocampal volume, the CFB in total tau, and the CFB in brain amyloid load will be conducted using the primary analysis model (with total tau log-transformed prior to analysis). Details pertaining to the collection of these measures will be described in separate procedure and technical manuals specific for volumetric MRI (vMRI) and PET imaging.

The analysis of the proportion of Week 78 responders will be conducted using logistic regression with the same covariates as those used in the primary analysis model, with the exception of any terms involving “time”. The odds ratios, corresponding 95% CIs, and P-values will be estimated from this model. In the event that the model fails to converge, terms will be sequentially removed from the model (until convergence is obtained) in the following order: study cohort, gender, geographic region, baseline use of Vitamin E, APOE genotype, baseline AD medication.

In order to determine which subjects are responders, a linear regression will be conducted, on the subject level, yielding an estimated 78-week rate of change (ie, a slope) for each subject. Each subject must meet three criteria in order to be declared as a 78-week responder. Each subject must have: 1) ADAS-Cog and ADCS-ADL responses at baseline and 78 weeks of treatment, 2) an ADAS-Cog slope less than 4.0 over 78 weeks, and 3) an ADCS-ADL slope greater than -6.3 over 78 weeks. A subject failing to meet any of these criteria will be designated as a Week 78 nonresponder. Separate cumulative distribution plots will also be provided for each endpoint, with the 78-week CFB score on the x-axis.

The analysis of the other secondary endpoints (ie, changes-from-Baseline at 78 weeks of treatment for NPI and MMSE scores) will be conducted in a manner similar to that used for the Coprimary Endpoints.

8.2.5 Exploratory Analyses

It is noted that no supportive analyses (eg, to account for the effect of missing data or protocol violations) will be conducted for any of the exploratory analyses. Basic summary statistics will be provided (mean, standard deviation, quartiles, counts and percentages, as appropriate) by treatment, timepoint, and baseline AD severity (MMSE >20, MMSE ≤ 20) for all endpoints.

ADAS-Cog, ADCS-ADL, CDR-SB, and MMSE at Other Timepoints

The analysis of ADAS-Cog, ADCS-ADL, CDR-SB, and MMSE at time points other than 78 Weeks will be conducted using the same model that was used to analyze the Coprimary Endpoints.

Additionally, summary statistics by timepoint (Month 6, Month 12, and Month18) and baseline AD severity will be provided either by question (ADAS-Cog and CDR-SB) or by domain (ADCS-ADL and MMSE), as appropriate.

NPI

The analysis of NPI at time points other than 78 Weeks will be conducted using the same model that was used to analyze the Coprimary Endpoints. Further, model-based analyses (using the primary analysis model) will be conducted on each of the domains of the NPI at the 78-Week timepoint.

Structural MRI

Analyses of the volumes of other brain regions (including ventricular volume, left hippocampal volume, right hippocampal volume, whole brain volume) will be conducted at all available timepoints using the same model that was used to analyze the Coprimary Endpoints.

CSF Biomarkers

Analyses of the other CSF biomarkers (Aβ40, Aβ42, sAPPβ, and p-tau) at 78 weeks of treatment will be conducted using the same model that was used to analyze the Coprimary Endpoints. Historical data indicates that p-tau is lognormally distributed and will therefore be analyzed on the log-scale.

Health Economic and Quality of Life Endpoints

The Modified RUD Lite will be evaluated by tabulating the counts and percentages of each living-arrangement category across the treatment arms and visits.

For HEA, the counts and percentages of each category of non-trial medical visit will be tabulated by treatment group and visit.

The EuroQol Five Dimension Questionnaire (EQ-5D) will be evaluated by tabulating the counts and percentages of each health dimension by treatment group and visit. Basic summary statistics will also be computed for the assessment of subject health state (0-100 scale) by treatment and visit.

Pharmacogenetic Analyses

The relationship between APOE genotype and the response to therapy will be evaluated as an exploratory outcome. Additional exploratory pharmacogenetic studies may be performed, eg, if significant pharmacokinetic/pharmacodynamic (PK/PD) relationships are observed or AEs are identified. Genomic markers of disease may also be investigated. Pharmacogenetic studies will be conducted with biostatistical design and analysis, and compared to PK/PD results or clinical outcomes.

8.2.6 Dropping Dose Arms

Three dose groups will be included in the initial Safety Cohort. The 60 mg dose will not continue into the Main Cohort; the 12 mg and/or the 40 mg doses will move into the Main Cohort depending on a review of the Safety Cohort data by the governance committees. While the current expectation is to take both doses forward for the Main Cohort, it is possible that only one dose will continue into the Main Cohort depending on the observed safety and tolerability from the Safety Cohort.

In the event that only one dose is brought forward into the Main Cohort, all efficacy hypotheses will be formally tested at the α = 0.0498 level and the sample size will be readjusted (to 690 subjects per group) to maintain power at 90%. The increase in sample size will maintain 90% for the joint success of both primary hypotheses and would be required to compensate for the loss of power due to the loss of one of the active arms.

If the high dose arm is dropped after the initiation of the Main Cohort but prior to the futility analyses: The number of subjects randomized into the remaining arms will be increased from 570 (three arms) to 760 (two arms), still testing the one remaining active at α=0.0249 at the final analysis.

The total enrollment that is necessary, which requires unblinded enrollment information to account for the additional dropped arm(s), will be communicated to the Sponsor by the eDMC.

If the high dose arm is dropped after the futility analyses: The number of subjects to be randomized will not be changed, maintaining the α-level for the one remaining treatment comparison for the primary hypothesis at α=0.02495. In addition, the study may be discontinued if the low dose arm, absent the high dose arm, would have failed the futility check at the time of the futility analyses (as determined by the unblinded statistician).

8.2.7 Subgroup Analyses

Subgroup analyses will be conducted on all nine primary and secondary endpoints. The consistency of treatment effect across various subgroups will be assessed through the computation of within-group summary statistics (model-based and as-observed) Further, model-based between-group treatment differences and the corresponding 95% CIs will be constructed for those comparisons (active vs. placebo), for which both treatment groups within the subgroup level have at least 85 subjects (roughly 15% of subjects randomized). No formal statistical testing of the treatment-by-subgroup interactions will be performed. The following subgroups will be examined:

- Gender (male, female);
- Age (< trial median age, ≥ trial median age);
- Race (white, black, Asian, other);
- Randomization Cohort (Safety Cohort, Main Cohort)
- Ethnicity (Hispanic, Not Hispanic);
- Geographic Region (US/Canada, Europe/Australia/New Zealand, Japan, Rest of the World);
- APOE Genotype (APOE 4 positive, APOE 4 negative);

- Disease Severity via MMSE (mild AD: MMSE >20; moderate AD: MMSE $\leq$ 20);
- Trial Completion Status (Completer, Non-completer);
- AD Treatment at Screening (use of AChEI alone, use of memantine alone, use of AChEI and memantine, no use of AChEI or memantine);
- Vitamin E use at Screening (0-400 IU/day, > 400 IU/day)
- Behavioral Symptoms at Baseline (without symptoms NPI = 0, with symptoms NPI > 0);
- Total Hippocampal Volume (AD profile positive, AD profile negative);
- Evidence of Brain Amyloidosis in CSF and/or amyloid PET substudies (positive, negative);
- Highest Education Level (No Undergraduate Degree, Undergraduate Degree or Higher)

Regarding total hippocampal volume, an AD profile will be determined as described in the technical manual. The precise criteria to define an AD profile positive and AD profile negative subjects have not yet been determined but will be selected based on ongoing research at academic sites which is expected to be published before the end of this trial. Similarly, cutoffs for evidence of brain amyloidosis, as measured by CSF and PET, will also be defined based on ongoing studies. All cutoffs will be prespecified prior to the final database lock and will be documented in a memo-to-file.

8.2.8 Justification of Sample Size

8.2.8.1 Parameter Estimates for Coprimary Endpoints and Key Secondary Endpoints

Estimates of the following parameters were compiled for the sample-size calculations for each of ADAS-Cog, ADCS-ADL, and CDR-SB:

- Mean progression rate on placebo over 78 weeks of treatment,
- Standard deviation on placebo at Baseline,
- Standard deviation on placebo at 78 weeks of treatment, and
- Correlation between the Baseline and 78-week measurement.

The primary sources for parameter estimates were 1) a manuscript from Schneider and Sano[12] containing a compilation of 18-month data from recently completed and ongoing Alzheimer's disease clinical trials and 2) manuscripts from two 12-month in-house trials (MK-0677 P030 and MK-0966 P091). A weighted average (according to sample size) of the respective parameter estimates (mean progression rate on placebo over 78 weeks of treatment and change-from-Baseline variance at 78 weeks of treatment) was taken from trials that had enrolled subjects with MMSE scores between 14 and 26, inclusive. Estimates of the within-endpoint correlation between Baseline and the 78-week measurement were obtained from the ongoing Alzheimer's Disease Neuroimaging Initiative (ADNI) natural history study. The ADNI dataset was also used to model a monotonically increasing function that describes the standard deviation for each endpoint. The standard deviation function, within-endpoint correlation and change-from-Baseline standard deviation were used to obtain estimates of the endpoint standard deviations at Baseline and at 78 weeks of treatment (since these manuscripts did not contain these estimates directly). Estimates from the in-house 12-month trials were converted to 18-month estimates using linear extrapolation. **Table 6** lists the final parameter estimates which serve as the underlying parameter assumptions for the sample-size calculations. All between-endpoint correlations were assumed equal to 0.5.

Table 6 Parameter Assumptions for Progression Rate on Placebo, Standard Deviations, and Within-Endpoint Correlations for ADAS-Cog, ADCS-ADL, and CDR-SB

Endpoint	78-Week Placebo Progression Rate	Baseline Standard Deviation	78-Week Standard Deviation	Correlation (Baseline, Week 78)
ADAS-Cog	6.12	7.75	12.71	0.70
ADCS-ADL	-9.64	10.71	17.24	0.68
CDR-SB	2.39	2.28	3.88	0.62

ADAS-Cog=Alzheimer's Disease Assessment Scale Cognitive subscale; ADCS-ADL=Alzheimer's Disease Cooperative Study Activities of Daily Living Inventory; CDR-SB=Clinical Dementia Rating Sum of Boxes

8.2.8.2 Parameter Estimates for Secondary Biomarkers / Substudy Endpoints

Total hippocampal volume (THV): Using data from the ADNI, estimates for the 18-month changes in THV were obtained for both AD subjects and healthy controls. The decrease in volume of the healthy controls was subtracted from the difference in AD subjects to obtain the decrease in volume due to AD (which is the volume open to improvement via intervention). These estimates are provided in **Table 7** along with the required estimates of the standard deviation and correlation.

Total tau: Using data from the ADNI, estimates for total tau were obtained for AD subjects and healthy control at both baseline and 12 months (only 12-month data were available). For both populations, there appears to be very minimal change in Total tau over 12 months; therefore expected drug effect will measured as a percent reduction in the difference between the two populations at 12 months. The relevant estimates are located in **Table 7**.

Cortical Amyloid Load: Using PiB PET data from the ADNI, estimates for cortical amyloid load were obtained for AD subjects and healthy control at both baseline and 18 months. For both populations, there does not appear to be any increase in PET signal over 18 months; therefore drug effect will be measured as a percent reduction in the difference between the two populations (rather than as a percent reduction in progression in the AD group as is the metric for most other endpoints). The relevant estimates are located in **Table 7**. It is noted that these estimates are based off of very limited data (n=13 at 12 months).

Table 7 Parameter Assumptions for Progression Rate on Placebo, Standard Deviations, and Within-Endpoint Correlations for THV, Total Tau, and Cortical Amyloid Load

Endpoint	Difference from Healthy Controls at 78 weeks	Baseline Standard Deviation	78-Week Standard Deviation	Correlation (Baseline, Week 78)
THV	197	1004	1035	0.985
log(Total Tau)	0.69	0.725	0.725	0.942
Amyloid Load	0.70	0.364	0.463	0.796

8.2.8.3 Assumptions for the Effect of MK-8931

The assumed true treatment difference used to calculate power and sample size is ideally based on the observed drug effect from previous trials. In the absence of such evidence (as is the case in this trial), the next best approach is to power a trial based on the minimal effect that is considered clinically meaningful. Unfortunately, there is no firm guidance as to what this effect should be for an Alzheimer's drug which is thought to have a disease-modifying effect.

Based on expert opinions, some previously conducted disease-modifying trials have been powered to detect a minimal treatment difference of 25% to 30% under various assumed rates of disease progression(13,14,2,15). On the other hand, a 2-point difference in ADAS-Cog change from Baseline at 78 weeks has been suggested as the minimal clinically important change(16) based on the results of a natural history trial(17) that showed that a decline of more than 2 points in the first 78 weeks of the trial was associated with a significantly increased risk of losing one basic ADL in the next 78 weeks of the trial.

Similar recommendations of clinical significance are not available for either ADCS-ADL or CDR-SB. The ADAS-Cog, ADCS-ADL, and CDR-SB, however, all have similar standardized effect sizes for change. Therefore, a reasonable assumption is that a clinically meaningful change on ADAS-Cog correlates with a clinically meaningful change in the other two endpoints.

Using a 2-point difference in ADAS-Cog change from Baseline as the definition for a clinically meaningful change, in conjunction with the estimated natural ADAS-Cog progression rate of 6.1 points over 78 weeks, the clinically meaningful effect can be computed as a percentage and is approximately 35%. Assuming that a 35% change is also clinically meaningful for both the ADCS-ADL and CDR-SB, a 3.37-point difference in ADCS-ADL change from Baseline and a 0.84-point difference in CDR-SB change from Baseline are also considered as clinically meaningful.

Without any efficacy data on MK-8931 this trial is powered under the assumption that the low dose will produce the minimal clinically meaningful effect (ie, a slowing of progression). Further, it is conservatively assumed that the high dose is no more efficacious than the low dose.

Table 8 contains the assumed change-from-Baseline treatment effects (active - placebo) at 78 weeks of treatment under different assumed effects of the low and high doses. The bolded row within the table indicates the assumptions on which the current trial is powered.

Table 8 Assumed Change-From-Baseline Treatment Effects (Active – Placebo) at 78 Weeks of Treatment for Various Potential Efficacy Configurations of the High and Low Doses of MK-8931

	Endpoint [a]		
Drug Effect [b]	ADAS-Cog	ADCS-ADL	CDR-SB
30%	1.84	-2.89	0.72
**35%**	**2.14**	**-3.37**	**0.84**
40%	2.45	-3.86	0.96

ADAS-Cog=Alzheimer's Disease Assessment Scale Cognitive subscale; ADCS-ADL=Alzheimer's Disease Cooperative Study Activities of Daily Living Inventory; CDR-SB=Clinical Dementia Rating Sum of Boxes; HD=high dose; LD=low dose.

[a] Progression rate at 78 weeks of treatment with placebo is assumed to be 6.12 points for ADAS-Cog, -9.64 points for ADCS-ADL, and 2.39 points for CDR-SB.

[b] Drug Effect is the improvement in 78-week progression rate provided. The true underlying treatment effect of the LD and HD groups are assumed to be equal.

Similarly, a 35% drug effect was also assumed for all endpoints present in Section 8.2.7, and is based on the population differences (AD – healthy controls).

8.2.8.4 Determining Sample Size / Power

8.2.8.4.1 Primary and Key Secondary Endpoints

The sample size computations were conducted via simulation to account for the selected multiplicity approach, as well as to incorporate the effect of the futility analysis on the overall Type II error (ie, power). This simulation was conducted using the assumptions from Table 6 and Table 8 above. Sample sizes were calculated to achieve 90% overall power to 1) demonstrate significance of both Coprimary Efficacy Endpoints for at least one dose and 2) demonstrate significance of both Coprimary Endpoints as well as the first Key Secondary Efficacy Endpoint (CDR-SB) for at least one dose. The required number of randomized subjects (after accounting for a 5.8% dropout rate every 13 weeks; ie, a 30% dropout rate at 78 weeks) for various scenarios is contained in Table 9. The bolded row within the table represents the scenario on which the current trial is powered.

Confidential

It is noted that the sample sizes presented below do not account for the first 150 subjects in the three retained treatment arms that will be excluded from the efficacy analysis, nor does it account for the approximately 100 subjects in the dropped dose arm (assuming that two active dose arms go forward into the Main Cohort). That is, approximately 250 (overall) more subjects will need to be enrolled above those numbers provided in the table below.

Table 9 Number of Randomized Subjects Required for 90% Overall Power Under Different Assumed Drug Effects[e]

	Sample Size Per Arm[a]	
**Assumed Drug Effect[b]**	**Both Primary Endpoints[c]**	**Both Primaries and the first Key Secondary Endpoint[c.d]**
30%	770	810
**35%**	**570**	**585**
40%	440	455

HD=High Dose; LD=Low Dose.

a Sample sizes were simulated using 20,000 runs and are rounded to the nearest 5 subjects.

b Drug Effect refers to the effect (% improvement) of an active dose as compared to placebo. The true underlying treatment effect of the LD and HD groups are assumed to be equal.

c ADAS-Cog and ADCS-ADL are the Coprimary Efficacy Endpoints; CDR-SB is the Key Secondary Efficacy Endpoint.

d Multiplicity approach tests HD and LD simultaneously and separately at $\alpha = 0.0249$. For either dose, if both primaries are rejected, then the first key secondary at the corresponding dose will be tested at $\alpha = 0.0249$.

e It is noted that these sample sizes were computed using the original futility IA criteria prior to Amendment 017-17 (i.e., continue the study if the product of the CPs corresponding to the primaries ≥ 0.25 OR if at least one of the primaries and the key secondary both had a CP > 0.70). Using the revised futility IA criteria, power is slightly (2-3%) higher.

8.2.8.4.2 Secondary Biomarkers and Substudy Endpoints

Given the position of the biomarkers in the multiplicity strategy and the presence of multiple doses, the reporting of power (or the computation of sample size) for any one biomarker is complicated and of limited interpretability. Therefore, the relevant computation (power for THV, sample size for Total Tau and Cortical Amyloid Load) is provided in a marginal sense, ignoring the multiplicity strategy and the fact that multiple doses are present (though an α of 0.0249 will be used). All calculations in this section are based on the same assumed dropout rate as was assumed for the primary endpoint sample size calculations.

THV: Since MRI will be conducted on all subjects, and the sample size for the trial was dictated by the primary endpoints, the marginal power for THV was computed and is equal to 99.9%.

Total tau: The number of randomized subjects from which CSF collection is required for 90% marginal power (at one dose level) is 38 subjects per arm to detect a 35%

reduction towards normal values. However, it is uncertain what a biologically meaningful reduction in tau is. Furthermore, total tau, along with Aβ, strongly correlates with brain amyloid. Subjects who have a tau/Aβ ratio in the normal range have a relatively low probability of having AD and may have another type of dementia. The CSF tau/Aβ ratio can be used in an exploratory fashion to evaluate diagnostic accuracy of AD. Based on these considerations, CSF will be collected at all sites qualified to perform lumbar punctures (estimated at about 50% of sites) from all willing subjects (estimated at 50%).

Cortical Amyloid Load: The number of randomized subjects required to be scanned at baseline to achieve 90% marginal power (at one dose level) is 49 subjects per arm. As it is also uncertain what a biologically meaningful reduction in cortical amyloid load is, up to 100 subjects per arm will be enrolled to participate in the PET substudy.

8.2.9 Interim Analysis

Access to Unblinded Reports

As noted above, the eDMC (and any necessary consultants thereto, e.g. ophthalmologists) will have access to unblinded safety reports throughout the trial. The siDMC will have access to unblinded safety reports based on the first 200 subjects enrolled only. The EOC will not have access to unblinded reports, though the EOC may be unblinded to results at the treatment level if the eDMC recommends modifications to the design of the protocol or recommends the discontinuation of the trial.

Unblinded analyses will be provided for the ADAS-Cog, in order to assess for potential cognitive worsening. For the first formal safety IA (for review by both the eDMC and the siDMC), these reports will be based on the first 200 subjects only. Since the first 200 subjects will be excluded from the primary efficacy analyses, similar analyses on these subjects may be presented to the eDMC and the siDMC at the first formal IA for the ADCS-ADL and the CDR-SB.

The subsequent ongoing safety analyses will be based on data from all available subjects and will include the ADAS-Cog analyses. These subsequent safety analyses (based on all subjects) will ONLY be presented to the eDMC. The siDMC will not play a role in these safety interim analyses and will not review these data.

As the trial progresses the siDMC will periodically review clinical data (eg, ADAS-Cog, ADCS-ADL, CDR-SB) from the first 200 subjects only. Further details are provided in the charters for the eDMC and siDMC.

Blinding to treatment assignment will be maintained at all investigational sites.

Interim Analyses

Three types of interim analyses will be performed during this trial: 1) the first formal analysis for safety and dose selection, 2) multiple ongoing safety analyses and 3) one futility analysis. Trial enrollment will continue during all interim analyses.

The endpoints, timing, and purpose of the interim analysis are summarized in **Table 10**. All interim analyses will be prepared by external personnel (eg, external unblinded statistician).

Table 10 Summary of Interim Analysis Strategy

Type Of Interim Analysis	Key Endpoints for Interim Analysis	Timing of Interim Analysis	Purpose of Interim Analysis
First formal analysis for safety and dose selection	All Safety Endpoints, in addition to analyses on the ADAS-Cog (first 200 subjects only), to inform potential cognitive worsening. A PK/PD assessment of any AE of interest if requested by eDMC or siDMC.	The first 200 subjects have either completed at least 13 weeks of treatment or have discontinued the trial before completing 13 weeks of treatment.	Safety, Dose Selection
Ongoing safety analyses	All Safety Endpoints, in addition to analyses on the ADAS-Cog, to inform potential cognitive worsening.	Additional analyses will be conducted when all remaining members of the Safety Cohort have completed 26, 39, 52 and 78 weeks treatment, respectively. Additional safety analyses will be conducted throughout the trial (Parts I and II) as requested by the eDMC.	Safety
Futility analysis	Change-from-Baseline in ADAS-Cog<br>Change-from-Baseline in ADCS-ADL<br>Change-from-Baseline in CDR-SB	50% of the planned subjects in each treatment arm have either completed 78 weeks of treatment or discontinued before completing 78 weeks of treatment	Assess potential futility of MK-8931 to potentially stop the trial
ADAS-Cog=Alzheimer's Disease Assessment Scale Cognitive subscale; ADCS-ADL=Alzheimer's Disease Cooperative Study Activities of Daily Living Inventory; CDR-SB=Clinical Dementia Rating Sum of Boxes.			

First Formal Interim Analysis for Safety and Dose Selection

An independent eDMC, as well as an siDMC, will assess unblinded 3-month data to determine if the trial should continue. At this first IA, both the siDMC and the independent eDMC will assess unblinded 3-month data from Subjects 1-200, with the eDMC also having access to safety data from the remaining ~200 Safety Cohort subjects.

This first formal interim analysis will include standard safety analyses, including Clinical and Laboratory AE counts and percentages, as well as change-from-baseline summary statistics for labs, ECGs, and vital signs.

Further, exposure estimates (AUC, Cmax) for the subjects in the Safety Cohort will be provided based on the concentration data and the previously developed population PK model for MK-8931. The eDMC and siDMC will also have the option to request a PK/PD assessment of any AE of interest to better assess the association with exposure.

Basic summary statistics and slope analyses will be provided for the ADAS-Cog, in order to assess for potential cognitive worsening. For this first formal safety IA (for review by both the eDMC and the siDMC), this analysis will be based on the first 200 subjects only. Since the first 200 subjects will be excluded from the primary efficacy analyses, similar analyses on these subjects may be presented to the eDMC and the siDMC at the first formal IA for the ADCS-ADL and the CDR-SB.

Should it be decided that the trial will continue, the siDMC will provide a recommendation as to whether both the 12 mg and 40 mg doses should continue into the Main Cohort as planned, or whether only the 12 mg dose should continue. This recommendation will be provided in writing to the eDMC.

The eDMC will provide a recommendation as to which doses are sufficiently safe to continue into the Main Cohort, and based on the recommendations from the siDMC, which dose(s) should continue on into the Main Cohort. The 12 mg and/or the 40 mg doses may move into the Main Cohort (the 60 mg dose will not continue into the Main Cohort).

The eDMC will also make recommendations to the sponsor regarding the potential cessation of the additional safety measures implemented for the Safety Cohort (ie, the extra visit at Week 8 and the extra telephone visits at Weeks 6 and 10).

The standard safety analyses will be analyzed by the external unblinded statistician. The PK analysis will be conducted by an external modeling group, including data set assembly and evaluation, model applications, exposure estimation and optional PK/AE assessment.

Additional details will be specified in the eDMC and siDMC Charters.

Confidential

Ongoing Safety Analyses

The independent eDMC will also evaluate unblinded safety analyses of all trial subjects every 4 to 6 months. Additional safety analyses may be conducted throughout the trial as requested by the eDMC.

These ongoing safety analyses will also consist of unblinded reports for the ADAS-Cog, to assess for potential cognitive worsening and will be based on data from all subjects. The sole purpose for including these reports on the ADAS-Cog is to monitor for safety, with no intent for stopping the trial due to overwhelming efficacy. However, a small amount of α (=.0001) has been allocated to each of these safety analyses, for statistical rigor.

Futility Analyses

One interim efficacy analysis will be performed when the first 50% of the planned subjects in the remaining treatment arms have either completed 78 weeks of treatment or have discontinued before completing 78 weeks of treatment. The main purpose of this interim analysis is to assess futility, with no intent for stopping the trial due to overwhelming efficacy. However, a small amount of α (=.0001) has been allocated, primarily for statistical rigor. The external unblinded statistician will conduct the unblinded interim analysis and will present the results to the eDMC (who will then make their recommendations to the EOC).

The decision to stop the trial early for futility will be based on the conditional power, ie, the probability of detecting a treatment difference at the end of the trial given the interim results (assuming that the underlying true parameter means are equal to the corresponding observed point estimates at the time of the interim analysis, ie, assuming that the current trend is true).

At the interim analysis, the conditional power (CP) will be independently computed for ADAS-Cog, ADCS-ADL, and CDR-SB at each of the two MK-8931 doses. Consideration should be given to terminating the trial for futility if both of the following within-dose conditions hold for all remaining doses:

Criteria 1) The product of the CPs corresponding to ADAS-Cog and ADCS-ADL is less than 5%.

Criteria 2) The minimum of (MaxPrimCP, CP for CDR-SB) is less than 5%, where MaxPrimCP is defined as the maximum of the CPs corresponding to ADAS-Cog and ADCS-ADL.

In other words, the trial should be allowed to continue if either 1) the probability that both primaries will be supported is greater than 5% or 2) the probability that at least of one the primaries in addition to the key secondary will be supported is greater than 5% .

It is also noted that the probability of terminating the trial at the futility IA (ie, failing to meet the minimum requirements set by the above futility criteria), under the assumptions used to calculate the sample size is <1% (ie, $\beta < 0.01$).

The usefulness of the futility criteria may be assessed by examining the probability of terminating the trial in the presence of a non-meaningful drug effect. Assuming a 10% drug effect for both doses (thought to not be clinically meaningful), results in a 36% probability of terminating the trial at the futility IA.

Table 11 contains the observed treatment differences which must be obtained (assuming true underlying variances consistent with **Table 6**) to achieve various conditional powers of interest.

Table 11 Observed Treatment Differences Required to Obtain Various Marginal Conditional Powers

Conditional Power for Given Endpoint	Observed Treatment Difference Required at Interim Analysis to Obtain Given Conditional Power†		
	ADAS-Cog	ADCS-ADL	CDR-SB
5%	0.70	0.97	0.23
10%	0.86	1.20	0.29
20%	1.06	1.48	0.36
30%	1.21	-1.68	0.41
40%	1.33	-1.85	0.45
50%	1.44	-2.01	0.49
60%	1.56	-2.17	0.50
70%	1.68	-2.34	0.57
80%	1.83	-2.54	0.61

†Conditional power computed using 200 subjects per arm (assuming a 30% dropout rate), assuming observed variances and correlations as given in Table 6, and assuming that the underlying treatment effect is equal to the observed treatment effect at the interim (ie, assuming the current trend). Conditional power refers to the probability that the final nominal p-value for the given endpoint will be less than 0.0249.

The trial is powered assuming true underlying 18-month drug effects of 2.14, -3.37, and 0.84 for ADAS-Cog, ADCS-ADL, and CDR-SB, respectively.

8.2.10 Accounting for Missing Data

Common reasons for discontinuation from the trial may include lack of efficacy, clinical or laboratory adverse experiences, relocation, withdrawal of consent, protocol violations, and/or data processing issues. Missing data caused by relocation and data processing issues are likely to be missing-completely-at-random (MCAR). On the other hand, missing data caused by discontinuation due to lack of efficacy may be missing-at-random (MAR) because the discontinuation may depend on the observed efficacy outcomes. The MAR or missing-not-at-random (MNAR) mechanisms might each underlie the other reasons to some extent. If treatment in large part determines the loss of data for these other reasons (such as clinical or laboratory adverse experiences), the mechanism may be close to MAR because treatment assignment is an observed variable and included in the analysis model. Based on prior trial results, missing data due to other reasons is relatively infrequent.

In order to better adhere to the intent-to-treat analysis principle, an attempt will be made to collect ADAS-Cog, ADCS-ADL, MMSE, and CDR-SB from subjects who discontinue trial medication prior to the end of the trial (78 weeks).

No explicit imputation of missing data (beyond the limited imputation performed within an assessment) will be done for the primary analysis approach.

A pattern-mixture model based on the tipping-point approach will be used to assess the robustness of the primary analysis approach. For a given constant, *c*, the tipping point analysis is conducted in a fashion similar to that used in standard multiple imputation (MI) [25,26,27], whereby *m* complete datasets are randomly generated using the original observed dataset. These *m* complete datasets are subsequently analyzed using the primary model, and the results of those analyses are then combined. The construction and analysis of these *m* (=50) datasets requires four primary steps:

1) Using a Markov Chain Monte Carlo method[28], make the observed dataset monotone-missing. This will be accomplished for each treatment group using "proc mi" within SAS 9.3 by utilizing the options "mcmc chain=multiple impute=monotone;", in conjunction with all of the covariates (excluding treatment) included in the primary analysis model. The random seed will be set equal to 8931017. This step will generate *m* monotone-missing datasets.

2) Applying parametric regression to the monotone-missing datasets, impute the missing values in a stepwise fashion starting with the first postdose timepoint. This will be accomplished for each treatment group using "proc mi" within SAS 9.3 utilizing the option "monotone reg", in conjunction with all of the covariates (excluding treatment) included in the primary analysis model. The random seed will be set equal to 8931017. This step will generate *m* complete datasets.

3) To implement the tipping-point aspect of the procedure, subtract a constant *c* from each of the imputed values of the active arms (to the detriment of active).

4) Analyze each of the post-imputation complete datasets using the primary model, obtaining point estimates for the mean of interest (eg, change-from-baseline treatment difference at 104 weeks) and the associated variance.

Using "proc mianalyze" within SAS 9.3, the *m*=50 means and variances from the *m* analyses will be combined to obtain the final test statistic and p-value[25]. The final test statistic . / ($T^{-(1/2)}$) is approximately distributed as t_v, where is the sample mean of the *m* mean estimates, T= + (*m+1) (B/m*), is the sample mean of the *m* variance estimates, and *B* is the sample variance of the *m* mean estimates. The degrees of freedom, v, will be computed as follows[29], $v = [(v_1)^{-1} + (v_2)^{-1}]^{-1}$, where $v_1 = (m\text{-}1)\ [\ 1 + (\ \ /(1+m^{-1})\ B)]^2$ and $v_2 = (1\text{-}\gamma)\ v_0\ (v_0 + 1)\ /\ (v_0 + 3)$, with $\gamma = (1+m^{-1})\ B\ /\ T$ and where v_0 represents the complete-data degrees of freedom .

This procedure will be repeated (using the same *m* imputed datasets) until the smallest *c* is found such that the significant result turns non-significant (ie, $p \geq 0.02495$). This tipping point value *c* provides a measure of robustness of the primary result. A relatively large value of *c* implies better robustness of the primary analysis against the impact of missing data in the study.

It is noted that when $c=0$ the tipping point analysis described above corresponds to an analysis conducted under the assumption that the missing data are MAR. For values of *c* larger than 0, the tipping point analyses do not assume that the missing values follow a MAR mechanism. In fact, the analysis is based on a special MNAR mechanism in which all missing data in the active arm are assumed to have a worse response by a constant amount of *c* than the values would have had under MAR, while the missing data in the control group are assumed to be the same as that obtained under MAR.

It may be necessary to adjust *c*, in order to calibrate between the two analysis approaches (ANCOVA vs. MI), should a value of c=0 yield a different p-value than is produced from the primary ANCOVA analysis model. This will be accomplished by subtracting the calibration value c_{prim} from *c*, where c_{prim} is the offset to be applied to the active arm, per the above MI approach, that will yield the same p-value as prodcued by the primary ANCOVA analysis.

<u>Handling of Missing Items Within a Clinical Assessment</u>

The final scores of the ADAS-Cog, ADCS-ADL, NPI, MMSE, and CDR-SB are all constructed from multiple subquestions within each assessment. It is possible that one or more subquestions may be missing within each assessment. In this event, the last recorded score for this subquestion(s) may be carried forward from the most recent postdose visit (Last Observation Carried Forward approach). In a similar fashion, missing baseline values may be carried over from the most recent screening visit, if available. Baseline/screening values will never be carried forward to impute missing postdose values. Due to the degenerative properties of AD, with subjects expected to worsen over time, an individual subquestion will not be carried forward for more than one visit. If the same subquestion is missing two visits in a row, then LOCF will be applied to the first missing visit and the subquestion will remain as missing for the second visit (with the total score to then be computed as missing). Further, the total score will be computed as missing if too many subquestions, prior to applying the LOCF approach, are missing (see endpoint-specific details below). This single imputation approach allows the total score to be calculated using the strength of the other subquestions collected at that time, for that subject. The Sponsor believes this approach to be more accurate than either setting the entire score to missing or to imputing the worst possible score. More complicated missing data approaches are not thought to be warranted, since the amount of missing data within an assessment is expected to be extremely low.

For Baseline ADCS-ADL only: Past experience indicates that some subquestions on the ADCS-ADL may mistakenly be omitted at the time of administration. Should this occur at baseline, there will be no opportunity to employ the stated LOCF approach. To avoid a missing total score on the ADCS-ADL (and the subsequent removal of the subject from the primary ADCS-ADL population), the worst possible score for that subquestion will be imputed. Note that this imputation approach will only be implemented for ADCS-ADL (given the relatively small impact of the individual subquestions on the overall score) and only at baseline. A total score will only be computed if the number of missing subtotals is strictly less than three.

Endpoint specific details are as follows:

ADAS-Cog and MMSE: The site will be instructed to enter the worst possible score if the subject is unwilling or unable to answer a subquestion due to reasons related to the area the subquestion is trying to address. If the subject is unable to answer a subquestion for some other reason, then the site will be instructed to leave the subquestion as missing. For both the ADAS-Cog and the MMSE, a total score will be calculated if the original number (prior to applying LOCF) of missing subtotals is strictly less than three.

CDR-SB, ADCS-ADL, and NPI: The CDR-SB, ADCS-ADL, and NPI are all administered to the caregiver, not the subject, so no within-assessment missing data are expected (though missing subquestion data is still possible due to data entry error or errors in test administration). For ADCS-ADL and NPI, a total score will be calculated if the original number of missing subtotals is strictly less than three. For CDR-SB, a total score will be calculated if the original number of missing subtotals is strictly less than two.

8.3 Safety

Subjects from both cohorts will be included in the safety analyses, though the first 200 subjects enrolled will be excluded from the primary safety analyses. Supportive safety analyses in which these subjects are included will be conducted.

Subjects enrolled in a dose that is dropped during the trial will be excluded from the primary safety analyses. Supportive analyses in which these subjects are included will be conducted by treatment sequence, with a treatment sequence possibly including more than one active dose of MK-8931. For example, the treatment sequence would be "MK-8931 60 mg / MK-8931 40 mg" in the event that the MK-8931 60 mg dose is dropped, with all subjects initially randomized to the MK-8931 60 mg group assigned to receive MK-0931 40 mg for the remainder of the trial.

Safety and tolerability will be assessed by a clinical review of all relevant parameters including AEs, laboratory tests, vital signs, and ECG measurements. The primary approach to analyzing clinical AEs will only include those AEs occurring within 14 days of trial medication administration. A supportive approach will include all AEs reported within 14 days of the subject's last visit (including AEs reported from those subjects who have stopped taking trial medication).

The analysis of safety results will follow a tiered approach (Table 11). The tiers differ with respect to the analyses that will be performed. Safety parameters or AEs of special interest that are identified a priori constitute "Tier 1" safety endpoints that will be subject to inferential testing for statistical significance with P-values and 95% CIs provided for between-group comparisons (active vs. placebo). All other safety parameters will be considered Tier 2 or Tier 3 safety endpoints. Tier 2 safety endpoints will be assessed via point estimates with 95% CIs provided for between-group comparisons. Tier 3 safety endpoints will be summarized using only point estimates by treatment group.

Adverse events (specific terms as well as system organ class [SOC] terms) and predefined limits of change (PDLC) in laboratory, vital signs, and ECG parameters that are not prespecified as Tier 1 endpoints will be classified as belonging to "Tier 2" or "Tier 3", based on the number of events observed. Membership in Tier 2 requires that at least 1% of subjects in any treatment group exhibit the event; all other AEs and PDLCs will belong to Tier 3.

It is noted that the 95% CI for the between-group difference in percent incidence will always include zero when treatment groups of equal size each have less than four events and thus would add little to the interpretation of potentially meaningful differences. The threshold of "at least 1%" was chosen because the expected number of subjects in the APaT population is greater than 400, thus an incidence of at least 1% in a treatment group translates to at least four subjects experiencing that event in the treatment group. Because many 95% CIs may be provided without adjustment for multiplicity, the CIs should be regarded as a helpful descriptive measure to be used in review, not a formal method for assessing the statistical significance of the between-group differences in AEs and PDLCs.

Continuous measures such as changes from Baseline in laboratory parameters, vital signs, and ECG parameters that are not pre-specified as Tier 1 endpoints will be considered Tier 3 safety parameters. Summary statistics for Baseline, on-treatment, and change-from-Baseline values will be provided by treatment group in table format. A similar approach will be taken for continuous ophthalmic measures such as percent change from baseline in RPE thickness. Proportions of subjects in each category will be provided for semi-quantitative ophthalmic measures such as change from baseline in area of drusen, iris pigmentation, thinning of the outer nuclear layer, and changes from a normal area of normal autofluoresence to an abnormal area of autoflouresence.

P-values (Tier 1 only) and 95% CIs (Tier 1 and Tier 2) will be provided for between-treatment differences in the percentage of subjects with events; these analyses will be performed using the Miettinen and Nurminen method, an unconditional asymptotic method.

A composite endpoint of hypopigmentation adverse events is defined based on the following preferred AE terms or equivalent: (noting that MedDRA terms are subject to change over time): skin hypopigmentation, skin depigmentation, vitiligo, leukoderma, hypopigmentation of eyelid, and idiopathic guttate hypomelanosis . This composite AE of hypopigmentation will be considered as either a Tier 2 or Tier 3 AE, per the criteria for Tier 2 AEs defined above.

Table 11 Analysis Strategy for Safety Parameters

Safety Tier	Safety Endpoint	P-Value	95% CI for Treatment Comparison	Descriptive Statistics
Tier 1	Incident microhemorrhage, superficial siderosis or macrohemorrhage in brain MRI scans	X	X	X
	Incident vasogenic edema	X	X	X
	Delirium	X	X	X
	Rash ECI	X	X	X
Tier 2	Any AE[a]		X	X
	Any Serious AE		X	X
	Any Fatal AE		X	X
	Any Drug-Related AE		X	X
	Any Serious and Drug-Related AE		X	X
	Discontinuation due to AE		X	X
	Specific AEs, SOCs, or PDLC (incidence ≥ 1% in one of the treatment groups)		X	X
Tier 3	Specific AEs, SOCs or PDLC (incidence < 1% in all of the treatment groups)			X
	Change from Baseline Results (Labs, ECGs, Vital Signs)			X

AE=adverse event; CI=confidence interval; ECG=electrocardiogram; PDLC=predefined limit of change; SOC=system organ class; X=results will be provided.

a Adverse experience references refer to both clinical and laboratory AEs. Includes only those endpoints not prespecified as Tier 1 or not already prespecified as Tier 2 endpoints.

8.3.1 Analysis of Prespecified Safety Endpoints

The Prespecified Safety Endpoints of 1) microhemorrhage, superficial siderosis or macrohemorrhage in brain MRI scans, 2) vasogenic edema, 3) delirium, and 4) rash ECI are considered as Tier 1 events and will be analyzed as indicated in Table 11.

8.3.2 Analysis of Commonly Occurring Safety Endpoints

The broad clinical and laboratory AE categories consisting of the percentage of subjects with any AE, with a drug-related AE, with an SAE, with an AE which is both drug-related and serious, or who discontinued because of an AE are considered as Tier 2 endpoints and will be analyzed as indicated in Table 11.

8.3.3 Analysis of Descriptive Safety Endpoints

Descriptive Safety Endpoints are considered as Tier 3 events and will be analyzed as indicated in Table 11.

8.4 Trial Medication Compliance and Exposure

8.4.1 Compliance

As part of the routine recording of the amount of trial treatment taken by each subject, the number of pills remaining in trial packaging will be counted and recorded at regular intervals. These results will be used to calculate subject compliance. Instances where the subject took more or less trial medication than prescribed will be recorded on the study medication eCRF. Events that meet criteria for overdose as defined in Section 7.7.2.2.4 will also be reported as an AE (ECI or SAE, as applicable). To monitor for potential misuse, the site will document significant discrepancies in drug returns where the subject or trial partner returns less trial medication than expected but denies taking extra trial medication (eg, lost or missing medication equivalent to more than one pill per week).

A day within the trial will be considered an "On-Therapy" day if the subject doses on that day. For a subject who is followed for the entire trial period, the "Number of Days Should be on Therapy" is the total number of days from the first dose to the last scheduled day for treatment administration for that subject. For a subject who permanently discontinued trial medication, the "Number of Days Should be on Therapy" is the total number of days from the first dose to the last dose of trial medication.

For each subject, percent compliance will be calculated as 100 times the number of days 'On Therapy" divided by the "Number of Days Should be on Therapy". The average compliance overall, and over each 3-month time frame, will be calculated by treatment group. The percent of subjects who meet various compliance thresholds (eg, 75%, 95% compliant) will also be calculated overall, and over each 3-month time frame, by treatment group. The FAS population will be used for all trial medication compliance calculations.

8.4.2 Exposure

Basic summary statistics for the number of doses of trial medication taken will be calculated overall, and as well as over each 3-month time frame by treatment group. The cumulative percent of subjects taking various numbers of doses of trial medication (eg, one dose, 30 doses) will be calculated by treatment group. The APaT population will be used for all trial medication exposure calculations.

8.5 Demography

Basic summary statistics (means, standard deviations, counts, percentages) will be provided, as applicable, by treatment group, for subject baseline characteristics, subject disposition, and prior and concomitant medication usage.

9.0 ADHERENCE TO ETHICAL, REGULATORY, AND ADMINISTRATIVE CONSIDERATIONS

The trial (Part I and Part II) must be conducted in accordance with Good Clinical Practice (GCP) as outlined in the International Conference on Harmonisation of Technical Requirements for Registration of Pharmaceuticals for Human Use (ICH) Guidelines, E6 Good Clinical Practice: Consolidated Guidance and other applicable laws and regulations. In addition, the trial must be conducted in accordance with: (i) the USA Code of Federal Regulations (CFR) if the trial is conducted under a USA IND, regardless of the country involved; (ii) the European Union (EU) Clinical Trial Directive (CTD) and local regulations if the trial is conducted in the EU; and (iii) any specific local regulations if the trial is conducted elsewhere.

9.1 Ethical Conduct of the Trial

9.1.1 Independent Ethics Committee or Institutional Review Board

Prior to initiation of the trial at any site, the trial, including the protocol, informed consent, and other trial documents must be approved by an appropriate Institutional Review Board (IRB) or Independent Ethics Committee (IEC). The IRB/IEC must be constituted according to applicable regulatory requirements. As appropriate, amendments to the protocol must also be approved by the IRBs/IECs before implementation at the sites, unless warranted to eliminate an immediate hazard. The IRB/IEC approval should be obtained in writing, clearly identifying the trial, the documents reviewed (including informed consent), and the date of the review. The trial as described in the protocol (or amendment), informed consent, and other trial documentation may be implemented only after all the necessary approvals have been obtained and the sponsor has confirmed that it is acceptable for the investigator to do so.

In the event that the IRB/IEC requires changes in the protocol, the sponsor shall be advised and must approve the changes prior to implementation. The investigator shall not modify the trial described in the protocol once finalized and after approval by the IRB/IEC without the prior written approval of sponsor.

In countries where the investigator submits the trial protocol and statement of informed consent to the IRB/IEC, the investigator or qualified designee will forward the approvals to the sponsor.

9.1.2 Subject Information and Consent

The details of the protocol must be provided in written format and discussed with each potential subject, and written informed consent must be obtained for all subjects before any trial-related procedure is performed. Informed consent will be obtained separately for Part I and Part II. NOTE subject participation in Part II is

optional and will only be conducted where approved by local authorities. In addition, separate informed consents will be obtained for the Biomarker substudies. In obtaining informed consent, the information must be provided in language and terms understandable to the subject. The subject, or the subject's legal representative, must give their written consent to participate in the trial. The signed and dated consent form itself must be retained by the investigator as part of the trial records. A copy of the signed and dated consent forms must be given to the subject. The consent forms must include all of the required elements of informed consent in accordance with ICH Guidelines E6 and local laws. In addition, the sponsor specifically requests that the consent forms identify it as the sponsor and state that use of the investigational product(s) is experimental and the side effects of the investigational product(s) are not completely known. The consent forms must be approved by the appropriate IRB/IEC and sponsor before trial initiation at a trial site. Any subsequent changes to the approved informed consent forms must be reviewed and approved by the appropriate IRB/IEC and sponsor before implementation.

9.1.3 Subject Identification Card

All subjects will be given a Subject Identification Card identifying them as participants in a research trial. The card will contain trial site contact information (including direct telephone numbers) to be utilized in the event of an emergency. The investigator or qualified designee will provide the subject with a Subject Identification Card after the subject provides written informed consent. The card is to be shown to caregivers in the event of an emergency.

At a minimum, the card must contain the following information:

1. Protocol number;
2. The subject's protocol identification number;
3. A statement identifying the card-carrier as a participant in a clinical trial (eg, "This person is participating in a clinical research trial.");
4. A statement indicating the person might be taking an investigational drug (eg, "This person is taking an experimental drug which could have interactions with other medications, or placebo"); and
5. Contact information in the event of an emergency or hospitalization. The contact information on the card is to be the investigator or a designated site contact, rather than contact from within the sponsor;

The cards may also include other trial-specific information to assist with treatment decisions in the event of an emergency, such as types of concomitant therapies that may, or may not be, permitted as part of emergency treatment. As with any other information provided to subjects, the Subject Identification Card must be approved by the IRB/IEC. Monitors will request that Investigators provide Subject Identification Cards to each subject. Investigators will be asked to request that subjects carry the cards with them while they are participating in the trial.

9.1.4 Registration of the Trial

The trial will be registered by the sponsor on a publicly accessible database. The results will be disclosed by the sponsor on a publicly accessible database.

9.2 Reporting Trial Data to the Sponsor

9.2.1 Data Collection Forms

The Sponsor will provide the site with data collection forms, be they Case Report Forms (CRF), either in paper format or electronic Case Report Forms (eCRF); diaries; Electronic Data Capture (EDC) screens; or other appropriate data collection forms as the trial requires. The investigator is to provide subject data according to the Sponsor's instructions, in the designated data collection form, compliant with GCP practices. The Sponsor will also provide the site with instructions for assisting other parties - such as a central laboratory - to collect data. As instructed by the Sponsor, a designated central laboratory may collect data in a database and provide the completed database to sponsor. All data collection forms and the databases from the trial are the exclusive property of sponsor.

The investigator must maintain records and data during the trial in compliance with all applicable legal and regulatory requirements. Each data point must be supported by a source document at the trial site. Any records or documents used as the source of information (called the "subject source data") are to be retained for review by authorized representatives of the sponsor or a regulatory agency.

The investigator will ensure that there are sufficient time, staff, and facilities available for the duration of the trial to conduct and record the trial as described in the protocol and according to all applicable guidances, laws, and regulations.

All data collection forms (eg, CRFs, diaries; EDC screens), electronic database entries, etc, should be completed as soon as possible after the evaluation has occurred. All dates appearing on the sponsor's subject data collection forms for laboratory tests, cultures, and other data collected, must be the dates on which the specimens were obtained, or the procedures performed.

9.2.2 Preparing Case Report Forms for All Subjects

A CRF must be completed for all subjects who have given informed consent. The Sponsor must not collect subject names, initials, or other personal information that is beyond the scope of the trial from any subject. Subjects are not to be identified by name or initials on the CRF or any trial documents. The only acceptable identification for a subject who may appear on a CRF or trial document is the unique subject identification number. The investigator must maintain contact information for each participant so that all can be quickly contacted by the investigator, if necessary.

All entries into CRFs are the responsibility of the investigator and must be completed by the investigator or a qualified designee. Through signing the Investigator Signature Page of the protocol the investigator acknowledges that his/her electronic signature is the legally binding equivalent of a written signature. By entering his/her electronic signature, the investigator confirms that all recorded data have been verified as accurate.

9.2.3 Preparing Case Report Forms for Subjects Who Fail Screening

Data are to be collected from the time the informed consent form is signed until the subject is determined to have failed screening. A CRF with a minimum of the following information must be completed for subjects who fail screening: (1) demographics, (2) subject status, (3) reason for screen failure, (4) serious adverse events, and (5) MMSE.

9.3 Publications and Other Rights

9.3.1 Rights to Publish by the Investigator

The investigator has the right to publish or publicly present the results of the trial in accordance with this Section 9.3 of the protocol. In the event that the protocol is a part of a multi-site trial, it is understood that it is the intent of the sponsor and the investigator to initially only publish or present the trial results together with the other sites, unless specific written permission is obtained in advance from the sponsor to publish separate results. The sponsor shall advise as to the implications of timing of any publication in the event clinical trials are still in progress at sites other than the investigator's site.

The investigator agrees not to publish or publicly present any interim results of the trial without the prior written consent of the sponsor. The investigator further agrees to provide to the sponsor 45 days prior to submission for publication or presentation, review copies of abstracts or manuscripts for publication (including, without limitation, slides and texts of oral or other public presentations and texts of any transmission through any electronic media, eg, any computer access system such as the Internet, World Wide Web, etc) that report any results of the trial. The sponsor shall have the right to review and comment with respect to publications, abstracts, slides, and manuscripts and the right to review and comment on the data analysis and presentation with regard to the following concerns:

1. Proprietary information that is protected by the provisions contained in **Section 9.3.2**;
2. The accuracy of the information contained in the publication; and
3. To ensure that the presentation is fairly balanced and in compliance with US FDA regulations.

If the parties disagree concerning the appropriateness of the data analysis and presentation, and/or confidentiality of the sponsor's confidential information, investigator agrees to meet with the sponsor's representatives at the clinical trial site or as otherwise agreed, prior to submission for publication, for the purpose of making good faith efforts to discuss and resolve any such issues or disagreement.

9.3.2 Use of Proprietary or Confidential Information in a Publication

No publication or manuscript shall contain any trade secret information of the sponsor or any proprietary or confidential information of the sponsor and shall be confined to new discoveries and interpretations of scientific fact. If the sponsor believes there is patentable subject matter contained in any publication or manuscript submitted for review, the sponsor shall promptly identify such subject matter to investigator. If sponsor requests and at sponsor’s expense, investigator shall use its best efforts to assist sponsor to file a patent application covering such subject matter with the USA Patent and Trademark Office or through the Patent Cooperation Treaty prior to any publication.

9.3.3 Use of Trial Information in a Publication

Investigator is granted the right subject to the provisions of this protocol to use the results of all work provided by investigator under this protocol, including but not limited to, the results of tests and any raw data and statistical data generated for investigator's own teaching, research, and publication purposes only. Investigator/Institution agrees, on behalf of itself and its employees, officers, trustees, and agents, not to cause said results to be knowingly used for any commercial purpose whatsoever except as authorized by the sponsor in writing.

9.3.4 Authorship of Publications

Authors of publications must meet the International Committee of Medical Journal Editors (ICMJE) guidelines for authorship and must satisfy the 3 criteria that follow:

1. Authors must make substantial contributions to the conception and design of the trial, acquisition of data, or analysis of data and interpretation of results;
2. Authors must draft the publication or, during draft review, provide contributions (data analysis, interpretation, or other important intellectual content) leading to significant revision of the manuscript with agreement by the other authors;
3. Authors must provide written approval of the final draft version of the publication prior to submission.

All contributors who do not meet the 3 criteria for authorship should be listed in an acknowledgments section within the publication, if allowed by the journal, per the ICMJE guidelines for acknowledgment.

9.4 Trial Documents and Records Retention

During the trial and after termination of the trial – including after early termination of the trial – the investigator must maintain copies of all documents and records relating to the conduct of the trial. This documentation includes, but is not limited to, protocols, CRFs and other data collection forms, advertising for subject participation, adverse event reports, subject source data, correspondence with health authorities and IRBs/IECs, consent forms, investigator's curricula vitae/biosketch, monitor visit logs, laboratory reference ranges, and laboratory certification or quality control procedures and laboratory director curriculum vitae. Subject files and other source data must be kept for the maximum period of time permitted by the hospital, institution or private practice, or as specified below. The sponsor must be consulted if the investigator wishes to assign the files to someone else, remove them to another location, or is unable to retain them for the specified period.

The investigator must retain trial records for the amount of time specified by applicable laws and regulations. At a minimum, trial records must be retained for the amount of time specified by ICH Guidelines, the EU Good Clinical Practices Directive, or applicable local laws, whichever is longer:

1. The ICH Guidelines specify that records must be retained for a minimum of 2 years after a marketing application for the indication is approved (or not approved) or 2 years after notifying the appropriate regulatory agency that an investigation is discontinued.
2. The European Union (EU) Commission Directive 2003/63/EC which requires that Essential Documents (including Case Report Forms) other than subjects' medical files, are retained for at least fifteen (15) years after completion or discontinuation of the trial, as defined in the protocol.

All trial documents shall be made available if required by relevant health authorities. The investigator should consult with the sponsor prior to discarding trial and/or subject files.

Sponsor will retain all sponsor-required documentation pertaining to the trial for the lifetime of the investigational product. Archived data may be held on microfiche or electronic record, provided that a back-up exists and that a paper copy can be obtained from it, if required.

10.0 INVESTIGATORS AND TRIAL ADMINISTRATIVE STRUCTURE

This section applies to Part I and Part II.

10.1 Sponsor

The sponsor of this trial is indicated in Section 1, Title Page.

10.2 Investigators

10.2.1 Selecting Investigators

Only investigators qualified by training and experience to perform a clinical investigation with MK-8931 are selected. The sponsor will contact and select all investigators (ie, the legally responsible party[ies] at each trial site), who, in turn, will select their staff.

10.2.2 Financial Disclosure Requirement

In connection with the clinical trial described in the protocol, the investigator certifies that, if asked, the investigator will read and answer the Certification/Disclosure Form or equivalent document truthfully and to the best of investigator's ability. Investigator also certifies that, if asked, the investigator will have any other applicable party(s) (eg, subinvestigators) read and answer the Certification/Disclosure Form as a condition of their participation in the trial.

If the financial interests reported on the Certification/Disclosure Form change during the course of the trial or within 1 year after the last subject has completed the trial as specified in the protocol, the investigator and the other applicable party(s) are obligated to inform the sponsor of such financial change.

10.2.3 Clinical Study Report Coordinator Investigator

A Clinical Study Report (CSR) will be prepared by the sponsor or its qualified designee to describe the results of the trial. One of the investigators shall be selected by the sponsor to review the CSR and provide approval of the final CSR in writing. The investigator chosen to review and approve the CSR is to be called the CSR Coordinating Investigator. A second investigator shall be selected as the Alternate CSR Coordinating Investigator. The Alternate CSR Coordinating Investigator is to review and approve the CSR should the first CSR Coordinating Investigator be unable to do so. The sponsor is to select the CSR Coordinating Investigator and Alternate CSR Coordinating Investigator from the investigators using the following criteria:

1. Must be the Principal Investigator at a trial site actively enrolling subjects and participating in the trial;
2. Must be willing and capable of completing the necessary reviews and providing approval of the CSR in writing;

10.3 Central Organizations

Central organizations to be used in the conduct, monitoring and/or evaluation of this trial are provided on the Contact List.

10.3.1 Scientific Advisory Committee

This trial was developed in collaboration with a Scientific Advisory Committee (SAC). The SAC comprises both Sponsor and non-Sponsor scientific experts who provide input with respect to trial design, interpretation of trial results and subsequent peer-reviewed scientific publications.

10.3.2 Executive Oversight Committee

The EOC is comprised of members of Sponsor Senior Management. The EOC will receive and decide upon any recommendations made by the eDMC and siDMC regarding the trial.

10.3.3 Data Monitoring Committee

To supplement the routine trial monitoring outlined in this protocol, an eDMC will monitor the interim data from this trial. The voting members of the committee are external to the Sponsor. The members of the eDMC must not be involved with the trial in any other way (eg, they cannot be trial investigators) and must have no competing interests that could affect their roles with respect to the trial.

Confidential

The eDMC will make recommendations to the EOC regarding steps to ensure both subject safety and the continued ethical integrity of the trial. Also, the eDMC will review interim trial results, consider the overall risk and benefit to trial participants (see **Section 8.2.9** - Interim Analyses) and recommend to the EOC if the trial should continue in accordance with the protocol.

Specific details regarding responsibilities and governance, including the roles and responsibilities of the various members and the Sponsor protocol team; meeting facilitation; the trial governance structure; and requirements for and proper documentation of eDMC reports, minutes, and recommendations will be described in a separate charter that is reviewed and approved by the eDMC. The eDMC will monitor the trial at an appropriate frequency, as described in the detailed eDMC charter.

To supplement the routine monitoring outlined in this protocol, the siDMC of the Sponsor will receive unblinded analyses for the first 200 subjects only. The siDMC is comprised of members of Sponsor Senior Management, none of whom are directly associated with the conduct of this trial. The siDMC will be primarily responsible for the recommendation to the eDMC to continue the trial after the first safety IA, thus opening up enrollment to the remainder of sites and subjects (Main Cohort), considering the overall risk and benefit to trial participants. Specific details regarding responsibilities of the siDMC will be described in a separate charter that is reviewed and approved by the siDMC.

11.0 TRIAL EXTENSION (PART II)

11.1 EXTENSION DESIGN

A long-term extension to the initial 78-week period (Part I) is included to allow all subjects who completed Part I the option of continued treatment with MK-8931. NOTE, subject participation in Part II is optional and will only be conducted where approved by local authorities. Subjects who did not complete Part I of the study will not be permitted to continue in Part II. In addition, subjects who are less than 75% compliant with trial medication in Part I will not be permitted to continue in Part II, except in special circumstances, which will require Sponsor approval. The extension will continue until MK-8931 becomes commercially available or MK-8931 is proven to be inefficacious or unsafe. It is expected that Part II will have a maximal duration of up to approximately 260 weeks (5 years) for the first subject enrolled. Subjects will continue in one of three treatment arms:

1. Subjects who received MK-8931 12mg in Part I will continue to receive 12mg in Part II.

2. Subjects who received MK-8931 40mg in Part I will continue to receive 40mg in Part II.

3. Subjects who received placebo in Part I will receive MK-8931 40mg in Part II.

Specific procedures to be performed during the extension, as well as their prescribed times and associated visit windows, are outlined in the Extension Flow Chart - Section 11.1.2. Details of each procedure are provided in Section 11.6.5– Trial Procedures.

In an effort to maintain quality control of the clinical ratings, this trial will continue to include a review of the ratings by outside expert(s).

Raters will be asked to audio record interviews and ratings through Week 104. Some or all of these recorded interviews will be reviewed by outside experts. Raters will be provided feedback on the quality of their interviews and ratings by the outside experts by e-mail, telephone or in meetings in order to develop and maintain good rater reliability. Based on this feedback, raters may change their initially recorded scores if errors are identified. Raters who do not perform adequately may be required to undergo additional remediation or may be replaced.

Subjects may participate in this protocol and continue to participate in certain observational studies , if approved by the Sponsor. These studies must involve only limited cognitive testing (eg, annual) and **subjects will not be permitted to undergo any non-protocol cognitive testing within 2 months prior to Week 104**.

Confidential

11.1.1 Extension Diagram

The trial design is depicted in Figure 2.

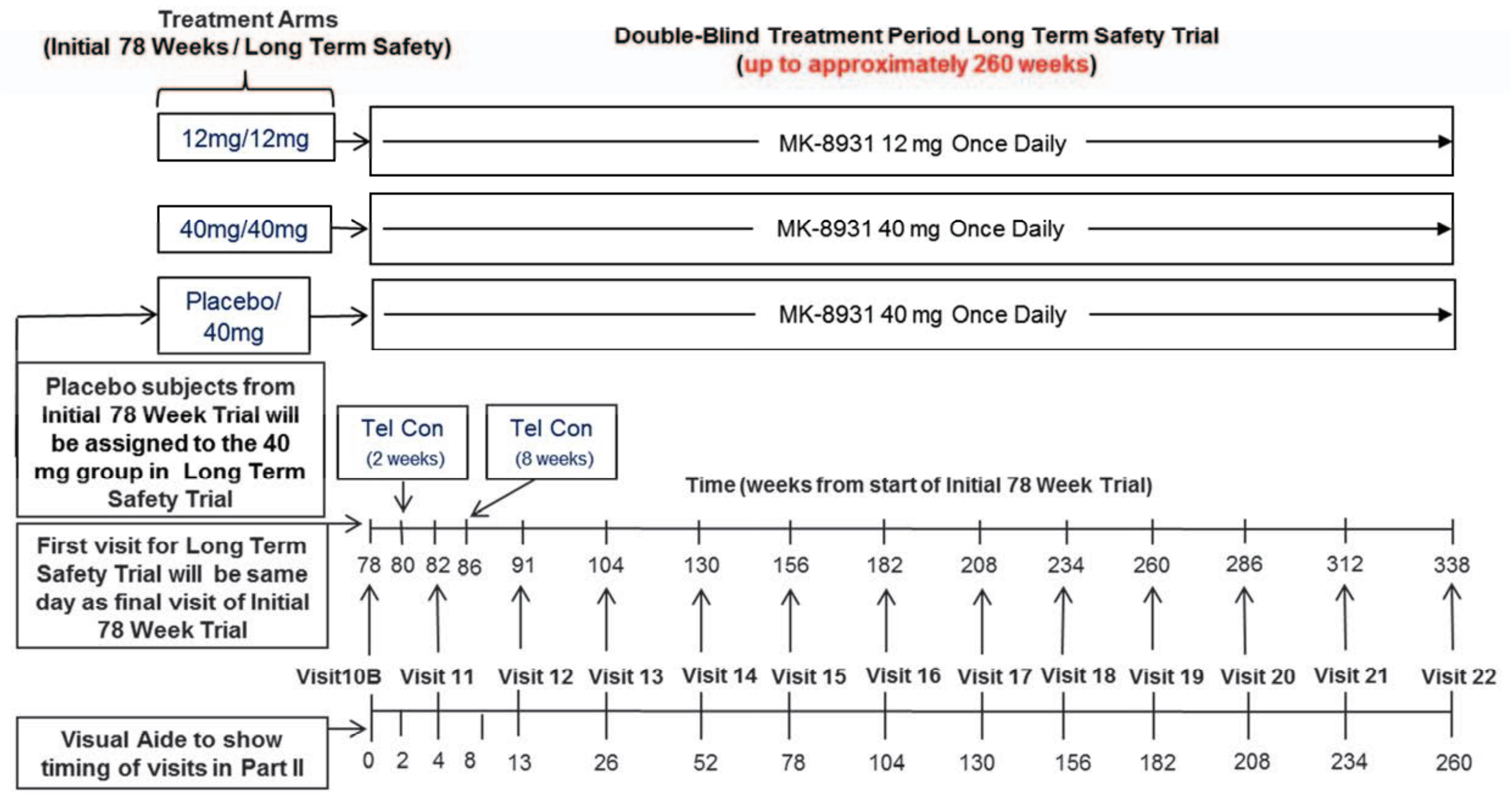

Figure 2 Trial Design Diagram

11.1.2 Extension Trial Flow Chart

NOTE, subject participation in Part II is optional and will only be conducted where approved by local authorities.

Extension Trial Period:	Treatment																	
Visit Number (continuing from Part I)	10B[b]	TC[c]	11	TC[c]	12	13	14	15	16	17	18	19	20	21	22	TC[c,e]	UV	ETV
Scheduled Week [a] (based on date of Randomization - in Part I)	78	80	82	86	91	104	130	156	182	208	234	260	286	312	338			
Visit Window (Weeks)		+/-1	+/-1	+/-1	+/-1	+/-4	+/-4	+/-4	+/-4	+/-4	+/-4	+/-4	+/-4	+/-4	+/-4			
Informed Consent	X																	
Issue/Collect Subject Identification Card															X			
Record Concomitant Medication	X	X	X	X	X	X	X	X	X	X	X	X	X	X	X	X	X[d]	X
Vital Signs			X		X	X	X		X		X				X		X[d]	X
Body Weight			X		X	X	X		X		X				X		X[d]	X
Directed Physical Exam							X		X		X		X		X		X[d]	X
12-Lead Electrocardiogram					X		X		X		X		X		X		X[d]	X
Hematology and Chemistry Samples			X		X	X	X		X		X		X		X		X[d]	X
PK/PD Blood Samples[g]			X		X													
Urinalysis			X		X	X	X		X		X		X		X		X[d]	X
Inclusion/Exclusion Criteria	X																	
ADAS-Cog					X	X	X		X		X		X		X			X
ADCS-ADL					X	X	X		X		X		X		X			X
CDR-SB						X	X		X		X		X		X			X
Mini-Mental State Examination (MMSE)						X	X		X		X		X		X			X
Neuropsychiatric Inventory (NPI)						X	X		X		X		X		X			X
Health Economic Assessment (HEA)						X	X		X		X		X		X			X
Modified Resource Utilization in Dementia (RUD) Lite						X	X		X		X		X		X			X
EuroQol Five Dimension Questionnaire (EQ-5D)						X	X		X		X		X		X			X
Columbia Suicide Severity Rating Scale (C-SSRS)			X		X	X	X	X	X	X	X	X	X	X	X		X[d]	X

Extension Trial Period:	Treatment																	
Visit Number (continuing from Part I)	10B[b]	TC[c]	11	TC[c]	12	13	14	15	16	17	18	19	20	21	22	TC[c,e]	UV	ETV
Scheduled Week [a] (based on date of Randomization - in Part I)	78	80	82	86	91	104	130	156	182	208	234	260	286	312	338			
Visit Window (Weeks)		+/-1	+/-1	+/-1	+/-1	+/-4	+/-4	+/-4	+/-4	+/-4	+/-4	+/-4	+/-4	+/-4	+/-4			
Structural Magnetic Resonance Imaging (MRI)																	X[d]	
Record Adverse Events		X	X	X	X	X	X	X	X	X	X	X	X	X	X	X	X	X
Dispense Trial Medication	X		X		X	X	X	X	X	X	X	X	X	X			X[f]	
Assess Medication Compliance			X		X	X	X	X	X	X	X	X	X	X	X		X	X
Drug Accountability Assessment			X		X	X	X	X	X	X	X	X	X	X	X		X	X
CAM	X --------------------To be completed only for adverse events of delirium (see 7.7.2.2.3).----------------X																	

a Visits scheduled during Part II will be based off of the date of randomization in Part I.
b Visit 10 from Part I and Visit 10B from Part II are considered a single visit and can be conducted on the same day.
c Telephone contact with subject and trial partner/caregiver by site to assess safety, AEs, medication compliance, and any other issues. Any telephone contact may be conducted as an in-person unscheduled visit if the subject or caregiver expresses a preference for this or if the site has significant safety/tolerability concerns.
d Procedures should be performed if clinically indicated as determined by the investigator.
e Telephone contact will be performed 14 to 21 days after the final visit.
f Drug dispensing may be performed at unscheduled visits at the discretion of the Investigator.
g Both plasma and dried blood spot (DBS) PK samples will be taken at each timepoint. The site will record the date and time of each PK sample and the date and time of the last two doses of trial medication before each PK sample. PK samples can be taken at the same time when blood samples for hematology and chemistry are taken.

11.2 EXTENSION RATIONALE

11.2.1 Rationale for Trial Extension

This is a parallel group, multi-site, double-blind, trial extension to provide access to MK-8931 before it is commercially available, to estimate the effects of delayed start of MK-8931, and to evaluate the safety and tolerability of two doses of MK-8931 in the extended treatment of mild to moderate AD. MK-8931 is hypothesized to exert disease modifying effects in subjects with AD. Delayed start designs may provide supportive evidence of disease modification in AD if the treatment effect is sustained and greater in a group that starts the active treatment early compared to a group with a substantial delay in start of active treatment. Results from Part II will also provide data regarding the sample sizes necessary for and the feasibility of delayed start studies with disease modifying therapeutics for AD. In addition, this extension period (Part II) will be used to further assess the long term effects of MK-8931 on disease progression, health economic effects, and the safety and tolerability of MK-8931. Some Sponsor personnel will be unblinded following the completion of Part I. Some Sponsor personnel will remain blinded during Part II, as necessary, for the conduct and collection of efficacy data, including the 24-month timepoint to support the primary objective regarding efficacy. Details are included in the Sponsor's blinding document.

Once a subject enters the extension period, participation will continue until one of the following occurs: 1) MK-8931's development is terminated for mild to moderate AD (eg. due to a lack of efficacy), 2) MK-8931 is approved by regulatory agencies and becomes commercially available, 3) the subject voluntarily withdraws or 4) the subject is discontinued for safety reasons. The extension period is expected to continue up to five years after the first subject enters the extension.

11.2.2 Dose and Administration Rationale for Extension

Subjects who tolerated study medication and completed Part I may continue in Part II. Those subjects who did not complete treatment in Part I, but did continue in the trial and completed Part I visits, may be permitted to enter Part II based on the discretion of the Sponsor. Subjects who received the 12 mg or 40 mg dose in Part I will continue to receive the same dose in Part II. They will be referred to as the 12/12 and the 40/40 groups respectively. Subjects who received placebo in Part I will receive the 40 mg dose in Part II. They will be referred to as the placebo/40 group.

C Confidential

The 40 mg dose has been shown to substantially inhibit CSF Aβ. The 12 mg dose produces a more moderate reduction in CSF Aβ and is included since the long term safety of greater inhibition is unknown. Using PK/PD modeling and simulation, 12 mg and 40 mg doses are projected to reduce CSF Aβ by at least 50% and 75%, respectively, in more than 90% of subjects. It is unknown how much Aβ lowering is optimal from the perspective of both safety/tolerability as well as efficacy. Subjects on placebo in the double blind period will be treated with the 40 mg dose in Part II to provide additional information about the long term safety of MK-8931 and to estimate the clinical effects of delayed start of this higher dose.

11.3 EXTENSION OBJECTIVE (S)

11.3.1 Extension Primary Objectives

There are two primary objectives for Part II, in which the subjects who received MK-8931 40 mg / MK-8931 40 mg will be compared to those who received placebo / MK-8931 40 mg. Similarly, subjects who received MK-8931 12 mg / MK-8931 12 mg will be compared to those who received placebo / MK-8931 40 mg. The two objectives are as follows:

1. To evaluate the safety and tolerability of MK-8931 in the long term treatment of mild to moderate Alzheimer's Disease.
2. To compare the efficacy of MK-8931 administered to subjects for 24 months to that of subjects administered placebo for 18 months followed by MK-8931 for 6 months using endpoints at follows.
    - the change-from-Baseline score in the ADAS-Cog at Week 104 (Visit 13).
    - the change-from-Baseline score in the ADCS-ADL at Week 104 (Visit 13).

11.3.2 Extension Exploratory Objective

To compare the efficacy of MK-8931 administered to subjects for 18 months to that of subjects administered placebo for 18 months in Part I followed by long term treatment of MK-8931 in Part II on cognition, function, disease progression, and health economic burden at multiple time points.

11.4 BEGINNING AND END OF TRIAL EXTENSION (PART II)

Each subject is considered to be enrolled in Part II when the subject (or the subject's legal representative) has provided written informed consent **in accordance with local requirements**.

Part II will start with enrollment of the first subject who completes Part I of the study and will end when the drug either becomes commercially available or when the MK-8931 program is terminated. Part II has an expected maximum duration of approximately 260 weeks, with the duration of individual subject participation dependent on the timing of enrollment in Part I, and hence Part II.

Each subject is considered to have ended participation in the trial when he/she has completed the last protocol-specified contact (eg, visits or telephone contacts) or has prematurely discontinued from the trial. A subject will be considered a completer of Part II if he/she is still continuing in the trial when the trial is stopped.

A subject is considered to have discontinued after he/she has withdrawn consent or has been discontinued under the conditions specified in **Section 11.5.3**. Given the exploratory nature of the Part II endpoints, the retrieved dropout approach employed in Part I of the trial, will not be extended to Part II. Thus, subjects who discontinue study medication in Part II, will be discontinued from the study. All applicable activities scheduled for the final trial visit should be performed at the time of treatment discontinuation as defined in the Extension Trial Flow Chart in **Section 11.1.2**.

A subject is considered to have been lost to follow-up if he/she is unable to be contacted by the investigator. The end of participation for a subject lost to follow-up is the last known contact (eg, visit or telephone contact).

Each subject will be monitored for the occurrence of AEs beginning immediately after the subject has signed informed consent through 14 days after following cessation of treatment. Follow-up procedures related to pregnancy or existing SAEs may continue beyond the end of the clinical trial.

11.5 EXTENSION POPULATION

Part II is designed to allow all subjects who completed Part I of the study to continue treatment until MK-8931 is commercially available or it is proved to be inefficacious.

11.5.1 Extension Inclusion Criteria

In order to be eligible for participation in the extension, the subject must:

1. have tolerated study medication and completed the initial 78-week period of the trial. Subjects who did not complete the initial 78 weeks of treatment but continued with scheduled visits may be permitted to continue in Part II at the discretion of the Sponsor. Subjects who repeatedly deviate from protocol requirements will not be permitted to continue in the extension. In addition, subjects who are less than 75% compliant with trial medication in Part I will not be permitted to continue in Part II, except in special circumstances, which will require Sponsor approval.

2. have a trial partner who is reliable and competent. The trial partner must have a close relationship with the subject, have face to face contact at least 3 days/wk for a minimum of 6 waking hours/wk (or more, based on local requirements), be willing to accompany the subject to all required trial visits, and be willing to monitor compliance of the administration of the trial medication. The trial partner should understand the nature of the trial and adhere to trial requirements (eg, dose, visit schedules, and evaluations). It is recommended that the trial partner accompany the subject to all trial visits.

3. sign (or legal representative sign) the informed consent form in accordance with local requirements, after the scope and nature of the investigation have been explained.

11.5.2 Extension Exclusion Criteria

The subject must be excluded from participating in the trial if the subject:

1. is at imminent risk of self-harm, based on clinical interview or on the Columbia Suicidality Severity Rating Scale (C-SSRS), or of harm to others in the opinion of the investigator. Subjects must be excluded if they report suicidal ideation with intent, with or without a plan (eg, suicidal ideation item 4 or 5 on the C-SSRS) in the past 1 month or suicidal behavior in the past 6 months.
2. has developed a recent or ongoing, uncontrolled, clinically significant medical condition (such as, but not limited to, diabetes, hypertension, thyroid or endocrine disease, congestive heart failure, angina, cardiac or gastrointestinal disease, dialysis, or abnormal renal function with estimated creatinine clearance < 30 mL/min) other than Alzheimer's disease such that, in the judgment of the investigator, participation in the trial would pose a significant medical risk to the subject. Controlled co-morbid conditions (including diabetes, hypertension, heart disease, etc) are not exclusionary if stable. All concomitant medications, supplements, or other substances must be kept as stable as medically possible during the trial.

Note: urinary tract infections at Visit 10B are not exclusionary if adequately treated (as documented by repeat urinalysis).

3. has a history of, or has developed during Part I evidence of long QT syndrome, QTC interval ≥ 470 milliseconds (for male subjects) or ≥ 480 milliseconds (for female subjects), or torsades de pointes.

4. anticipates receiving any of the treatments listed in Table 12 during Part II.

Table 12 Prohibited Medications, Supplements, and Other Substances
Anti-amyloid agents (eg, tarenflurbil, tramiprosate)
Anti-amyloid antibodies (eg, bapineuzumab)
Anti-amyloid vaccine<br>(Subjects who received placebo in a vaccine trial may participate in this trial.)
CYP3A4 inducers (strong) including: rifampicin and St. John's Wort, phenytoin, carbamazepine<br>**Exceptions:** Use of the following is acceptable: topical use; short-term (<2 weeks) oral treatment during the trial; use of oral St. John's Wort <300 mg three times a day during the trial, regardless of duration.
Drugs known to cause ocular changes or damage (eg, chloroquine, hydroxychloroquine [sometimes used for arthritis], many anti-malarial treatments, ethambutol for tuberculosis, amiodarone for ventricular arrhythmias, and tamoxifen for breast cancer)

NOTE: This is not a complete list of excluded medications. Contact the Sponsor if there is a question about a specific medication.

5. has developed a form of dementia that is not Alzheimer's disease, including but not limited to, dementia due to HIV infection, head trauma, vascular disease, Parkinson's disease, frontotemporal dementia, or Huntington's disease, as determined by the investigator.

11.5.3 Extension Discontinuation Criteria

A subject may discontinue from the clinical trial at any time for any reason. A subject **must** be discontinued from the trial if the subject or legal representative (such as a parent or legal guardian) withdraws consent.

The investigator or Sponsor should stop trial medication in any case in which emerging effects are of unacceptable risk to the individual subject, or if unmanageable factors arise that may interfere significantly with the trial procedures and/or the interpretation of results.

A subject must discontinue trial medication for any of the following reasons:

1. The subject or legal representative withdraws consent;
2. Elevated ALT, AST, or T-BIL meeting any one of the following criteria:
   a. ALT or AST $\geq$ 8 x ULN;
   b. ALT or AST $\geq$ 5 x ULN for more than 2 weeks;
   c. ALT or AST $\geq$ 3 x ULN and T-BIL $\geq$ 2 x ULN at the same visit;
   d. ALT or AST $\geq$ 3 x ULN with the appearance of symptoms indicating hepatitis (eg, worsening fatigue, nausea, vomiting, right upper quadrant pain or tenderness, fever, rash, or eosinophilia).

   Exception: if elevations are determined to be due to something other than study medication, subject may resume trial medication with Sponsor approval.

3. An imaging abnormality consistent with macrohemorrhage appears (see details in Section 7.1);
4. QTc prolongation (defined as QTc interval > 500 ms or QTc change from baseline > 60 ms, based on the average of three measurements using the Fridericia formula for correction);

   Exception: if QTc change from baseline > 60 ms is determined to be due to some other medical condition, or if subject has a new onset of bundle branch block, subject may continue treatment with Sponsor approval;
5. The subject develops a form of dementia that is not Alzheimer's disease, including but not limited to, dementia due to HIV infection, head trauma, vascular disease, Parkinson's disease, frontotemporal dementia, or Huntington's disease, as determined by the investigator;
6. The subject develops a severe rash. For the purpose of this program, a "**severe rash**" is defined as one of the following:
   - A vesicular rash (ie, one with blistering lesions) that is not clearly caused by herpes simplex virus or contact allergy such as poison ivy AND has EITHER a) extensive body surface area (BSA) involvement OR b) involves oral/mucosal surfaces
   - Stevens-Johnson Syndrome, erythroderma, or toxic epidermal necrolysis
   - DRESS syndrome
7. The subject develops an uncontrolled clinically significant rash defined as follows:
   - A clinically significant rash (see Section 7.7.2.2.3) that is not controlled by topical medications or oral medications such as antihistamines (detailed in the Rash Guidance Document), and
   - A clinically significant rash that causes intolerable discomfort for the subject.
8. The subject's trial partner is no longer willing or able to participate in the study and a suitable replacement trial partner cannot be found in a reasonable period of time

11.5.4 Replacement of Extension Subjects

A subject who discontinues from the extension will not be replaced.

11.6 Treatment in the Extension

11.6.1 Treatment in the Extension

The rationale for selection of doses to be used in this trial is provided in **Section 5.5** – Dose and Administration Rationale. There are no specific calculations or evaluations required to be performed in order to administer the proper dose to each subject.

The trial medication should be administered by the subject, by the subject's trial partner, or by a caregiver. Each subject should take one tablet at the same time every day. For each of the treatment groups, one tablet equals one dose of trial medication as summarized below:

- 12/12 group: MK-8931 12 mg (1 Tablet)
- 40/40 and placebo/40 groups: MK-8931 40 mg (1 Tablet)

If a subject misses a dose, the subject may take the dose later in the day and should continue with the regular dosing schedule by taking the next dose at the usual time the next day. Any changes in dosing schedule should be noted by the subject or subject's trial partner/caregiver and recorded by the site at the next visit. Subjects should not take more than one dose on the same calendar day.

With the exception of any subjects enrolled to a dose that is dropped for reasons of safety or tolerability, there will be no adjustments to the dose of any subject in the trial.

11.6.2 Investigational Medicinal Product

A double-blind technique will be used: all doses of MK-8931 will be identical in appearance and will be packaged identically so that the treatment blind is maintained. Neither the subject nor the investigational staff (sponsor, investigator, and evaluators) will know which treatment the subject is receiving. Sites and Subjects will remain blinded during Part II. The Sponsor will be unblinded following the completion of Part I.

See **Section 7.7.2.5.3** for a description of the method of unblinding a subject during the trial, should such action be warranted.

11.6.2.1 Source

The sponsor will provide trial medication in the extension as follows: MK-8931 12mg tablets; MK-8931 40 mg tablets.

11.6.2.2 Packaging

Extension medication will be provided in bottles.

At the visits specified in the Trial Flow Chart (**Section 11.1.2**), the site will dispense medication using the IVRS system. The site will provide the medication to the caregiver/trial partner and may provide a 'calendar sleeve' to attach to the subject's trial medication bottle. Additional instructions regarding the calendar sleeves will be provided to sites.

11.6.2.3 Labelling

Trial medication bottle labels should include the following information and comply with the regulatory requirements appropriate for clinical site: Dosing directions will state, "Take 1 tablet once a day".

11.6.2.3.1.1 Calendar Sleeves for Study Medication Bottles

In addition to routine surveillance of subject trial medication compliance, subjects/ caregiver/trial partner may be given trial medication 'calendar sleeves' which affix to the exterior of trial medication bottles to help subjects remember when to take trial medication. The intention of the sleeves is to assist the subject/caregiver/trial partner to remember when the subject missed a dose. The sponsor will provide the sleeve to the sites and sites personnel will slide them onto each bottle. The site will then instruct the caregiver/trial partner on how to make sleeve subject specific (ie document on the 'calendar sleeve' the date to start taking trial medication, bottle number).

When the subject/caregiver/trial partner returns to the clinical site, the site will perform a pill count and also review the 'calendar sleeves'(if they have utilized the sleeve), to verify pill count and any possible missed or extra doses taken. If there is a discrepancy between the bottle and the 'calendar sleeve' then the physical pill count will be the final decision to be documented in the eCRF. Discrepancies should be documented in the site's source documents and the reasoning for what was ultimately recorded in the eCRF.

11.6.3 Prior and Concomitant Medications

11.6.3.1 Medications, Supplements and Other Substances Prohibited Prior to Screening and During the Extension

The subject must not take the treatments listed in Table 12 prior to Screening and during the Extension.

During the extension, initiation of treatment with medications known to be associated[21,22] with substantial increased risk of Stevens-Johnson Syndrome and toxic epidermal necrolysis should be avoided when possible. Subjects who have been safely treated with at least one routine course of treatment with these medications in the past are exempted from this requirement. Examples of such medications are included below. The sponsor should be consulted for questions about specific medications.

- trimethoprim-sulfamethoxazole, azithromycin, allopurinol, phenobarbital, oxicam NSAIDS (eg, celecoxib, valdecoxib, meloxicam) carbamazepine, phenytoin, valproic acid, nevirapine, lamotrigine, and chlormezanone

11.6.3.2 Concomitant Medications, Supplements, and Other Substances Allowed During the Extension

Medications, supplements, and other substances allowed during the trial include, but are not limited to, those listed in Table 13.

Table 13: Medications, Supplements, and Other Substances Allowed During the Trial

**Allowed Medications, Supplements, and Other Substances**
Acetylcholinesterase inhibitors (eg, donepezil, tacrine, rivastigmine, galantamine)
Memantine
Huperzine A
Vitamin E
Herbal supplements from *Ginkgo biloba*, ginseng, *Huperzia serrata* (Qian Ceng Ta)
Medical foods/supplements (eg, Axona®, Souvenaid®)
Estrogens and estrogen-like compounds
Antihypertensives
Nonsteroidal anti-inflammatory drugs (NSAIDs)
Cycloxygenase 2 inhibitors
Analgesics/Narcotics: Use of ≤ 2 doses/week or short-term use (<1 month) of more than 2 doses/week for temporary conditions is acceptable (eg, codeine, morphine , hydromorphone, oxycodone, propoxyphene (Darvon) and its variations, & combination products that contain a narcotic).
**Neuroleptics**: asenapine, aripiprazole, olanzapine, quetiapine, risperidone, ziprasidone
**Sedative/benzodiazepines:** Use of the following medications is acceptable: trazodone, mirtazapine, zaleplon ≤ 5 mg, zopiclone≤ 7.5 mg, eszopiclone ≤ 3 mg, zolpidem ≤ 5 mg, or lorazepam ≤ 1.0 mg. For other medications in this category not specified here, please contact the Sponsor for guidance
**Antidepressants:** bupropion, citalopram (40 mg or less), escitalopram, fluoxetine, mirtazapine, paroxetine, sertraline, venlafaxine. Use of 50mg or less at night of nortriptyline or desipramine during the trial is acceptable.
**Carbidopa/levodopa and dopamine agonists** are allowed for treating restless leg syndrome
**Pregabalin and gabapentin:** Treatment for neuropathic pain
**Anticholinergic medications:** Daily use of the anticholinergic medications for incontinence (eg, oxybutynin, tolterodine, darifenacin, solifenacin, trospium, fesoterodine), nasal spray for rhinorrhea (ipratropium) or inhalants for pulmonary disorders (eg, tiotropium) is acceptable.
Rifampicin and St. John's Wort: Topical use, short-term (<2 weeks) oral treatment during the trial, use of oral St. John's Wort <300 mg three times a day during the trial, regardless of duration.
Corticosteroids: Low dose oral treatment with the equivalent of 10 mg prednisone or less, short-term (<3 weeks) oral treatment with the equivalent of 60 mg prednisone or less, if needed for management of rash, local injections into joints or bursae, topical use, inhaled and nasal use
Selective H1 blockers, selective H2 blockers, and topical anti-pruritic treatments for treatment of rash during the trial, as specified in the Rash Guidance Document

11.6.4 Extension Schedule

A visit-by-visit schedule of extension activities is provided in The Extension Flow Chart in **Section 11.1.2**.

11.6.5 Extension Procedures

The Extension Flow Chart in Section 11.1.2 summarizes the extension procedures to be performed at each visit.

In order to minimize variability of evaluations, it is preferred that the same individuals perform the same types of evaluations for all subjects at each trial site.

1. Explain Trial Extension and Obtain Written Informed Consent

   The investigator or qualified designee will explain the extension to the subject, answer all of his/her questions, and obtain written informed consent before performing any extension-related procedure. A copy of the informed consent will be given to the subject (see Section 9.1.2 for further description of the Informed Consent).

   Given the trial population and the duration of the trial, it is possible that a subject's cognition may decline to a point where they no longer have capacity to provide informed consent. If this occurs, the site should obtain consent to continue in the trial from the subject's legally acceptable representative and in accordance with local standards and requirements. The subject's assent to continue should also be obtained.
2. Issue or Collect Subject Identification Card
3. The investigator or qualified designee will provide the subject with a Subject Identification Card in Part I after the subject provides written informed consent. The investigator or qualified designee will retrieve the card from the subject at the last contact (see Section 9.1.3 for further description of the Subject Identification Card).Record Concomitant Medications

   A record of concomitant medication taken by the subject during the trial will continue to be obtained.
4. Record (Serious) Adverse Events

   See Section 7.7.2.4, for instructions on the assessment and reporting of (Serious) Adverse Events and Section 7.7.2.5 for instructions on the reporting of (Serious) Adverse Events to the sponsor.
5. Vital Signs

   The following vital signs will be measured and recorded: pulse (beats/minute), BP (mm Hg), temperature (°C/°F), and respiratory rate (breaths per minute). Blood pressure should be measured in the sitting position.
6. Body Weight (kg/lbs)

   Body weight data will be collected and recorded. Body weight data will be collected without shoes and with heavy clothing removed. Body weight should be performed on the same scale for the same individual. Measurements should be recorded to the nearest kilogram/pounds and centimeter/inches.

7. Physical & Neurological Examinations

   A physical examination including a standard neurological examination will be performed. If the subject is discontinued for any reason during the treatment phase, every attempt should be made to perform a final physical examination.

8. Mini-Mental State Examination (MMSE)

   The MMSE will be administered to the subject and scored and recorded by the principal investigator or trained designee according to the instructions in the **Manual of Assessments**.

9. Columbia Suicide Severity Rating Scale (C-SSRS)

   The C-SSRS will be administered to the subject and scored and recorded by the principal investigator or trained designee according to the instructions in the **Manual of Assessments**.

   The C-SSRS provides a detailed assessment of suicidal ideation and behaviors. The C-SSRS will be completed at each visit as indicated in the Extension Flow Chart (and unscheduled visits as clinically indicated). At baseline, information will be collected regarding the subject's lifetime history of suicidal ideation and behavior, while information collected at subsequent visit will be based on the time interval since the previous visit. In addition, subjects who at any time during this study spontaneously report AEs of suicidal ideation or behavior with intent (with or without a plan), either as outpatient or during visit interviews, must be assessed by the Investigator and referred for further mental health evaluation as clinically indicated. Subjects with treatment-emergent suicidal ideation and/or behavior with intent (with or without a plan), must be evaluated that day by a psychiatrist or other trained mental health professional who is a licensed psychologist, social worker or nurse practitioner (or comparable professional qualification in countries outside the United States). Only subjects whose suicidal ideation is passive, who expressly deny any intent to act, and who, after evaluation, are not judged to be at serious risk for self-harm during the course of the trial may continue with trial treatment; others must be discontinued from trial treatment and receive appropriate clinical follow-up care to assure their safety. After appropriate follow-up care, if the investigator judges that the subject can safely resume trial treatment, re-dosing can be considered with Sponsor approval. As part of site validation, sites are to indicate which health care professionals are to be responsible for acute care on-site and to specify referral center(s) to be used for further evaluation. All reports of suicidal ideation or behavior must be recorded as an Event of Clinical Interest (ECI).

10. Neuropsychiatric Inventory (NPI)

    The NPI will be administered to the subject's trial partner/caregiver and scored and recorded by the principal investigator or trained designee according to the instructions in the **Manual of Assessments**.

11. Review Inclusion/Exclusion Criteria

    The inclusion and exclusion criteria will be reviewed by the investigator or qualified designee to ensure that the subject qualifies for the extension.

C Confidential

12. Alzheimer's Disease Assessment Scale Cognitive Subscale (ADAS-Cog)

    The ADAS-Cog will be administered to the subject and scored and recorded by the principal investigator or trained designee according to the instructions in the **Manual of Assessments**.

13. Alzheimer's Disease Cooperative Study Activities of Daily Living Inventory (ADCS-ADL)

    The ADCS-ADL will be administered to the subject's trial partner/caregiver and scored and recorded by the principal investigator or trained designee according to the instructions in the **Manual of Assessments**.

14. 12-Lead Electrocardiogram (ECG)

    A 12-Lead Electrocardiogram will be performed according to the instructions in a separate **ECG Instruction Manual.**

15. Laboratory Tests

    Laboratory tests for hematology, blood chemistry, and urinalysis are specified in Table 14.

16. Pharmacokinetic/Pharmacodynamic Blood Samples

    Blood samples for PK/PD analyses should be collected, processed, stored, and packaged according to the instructions in the **Laboratory Manual**.

    Record the following information for each PK/PD blood sample collected:

    - Date and time of each PK/PD blood sample
    - Date and time of the last two doses of trial medication before each PK/PD blood sample

    Careful attention to the collection, handling, and storage of the PK/PD blood samples is essential to reduce the risk of hemolysis and PK/PD variability. Any deviations from the PK/PD blood collection schedule, such as a missing or breaking a sample, should be documented**.**

Table 14 Laboratory Tests

Hematology	Chemistry	Urinalysis
Basophils	Albumin	Blood
Eosinophils	Alkaline phosphatase	Glucose
Hematocrit	ALT (SGPT)	Ketones
Hemoglobin	AST (SGOT)	Microscopic exam
Lymphocytes	Bicarbonate	pH
Monocytes	Blood urea nitrogen (BUN)	Protein
Neutrophils	Calcium	Specific gravity
Platelets	Chloride	
RBC	Cholesterol	
WBC	Creatinine	
	Glucose	
	Inorganic phosphorus	
	LDH	
	Potassium	
	Sodium	
	Total Bilirubin	
	Total protein	

17. Clinical Dementia Rating (CDR)

    The CDR will be administered to both the subject and the subject's trial partner/caregiver, and the CDR Sum of Boxes (CDR-SB) will be scored and recorded by the principal investigator or trained designee according to the instructions in the **Manual of Assessments**.

18. Modified Resource Utilization in Dementia (RUD) Lite Questionnaire

    A modified **RUD Lite** Questionnaire will be administered to the subject's trial partner/caregiver and recorded by the principal investigator or trained designee according to the instructions in the **Manual of Assessments**.

19. Health Economic Assessment (HEA)

    The **HEA** will be administered to the subject's trial partner/caregiver and recorded by the principal investigator or trained designee according to the instructions in the **Manual of Assessments**.

20. EuroQol Five Dimension Questionnaire (EQ-5D)

    The EQ-5D will be administered to the subject's trial partner/caregiver and recorded by the principal investigator or trained designee according to the instructions in the **Manual of Assessments**.

21. Structural Magnetic Resonance Imaging (MRI)

    Structural MRI for safety monitoring may be conducted at an unscheduled visit if the investigator considers that it is clinically indicated due to an adverse event.

22. Dispense Trial Medication

    The investigator or qualified designee will dispense the subject's treatment kit (see Section 7.4.1.5.8) and instruct the subject and subject's trial partner/caregiver regarding dosing with trial medication (see Section 7.4.1.3.2).

23. Medication Compliance/Drug Accountability Assessment

    The investigator or qualified designee will account for trial medication as described in Section 7.4.1.5.8.

24. Confusion Assessment Method

    The CAM is completed by the rater based on information provided by other sources (i.e. medical records, asking the subject questions). It should be completed only if and when an adverse event of delirium is suspected to ascertain the diagnosis of delirium.

11.6.6 Extension Assessments

11.6.6.1 Efficacy Assessments

It is noted that baseline refers to the baseline from Part I (Week 0).

11.6.6.1.1 Extension Primary Efficacy Endpoints

The primary efficacy endpoints for Part II are:

1. the change-from-Baseline score in the ADAS-Cog at Week 104 (Visit 13)
2. the change-from-Baseline score in the ADCS-ADL at Week 104 (Visit 13)

11.6.6.1.2 Extension Exploratory Efficacy Endpoints

The exploratory efficacy endpoints for Part II are:

1. the change-from-Baseline score in the ADAS-Cog (at all scheduled timepoints with the exception of Week 104),
2. the change-from-Baseline score in the ADCS-ADL (all scheduled timepoints with the exception of Week 104)
3. the change-from-Baseline score in the CDR-SB (all scheduled timepoints).
4. the change-from-Baseline score in the MMSE (all scheduled timepoints)
5. the change-from-Baseline score in the NPI (all scheduled timepoints)
6. the Health Economics and Quality of Life Endpoints
7. the Pharmacogenetic Analyses

11.6.7 Safety Assessments

11.6.7.1 Extension Safety Endpoints

The same safety parameters and AEs designated as being of special interest in Part I will continue to be monitored in Part II, as follows:1) delirium; and 2) rash ECI (see **Section 7.7.2.2.3**). Some of these AEs are combined into composite endpoints for formal safety analyses (see Table 11).

11.7 STATISTICAL AND ANALYTIC PLAN FOR EXTENSION POPULATION

11.7.1 Subjects to be Analyzed

The same analysis populations used in Part I (the Full Analysis Set population, the Modified Full Analysis Set population and the All-Patients-as-Treated population), as defined in **Section 8.1**, will also be used in Part II, adhering to the following conditions: All available observations from Part I will be included in the relevant Part II model-based FAS/MFAS analyses. Observations from Part II will be included according to the FAS/MFAS principle outlined in Section 8.1, with the following additional requirement: the subject must have taken at least one dose of study medication within the 28 days preceding Visit 10B and within the 28 days following Visit 10B.

Confidential

11.7.2 Efficacy Analyses

Table 15 Analysis Strategy for Primary Endpoints

Endpoint/Variable (Description, Time Point)	Primary vs. Supportive Approach	Statistical Method†	Analysis Population	Missing Data Approach
**Primary Endpoints**				
**Primary Endpoints:**				
CFB at Week 104 (Visit 13) in ADAS-Cog score	P	Longitudinal ANCOVA	FAS	Model-based
	S	Longitudinal ANCOVA	FAS	Pattern-Mixture Model (Tipping Point)
	S	Longitudinal ANCOVA (Exclude data collected after the introduction of AChEIs and/or study medication discontinuation)	FAS	Model-based
	S	cLDA	MFAS	Model-Based
CFB at Week 104 (Visit 13) in ADCS-ADL score	P	Longitudinal ANCOVA	FAS	Model-based
	S	Longitudinal ANCOVA	FAS	Pattern-Mixture Model (Tipping Point)
	S	Longitudinal ANCOVA (Exclude data collected after the introduction of AChEIs and/or study medication discontinuation)	FAS	Model-based
	S	cLDA	MFAS	Model-Based

The primary model contains categorical terms for treatment, time, geographic region, gender, APOE genotype, study cohort, and the interaction of time-by-treatment, with the Baseline values of MMSE, and age included as continuous covariates. Terms for the baseline value and the baseline-by-time interaction of the dependent variable will also be included.

ADAS-Cog=Alzheimer's Disease Assessment Scale Cognitive subscale; ADCS-ADL=Alzheimer's Disease Cooperative Study Activities of Daily Living Inventory; CDR-SB=Clinical Dementia Rating Sum of Boxes; CFB=Change from Baseline; ANCOVA=Analysis of Covariance; cLDA=Constrained Longitudinal Data Analysis; FAS=Full Analysis Set; MMSE=Mini-Mental State Examination; NA=Not Applicable; NPI=Neuropsychiatric Inventory; P=Primary; S=Supportive.

11.7.2.1 Primary Efficacy Analysis

The primary efficacy endpoints are the change from Baseline (CFB) in ADAS-cog score and the change from Baseline in ADCS-ADL score at Week 104 (Visit 13).

The primary analysis approach will be conducted separately on each of the endpoints and will be conducted as outlined in **Section 8.2**. In addition, a delayed start design will be used to support the potential disease modifying effect of MK-8931 by comparing the 40mg/40mg arm to the Pbo/40mg arm. However, due to increased variance and anticipated dropout rate over time, the study is not powered to demonstrate significance on these endpoints, even if significance was demonstrated at 18 months.

All available Part I data will be utilized in the anlayses, even that from subjects who do not continue into Part II.

It is possible that the 40 mg dose may be dropped for reasons due to safety, in which case all subjects would receive 12 mg. The primary comparison in this case would be between the 12 mg / 12 mg arm and the Pbo / 12 mg arm (with any subjects receiving 40 mg or 60 mg excluded from the analysis).

11.7.2.2 Exploratory Analysis

All analyses of exploratory endpoints, as listed in **Section 11.6.6.1.2** , will be conducted in the same manner as was used in Part I (**Section 8.2**).

11.7.2.3 Multiplicity

As there are no formal hypotheses for the extension study, there is no need for a formal multiplicity strategy. However, 97.51% CIs will be produced for all endpoints originally specified in Figure 1 even though formal hypotheses are not defined for the extension study and it is acknowledged that strong control is not present.

11.7.2.4 Subgroup Analyses

Subgroup analyses will be performed as outlined in **Section 8.2.7**.

11.7.2.5 Parameter Estimates for the Primary Endpoints

11.7.2.5.1 Assumptions for the Effect of MK-8931

Extrapolations out to 24 months (Week 104) were performed on the 18-month (Week 78) parameter assumptions presented in Table 6 and these extrapolations are presented below in Table 16.

Table 16 Parameter Assumptions for Progression Rate on Placebo, Standard Deviations, and Within Endpoint Correlations for ADAS-Cog, ADCS-ADL, and CDR-SB

Endpoint	104-Week Placebo/ MK-8931 40 mg Progression Rate	Baseline Standard Deviation	104-Week Standard Deviation	Correlation (Baseline, Week 104)
ADAS-Cog	7.45	7.75	14.97	0.65
ADCS-ADL	-11.73	10.71	20.21	0.61
CDR-SB	2.91	2.28	4.65	0.56

ADAS-Cog=Alzheimer's Disease Assessment Scale Cognitive subscale; ADCS-ADL=Alzheimer's Disease Cooperative Study Activities of Daily Living Inventory; CDR-SB=Clinical Dementia Rating Sum of Boxes

11.7.2.5.2 Power Calculations

Power calculations for the change-from-baseline treatment difference at 24 months (Week 104) were performed (via simulation) for ADAS-Cog and ADCS-ADL, even though there are no formal hypotheses for these endpoints in the extension study. As shown in Table 17, both of these hypotheses are underpowered at Week 104, both marginally and when applying an informal sequential testing approach. Specifically, it is possible that formal statistical significance may be observed in the base study (under the prespecified multiplicity approach), while nominal marginal significance (ie, p-value < 0.02495) fails to be observed at Week 104, even if the true underlying effect of MK-8931 continues to increase. This is due to the assumed increased variance and dropout rate over time, with 90% of subjects who complete the base study assumed to continue on into the extension.

Table 17 Power Calculations (MK-8931 40 mg / MK-8931 40 mg vs. Placebo / MK-8931 40 mg)

<u>**Marginal Probability**</u>†	**(%)**
**ADAS-Cog** at Week 104	**62.3**
**ADCS-ADL** at Week 104	**74.1**
**ADAS-Cog** and **ADCS-ADL** at Week 104	**48.0**
<u>**Sequential Probability**</u>	
Success‡ for ADAS-Cog and ADCS-ADL at Week 78 and **ADAS-Cog** at Week 104	**58.0**
Success‡ for ADAS- Cog and ADCS-ADL at Week 78 and **ADCS-ADL** at Week 104	**66.5**
Success‡ for ADAS-Cog and ADCS-ADL at Week 78 and **ADAS-Cog** and **ADCS-ADL** at Week 104	**46.1**
<u>**Conditional Probability**</u>	
Success‡ for **ADAS-Cog** at Week 104 given success on ADAS-Cog and ADCS-ADL at Week 78	**68.4**

Success‡ for **ADCS-ADL** at Week 104 given success on ADAS-Cog and ADCS-ADL at Week 78	**78.4**
Success‡ for **ADAS-Cog and ADCS-ADL** at Week 104 given success on ADAS-Cog and ADCS-ADL at Week 78	**54.4**

† Marginal power calculations at Week 104 do not require success at Week 78.
‡ 40 mg dose only
Calculations are based on N=1710 randomized (570 subjects/arm) incorporating the assumed dropout rate of 30% at Week 78 and 45% at Week 104.(α = 0.0249).

11.7.3 Dropping Dose Arms

In the event of a dose being dropped in the main study, the placebo arm will be given the dose that is still active.

11.7.4 Accounting for Missing Data

The same approach outlined in Section 8.2.10 for accounting for missing data will be followed in the extension study.

11.7.5 Safety Analyses

Subjects from all three arms (40 mg / 40 mg, 12 mg / 12 mg, Pbo / 40 mg) will be included in the safety analyses (with any subject treated with MK-8931 60 mg still excluded from the primary analysis). Where applicable, treatment comparisons will be conducted between the 40 mg / 40 mg and Pbo / 40 mg arms, as well as between the 12 mg / 12 mg and Pbo / 40 mg arms.

The broad clinical and laboratory AE categories consisting of the percentage of subjects with any AE, with a drug related AE, with an SAE, with an AE which is both drug-related and serious, or who discontinued because of an AE are considered as Tier 2 endpoints and will be analyzed. Descriptive Safety Endpoints are considered as Tier 3 events and will also be analyzed.

Analyses will be conducted both on the cumulative data only (Part I and Part II), no analyses will be conducted on Part II alone.

11.7.6 Trial Medication Compliance and Exposure

11.7.6.1 Compliance

Compliance procedure specified in Section 8.4.1 will be followed in the extension study.

11.7.6.2 Exposure

Basic summary statistics for the number of doses of trial medication taken will be calculated overall, and as well as over each 3-month time frame by treatment group and dose. The cumulative percent of subjects taking various numbers of doses of trial medication (eg, one dose, 30 doses) will also be calculated. The APaT population will be used for all trial medication exposure calculations.

11.7.7 Demography

Basic summary statistics (means, standard deviations, counts, percentages) will be provided, as applicable, by treatment group, for subject baseline characteristics, subject disposition, and prior and concomitant medication usage.

12.0 REFERENCES

1. Salloway S, Sperling R, Gilman S, Fox NC, Blennow K, Raskind M, Sabbagh M, Honig LS, Doody R, van Dyck CH, Mulnard R, Barakos J, Gregg KM, Liu E, Lieberburg I, Schenk D, Black R, Grundman M; Bapineuzumab 201 Clinical Trial Investigators. A phase 2 multiple ascending dose trial of bapineuzumab in mild to moderate Alzheimer disease. Neurology. 2009 Dec 15;73(24):2061-70.
2. Sevigny JJ, Ryan JM, van Dyck CH, Peng Y, Lines CR, Nessly ML; MK-677 Protocol 30 Study Group. Growth hormone secretagogue MK-677: no clinical effect on AD progression in a randomized trial. Neurology. 2008 Nov 18;71(21):1702-8.
3. Fleisher AS, Raman R, Siemers ER, Becerra L, Clark CM, Dean RA, Farlow MR, Galvin JE, Peskind ER, Quinn JF, Sherzai A, Sowell BB, Aisen PS, Thal LJ. Phase 2 safety trial targeting amyloid beta production with a gamma-secretase inhibitor in Alzheimer disease. Arch Neurol. 2008 Aug;65(8):1031-8.
4. Wilcock GK, Black SE, Hendrix SB, Zavitz KH, Swabb EA, Laughlin MA; Tarenflurbil Phase II Study investigators. Efficacy and safety of tarenflurbil in mild to moderate Alzheimer's disease: a randomised phase II trial. Lancet Neurol. 2008 Jun;7(6):483-93.
5. http://newsroom.lilly.com/releasedetail.cfm?releaseid=499794
6. Buchhave P, Blennow K, Zetterberg H, Stomrud E, Londos E, Andreasen N, Minthon L, Hansson O. Longitudinal study of CSF biomarkers in patients with Alzheimer's disease. PLoS One. 2009 Jul 17;4(7):e6294.
7. Becker RE, Greig NH. Alzheimer's disease drug development: old problems require new priorities. CNS Neurol Disord Drug Targets. 2008 Dec;7(6):499-511.
8. Becker RE, Greig NH. Alzheimer's disease drug development in 2008 and beyond: problems and opportunities. Curr Alzheimer Res. 2008 Aug;5(4):346-57.

9. Rosen WG, Terry RD, Fuld PA, Katzman R, Peck A. Pathological Verification of Ischemic Score in Differentiation of Dementias. Ann Neurol. 1980 May; 7(5):486-488.

10. Inouye SK, van Dyck CH, Alessi CA, Balkin S, Siegal AP, Horwitz RI. Clarifying confusion: the confusion assessment method. A new method for detection of delirium. Ann Intern Med. 1990 Dec 15;113(12):941-8.

11. Liang K, Zeger, S. Longitudinal data analysis of continuous and discrete responses for pre-post designs. Sankhyā: The Indian Journal of Statistics. 2000;62(Series B, Pt. 1):134-148.

12. Schneider LS, Sano M. Current Alzheimer's disease clinical trials: methods and placebo outcomes. Alzheimers Dement. 2009 Sep;5(5):388-97.

13. Aisen PS, Schneider LS, Sano M, Diaz-Arrastia R, van Dyck CH, Weiner MF, Bottiglieri T, Jin S, Stokes KT, Thomas RG, Thal LJ; Alzheimer Disease Cooperative Study. High-dose B vitamin supplementation and cognitive decline in Alzheimer disease: a randomized controlled trial. JAMA. 2008 Oct 15;300(15):1774-83.

14. Saumier D, Aisen PS, Gauthier S, Vellas B, Ferris SH, Duong A, Suhy J, Oh J, Lau W, Garceau D, Haine D, Sampalis J. Lessons learned in the use of volumetric MRI in therapeutic trials in Alzheimer's disease: the ALZHEMED (Tramiprosate) experience. J Nutr Health Aging. 2009 Apr;13(4):370-2.

15. Feldman HH, Doody RS, Kivipelto M, Sparks DL, Waters DD, Jones RW, Schwam E, Schindler R, Hey-Hadavi J, DeMicco DA, Breazna A; LEADe Investigators. Randomized controlled trial of atorvastatin in mild to moderate Alzheimer disease: LEADe. Neurology. 2010 Mar 23;74(12):956-64.

16. Vellas B, Andrieu S, Sampaio C, Coley N, Wilcock G; European Task Force Group. Endpoints for trials in Alzheimer's disease: a European task force consensus. Lancet Neurol. 2008 May;7(5):436-50.

17. Vellas B, Andrieu S, Cantet C, Dartigues JF, Gauthier S. Long-term changes in ADAS-cog: what is clinically relevant for disease modifying trials in Alzheimer? J Nutr Health Aging. 2007 Jul-Aug;11(4):338-41.

18. McKhann G, Drachman D, Folstein M, Katzman R, Price D, Stadlan EM. Clinical diagnosis of Alzheimer's disease: report of the NINCDS-ADRDA Work Group under the auspices of Department of Health and Human Services Task Force on Alzheimer's Disease. Neurology. 1984 Jul;34(7):939-44.

19. Doc ID 5706793. Investigator's Brochure of MK-8931 (Formerly SCH 900931)

20. Morse AR, Teresi J, Rosenthal B, Holmes D, Yatzkan ES. J. Vis. Impair. Blind. Visual acuity assessment in persons with dementia. 2004 Sep;98(9) 560-566.

21. Roujeau JC, Kelly JP, Naldi L, Rzany B, Stern RS, Anderson T, Auquier A, Bastuji-Garin S, Correia O, Locati F, et al. Medication use and the risk of Stevens-Johnson syndrome or toxic epidermal necrolysis. N Engl J Med. 1995 Dec 14;333(24):1600-7.

22. Mockenhaupt M, Viboud C, Dunant A, Naldi L, Halevy S, Bouwes Bavinck JN, Sidoroff A, Schneck J, Roujeau JC, Flahault A. Stevens-Johnson syndrome and toxic epidermal necrolysis: assessment of medication risks with emphasis on recently marketed drugs. The EuroSCAR-study. J Invest Dermatol. 2008 Jan;128(1):35-44.

23. Haybittle JL. Repeated assessment of results in clinical trials of cancer treatment. Br J Radiol. 1971 Oct;44(526):793-7.

24. Peto R, Pike MC, Armitage P, Breslow NE, Cox DR, Howard SV, Mantel N, McPherson K, Peto J, Smith PG. Design and analysis of randomized clinical trials requiring prolonged observation of each patient. I. Introduction and design. Br J Cancer. 1976 Dec;34(6):585-612.

25. Rubin, D. B. (1987) Multiple Imputation for Nonresponse in Surveys. John Wiley:NY.

26. Ratitch B, O'Kelly, M, Tosiello, R. "Missing data in clinical trials: from clinical assumptions to statistical analysis using pattern mixture models." Pharmaceutical Statistics. 2013; 12: 337–347

27. SAS Institute Inc. 2011. SAS/STAT® 9.3 User's Guide. Cary, NC: SAS Institute Inc. (Chapter 57).

28. Schafer, J.L. (1997) Analysis of Incomplete Multivariate Data. Chapman & Hall:London.

29. Barnard J, Rubin DB. Small-sample degrees of freedom with multiple imputation. Biometrika, 1999; 86: 948–55.

30. Rochin L, Hurbain I, Serneels L, Fort C, Watt B, Leblanc P, Marks MS, De Strooper B, Raposo G, van Niel G. BACE2 processes PMEL to form the melanosome amyloid matrix in pigment cells. Proc Natl Acad Sci U S A. Jun 25;110(26):10658-63. 2013.

Confidential

Appendix 1 Code of Conduct for Clinical Trials

Merck*

Code of Conduct for Clinical Trials

I. **Introduction**

A. **Purpose**

Merck, through its subsidiaries, conducts clinical trials worldwide to evaluate the safety and effectiveness of our products. As such, we are committed to designing, implementing, conducting, analyzing and reporting these studies in compliance with the highest ethical and scientific standards. Protection of subject safety is the overriding concern in the design of clinical trials. In all cases, Merck clinical studies will be conducted in compliance with local and/or national regulations and in accordance with the ethical principles that have their origin in the Declaration of Helsinki.

B. **Scope**

Such standards shall be endorsed for all clinical interventional investigations sponsored by Merck irrespective of the party (parties) employed for their execution (eg, contract research organizations, collaborative research efforts). This Code is not intended to apply to studies which are observational in nature, or which are retrospective. Further, this Code does not apply to investigator-initiated studies (eg, Medical School Grant Program), which are not under the control of Merck.

II. **Scientific Issues**

A. **Trial Conduct**

1. **Trial Design**

Except for pilot or estimation studies, clinical trial protocols will be hypothesis-driven to assess safety, efficacy and/or pharmacokinetic or pharmacodynamic indices of Merck or comparator products. Alternatively, Merck may conduct outcomes research trials, studies to assess or validate various endpoint measures, or studies to determine patient preferences, etc.

The design (ie, subject population, duration, statistical power) must be adequate to address the specific purpose of the trial. Research subjects must meet protocol entry criteria to be enrolled in the trial.

2. **Site Selection**

Merck selects investigative sites based on medical expertise, access to appropriate patients, adequacy of facilities and staff, previous performance in Merck studies, as well as budgetary considerations. Prior to trial initiation, sites are evaluated by Merck personnel to assess the ability to successfully conduct the trial.

3. **Site Monitoring/Scientific Integrity**

Trial sites are monitored to assess compliance with the trial protocol and general principles of Good Clinical Practice. Merck reviews clinical data for accuracy, completeness and consistency. Data are verified versus source documentation according to standard operating procedures. Per Merck policies and procedures, if fraud, misconduct or serious GCP-non-Compliance are suspected, the issues are promptly investigated. When necessary, the clinical site will be closed, the responsible regulatory authorities and ethics review committees notified and data disclosed accordingly.

D. **Publication and Authorship**

To the extent scientifically appropriate, Merck seeks to publish the results of studies it conducts. Some early phase or pilot studies are intended to be hypothesis-generating rather than hypothesis testing. In such cases, publication of results may not be appropriate since the trial may be underpowered and the analyses complicated by statistical issues of multiplicity.

Merck's policy on authorship is consistent with the requirements outlined in the ICH-Good Clinical Practice guidelines. In summary, authorship should reflect significant contribution to the design and conduct of the trial, performance or interpretation of the analysis, and/or writing of the manuscript. All named authors must be able to defend the trial results and conclusions. Merck funding of a trial will be acknowledged in publications.

III. **Subject Protection**

A. **IRB/ERC Review**

All clinical trials will be reviewed and approved by an independent IRB/ERC before being initiated at each site. Significant changes or revisions to the protocol will be approved by the IRB/ERC prior to implementation, except that changes required urgently to protect subject safety and well-being may be enacted in anticipation of IRB/ERC approval. For each site, the IRB/ERC and Merck's Consent Form Review department (U.S. studies) or Clinical Research Director (non-U.S. studies) will approve the subject informed consent form.

B. **Safety**

The guiding principle in decision-making in clinical trials is that subject welfare is of primary importance. Potential subjects will be informed of the risks and benefits of, as well as alternatives to, trial participation. At a minimum, trial designs will take into account the local standard of care. Subjects are never denied access to appropriate medical care based on participation in a Merck clinical trial.

All participation in Merck clinical trials is voluntary. Subjects are enrolled only after providing informed consent for participation. Subjects may withdraw from a Merck study at any time, without any influence on their access to, or receipt of, medical care that may otherwise be available to them.

C. Confidentiality

Merck is committed to safeguarding subject confidentiality, to the greatest extent possible. Unless required by law, only the investigator, sponsor (or representative) and/or regulatory authorities will have access to confidential medical records that might identify the research subject by name.

D. DNA Research

DNA sequence analyses, including use of archival specimens collected as part of a clinical trial, will only be performed with the specific informed consent of the subject. With IRB approval, an exception to this restriction on use of archival specimens may be possible (for instance, if specimens are de-identified and are not referable to a specific subject).

IV. Financial Considerations

A. Payments to Investigators

Clinical trials are time- and labor-intensive. It is Merck's policy to compensate investigators (or the sponsoring institution) in a fair manner for the work performed in support of Merck trials. Merck does not pay incentives to enroll subjects in its trials. However, when enrollment is particularly challenging, additional payments may be made to compensate for the time spent in extra recruiting efforts.

Merck does not pay for subject referrals. However, Merck may compensate referring physicians for time spent on chart review to identify potentially eligible subjects.

B. Clinical Research Funding

Informed consent forms will disclose that the trial is sponsored by Merck, and that the investigator or sponsoring institution is being paid or provided a grant for performing the trial. However, the local IRB/ERC may wish to alter the wording of the disclosure statement to be consistent with financial practices at that institution. As noted above, publications resulting from Merck studies will indicate Merck as a source of funding.

C. Funding for Travel and Other Requests

Funding of travel by investigators and support staff (eg, to scientific meetings, investigator meetings, etc) will be consistent with local guidelines and practices including, in the U.S., those established by the American Medical Association (AMA).

V. Investigator Commitment

Investigators will be expected to review Merck's Code of Conduct as an attachment to the trial protocol, and in signing the protocol, agree to support these ethical and scientific standards.

* In this document, "Merck" refers to Merck Sharp & Dohme Corp., which is a subsidiary of Merck & Co., Inc. Merck is known as MSD outside of the United States and Canada. As warranted by context, Merck also includes affiliates and subsidiaries of Merck & Co., Inc."

Appendix 2 DNA Sampling and Pharmacogenetic Analysis Procedures

1. **Definitions**
   a. Pharmacogenomics: The investigation of variations of DNA and RNA characteristics as related to drug response.
   b. Pharmacogenetics: A subset of pharmacogenomics, pharmacogenetics is the influence of variations in DNA sequence on drug response.
   c. Genomic Biomarkers: A measurable DNA and/or RNA characteristic that is an indicator of normal biologic processes, pathogenic processes, and/or response to therapeutic or other interventions.
   d. DNA: Deoxyribonucleic acid.
   e. RNA: Ribonucleic acid.
2. **Summary of Procedures for Pharmacogenetics**
   a. Subjects for Enrollment: All subjects enrolled in the current clinical trials will be considered for enrollment.
   b. Consent

      Informed consent for biosamples (ie, DNA, RNA, protein, etc) will be obtained during screening for protocol enrollment from all subjects or legal guardians, at an outpatient visit, or during an inpatient stay by the investigator or his or her designate.

      Subjects are not required to participate in the pharmacogenetic sub-study in order to participate in the main trial.
3. **Scope of Pharmacogenetic Study**

   The DNA sample collected in the current trial will be used to study various genetic causes for how subjects may respond to a drug. The DNA sample will be stored to provide a resource for future studies conducted by Merck focused on the study of genes responsible for how a drug enters and is removed by the body, how a drug works, other pathways a drug may interact with, or other aspects of disease. All samples will be used by Merck or designees and research will be monitored and reviewed by a committee of our scientists and clinicians.
4. **Techniques to Collect Samples**

   Blood samples will generally be obtained for all trial participants. Blood samples for both DNA and RNA isolation will usually be obtained at a time when the subject is having blood drawn for other trial purposes.
5. **Confidential Subject Information for Pharmacogenetic Analysis**

   Samples will be collected and sent to the laboratory designated for the trial where they will be processed (ie, DNA or RNA extraction, etc) following the Merck approved policies and procedures for sample handling and preparation.

When samples are collected for a specific genotype or expression analysis, this analysis will be detailed in the main body of the clinical protocol (**Section 7.7.3.1**). These samples will be processed, analyzed, and the remainder of the sample will be destroyed. The results of these analyses will be reported along with the other trial results. A separate sample will be obtained from subjects in these protocols for storage in the biorepository for future analyses.

To maintain privacy of information collected from samples obtained for storage and future analysis, Merck has developed secure policies and procedures to maintain subject privacy. At the clinical site, a unique Code will be placed on the blood sample for transfer to the storage facility. The Code is a random number used only to identify the biosample of each subject. No other personal identifiers will appear on the sample tube. The first Code will be replaced with a Sample Code (eg, Genetic Sample Code for DNA sample, Serum Sample code for serum sample) at the Central Laboratory or at the Merck designated facility. This sample is now a single coded sample. The Sample Code is stored separately from all previous sample identifiers. A secure code, hereinafter referred to as a “first coding key”, will be utilized to match the Sample Code to the original blood code and subject number to allow clinical information collected during the course of the study to be associated with the biosample. This “first coding key” will be transferred by the central laboratory or Merck designated facility under secure procedures to the Merck group designated as the entrusted keyholder to maintain confidentiality of the biosamples. The Sample Code will be logged into the primary biorepository database, and in this database this identifier will not have identifying demographic data or identifying clinical information (ie, race, sex, age, diagnosis, lab values) associated with it. The sample will be stored in a designated repository site with secure policies and procedures for sample storage and usage.

For DNA samples, a Storage Code will replace the Sample Code at the Merck designated facility. The DNA sample is now a double coded sample. This storage code will be stored separately from all previous sample identifiers. The second secure key referred to as a “second coding key” file will be transferred by the Merck designated facility under secure procedures to the Merck entrusted keyholder. Samples with the second code are sometimes referred to as de-identified samples. The use of the second code provides additional confidentiality and privacy protection for subjects over the use of a single code. Access to both coding keys is needed to link any data or samples back to a subject identifier.

The “keys” could be utilized to reconstruct the link between genetic information and identifiable clinical information, at the time of analysis. This linkage would not be possible for the investigator conducting the analysis, but may only be done by the Merck entrusted keyholder under strict security policies and procedures. The Merck entrusted keyholder will link the information, conduct the analysis, then issue an anonymized data summary on the initially single or double coded samples to the investigator conducting the genetic analysis. The only circumstance by which genetic information would be linked to clinical information would be those situations mandated by health authorities (eg, EMEA,

FDA), whereby this information would be directly transferred to the health authority. Once the link between subject's identifiers and the unique codes is deleted, it is no longer possible to trace the data and samples back to individual subjects through the coding keys. Anonymization is intended to prevent subject re-identification.

6. **Biorepository Sample Usage**

Samples obtained for the Merck biorepository will be used for analyses using good scientific practices. Exploratory analyses will not be conducted under highly validated conditions. The scope of research performed on these samples is limited to the investigation of the variability in inherited biomarkers that may correlate with a clinical phenotype in subjects.

Genetic analysis utilizing the DNA samples may be performed by the sponsor, or an additional third party (eg, a university investigator) designated by the sponsor. The investigator conducting the analysis will be provided with a double (single) coded sample. Reassociation of analysis results with corresponding clinical data will only be conducted by the Merck entrusted keyholder. Any contracted third party genetic analysis will conform to the specific genetic analysis outlined in the clinical protocol. DNA sample remaining with the third party vendor after genetic analysis will be returned to the sponsor or destroyed and documentation of destruction will be reported to Merck.

Consent form signed by the subject will be kept under secure storage for regulatory reasons. Information contained on the consent form alone cannot be traced to any samples, test results, or medical information once the specimens have been rendered de-identified. Laboratory personnel performing the genetic testing will not have access to the informed consent document, nor will they be able to identify subjects from the double (single) coded specimens. Specimens will be identified to the laboratory only by the Sample double (single) code. Subjects who decline to sign the informed consent document for the sub-study will not have the sample collected or stored, nor will they be discontinued from the main trial unless the pharmacogenetics sample is specifically required for trial enrollment.

A template of each site's informed consent will be stored in the Sponsor's clinical document repository. Each consent will be assessed for appropriate sample permissions. The tracking number on this document will be used to assign sample permissions for each sample in the entrusted keyholder's Sample Database.

7. **Withdrawal From the Biorepository and Pharmacogenetic Database**

   Subjects may withdraw their consent to store the blood sample or the DNA or RNA derived from it. Subjects can also request that their sample be destroyed at any time. If samples can be identified in any way (ie, are not anonymized samples), subjects may withdraw consent for banking samples at any time by contacting the investigator responsible for administering their initial informed consent. At that time, subject samples will be removed from the biorepository. Any DNA, RNA, or other biologic samples will be destroyed, destruction will be documented, and sample database information deleted. However, any analyses performed or data obtained from the samples prior to the subject withdrawing consent will not be deleted.

8. **Retention of Data and Biosamples**

   It is anticipated that data generated from processed samples collected during the course this trial will be retained for an indefinite period. DNA specimens will be maintained for potential analysis for 20 years from the acquisition. Samples will be destroyed according to Merck policies and procedures and this destruction will be documented in the repository database.

9. **Data Security**

   Pharmacogenetic and other research databases are accessible only to authorized sponsor and trial administrator research personnel and/or designated collaborators and are only stored and accessible as anonymized data. Database user authentication is highly secure, and is accomplished using network security policies and practices based in international standards (eg, ISO17799) to protect against unauthorized access. The Merck entrusted key holder maintains control over access to all sample data. These data are collected for pharmacogenetic research purposes only as specified in the clinical protocol and will not be used for any other purpose without explicit consent from the research subject.

10. **Reporting of Data to Subjects**

    There is no definitive requirement in either authoritative ethical guidelines or in relevant laws/regulations globally that research results have to be, in all circumstances, returned to trial participant. Some guidelines advocate a proactive return of data in certain instances.

    No information obtained from exploratory laboratory studies will be reported to the subject or family, and this information will not be entered into the clinical database maintained by Merck on subjects. Principle reasons not to inform or return results to the subject include: lack of relevance of data, limitations of predictive capability of research data, concerns of misinterpretation of data, absence of good clinical practices standards in exploratory research.

    If any exploratory results are definitively associated with clinical significance for subjects while the Merck clinical trial is still ongoing, investigators will be contacted with information as to how to offer genetic testing (paid for by Merck) to subjects enrolled and will be advised that genetic counseling should be made available for all who choose to participate.

If any exploratory results are definitively associated with clinical significance after completion of a clinical trial, Merck will publish the results without revealing specific subject information, inform all sites who participated in the Merck clinical trial, and post the anonymized results on our website or other accredited website(s) that allow for public access (eg, Disease-societies who have primary interest in the results) in order that physicians and subjects may pursue genetic testing if they wish to do so.

11. **Gender, Ethnicity, and Minorities**

Although many diagnoses differ in terms of frequency by ethnic population and gender, every effort will be made to recruit all subjects diagnosed and treated on Merck clinical trials for pharmacogenetic sampling. When studies with samples are conducted and subjects identified to serve as controls, every effort will be made to group samples from subjects and controls to represent the ethnic and gender population representative of the disease under current investigation.

12. **Risks Versus Benefits of Pharmacogenetic Testing**

For pharmacogenetic testing, risks to the subject have been minimized. Risks include those associated with venipuncture to obtain the whole blood sample. This sample will be obtained at the time of routine blood samples drawn for clinical reasons.

Data privacy concerns of the subject have been strictly protected against with Merck security, policies and procedures. Data privacy risks are largely limited to rare situations involving possible breach of confidentiality. In this highly unlikely situation there is risk that the information, like all medical information, may be misused.

It is necessary for subject-related data (ie, ethnicity, diagnosis, drug therapy and dosage, age, toxicities, etc) to be reassociated to double (single) coded samples at the time of data analysis. These subject data will be kept in a separate, secure Merck database, and all samples will be stripped of subject identifiers. No information concerning results obtained from genotyping or biomarker studies conducted with samples from the biorepository will be entered into clinical records, nor will it be released to outside persons or agencies, in any way that could be tied to an individual subject.

13. **Self-Reported Ethnicity**

Subjects who participate in pharmacogenetic study will be asked to provide self-reported ethnicity. Subjects who do not wish to provide this data may still participate in the pharmacogenetic study.

14. **Questions**

Any questions related to the genetic informed consent, genetic sampling, genetic sample handling, or genetic sample storage should be e-mailed directly to PPD [redacted]

Appendix 3 The National Institute of Neurological and Communicative Diseases and Stroke/Alzheimer’s Disease and Related Disorders Association (NINCDS-ADRDA) Criteria for Probable AD[18]

Confidential

I. The criteria for the clinical diagnosis of PROBABLE Alzheimer's disease include:

• dementia established by clinical examination and documented by the Mini-Mental Test; Blessed Dementia Scale, or some similar examination, and confirmed by neuropsychological tests;

• deficits in two or more areas of cognition;

• progressive worsening of memory and other cognitive functions;

• no disturbance of consciousness;

• onset between ages 40 and 90, most often after age 65; and

• absence of systemic disorders or other brain diseases that in and of themselves could account for the progressive deficits in memory and cognition.

II. The diagnosis of PROBABLE Alzheimer‘s disease is supported by:

• progressive deterioration of specific cognitive functions such as language (aphasia), motor skills (apraxia), and perceptions (agnosia);

• impaired activities of daily living and altered patterns of behavior;

• family history of similar disorders, particularly if confirmed neuropathologically; and

• laboratory results of:

  • normal lumbar puncture as evaluated by standard techniques,

  • normal pattern or non-specific changes in EEG, such as increased slow-wave activity, and

  • evidence of cerebral atrophy on CT with progression documented by serial observation.

III. Other clinical features consistent with the diagnosis of PROBABLE Alzheimer‘s disease, after exclusion of causes of dementia other than Alzheimer‘s disease, include:

- plateaus in the course of progression of the illness;

- associated symptoms of depression, insomnia, incontinence, delusions, illusions, hallucinations, catastrophic verbal, emotional, or physical outbursts, sexual disorders, and weight loss;

- other neurologic abnormalities in some patients, especially with more advanced disease and including motor signs such as increased muscle tone, myoclonus, or gait disorder;

- seizures in advanced disease; and

- CT normal for age.

IV. Features that make the diagnosis of PROBABLE Alzheimer‘s disease uncertain or unlikely include:

- sudden, apoplectic onset;

- focal neurologic findings such as hemiparesis, sensory loss, visual field deficits, and incoordination early in the course of the illness; and

- seizures or gait disturbances at the onset or very early in the course of the illness.

Appendix 4 Comparison of the Constrained Longitudinal Data Analysis Model and the Longitudinal ANCOVA Model and Guidance on Longitudinal Data Analysis: *Efficient Use of Baseline Information in Longitudinal Data Analyses*

Comparison of the Constrained Longitudinal Data Analysis Model and the Longitudinal ANCOVA Model

The Constrained Longitudinal Data Analysis (cLDA) model will be used as the primary analysis model in the BACE Phase 2/3 program. This one-page document highlights the relevant differences and similarities between the cLDA model and the traditional longitudinal ANCOVA model. These two models are fully defined and characterized in the attached Merck & Co., Inc. internal technical document "Guidance on Longitudinal Data Analysis: Efficient Use of Baseline Information in Longitudinal Data Analyses". This attached guidance document also provides a fuller explanation of the cLDA model, including additional advantages held by the cLDA model, not listed below, which are not applicable to the BACE program (but nevertheless, provide evidence that the cLDA model is preferred in general over the longitudinal ANCOVA model).

- Both models provide unbiased estimates for their model parameters, resulting in estimates of the treatment difference that are unbiased; this is true for the case of no missing data as well as when the data are missing-at-random (MAR).

- The estimated variance for the treatment difference is unbiased for the cLDA model. On average, the variance for the estimated treatment difference is also unbiased for the longitudinal ANCOVA model.

- The variance of the estimated treatment difference from the cLDA model will not be larger than the average variance from the longitudinal ANCOVA model. However, in most cases the variances from the two models are similar (as confirmed via simulation, see Appendix of the attached).

- The cLDA model provides unbiased standard errors for within-group treatment effects and provides coverage at the appropriate 100(1-α)% level, whereas the longitudinal ANCOVA does not.

- The cLDA model assumes that the underlying population means of all treatments are equal at baseline. It is noted that this assumption need not be verified as it is theoretically known to be true; the only way in which this assumption can be violated is if there is a problem with the blinded randomization to treatment group (a problem which is more severe than unequal baseline means).

Guidance on Longitudinal Data Analysis:

Efficient Use of Baseline Information in Longitudinal Data Analyses

1. Introduction

Measurements are often collected prior to treatment randomization in longitudinal clinical trials. This information can potentially be used for several purposes, including subject selection in studies targeting a study population with a certain disease condition, and as a starting point for measuring the treatment effect in a change from baseline analysis. Comparing treatments in terms of mean change from baseline is common in many longitudinal clinical trials. When there is only one post-randomization measure, treatment effects on mean change from baseline are often assessed using an analysis of covariance (ANCOVA) model with the baseline value as a covariate and either the post-baseline value or the calculated change from baseline value as the dependent variable; throughout this document, without loss of generality, we assume that the dependent variable is the change from baseline for ANCOVA-based analyses. Estimates and statistical tests from an ANCOVA model conditional on baseline values are unbiased under an assumption of normality [1]. When the baseline measurement is correlated with the post-baseline measurement, adjusting for baseline using ANCOVA has been shown to remove conditional bias in treatment group comparisons due to chance imbalances [2], and to improve efficiency over unadjusted comparisons [3].

With several post-randomization measurements (repeated measures over time), a longitudinal data analysis (LDA) model can be used, in which the change from baseline is calculated at each post-randomization time point and the baseline measurement is included as a covariate in the model. This model will hereafter be referred to as the Longitudinal ANCOVA model. Alternatively, a full likelihood approach can be used. First proposed by Liang and Zeger [4], in the full likelihood LDA model the baseline value is included as a dependent variable in the response vector, along with the post-baseline values. Of note, in the Longitudinal ANCOVA model, the baseline mean responses for each treatment group are implicitly assumed equal, which is reasonable due to randomization. A similar "constraint" can also be imposed in the full likelihood LDA model; the constrained full likelihood LDA model will hereafter be referred to interchangeably simply as the LDA model.

While both the Longitudinal ANCOVA and LDA models produce identical point estimates for the treatment effect in simple models that adjust for [categorical] time when no data are missing, estimates of standard errors for within-group mean changes from baseline from the LDA (but not the Longitudinal ANCOVA) model account for the variability of the baseline measurements. In addition, the LDA model does not exclude from the analysis post-randomization values for subjects with missing baseline values, leading to more efficient use of all the data.

A comprehensive review of late-stage clinical trials at Merck revealed inconsistencies and inefficiencies in methods being used for the analysis of longitudinal data among various therapeutic areas. This guidance document, produced by the Longitudinal Data Analysis Working Group of the Early/Late-stage Statistical Technical Issues Committee (ELSTIC), is intended to remove unnecessary disparities across BARDS sites for addressing analyses of mean change from baseline by recommending an efficient and statistically sound default strategy with supporting rationale.

The recommendation is to **use the full likelihood LDA model proposed by Liang and Zeger [4] as the default strategy for comparing treatments in terms of mean change from baseline in longitudinal clinical trials [5]. This LDA model will include baseline as one of the repeated measures with a constraint of equal mean across randomized groups at baseline, and use Kenward-Roger adjustment [6] with REML approach and an unstructured covariance [7].**

Table 1 provides a summary of the reasons for recommending the LDA over the Longitudinal ANCOVA model. When there are no missing data, the Longitudinal ANCOVA and the LDA models are generally comparable with respect to treatment group comparisons. However, in the presence of missing baseline data, the LDA model has superior power to detect treatment differences. Moreover, in both cases (missing or no missing data), confidence intervals for the least square means (LSMEANS) of the individual treatment groups are not covered at the appropriate 100(1-α)% level in the Longitudinal ANCOVA model, or in any model that uses baseline as a covariate. Note that this limitation also exists in the special case where there is only 1 post-baseline time point. As such, the recommendation is to use the LDA model in lieu of the standard ANCOVA model even when there is only one post-baseline time point. This recommendation also holds whether or not stratification factors are being adjusted for in the model.

Table 1
Advantages of the full likelihood LDA versus Longitudinal ANCOVA

Issue	Advantage of the LDA model
1) Missing baseline data.	The LDA model provides more efficient between-group comparisons because, unlike the Longitudinal ANCOVA model, it includes subjects who are missing a baseline measurement but have at least one post-baseline measurement.
2) Variability estimate for designing future trials.	The LDA model provides unbiased standard errors for within-group treatment effects, whereas the longitudinal ANCOVA model underestimates the variance.
3) Coverage of within-group confidence intervals for mean change from baseline.	The LDA model provides coverage at the appropriate 100(1-α)% level (under normality), whereas the Longitudinal ANCOVA model does not.
4) Subjects missing all post-baseline measurements.	The LDA model includes baseline measurements of such subjects in the analysis, whereas the Longitudinal ANCOVA model does not. If the probability of missing a post-baseline measurement depends on the magnitude of the baseline measurement (missing at random), then the LDA model will yield unbiased result but the Longitudinal ANCOVA model will not.
5) Implicit modeling assumptions regarding baseline means in models that adjust for stratification and stratification by time interaction.	The LDA model provides more flexibility and is less restrictive than the Longitudinal ANCOVA model. For example, a standard implementation of the latter implicitly (and often, erroneously) assumes that the baseline mean is the same for all strata. Moreover, the LDA model can more easily accommodate user-specified weights for the different strata for estimating and testing treatment effects.
6) Implicit modeling assumptions regarding correlation between baseline and post-baseline measurements in models that adjust for stratification and stratification by time interaction.	The Longitudinal ANCOVA model implicitly (and often, erroneously) assumes that the correlation between baseline and each post-baseline measurement is the same for all levels of the stratification factor. The LDA model does not make this restrictive assumption.

If the parameter of interest is the mean *percent change from baseline* rather than the mean change from baseline, then the following two options may be considered: (1) Use the LDA model in which the longitudinal response vector includes the baseline measurement and the calculated post-baseline percent change from baseline measurements; (2) Use the LDA model in which the longitudinal response vector includes the log-transformed baseline and post-baseline measurements, and then use the delta method, or any other appropriate method (eg, see [8]), to get point estimates and standard errors on the original measurement scale.

Of note, ignoring baseline values in the analysis is not recommended in general, unless baseline values are non-informative or confounded with covariates included in the analysis (eg, a categorical baseline severity variable).

A detailed description of the rationale for recommending use of the LDA model, along with suggestions on appropriate implementation and template language for the Data Analysis Section (DAS) of a clinical protocol, is provided in the sections that follow.

2. Longitudinal data analysis methods for comparing treatments in terms of mean change from baseline

Sections 2 through 5 consider models to compare treatments in terms of mean change from baseline while adjusting for time as a categorical factor in the model. Adjusting for additional variables (eg, stratification factors) is considered in Section 6.

2.1 Longitudinal ANCOVA model

Suppose responses are measured at baseline ($T = 0$) and at T post-baseline time points in a clinical trial. Let Y_{ijt} be the response for subject i, with treatment assignment j, at time t. The marginal mean of the change from baseline, conditional on baseline Y_{ij0}, at time t can be modeled as:

$$E(Y_{ijt}^* \mid Y_{ij0}) = \alpha_t Y_{ij0} + \beta_{jt} I(treatment = j) I(time = t), \quad t = 1, 2, \ldots, T,$$

where $Y_{ijt}^* = Y_{ijt} - Y_{ij0}$ is the change from baseline value at time t. The slope, α_t, can be different for each time t and β_{jt} is the effect for treatment j at time t after adjusting for the baseline effect. The standard analysis using this model assumes that post-baseline values are multivariate normally distributed. The model conditions on a subject's baseline value. As such, the baseline is treated as fixed (rather than as a random variable) in the analysis, and subjects with missing baseline values are excluded. This model corresponds to the commonly used ANCOVA model for a pre-post study design when $T = 1$.

With repeated measures (ie, $T > 1$), a covariance matrix can be specified in the mixed model to account for within subject correlation at times $t > 0$. A separate covariance matrix can be specified for each treatment group; however, because the baseline value is not part of the response vector, the correlation between baseline and each post-baseline measurement in the ANCOVA model is implicitly assumed to be the same for each treatment group. For convenience, we focus on the last time point ($t = T$) and assume the study has two arms, a test drug ($j = 1$) and a control ($j = 0$). The comparison of interest is the treatment effect on the change from baseline at the last time point:

$$\theta_T = \beta_{1T} - \beta_{0T} .$$

The change from baseline LSMEANS for test drug and control are estimated as:

$$\hat{\theta}_{1T} = \hat{\alpha}_T \tilde{Y}_{\bullet\bullet 0} + \hat{\beta}_{1T} \text{ and } \hat{\theta}_{0T} = \hat{\alpha}_T \tilde{Y}_{\bullet\bullet 0} + \hat{\beta}_{0T},$$

respectively, where $\tilde{Y}_{\bullet\bullet 0}$ is the overall mean at baseline of all subjects included in the analysis.

2.2 LDA model

Utilizing the same notation, the LDA model includes the baseline value as part of the response vector. The marginal mean responses can be modeled as:

$$E(Y_{ijt}) = \gamma_0 + \gamma_{jt} I(treatment = j) I(time = t \quad and \quad t > 0), \quad t = 0, 1, 2, \dots, T ,$$

where γ_0 is the mean response at $t = 0$, which is constrained to be the same for both treatment groups due to randomization, γ_{jt} is the effect for treatment j at time t. The LDA model assumes that baseline and post-baseline values are jointly multivariate normally distributed. A covariance matrix can be specified in the mixed model to account for within subject correlation at times $t \geq 0$ (including baseline). Unlike the ANCOVA model, when a separate covariance matrix is specified for each treatment group the correlation between baseline and each post-baseline measurement is not assumed to be the same for each treatment group.

The treatment effect on the change from baseline at the last time point is estimated from this model as:

$$\eta_T = \gamma_{1T} - \gamma_{0T} .$$

The change from baseline LSMEANS for test drug and control are estimated as:

$\hat{\gamma}_{1T}$ and $\hat{\gamma}_{0T}$, respectively.

3. Comparing the Longitudinal ANCOVA and LDA models

3.1 Treatment difference estimates

Under the Longitudinal ANCOVA model, the treatment difference is the conditional mean difference between treatment groups:

$$\theta_T = \beta_{1T} - \beta_{0T} = E(Y_{i1T}* - Y_{i0T}* \mid Y_0) = E(Y_{i1T} - Y_{i0T} \mid Y_0) .$$

Using conditional expectation, it can be seen that

$$\eta_T = \gamma_{1T} - \gamma_{0T} = E(Y_{i1T} - Y_{i0T}) = \theta_T .$$

Both models provide unbiased estimates for their parameters, resulting in estimates of the treatment difference that are unbiased; this is true for the case of no missing data, and when data are missing at random (MAR). Furthermore, baseline means are assumed equal for the test drug and control groups in both models. Therefore, the resulting treatment difference estimate for both models is the expectation of the response difference between groups under the constraint of common baseline mean.

The overall variance of the estimated treatment difference, $Var(\hat{\theta}_T)$, can be determined using the conditional variance formula:

$$Var(\hat{\theta}_T) = E(Var(\hat{\theta}_T \mid Y_0)) + Var(E(\hat{\theta}_T \mid Y_0))$$

where $Var(\hat{\theta}_T \mid Y_0)$ is the variance estimated from the Longitudinal ANCOVA model. The second term on the right hand side of the equation is zero since $E(\hat{\theta}_T \mid Y_0) = E(\hat{\beta}_{1T} \mid Y_0) - E(\hat{\beta}_{0T} \mid Y_0) = \beta_{1T} - \beta_{0T}$ is constant. Therefore,

$$Var(\hat{\theta}_T) = E(Var(\hat{\theta}_T \mid Y_0)) .$$

As such, on average, the variance for the estimated treatment difference is unbiased from the longitudinal ANCOVA model. This was confirmed empirically through simulations (see Appendix).

Under the LDA model, the estimated variance for the treatment difference estimate is unconditional. The variance estimate is based on REML and unbiased under the model assumption. It can be shown that

$$Var(\hat{\eta}_T) = Var(E_{Y_0}(\hat{\theta}_T)) \le E_{Y_0}(Var(\hat{\theta}_T \mid Y_0)) = Var(\hat{\theta}_T).$$

Therefore, the variance of the estimated treatment difference from the LDA model will not be larger than the average variance from the longitudinal ANCOVA. When there are no missing data, the point estimate of the treatment difference is identical. This implies that the LDA model will be at least powerful as the longitudinal ANCOVA for the treatment comparisons. In most of the cases, the variances from these two models are fairly similar though. This was also confirmed through simulations (see Appendix).

3.2 LSMEAN estimates

Under the Longitudinal ANCOVA model, the LSMEAN for individual treatment groups is defined as:

$$\hat{\theta}_{jT} = \hat{\alpha}_T Y_{\bullet\bullet 0} + \hat{\beta}_{jT}.$$

When there are no missing data, the baseline slope coefficient can be estimated as:

$$\hat{\alpha}_T = \frac{\sum_i \sum_j (y_{ijT} - y_{\bullet jT})(y_{ij0} - y_{\bullet j0})}{\sum_i \sum_j (y_{ij0} - y_{\bullet j0})^2} - 1$$

and

$$E(\hat{\alpha}_T) = \rho(\sigma_T / \sigma_0) - 1$$

where σ_T and σ_0 are the standard deviations of the responses at time T and baseline, respectively. In general, the parameter estimates for α_T, β_{1T} and β_{0T} are asymptotically unbiased under MAR. In fact, the change from baseline LSMEAN for each treatment group estimates the expected difference between response at time T and baseline for that group:

$$E(\hat{\theta}_{jT}) = E[E(\hat{\alpha}_T \widetilde{Y}_{\bullet\bullet 0} + \hat{\beta}_{jT} \mid Y_0)] = \alpha_T E[\widetilde{Y}_{\bullet\bullet 0}] + \beta_{jT} = E(Y_{ijT} - Y_{ij0}).$$

The last equality uses that the expected baseline means are the same across the subjects due to randomization.

However, the conditional variance estimate of $\hat{\theta}_{jT}$, $Var(\hat{\theta}_{jT} \mid Y_0)$, obtained from the Longitudinal ANCOVA model may incorrectly estimate the overall variance of $\hat{\theta}_{jT}$. Using the conditional variance formula, we have:

$$
\begin{aligned}
Var(\hat{\theta}_{jT}) &= E[Var(\hat{\theta}_{jT} \mid Y_0)] + Var[(\rho\sigma_T/\sigma_0 - 1)Y_{\bullet\bullet 0} + \beta_{jT}] \\
&= E[Var(\hat{\theta}_{jT} \mid Y_0)] + (\rho\sigma_T/\sigma_0 - 1)^2\sigma_0^2/N
\end{aligned}
$$

where N is the total sample size used in calculating the baseline mean. To illustrate the issue, assume equal allocation between treatment groups, no missing data and the same measurement variances over time (ie, $\sigma_T^2 = \sigma_0^2$). Under these assumptions, the residual variance for the Longitudinal ANCOVA model conditional on the baseline is $(\sigma_0^2(1-\rho^2))$, resulting in $E[Var(\hat{\theta}_{jT} \mid Y_0)] = c(\sigma_0^2(1-\rho^2)/N$, where c is a constant independent of σ_0^2 and N. This reduces $Var(\hat{\theta}_{jT})$ to:

$$
\begin{aligned}
Var(\hat{\theta}_{jT}) &\approx E[Var(\hat{\theta}_{jT} \mid Y_0)] + (\rho - 1)^2\sigma_0^2/N \\
&\approx E[Var(\hat{\theta}_{jT} \mid Y_0)][1 + (\rho - 1)^2/\{c(1-\rho^2)\}] \\
&\approx E[Var(\hat{\theta}_{jT} \mid Y_0)][1 + (1-\rho)/\{c(1+\rho)\}]
\end{aligned}
$$

Therefore, the estimated variance of the change from baseline LSMEAN obtained from the Longitudinal ANCOVA approach ($Var(\hat{\theta}_{jT} \mid Y_0)$) underestimates the overall variance of $\hat{\theta}_{jT}$. The relative amount of underestimation is proportional to $((1-\rho)/((1+\rho))$. It is more extreme when the correlation between baseline and the response at time T is small or negative, and less extreme when the correlation is close to 1. The relative underestimation does not decrease with increasing sample size, as confirmed through simulation (see Appendix).

Note that the above derivation holds asymptotically when there are missing data because the restricted maximum likelihood (REML) estimates are asymptotically unbiased for all parameters, as long as the data are missing at random. If there are subjects who have an observed baseline value but all post-baseline values are missing, and the missing data depends on the baseline value, then the MAR condition is no longer satisfied when these subjects are excluded from the analysis

model. As such, the Longitudinal ANCOVA model may produce biased LSMEAN estimates.

For the full likelihood LDA model, the parameter estimates are unconditional. All subjects with available data either at baseline or post-baseline are included in the analysis. Inference based on maximum likelihood is valid, as long as the data are missing at random. Therefore, there are no bias or variance underestimation issues for the LDA model.

3.3 Normality assumption

The Longitudinal ANCOVA model assumes that the post-baseline measurements are multivariate normally distributed, while the LDA model assumes that the baseline and post-baseline measurements are jointly multivariate normally distributed. The robustness of both models to deviations from normality was assessed via simulation (see Appendix). Both models were robust to mild departures from normality, but were generally inefficient under more severe departures from normality. As such, robust parametric or non-parametric alternatives to the REML-based analysis of the LDA model may be more appropriate if a considerable departure from normality is suspected; users should refer to the Guidance for Analysis of Continuous Non-normal Longitudinal Data for further details.

4. Longitudinal data analysis methods for an endpoint calculated from baseline in a non-linear form

When baseline is used to calculate a dependent variable of a non-linear form, the LDA model can still be used. For example, if the parameter of interest is the mean *percent change from baseline* rather than the mean change from baseline, then the following two options may be considered: (1) Use the LDA model in which the longitudinal response vector includes the baseline measurement and the calculated post-baseline percent change from baseline measurements; (2) Use the LDA model in which the longitudinal response vector includes the log-transformed baseline and post-baseline measurements, and then use the delta method, or any other appropriate method (eg, see [8]), to get point estimates and standard errors on the original measurement scale.

5. Issues with SAS MIXED procedure for LSMEAN estimates

When the SAS MIXED procedure is used to fit a longitudinal ANCOVA model with repeated measures and missing data, the default LSMEAN estimates may not be appropriate. For a given treatment group, the individual treatment group LSMEANS should be:

$$\hat{\theta}_{jT} = \hat{\alpha}_T \tilde{Y}_{\bullet\bullet 0} + \hat{\beta}_{jT}$$

where $Y_{\bullet\bullet 0}$ is the mean response at baseline of all subjects in the analysis population. The default in the SAS MIXED procedure is to calculate the baseline mean from the analysis dataset. When data are missing, some subjects have fewer observations in the analysis dataset. As a result, the default baseline mean is a weighted average of baseline values across subjects, where the weight is proportional to the number of observations in the analysis dataset for a given subject. Under MAR, the weighted baseline mean may be biased.

To fix this problem, the baseline mean should be calculated outside of the SAS MIXED procedure, using one baseline observation per subject. This calculated mean can be used in the LSMEAN statement with the AT =option or in the ESTIMATE statement.

When the model is more complicated (adjustment for other factors), the ESTIMATE statement provides more flexibility.

6. Stratification

Adjustments for stratification factors (such as study center, gender, etc.) do not pose any additional theoretical difficulties, although assumptions between models that appear similar can be different. For example, adding a stratification and stratification by time interaction to the Longitudinal ANCOVA model defined in Section 2.1 implicitly makes several assumptions, including that (1) the correlation between baseline and post-baseline measurements is the same for each level of the stratification variable; and (2) the baseline means are the same for each level of the stratification factor. The LSMEAN estimates are calculated in SAS using this common mean. In contrast, an LDA model with the additional stratification and stratification by time factors does not impose the same implicit assumptions. As a result, estimates from the two models will not be the same, even when there are no missing data. An LDA model can be constructed that imposes the same implicit assumptions as the Longitudinal ANCOVA model when adjusting for additional stratification factors, although in most situations the less restrictive assumptions of the LDA model may make more sense.

7. REML and Kenward-Roger adjustment

Restricted (or residual) maximum likelihood (REML) is the default estimation method for the covariance parameters in SAS PROC MIXED. It is a particular form of the maximum likelihood applied to linear functions of **Y**, say **KY**, for which **K** is specifically designed so that **KY** contains none of the fixed effects which are part of the model for **Y**. Let $\mathbf{Y} = \mathbf{X}\boldsymbol{\beta} + \boldsymbol{\varepsilon}$. Then **K** is any matrix with row rank n – rank(**X**) satisfying $\mathbf{KX} = \mathbf{0}$. As a result, variance components are estimated without being affected by the fixed effects, $\boldsymbol{\beta}$, and, in estimating variance components, degrees of freedom for the fixed effects are taken into account implicitly [9]. In contrast, the conventional maximum likelihood based on the full set of observations generally underestimates the variance components. Therefore, REML is the recommended estimation method for the covariance parameters.

By default, the variance for the fixed effects estimate, $\mathrm{var}(\hat{\beta}) = (X'V^{-1}X)^{-1}$, is estimated by plugging in the REML estimates of the covariance parameters, $\hat{\mathrm{var}}(\hat{\beta}) = (X'\hat{V}^{-1}X)^{-1}$. This underestimates the true variability of $\hat{\beta}$ as the uncertainty associated with the estimation of the covariance parameters is not taken into account. In addition, statistical inference for a linear combination of the fixed effects, H: $\mathbf{L}\boldsymbol{\beta} = \mathbf{0}$, is often based on the general t-statistic, $t = L\hat{\beta} / \sqrt{L(X'\hat{V}^{-1}X)^{-1}L'}$, which, in general, is only approximately t-distributed, and its degrees of freedom must be estimated. The default method for degrees of freedom in SAS PROC MIXED procedure is the "between-within" approach [??], which does not have good statistical properties when there are missing data. It is recommended to use DDFM=KR option in the MODEL statement of SAS PROC MIXED, which first adjusts the estimated variance for $\hat{\beta}$ and then computes Satterthwaite-type degrees of freedom [6]. It is shown that the adjusted variance estimate and corresponding degrees-of-freedom provide better statistical properties, especially for studies with small sample sizes.

8. Assessing the Recommendation Using Simulations

Results from an extensive simulation study (see Appendix) empirically confirm the more desirable properties of the LDA model presented in this guidance. The simulation study compared the LDA model to the Longitudinal ANCOVA model under a variety of scenarios when considering treatment and time factors in the model. As noted in Section 6, adjustments for additional stratification factors do not pose any additional theoretical difficulties, although consideration for the assumptions in Longitudinal ANCOVA model versus the LDA model may warrant some attention. When both models contain the same constraints for the stratification factor(s) and baseline means, the more desirable properties of the LDA model hold.

It is worth noting that the constraint (mean baseline responses are the same for both treatment groups) implicitly assumed in the Longitudinal ANCOVA model does not have to be assumed in the LDA model, but it makes sense in the context of

randomized clinical trials where proper randomization ensures that the assumption is true. When the assumption is true, the LDA model with the constraint will adjust for observed chance imbalances in baseline measurements between treatment groups and increase efficiency of treatment group comparisons [2, 3].

Results from a separate simulation study also empirically confirm the more desirable properties of the Kenward-Roger adjustment. The simulation study compared three methods for computing the denominator degrees of freedom for the tests of fixed effects: the between-within method, the Satterthwaite approximation, and the Kenward-Roger adjustment.

9. Implementing the Recommendation using SAS

The SAS code required to fit the LDA model for analyses in terms of change from baseline is provided in this section. For illustration, assume that there is 1 baseline and 3 post-baseline measurements for each subject, and a stratification variable that has 3 levels. The data take the form:

Subj	Strata	Trt	time	Y	cy	Bl
1	1	1	0	5.25664	0.00000	5.25664
1	1	1	1	4.65575	-0.60089	5.25664
1	1	1	2	2.58320	-2.67344	5.25664
1	1	1	3	1.16863	-4.08801	5.25664
2	2	1	0	4.27706	0.00000	4.27706
2	2	1	1	2.79553	-1.48153	4.27706
2	2	1	2	1.85245	-2.42461	4.27706
2	2	1	3	0.69176	-3.58530	4.27706
3	3	1	0	4.59872	0.00000	4.59872
3	3	1	1	3.69201	-0.90671	4.59872
3	3	1	2	2.71452	-1.88420	4.59872
3	3	1	3	1.52998	-3.06874	4.59872
⋮	⋮	⋮	⋮	⋮	⋮	⋮
31	1	2	0	5.69308	0.00000	5.69308
31	1	2	1	3.44989	-2.24319	5.69308
31	1	2	2	5.40212	-0.29096	5.69308
31	1	2	3	3.12272	-2.57036	5.69308
32	2	2	0	4.30366	0.00000	4.30366
32	2	2	1	3.12098	-1.18268	4.30366
32	2	2	2	3.16588	-1.13778	4.30366
32	2	2	3	2.79032	-1.51334	4.30366
33	3	2	0	4.48876	0.00000	4.48876
33	3	2	1	3.14964	-1.33912	4.48876
33	3	2	2	2.71894	-1.76982	4.48876
33	3	2	3	2.37974	-2.10902	4.48876
⋮	⋮	⋮	⋮	⋮	⋮	⋮

9.1 Fitting the LDA model in SAS without adjusting for stratification variables

The LDA model described in Section 2.2 of the guidance document can be fit within SAS using the following code:

```
******************************************************************
**;
** data step necessary prior to running SAS PROC MIXED;
******************************************************************
**;
DATA long; SET long;
    ARRAY T{4} t0-t3; * time indicator variables;
    ARRAY TT{4} tt0-tt3; * time by treatment 1 (vaccine) indicator variables;
    ** define week times treatment 1 indicator variables;
    DO i = 1 TO 4;
        t{i} = (time=(i-1));
        tt{i} = t{i}*(trt=1);
    END;
    DROP i;
RUN;

******************************************************************
**;
** LDA model fit in SAS PROC MIXED;
******************************************************************
**;
PROC MIXED DATA=long;
    CLASS subj time;
    MODEL y=time tt1 tt2 tt3/ddfm=KR;
    REPEATED time / SUBJECT=subj TYPE=UN;
    ESTIMATE 'T1 Diff (V-P)' tt1 1;
    ESTIMATE 'T2 Diff (V-P)' tt2 1;
    ESTIMATE 'T3 Diff (V-P)' tt3 1;
    ESTIMATE 'T1 Placebo LSM' time -1 1 0 0;
    ESTIMATE 'T2 Placebo LSM' time -1 0 1 0;
    ESTIMATE 'T3 Placebo LSM' time -1 0 0 1;
    ESTIMATE 'T1 Vaccine LSM' time -1 1 0 0 tt1 1;
    ESTIMATE 'T2 Vaccine LSM' time -1 0 1 0 tt2 1;
    ESTIMATE 'T3 Vaccine LSM' time -1 0 0 1 tt3 1;
    ODS OUTPUT Estimates=outm1;
RUN;
```

The corresponding Longitudinal ANCOVA model can be fit within SAS using the following code:

```
******************************************************************
**;
** Longitudinal ANCOVA model fit in SAS PROC MIXED;
******************************************************************
**;
PROC SQL NOPRINT;
    SELECT mean(y) INTO: bb
    FROM long WHERE time=0;
QUIT;
PROC MIXED DATA=long;
    WHERE time>0;
    CLASS subj time trt;
    MODEL cy = bl trt time trt*time bl*time/ddfm=KR;
    REPEATED time / SUBJECT=subj TYPE=UN;
    LSMEANS trt trt*time / pdiff at bl=&bb;
    ODS OUTPUT LSMeans=outm2a Diffs=outm2b;
RUN;
```

Of note, the baseline by time interaction term is included in the Longitudinal ANCOVA model for consistency in the modeling assumptions. If this interaction term is removed in the Longitudinal ANCOVA model, the correlation of the baseline measurement to each post-baseline measurement is assumed constant (even when specifying an unstructured correlation matrix). The LDA model does not make this assumption. If in reality there is no baseline by time interaction, enforcing the assumption of different correlations between the baseline measurement and each post-baseline measurement may result in a minor efficiency loss. Given the sample sizes of most clinical trials, there is no material effect on the efficiency of the analysis. However, if there really is such an interaction, leaving it out could potentially lead to more bias.

Also of note, a typical exploratory analysis in the analyses of longitudinal data is to refit the Longitudinal ANCOVA model defined in Section 2.1 with a baseline by treatment interaction, in order to assess whether the treatment effect is consistent across varying baseline values. A similar test cannot be performed using the LDA model since the baseline measurement is part of the response vector. As an exploratory analysis, the need for a formal test (and p-value) is open to discussion. If required, a baseline by treatment interaction can be explored using the LDA model after creating a categorical baseline variable and adjusting for it in the model. Alternatively, graphical displays can be used to assess the consistency of the treatment effect for varying levels of baseline.

Confidential

9.2 Adjusting for stratification variables when fitting the LDA model in SAS

An LDA model that adjusts for stratification variables can be fit within SAS using the following code (assuming equal weights for combining estimates across strata):

```
*****************************************************************
**;
** data step necessary prior to running SAS PROC MIXED;
*****************************************************************
**;
DATA long; SET long;
    ARRAY T{4} t0-t3; * time indicator variables;
    ARRAY TT{4} tt0-tt3; * time by treatment 1 (vaccine) indicator variables;
    ** define week times treatment 1 indicator variables;
    DO i = 1 TO 4;
        t{i} = (time=(i-1));
        tt{i} = t{i}*(trt=1);
    END;
    DROP i;
RUN;

*****************************************************************
**;
** LDA model fit in SAS PROC MIXED;
*****************************************************************
**;
PROC MIXED DATA=long;
    CLASS subj time strata;
    MODEL y=strata time strata*time tt1 tt2 tt3/ddfm=KR;
    REPEATED time / SUBJECT=subj TYPE=UN;
    ESTIMATE 'T1 Diff (V-P)' tt1 1;
    ESTIMATE 'T2 Diff (V-P)' tt2 1;
    ESTIMATE 'T3 Diff (V-P)' tt3 1;
    ESTIMATE 'T1 Placebo LSM' time -3 3 0 0
                    strata*time -1 -1 -1 1 1 1 0 0 0 0 0 0 / divisor = 3;
    ESTIMATE 'T2 Placebo LSM' time -3 0 3 0
                    strata*time -1 -1 -1 0 0 0 1 1 1 0 0 0 / divisor = 3;
    ESTIMATE 'T3 Placebo LSM' time -3 0 0 3
                    strata*time -1 -1 -1 0 0 0 0 0 0 1 1 1 / divisor = 3;
    ESTIMATE 'T1 Vaccine LSM' time -3 3 0 0 tt1 3
                    strata*time -1 -1 -1 1 1 1 0 0 0 0 0 0 / divisor = 3;
    ESTIMATE 'T2 Vaccine LSM' time -3 0 3 0 tt2 3
                    strata*time -1 -1 -1 0 0 0 1 1 1 0 0 0 / divisor = 3;
    ESTIMATE 'T3 Vaccine LSM' time -3 0 0 3 tt3 3
                    strata*time -1 -1 -1 0 0 0 0 0 0 1 1 1 / divisor = 3;
```

```
    ODS OUTPUT Estimates=outm1;
RUN;
```

Note that this model does not implicitly assume that the baseline means or the correlation between the baseline and post-baseline measurements are the same for each stratum. The typical Longitudinal ANCOVA model that does make this implicit assumption is fit within SAS using the following code:

```
*****************************************************************
**;
** Longitudinal ANCOVA model fit in SAS PROC MIXED;
*****************************************************************
**;
PROC SQL NOPRINT;
    SELECT mean(y) INTO: bb
    FROM long WHERE time=0;
QUIT;
PROC MIXED DATA=long;
    WHERE time>0;
    CLASS subj time trt strata;
    MODEL cy = bl trt time trt*time bl*time strata strata*time/ddfm=KR;
    REPEATED time / SUBJECT=subj TYPE=UN;
    LSMEANS trt trt*time / pdiff at bl=&bb;
    ODS OUTPUT LSMeans=outm2a Diffs=outm2b;
RUN;
```

Note that for a stratification factor included in the analysis model the SAS default LSMEANS will apply equal weight over the levels of the stratification factor. In order to use different weights, such as a weight proportional to number of subjects in each level of the stratification factor in the analysis dataset, the OM option may be used in the LSMEANS statement. If the ESTIMATE statement is used, a two-step approach may be considered to incorporate a different weighting scheme for the stratification factor [10].

10. Standard template language for DAS/SAP

Recommended template language on the use of the cLDA model for analyzing longitudinal clinical trials with adjustment for baseline is provided below. This template language is also applicable in the special case where T=1. For completeness, the template is also given for the regular LDA model without adjustment for baseline, which may be used for studies without baseline or when baseline is not informative for the analysis.

Confidential

For cLDA Model (with adjustment for baseline)

In the primary analysis, a constrained longitudinal data analysis (cLDA) method proposed by Liang and Zeger [4] will be used. This model assumes a common mean across treatment groups at baseline and a different mean for each treatment at each of the post-baseline time points. In this model, the response vector consists of baseline and the values observed at each post-baseline time point. Time is treated as a categorical variable so that no restriction is imposed on the trajectory of the means over time. The analysis model will also adjust for factor1, factor2, … (add other adjustment factors here, if applicable, and consider the time by factor interaction terms, as appropriate). The treatment difference in terms of mean change from baseline to a given time point will be estimated and tested from this model. An unstructured covariance matrix will be used to model the correlation among repeated measurements. The Kenward-Roger adjustment will be used with restricted (or residual) maximum likelihood (REML) to make proper statistical inference.

Although the baseline measurement is included in the response vector, it is independent of treatment, and hence, the baseline means are constrained to be the same for different treatment groups. Of note, in the event that there are no missing data, the estimated treatment difference from the above cLDA model will be identical to that from a traditional longitudinal ANCOVA model which uses the baseline value as a covariate. However, unlike longitudinal ANCOVA, the cLDA model accounts for variability in the baseline values, thus providing more accurate standard errors and confidence intervals for individual treatment effects. Moreover, this model allows the inclusion of patients who are missing either the baseline or post-baseline measurements, thereby increasing efficiency. Details of the model specification, assumptions, and SAS implementation codes are given in Appendix II.

The above REML-based analysis assumes that the vector of model-based residuals follows a multivariate normal distribution. Under severe departures from normality, the REML-based analysis can be inefficient or potentially misleading. Accordingly, the residuals from the REML-based analysis, scaled by the inverse Cholesky root of the marginal variance-covariance matrix, will be subjected to a test for normality. If normality is not rejected at the α=0.001 level, then the above REML-based analysis will serve as the primary analysis. However, if normality is rejected, then the primary analysis will be conducted using multiple imputation (MI) of missing values (if any) in conjunction with a robust regression (RREG) approach that uses M-estimation. Details of the normality test and the MI→RREG method, along with sample SAS code, are provided in Appendix xx. Of note, the 0.001 level for the normality test was chosen so that the default REML-based analysis is abandoned only under a clear departure from normality; moreover, this choice guarantees that there is no material inflation in the type I error rate for the treatment effect comparison due to a potential correlation between the test statistics for the treatment effect and the normality test.

For LDA Model (without adjustment for baseline)

In the primary analysis, a longitudinal data analysis (LDA) method will be used. This model assumes a different mean for each treatment at each of the repeated time points in the analysis. In this model, time is treated as a categorical variable so that no restriction is imposed on the trajectory of the means over time. The analysis model will also adjust for factor1, factor 2… (add other adjustment factors here, if applicable, and also consider the time by factor interaction terms, as appropriate). The treatment difference at a given time point will be estimated and tested from this model. An unstructured covariance matrix will be used to model the correlation among repeated measurements. The Kenward-Roger adjustment will be used with restricted (or residual) maximum likelihood (REML) to make proper statistical inference.

Of note, in the event that there are no missing data, the estimated treatment difference from the above LDA model will be identical to that from a corresponding traditional ANOVA model at a given time point. However, the LDA model allows the inclusion of patients who have missing data at certain time points, thereby increasing efficiency. Details of the model specification, assumptions, and SAS implementation code are given in Appendix 2.

The above REML-based analysis assumes that the vector of model-based residuals follows a multivariate normal distribution. Under severe departures from normality, the REML-based analysis can be inefficient or potentially misleading. Accordingly, the residuals from the REML-based analysis, scaled by the inverse Cholesky root of the marginal variance-covariance matrix, will be subjected to a test for normality. If normality is not rejected at the α=0.001 level, then the above REML-based analysis will serve as the primary analysis. However, if normality is rejected, then the primary analysis will be conducted using multiple imputation (MI) of missing values (if any) in conjunction with a robust regression (RREG) approach that uses M-estimation. Details of the normality test and the MI→RREG method, along with sample SAS code, are provided in Appendix xx. Of note, the 0.001 level for the normality test was chosen so that the default REML-based analysis is abandoned only under a clear departure from normality; moreover, this choice guarantees that there is no material inflation in the type I error rate for the treatment effect comparison due to a potential correlation between the test statistics for the treatment effect and the normality test.

<u>Note</u>: sample SAS codes for the normality test and the MI→RREG method are provided in Section 4 of the ELSTIC document: *Analysis of Continuous but Potentially Non-Normal Longitudinal Data*

Prepared by the ELSTIC Longitudinal Data Analysis Working Group:

Guanghan (Frank) Liu, Kaifeng Lu, Madhuja Mallick, Devan Mehrotra, Robin Mogg, Xiaojiang Zhan and Xin Zhao.

References

1. Crager RM (1987). Analysis of covariance in parallel-group clinical trials with pretreatment baselines. *Biometrics*, 43, 895-901.
2. Senn SJ (1989). Covariate imbalance and random allocation in clinical trials. *Statistics in Medicine*, 8, 467-475.
3. Frison L, Pocock SJ (1992). Repeated measures in clinical trials: analysis using mean summary statistics and its implications for design. *Statistics in Medicine*, 11, 1685-1704.
4. Liang K, Zeger, S (2000). Longitudinal data analysis of continuous and discrete responses for pre-post designs. *Sankhyā: The Indian Journal of Statistics,* 62 (Series B), 134-148.
5. Liu G, Lu K, Mogg R, Mallick M, Mehrotra DV (2009). Should baseline be a covariate or dependent variable in analyses of change from baseline in clinical trials? *Statistics in Medicine*, 28, 2509-1530.
6. Kenward MG, Roger JH (1997). Small sample inference for fixed effects from restricted maximum likelihood. *Biometrics*, 53, 983-997.
7. Lu K, Mehrotra DV (2009). Specification of covariance structure in longitudinal data analysis for randomized clinical trials. *Statistics in Medicine*, DOI: 10.1002/sim.3820
8. Wong P, Mukhopadhyay S, Quan H, Larson P (1997). Confidence intervals for between-treatment mean difference when analysis is on the log percent scale. *Proceedings of the Biopharmaceutical Section of American Statistical Association*, 111-116.
9. McCulloch CE, Searle SR (2001). *Generalized, Linear, and Mixed Models*. Wiley: New York.
10. Qi E, Liu G (2002). Two step approach to automatically generate ESTIMATE statements for pair-wise treatment comparisons. *SAS Conference Proceedings: PharmaSUG*, Salt Lake City, Utah.

Confidential

Appendix I: Simulation Study

An extensive simulation study was undertaken to assess the performance of the Longitudinal ANCOVA and the LDA models under a variety of scenarios. Treatment difference LSMEAN and individual treatment group LSMEAN estimates were compared with respect to bias, coverage, MSE, Type I error and Power.

We simulated data from two treatment groups (placebo and treatment) and four repeated measures per subject (including baseline). Four scenarios were considered: (a) 50 subjects per treatment group, $\sigma^2 = 1$ at each time point; (b) 50 subjects per treatment group, $\sigma^2 = 4$ at each time point; (c) 30 subjects per treatment group, $\sigma^2 = 4$ at each time point; and (d) 80 subjects per treatment group, $\sigma^2 = 4$ at each time point. Data were generated under (i) multivariate normal (Case 0); (ii) a truncated normal distribution at baseline and multivariate normal post-baseline (Case 1); and (iii) a truncated t-distribution with 3 degrees of freedom at baseline and multivariate t distribution with 3 degrees of freedom post-baseline (Case 2). The following correlation structure used was used when generating the data:

$$\begin{bmatrix} 1 & 0.7 & 0.4 & 0.2 \\ 0.7 & 1 & 0.7 & 0.4 \\ 0.4 & 0.7 & 1 & 0.7 \\ 0.2 & 0.4 & 0.7 & 1 \end{bmatrix}$$

For all cases, data were generated with a mean vector for the placebo group of $\mu = (3.0 \ \ 2.5 \ \ 2.3 \ \ 2.0)$. For Cases 1 and 2, the baseline distribution was left-truncated at 2. The post-baseline means under the alternative were chosen to target ~80% power under each scenario when no data were missing. In the analysis, an unstructured covariance matrix was assumed. Two missing data scenarios were considered: (i) no missing data; and (ii) 10% of baseline measurements missing completely at random and a monotone MAR missing data mechanism post-baseline. The probability a post-baseline measurement was missing depended on the measurement at the previous time point, with higher values resulting in a higher probability of drop-out. The percent of data missing by study week is shown in Table 2 for each scenario.

Table 2
Summary of Percent of Data Missing in Simulation Study

	Baseline	Time 1	Time 2	Time 3
**CASE 0**				
Placebo/Treatment (under null)	~10%	~13%	~22%	~30%
Placebo (under alt)	~10%	~13%	~22%	~30%
Treatment (under alt)	~10%	~19%	~31%	~40%
**CASE 1**				
Placebo/Treatment (under null)	~10%	~18%	~29%	~37%
Placebo (under alt)	~10%	~18%	~29%	~37%
Treatment (under alt)	~10%	~25%	~40%	~49%
**CASE 2**				
Placebo/Treatment (under null)	~10%	~22%	~34%	~42%
Placebo (under alt)	~10%	~22%	~34%	~42%
Treatment (under alt)	~10%	~29%	~44%	~53%

Results under the null hypothesis are provided in Tables 3 and 4 for the treatment difference LSMEAN estimate and individual LSMEAN estimates, respectively. Similar results are provided in Tables 5 and 6 under the alternative. The results indicate the following:

- When there are no missing data, the Longitudinal ANCOVA and the LDA models are generally comparable with respect to treatment group comparisons.
- In the presence of missing baseline data, the LDA model has superior power to detect treatment differences.
- In both cases (missing or no missing data), confidence intervals for the least square means (LSMEANS) of the individual treatment groups are not covered at the appropriate 100(1-α)% level in the Longitudinal ANCOVA model.
- Both models were robust to mild departures from normality and quite inefficient under more severe departures from normality. As such, other non-parametric methods may be considered if a considerable departure from normality is suspected.
- In the presence of missing data, the LDA model also resulted in treatment group estimates that did not always cover at the appropriate 100(1-α)% level when the data were not multivariate normal. However, this may be explained by the small effective sample sizes, as the appropriate coverage was attained in the LDA model when no data were missing.

An additional scenario was considered to assess the coverage of the confidence intervals for the LSMEANS of the individual treatment groups with "large" sample sizes in the optimal situation where the data are multivariate normal (Case 0) and no data are missing: (e) 250 subjects per treatment group, $\sigma^2 = 4$ at each time point. Results are provided in Table 7, and clearly demonstrate that the confidence intervals for the LSMEANS of the individual treatment groups are not covered at the appropriate 100(1-α)% level in the Longitudinal ANCOVA model regardless of sample size.

A separate set of simulation studies were conducted to compare the following three methods for computing the denominator degrees of freedom for the tests of fixed effects in the context of small samples: (1) the between-within method (SAS default), DDFM=BETWITHIN (or DDFM=BW); (2) a general Satterthwaite approximation, DDFM=SATTERTH (or DDFM=SAT); and (3) the Kenward-Roger adjustment, DDFM=KENWARDROGER (or DDFM=KR). We simulated data from two treatment groups (placebo and treatment) and three repeated measures per subject (including baseline). Under the null hypothesis H_0, the mean vector was (3.0, 2.5, 2.0) for both groups; under the alternative hypothesis H_1, the mean vector was (3.0, 2.5, 2.0) for the placebo group, and (3.0, 2.0, 1.0) for the treatment group. The variance-covariance matrix for the repeated measures was

$$4\begin{bmatrix} 1 & 0.8 & 0.6 \\ 0.8 & 1 & 0.8 \\ 0.6 & 0.8 & 1 \end{bmatrix}$$

The endpoint was change from baseline at the last time point (t=2). Each simulated data set consisted of 20 subjects with 12 on placebo and 8 on treatment (unbalanced allocation). Twenty thousand (20,000) simulated data sets were generated for each case. Three degrees of missing data were considered under both H_0 and H_1: (1) no missing data; (2) low amount of missing data (~8% at t=1, ~13% at t=2); and (3) moderate amount of missing data (~15% at t=1, ~23% at t=2). The three DDFM methods have no impact on the point estimate of fixed effects, hence the comparison was based on the variance estimates, type I error/power and coverage of 95% CIs. Results are provided in Table 8 for the between-group LSMEAN difference at time 2 and in Table 9 for the LSMEAN change from baseline at time 2 for the treatment group, respectively. The results indicate the following:

- The default variance estimate (as used by DDFM=BW and DDFM=SAT) underestimated the true variability of the fixed effects estimate.
- Although the variance estimates were identical for the DDFM=SAT and DDFM=BW options, the former produced smaller denominator degrees of freedom and resulted in better coverage and less inflated Type I error.
- The DDFM=KR option appropriately adjusted the estimated variance and the denominator degrees of freedom, and produced proper coverage and controlled the Type I error.

Confidential

Table 3
Simulation Results Comparing the LDA and Longitudinal ANCOVA Models under H_0
LSMEAN Treatment Difference in Change from Baseline at Time 3

Simulation Scenario	Case	LSMEAN OF TREATMENT DIFFERENCE IN CHANGE FROM BASELINE AT TIME 3							
		Bias		Coverage (%)		MSE		Type I Error (%)	
		LDA	Longitudinal ANCOVA	LDA	Longitudinal ANCOVA	LDA	Longitudinal ANCOVA	LDA	Longitudinal ANCOVA
**No Missing Data under H_0 (5000 simulations)**									
(a)	Case 0	0.000	0.000	95.2	95.4	0.038	0.038	2.3	2.2
n = 50/group,	Case 1	-0.000	-0.000	94.8	95.2	0.038	0.038	2.4	2.3
$\sigma^2 = 1$	Case 2	-0.001	-0.001	95.2	95.6	0.087	0.087	2.6	2.4
(b)	Case 0	-0.002	-0.002	94.9	95.3	0.156	0.156	2.6	2.5
n = 50/group,	Case 1	-0.000	-0.000	95.4	95.5	0.150	0.150	2.3	2.2
$\sigma^2 = 4$	Case 2	-0.001	-0.001	95.2	95.4	0.373	0.373	2.3	2.2
(c)	Case 0	0.002	0.002	94.6	95.0	0.262	0.262	3.0	2.8
n = 30/group,	Case 1	-0.000	-0.000	94.5	94.9	0.261	0.261	2.7	2.5
$\sigma^2 = 4$	Case 2	0.001	0.001	94.8	95.2	0.625	0.625	2.6	2.3
(d)	Case 0	-0.000	-0.000	94.9	95.1	0.095	0.095	2.6	2.5
n = 80/group,	Case 1	0.000	0.000	95.1	95.2	0.096	0.096	2.7	2.5
$\sigma^2 = 4$	Case 2	0.002	0.002	95.2	95.3	0.228	0.228	2.3	2.3
**Missing Data under H_0 (5000 simulations)**									
(a)	Case 0	0.002	0.002	95.0	95.4	0.048	0.048	2.4	2.3
n = 50/group,	Case 1	-0.001	-0.001	95.5	95.8	0.050	0.050	2.2	2.0
$\sigma^2 = 1$	Case 2	-0.003	-0.003	94.6	95.1	0.098	0.098	2.7	2.5
(b)	Case 0	-0.003	-0.003	94.8	95.1	0.213	0.213	2.6	2.6
n = 50/group,	Case 1	-0.003	-0.003	95.1	95.3	0.225	0.225	2.5	2.3
$\sigma^2 = 4$	Case 2	0.003	0.002	95.1	95.4	0.428	0.428	2.3	2.2
(c)	Case 0	-0.000	-0.000	94.6	95.1	0.358	0.358	2.9	2.7
n = 30/group,	Case 1	0.002	0.002	94.5	95.2	0.397	0.397	2.6	2.3
$\sigma^2 = 4$	Case 2	-0.013	-0.013	94.0	94.6	0.744	0.744	3.0	2.7
(d)	Case 0	0.002	0.002	94.9	95.1	0.130	0.130	2.9	2.8
n = 80/group,	Case 1	0.000	0.000	94.4	94.5	0.145	0.145	2.8	2.7
$\sigma^2 = 4$	Case 2	-0.002	-0.002	94.4	94.6	0.257	0.257	3.0	3.0

Table 4
Simulation Results Comparing the LDA and Longitudinal ANCOVA Models under H_0
LSMEAN of Change from Baseline at Time 3 for Placebo and Treatment Groups

Simulation Scenario	Case	LSMEAN of change from baseline at Time 3 for Placebo				LSMEAN of change from baseline at Time 3 for Treatment			
		% Bias		Coverage (%)		% Bias		Coverage	
		LDA	Longitudinal ANCOVA	LDA	Longitudinal ANCOVA	LDA	Longitudinal ANCOVA	LDA	Longitudinal ANCOVA
**No Missing Data under H_0 (5000 simulations)**									
(a)	Case 0	0.01%	0.01%	95.2	91.5	-0.01%	-0.01%	95.2	91.1
n = 50/group,	Case 1	-0.02%	-0.02%	94.6	92.2	0.02%	0.02%	95.3	93.1
$\sigma^2 = 1$	Case 2	-0.03%	-0.03%	95.4	92.8	0.03%	0.03%	94.4	91.1
(b)	Case 0	-0.10%	-0.10%	95.3	91.5	0.10%	0.10%	95.4	91.4
n = 50/group,	Case 1	0.00%	0.00%	95.5	93.7	0.00%	0.00%	95.5	94.1
$\sigma^2 = 4$	Case 2	-0.03%	-0.03%	94.5	91.2	0.03%	0.03%	95.1	92.1
(c)	Case 0	0.08%	0.08%	95.0	90.9	-0.08%	-0.08%	94.9	90.9
n = 30/group,	Case 1	-0.01%	-0.01%	94.7	92.9	0.01%	0.01%	94.5	93.1
$\sigma^2 = 4$	Case 2	0.03%	0.03%	94.8	91.8	-0.03%	-0.03%	94.8	91.9
(d)	Case 0	0.00%	0.00%	94.9	91.2	0.00%	0.00%	95.2	91.2
n = 80/group,	Case 1	0.01%	0.01%	95.1	93.3	-0.01%	-0.01%	94.6	92.8
$\sigma^2 = 4$	Case 2	0.05%	0.05%	95.4	92.4	-0.05%	-0.05%	94.9	92.3
**Missing Data under H_0 (5000 simulations)**									
(a)	Case 0	0.04%	0.04%	95.1	92.3	-0.11%	-0.11%	95.1	92.0
n = 50/group,	Case 1	0.09%	0.09%	94.9	92.8	0.18%	0.18%	95.2	93.7
$\sigma^2 = 1$	Case 2	-0.20%	-0.20%	94.4	91.6	0.00%	0.00%	93.6	90.7
(b)	Case 0	-0.54%	-0.54%	94.6	92.0	-0.29%	-0.29%	94.6	92.1
n = 50/group,	Case 1	-0.22%	-0.22%	94.4	93.7	-0.04%	-0.04%	94.7	94.2
$\sigma^2 = 4$	Case 2	0.14%	0.18%	91.9	91.1	0.02%	0.06%	91.5	91.0
(c)	Case 0	-0.05%	-0.05%	94.6	92.4	-0.04%	-0.04%	94.2	91.9
n = 30/group,	Case 1	0.03%	0.03%	93.5	93.2	-0.10%	-0.10%	94.5	94.0
$\sigma^2 = 4$	Case 2	-0.82%	-0.82%	91.3	91.2	-0.22%	-0.22%	92.0	91.6

Simulation Scenario	Case	LSMEAN of change from baseline at Time 3 for Placebo				LSMEAN of change from baseline at Time 3 for Treatment			
		% Bias		Coverage (%)		% Bias		Coverage	
		LDA	Longitudinal ANCOVA	LDA	Longitudinal ANCOVA	LDA	Longitudinal ANCOVA	LDA	Longitudinal ANCOVA
(d)	Case 0	0.10%	0.10%	94.7	91.9	-0.08%	-0.09%	94.9	92.5
n = 80/group,	Case 1	0.02%	0.02%	94.6	94.0	0.01%	0.01%	94.0	93.3
$\sigma^2 = 4$	Case 2	-0.18%	-0.17%	91.2	90.4	-0.07%	-0.06%	90.9	90.1

Table 5
Simulation Results Comparing the LDA and Longitudinal ANCOVA Models under H_1
LSMEAN Treatment Difference in Change from Baseline at Time 3

Simulation Scenario	Case	LSMEAN OF TREATMENT DIFFERENCE IN CHANGE FROM BASELINE AT TIME 3							
		% Bias		Coverage (%)		MSE		Power	
		LDA	Longitudinal ANCOVA	LDA	Longitudinal ANCOVA	LDA	Longitudinal ANCOVA	LDA	Longitudinal ANCOVA
**No Missing Data under H_1 (2000 simulations)**									
(a)	Case 0	-0.19%	-0.19%	94.3	94.5	0.040	0.040	85.8	85.4
n = 50/group,	Case 1	0.11%	0.11%	94.6	95.0	0.039	0.039	86.0	85.5
$\sigma^2 = 1$	Case 2	-0.18%	-0.18%	94.3	94.5	0.093	0.093	55.1	54.4
(b)	Case 0	0.13%	0.13%	94.7	94.9	0.156	0.156	83.9	83.2
n = 50/group,	Case 1	0.15%	0.15%	95.1	95.3	0.158	0.158	82.8	82.1
$\sigma^2 = 4$	Case 2	-0.17%	-0.17%	95.1	95.3	0.377	0.377	48.3	47.5
(c)	Case 0	0.19%	0.19%	94.7	95.3	0.265	0.265	82.2	81.3
n = 30/group,	Case 1	-0.25%	-0.25%	94.2	95.0	0.268	0.268	82.7	81.8
$\sigma^2 = 4$	Case 2	0.23%	0.23%	94.8	95.3	0.598	0.598	51.9	50.4
(d)	Case 0	-0.19%	-0.19%	94.8	94.9	0.098	0.098	82.2	81.9
n = 80/group,	Case 1	-0.13%	-0.13%	95.1	95.3	0.095	0.095	83.0	82.3
$\sigma^2 = 4$	Case 2	0.14%	0.14%	96.2	96.2	0.229	0.229	49.1	48.6
**Missing Data under H_1 (2000 simulations)**									
(a)	Case 0	-0.63%	-0.62%	94.5	94.8	0.054	0.054	75.1	73.9
n = 50/group,	Case 1	0.35%	0.40%	94.7	95.2	0.053	0.053	74.7	73.7
$\sigma^2 = 1$	Case 2	-0.72%	-0.71%	94.7	95.1	0.105	0.105	49.6	48.6
(b)	Case 0	-0.38%	-0.38%	94.0	94.3	0.237	0.237	69.3	67.8
n = 50/group,	Case 1	0.38%	0.38%	95.0	95.2	0.258	0.258	62.0	61.0
$\sigma^2 = 4$	Case 2	-0.47%	-0.47%	95.0	95.3	0.464	0.464	40.5	39.4
(c)	Case 0	1.11%	1.11%	94.3	94.8	0.382	0.382	68.9	67.3
n = 30/group,	Case 1	-0.98%	-0.98%	93.6	94.3	0.455	0.455	61.7	60.1
$\sigma^2 = 4$	Case 2	2.01%	2.01%	94.1	95.2	0.733	0.733	42.9	40.8

Simulation Scenario	Case	LSMEAN OF TREATMENT DIFFERENCE IN CHANGE FROM BASELINE AT TIME 3							
		% Bias		Coverage (%)		MSE		Power	
		LDA	Longitudinal ANCOVA	LDA	Longitudinal ANCOVA	LDA	Longitudinal ANCOVA	LDA	Longitudinal ANCOVA
(d)	Case 0	-0.87%	-0.87%	95.3	95.7	0.139	0.139	67.9	67.5
n = 80/group,	Case 1	-0.83%	-0.83%	94.2	94.4	0.165	0.165	62.2	61.0
$\sigma^2 = 4$	Case 2	1.01%	1.01%	95.0	95.5	0.277	0.277	42.6	41.4

Confidential

Table 6
Simulation Results Comparing the LDA and Longitudinal ANCOVA Models under H_1
LSMEAN of Change from Baseline at Time 3 for Placebo and Treatment Groups

Simulation Scenario	Case	LSMEAN of change from baseline at Time 3 for Placebo				LSMEAN of change from baseline at Time 3 for Treatment			
		% Bias		Coverage (%)		% Bias		Coverage	
		LDA	Longitudinal ANCOVA	LDA	Longitudinal ANCOVA	LDA	Longitudinal ANCOVA	LDA	Longitudinal ANCOVA
**No Missing Data under H_1 (2000 simulations)**									
(a)	Case 0	0.06%	0.06%	95.2	91.6	-0.04%	-0.04%	94.9	91.6
n = 50/group,	Case 1	-0.03%	-0.03%	94.9	92.1	0.02%	0.02%	95.2	92.6
$\sigma^2 = 1$	Case 2	0.04%	0.04%	93.9	90.4	-0.03%	-0.03%	94.5	91.9
(b)	Case 0	-0.08%	-0.08%	95.2	91.8	0.04%	0.04%	94.6	90.5
n = 50/group,	Case 1	-0.05%	-0.05%	94.7	92.9	0.03%	0.03%	95.6	93.4
$\sigma^2 = 4$	Case 2	0.04%	0.04%	94.6	91.3	-0.03%	-0.03%	94.6	91.3
(c)	Case 0	-0.14%	-0.14%	94.6	90.0	0.06%	0.06%	95.4	92.1
n = 30/group,	Case 1	0.10%	0.10%	95.4	93.6	-0.06%	-0.06%	94.2	92.4
$\sigma^2 = 4$	Case 2	-0.08%	-0.08%	95.3	92.2	0.05%	0.05%	95.1	92.3
(d)	Case 0	0.09%	0.09%	94.6	91.3	-0.05%	-0.05%	95.7	91.5
n = 80/group,	Case 1	0.03%	0.03%	95.5	93.9	-0.02%	-0.02%	94.6	92.8
$\sigma^2 = 4$	Case 2	-0.03%	-0.03%	94.8	92.3	0.02%	0.02%	95.3	91.9

Confidential

Simulation Scenario	Case	LSMEAN of change from baseline at Time 3 for Placebo				LSMEAN of change from baseline at Time 3 for Treatment			
		% Bias		Coverage (%)		% Bias		Coverage	
		LDA	Longitudinal ANCOVA	LDA	Longitudinal ANCOVA	LDA	Longitudinal ANCOVA	LDA	Longitudinal ANCOVA
**Missing Data under H_1 (2000 simulations)**									
(a)	Case 0	0.37%	0.37%	94.6	92.1	-0.01%	-0.01%	95.1	92.0
n = 50/group,	Case 1	-0.11%	-0.11%	94.8	93.0	0.04%	0.04%	94.7	93.4
$\sigma^2 = 1$	Case 2	0.15%	0.29%	93.2	90.3	-0.11%	-0.01%	94.6	91.9
(b)	Case 0	0.03%	0.02%	93.6	92.2	-0.19%	-0.19%	93.9	91.8
n = 50/group,	Case 1	-0.20%	-0.20%	93.7	93.4	0.03%	0.02%	94.7	93.9
$\sigma^2 = 4$	Case 2	0.69%	0.69%	91.2	90.9	0.30%	0.30%	90.4	89.4
(c)	Case 0	-1.82%	-1.82%	93.4	91.6	-0.08%	-0.08%	94.4	92.5
n = 30/group,	Case 1	0.65%	0.65%	94.2	94.4	-0.09%	-0.09%	93.6	93.2
$\sigma^2 = 4$	Case 2	-0.52%	-0.52%	90.3	90.4	0.48%	0.48%	91.4	90.8
(d)	Case 0	0.54%	0.54%	94.7	92.8	-0.13%	-0.13%	95.2	93.0
n = 80/group,	Case 1	0.08%	0.08%	94.4	94.1	-0.22%	-0.22%	94.0	93.2
$\sigma^2 = 4$	Case 2	-0.15%	-0.15%	91.0	91.3	0.19%	0.19%	91.2	89.9

Table 7
Simulation Results Comparing the LDA and Longitudinal ANCOVA Models under H_1
LSMEAN of Change from Baseline at Time 3 for Placebo and Treatment Groups

Simulation Scenario	Case	LSMEAN of change from baseline at Time 3 for Placebo				LSMEAN of change from baseline at Time 3 for Treatment			
		% Bias		Coverage (%)		% Bias		Coverage	
		LDA	Longitudinal ANCOVA	LDA	Longitudinal ANCOVA	LDA	Longitudinal ANCOVA	LDA	Longitudinal ANCOVA
**No Missing Data under H_1 (2000 simulations)**									
(c) n = 30/group, $\sigma^2 = 4$	Case 0	-0.14%	-0.14%	94.6	90.0	0.06%	0.06%	95.4	92.1
(b) n = 50/group, $\sigma^2 = 4$	Case 0	-0.08%	-0.08%	95.2	91.8	0.04%	0.04%	94.6	90.5
(d) n = 80/group, $\sigma^2 = 4$	Case 0	0.09%	0.09%	94.6	91.3	-0.05%	-0.05%	95.7	91.5
(d) n = 250/group, $\sigma^2 = 4$	Case 0	0.03%	0.03%	94.3	90.2	0.02%	0.02%	95.5	91.6

Table 8
Simulation Results Comparing Three DDFM Methods
LSMEAN Treatment Difference in Change from Baseline at Time 2

		Avg. of Estimated Variance			Type I Error/Power (%)			Empirical Coverage (%)		
**Simulation Scenario**	**Monte Carlo Variance**	**BW**	**SAT**	**KR**	**BW**	**SAT**	**KR**	**BW**	**SAT**	**KR**
**Under H_0 (20,000 simulations)**										
No missing	0.567	0.504	0.504	0.560	6.4	6.3	5.2	93.6	93.7	94.8
Low missing	0.660	0.568	0.568	0.656	7.1	6.7	5.1	92.9	93.3	94.9
Moderate missing	0.758	0.639	0.639	0.777	7.5	6.7	4.8	92.5	93.3	95.2
**Under H_1 (20,000 simulations)**										
No missing	0.567	0.504	0.504	0.560	27.9	27.7	24.6	93.6	93.7	94.8
Low missing	0.656	0.565	0.565	0.651	26.5	25.7	22.0	93.0	93.4	95.0
Moderate missing	0.749	0.632	0.632	0.765	25.2	23.8	19.3	92.6	93.3	95.3

Table 9
Simulation Results Comparing Three DDFM Methods
LSMEAN Change from Baseline at Time 2 for the Treatment Group

		Avg. of Estimated Variance			Empirical Coverage (%)		
**Simulation Scenario**	**Monte Carlo Variance**	**BW**	**SAT**	**KR**	**BW**	**SAT**	**KR**
**Under H_1 (20,000 simulations)**							
No missing	0.368	0.342	0.342	0.362	94.2	94.1	94.7
Low missing	0.422	0.380	0.380	0.414	93.7	93.8	94.8
Moderate missing	0.493	0.423	0.423	0.479	93.1	93.4	94.8

Appendix II: Sample Protocol Appendix for Model Details and SAS Implementation

Appendix for cLDA Model (with adjustment for baseline)

Let Y_{ijt} be the response for subject *i,* with treatment assignment *j*, at time *t*. The marginal mean responses of the cLDA model can be formulated as

$$E(Y_{ij0}) = \gamma_0, \quad t = 0,$$

and

$$E(Y_{ijt}) = \gamma_0 + \gamma_{jt}, \quad j = 0,1, \quad t = 1,2,..., T.$$

The mean response γ_0 at t =0 is constrained to be the same for both treatment groups due to randomization. The effect γ_{jt} denotes the change from baseline for treatment *j* at time *t*. The cLDA model assumes that baseline and post-baseline values have a joint multivariate normal distribution. An unstructured covariance matrix can be specified in the mixed model to account for within subject correlation at times $t \geq 0$ (including baseline).

The treatment difference for the mean change from baseline at time point *t*, $t = 1,2,..., T$ is defined as:

$$\eta_t = \gamma_{1t} - \gamma_{0t}.$$

At each time point *t*, $t = 1,2,..., T$, the mean change from baseline (LSMEANS) for test drug and control are γ_{1t} and γ_{0t}, respectively, as defined in the cLDA model above.

This longitudinal model provides valid statistical inference in the presence of possible missing data if the missing data mechanism is ignorable (or more specifically, missing at random [MAR] or missing completely at random [MCAR]). This missing data mechanism requires that the probability of a data point being missing does not depend on the missing data after adjusting for the observed data. *Some justification may be provided here, for example:*

In this study, we expect that MAR/MCAR mechanisms will underlie most of the missingness and the proportion of data missing not at random [MNAR], driven solely by unobserved values of the study endpoints, will be small. Specifically, discontinuation reasons may include lack of efficacy, clinical or laboratory AEs, relocation, withdrawal of consent, protocol violations, and/or data processing issues. Reasons such as relocation and data processing issues are likely to be MCAR. On the other hand, lack of efficacy may belong to MAR because the discontinuation may depend on the observed efficacy outcomes. The MAR or MNAR mechanisms might each underlie the other reasons to some extent. If treatment in large part determines the loss of data for these other reasons (such as clinical or laboratory

Confidential

AEs), the mechanism may be close to MAR because treatment assignment is an observed variable and included in the analysis model. Based on prior study results, missing data due to other reasons is relatively infrequent.

Sample SAS code is provided below to fit the full likelihood cLDA model. *(project teams are responsible to modify the following code according to their requirements)*

For example, assume that there is a baseline and three post-baseline measurements from a vaccine trial. The primary interest here is to compare the treatments (vaccine vs. placebo) in terms of mean change from baseline in longitudinal clinical trial. The full likelihood LDA method models the response as a function of treatment, time and the interaction of time by treatment.

```
*******************************************************************;
** data step necessary prior to running SAS PROC MIXED;
*******************************************************************;
DATA long; SET long;
ARRAY T{4} t0-t3; * time indicator variables;
ARRAY TT{4} tt0-tt3; * time by treatment 1 (vaccine) indicator variables;
** define week times treatment 1 indicator variables;
DO i = 1 TO 4;
t{i} = (time=(i-1));
tt{i} = t{i}*(trt=1);
END;
DROP i;
RUN;
*******************************************************************;
** LDA model fit in SAS PROC MIXED;
*******************************************************************;
PROC MIXED DATA=long;
CLASS subj time;  ** subj is the patient id number **;
MODEL y=time tt1 tt2 tt3/ddfm=KR;
REPEATED time / SUBJECT=subj TYPE=UN;
ESTIMATE 'T1 Diff (V-P)' tt1 1;
ESTIMATE 'T2 Diff (V-P)' tt2 1;
ESTIMATE 'T3 Diff (V-P)' tt3 1;
ESTIMATE 'T1 Placebo LSM' time -1 1 0 0;
ESTIMATE 'T2 Placebo LSM' time -1 0 1 0;
ESTIMATE 'T3 Placebo LSM' time -1 0 0 1;
ESTIMATE 'T1 Vaccine LSM' time -1 1 0 0 tt1 1;
ESTIMATE 'T2 Vaccine LSM' time -1 0 1 0 tt2 1;
ESTIMATE 'T3 Vaccine LSM' time -1 0 0 1 tt3 1;
ODS OUTPUT Estimates=outm1;
RUN;
```

Appendix for LDA Model (without adjustment for baseline)

Let Y_{ijt} be the response for subject *i,* with treatment assignment *j*, at time *t*. The marginal mean responses of the full likelihood LDA model is modeled as

$$E(Y_{ijt}) = \gamma_{jt}, \quad j = 0,1, \quad t = 1,2,......, T,$$

The LDA model assumes that repeated measurements follow a multivariate normal distribution. An unstructured covariance matrix can be specified in the mixed model to account for within subject correlation.

The treatment difference at time point *t*, $t = 1,2,..., T$ is defined as:

$$\eta_t = \gamma_{1t} - \gamma_{0t}.$$

For $t = 1,2,..., T$, the mean response (LSMEANS) for test drug and control are γ_{1t} and γ_{0t}, respectively, as defined in the LDA model above.

This longitudinal model provides valid statistical inference in the presence of possible missing data if the missing data mechanism is ignorable (or more specifically, missing at random [MAR] or missing completely at random [MCAR]). This missing data mechanism requires that the probability of a data point being missing does not depend the missing data after adjusting for the observed data. *Some justification may be provided here, for example:*

In this study, we expect that MAR/MCAR mechanisms will underlie most of the missingness and the proportion of data missing not at random [MNAR], driven solely by unobserved values of the study endpoints, will be small. Specifically, the discontinuation reasons may include lack of efficacy, clinical or laboratory AEs, relocation, withdrawal of consent, protocol violations, and/or data processing issues. Reasons such as relocation and data processing issues, are likely to be MCAR. On the other hand, lack of efficacy may belong to MAR because the discontinuation may depend on the observed efficacy outcomes. The MAR or MNAR mechanisms might each underlie the other reasons to some extent. If treatment in large part determines the loss of data for these other reasons (such as clinical or laboratory AEes), the mechanism may be close to MAR since treatment assignment is an observed variable and included in the analysis model. Based on the prior study results, missing data due to other reasons is relatively infrequent.

Sample SAS code is provided below to fit the full likelihood LDA model. *(project teams are responsible to modify the following code according to their requirements)*

For example, assume that there are three repeated measurements from a vaccine trial. The primary interest here is to compare the treatments (vaccine vs. placebo) at last time point in this longitudinal clinical trial. The full likelihood LDA method models the response as a function of treatment, time and the interaction of time by treatment.

```
*******************************************************************;
** LDA model fit in SAS PROC MIXED;
*******************************************************************;
PROC MIXED DATA=long;
CLASS subj trt time;  ** subj is the patient id number, trt is the treatment variable, time is
the time points for repeated measures **;
MODEL y=trt time trt*time / noint ddfm=KR;
REPEATED time / SUBJECT=subj TYPE=UN;
ESTIMATE 'T1 Diff (V-P)' trt -1 1 trt*time -1 0 0  1 0 0 ;
ESTIMATE 'T2 Diff (V-P)' trt -1 1 trt*time  0 -1 0  0 1 0 ;
ESTIMATE 'T3 Diff (V-P)' trt -1 1 trt*time  0 0 -1  0 0 1 ;
ESTIMATE 'T1 Placebo LSM' trt 1 0 time 1 0 0 trt*time 1 0 0  0 0 0;
ESTIMATE 'T2 Placebo LSM' trt 1 0 time 0 1 0 trt*time 0 1 0  0 0 0;
ESTIMATE 'T3 Placebo LSM' trt 1 0 time 0 0 1 trt*time 0 0 1  0 0 0;
ESTIMATE 'T1 Vaccine LSM' trt 0 1 time 1 0 0 trt*time 0 0 0  1 0 0;
ESTIMATE 'T2 Vaccine LSM' trt 0 1 time 0 1 0 trt*time 0 0 0  0 1 0;
ESTIMATE 'T3 Vaccine LSM' trt 0 1 time 0 0 1 trt*time 0 0 0  0 0 1;
ODS OUTPUT Estimates=outm1;
RUN;
```

Appendix 5 Predefined Limits of Change Criteria

Table 1
Predefined Limits of Change Criteria for Laboratory Data

Laboratory Test	Criteria† as a % of a Limit of Normal Range
**Hematology**	
Hematocrit (M)	≤94.9% LLN
Hematocrit (F)	≤94.1% LLN
Hemoglobin (M)	≤90.5% LLN
Hemoglobin (F)	≤81.9% LLN
WBC	≤64.2% LLN
	≥149.0% ULN
Neutrophils	≤37.0% LLN
Eosinophils	≥147.0% ULN
Platelets	≤57.7% LLN
	≥177.7%ULN
**Hepatic Function**	
Bilirubin	≥166.7% ULN
Alkaline Phosphatase	≥300% ULN
SGOT	≥300% ULN
SGPT	≥300% ULN
**Renal Function**	
Creatinine	≥142.9% ULN
**Clinical Chemistry**	
Sodium	≤94.7% LLN
	≥105.4% ULN
Potassium	≤88.2% LLN
	≥111.1% ULN

† A laboratory value must represent a worsening from baseline (ie, be more abnormal in the direction of interest) to meet the definition.
LLN=Lower limit of normal.
ULN=Upper limit of normal.

Table 2

Predefined Limits of Change Criteria for Vital Signs, Weight, and Temperature

Measurement	Criteria
Systolic blood pressure	≥180 mm Hg and ≥20 mm Hg increase from baseline
	≤90 mm Hg and ≥20 mm Hg decrease from baseline
Diastolic blood pressure	≥105 mm Hg and ≥15 mm Hg increase from baseline
	≤50 mm Hg and ≥15 mm Hg decrease from baseline
Pulse	≥120 bpm and ≥15 bpm increase from baseline
	≤50 bpm and ≥15 bpm decrease from baseline
Weight	≥7 % increase from baseline
	≥7 % decrease from baseline
Temperature	≥101°F and ≥2°F increase from baseline (≥38.3°C and ≥1°C increase from baseline)
Respiratory rate	> 25 or increase of ≥10 (per minute) from baseline
	< 5 or decrease of ≥10 (per minute) from baseline

Table 3

Predefined Limits of Change Criteria for ECGs

Measurement	Criteria
QTc Interval Fridericia	Prolongation compared to baseline ≥30 to ≤60 msec
	Prolongation compared to baseline >60 msec
	Value ≥500 msec

Appendix 6 The use of [18F]Flutemetamol, a Positron Emission Tomography (PET) tracer

This appendix provides an overview of the PET tracer [^{18}F]Flutemetamol characteristics, administration procedure to subject, radiation safety, PET imaging and procedure to optimize irradiation for protocol MK-8931-017. A detailed Investigator's Imaging Operations Manual (IIOM) for PET imaging of the brain has been developed by the Sponsor and its partners. The IIOM contains additional information and it is required for the Investigator site and PET imaging site personnel to be familiar with this document.

[^{18}F]Flutemetamol Injection

The drug product is [^{18}F]Flutemetamol Injection, and the drug substance (the active component of the drug product) is [^{18}F]Flutemetamol. [^{18}F]Flutemetamol Injection contains 0.9% sodium chloride, 7% ethanol (volume per unit volume), 0.5% Polysorbate 80 weight per unit volume in phosphate buffer (0.015 M). The radiochemical purity is greater than 90%. Further details concerning the physical and chemical characteristics of [^{18}F]Flutemetamol Injection are provided in the IB.

[^{18}F]Flutemetamol Injection is a fluorine-18 labeled PET tracer prepared as a ready-to-inject solution with a maximum of 20 µg total flutemetamol. Each patient dose should be received by the imaging center with a nominal activity of 185 MBq (5 mCi) +/-10% at the time of administration and not more than 6 µg/mL flutemetamol plus related substances with a maximum administered dose volume of 10 ml.

[^{18}F]Flutemetamol F18 Injection Administration

Prior to administration the [^{18}F]Flutemetamol Injection should be checked for evidence of discoloration or particulate matter and not used if either are seen.

Subjects will receive [^{18}F]Flutemetamol Injection by a qualified personnel as an intravenous (i.v.) injection completed within 40 seconds. After administration of [^{18}F]Flutemetamol Injection, the line must be flushed with 5 to 15 mL of sodium chloride i.v. infusion (0.9% w/v). The administration site should be evaluated just prior to, during and after injection for extravasation of [^{18}F]Flutemetamol Injection and/or for the presence of local irritation, and to assess for the presence of excess [^{18}F]Flutemetamol Injection remaining at the injection site. The [^{18}F]Flutemetamol Injection must be administered as received and may not be diluted with any substance, including normal saline, at the imaging site.

Any residual or unused material should be treated as radioactive waste with final disposal to be in accordance with local regulations.

Radiation profile of [^{18}F]-Flutemetamol Injection

The adult Effective Dose resulting from a [^{18}F]Flutemetamol Injection administration with an administered activity of 185 MBq (5 mCi) is 5.92 mSv. This Effective Dose of 5.92 mSv to be used in subjects over 50 years of age (55-85 years of age) is well within the Effective dose range 1-10 mSv of Category IIB as defined in EU Radiation protection 99 guidance (Directorate-General Environment, Nuclear Safety and Civil Protection, 1998). Equivalent doses for each target organ are provided in Table 1 below.

Table 1 Estimated Adult Radiation Absorbed Doses from Flutemetamol (^{18}F) Injection

Target Organ/Tissue	Absorbed Radiation Dose µGy/MBq
Adrenals	13
Brain	11
Breasts	5
Gallbladder wall	287
Heart wall	14
Kidneys	31
Liver	57
Lower large intestine wall	42
Lungs	16
Muscle	9
Osteogenic cells	11
Ovaries	25
Pancreas	15
Red marrow	13
Skin	5
Small intestine wall	102
Spleen	15
Stomach wall	12
Testes	8
Thymus	6
Thyroid	6
Upper large intestine wall	117
Urinary bladder wall	145
Uterus	25
Total body	12
**EFFECTIVE DOSE**	**32**

This detailed dosimetry information is taken from Section 7 “Summary Date and Guidance for the Investigator”, and specifically Section 7.2.3, “Radiation Dosimetry” of the Investigator’s Brochure of GE-067 Flutemetamol (18F) Injection edition #7, April 2013.

PET Imaging with [^{18}F]-Flutemetamol Injection

The description of the protocol and the consecutive actions involving the subject, nurses, and imaging personnel for [^{18}F]Flutemetamol PET imaging are summarized below.

- Have the subject use the restroom to empty their bladder.

- A qualified medical professional should prepare the line for intravenous (IV) injections.

- Allow the subject to lie comfortably in a bed or reclining chair. Supply the subject with blankets/pillows as needed to maximize the subject's comfort.

- Obtain a dose syringe with **5 mCi ± 10%** (5 mCi = 185 mBq) of [^{18}F]Flutemetamol available for injection. The dose is prepared as a ready-to-inject solution with a maximum administered dose volume of 10 ml. Assay the activity of the syringe with a dose calibrator. Record the assay time to the nearest minute. Do not add saline to the dose prior to administration. Adding saline could potentially lead to precipitation of dissolved solids.

	**Record the assayed dose of [^{18}F]Flutemetamol to the nearest 0.01 mCi and the assay time to the nearest minute.**

- Before administering the [^{18}F]Flutemetamol, make sure the dose is sufficient for imaging.

	**The dose of the [^{18}F]Flutemetamol should be 5 mCi ± 10%** (5 mCi = 185 mBq) **at the time of injection. The site is encouraged to contact Bioclinica for immediate direction if there is any uncertainty as to whether the dose is sufficient to administer to the subject.** In case the activity has fallen below 3 mCi, it is recommended to not dose the subject and expose the subject to unnecessary radiation as images of acceptable quality are unlikely to be obtained with such a dose of tracer.

- Inject the [^{18}F]Flutemetamol IV over a period of 40 or less seconds. Flush the line with 5-15 mL of normal saline. Re-assay the activity of the residual dose in the syringe after the injection.

	**Record the residual activity and time of assay.**<br><br>**Record the injected dose of the [^{18}F]Flutemetamol to the nearest 0.01 mCi and the injection time to the nearest minute.**

- Allow the subject to rest comfortably in the uptake room for about 75-80 min for incorporation of [^{18}F]Flutemetamol into the brain. At the end of this period, have the subject use the restroom to empty his/her bladder. Then usher the subject into the scanner room, position and secure him/her in the scanner according to procedures outlined in section 7.1.3 of IIOM for PET Imaging of the Brain.

- The rest period, restroom period, subject positioning in the scanner, the CT scan (for PET/CT Scanners), and accompanying activities of the technologist should be timed to start the [^{18}F]Flutemetamol emission acquisition **90 ± 5 min after ^{18}F-Flutemetamol injection**.

- For PET-CT scanners, acquire the CT scan (for attenuation correction) prior to the emission scan. Use CT acquisition parameters stored on your scanner for this specifically for this study, and described in the PET/CT specification sheet developed for your site by Bioclinica.

	**The [^{18}F] Flutemetamol emission acquisition should start 90 ± 5 min after [^{18}F] Flutemetamol injection.**

- Acquire a **dynamic**, 3D scan consisting of four (4) 300-sec frames.

- It is crucial that the subject's position is checked several times throughout the 20 minute emission scan. **THE MOST COMMON CAUSE OF FAILED STUDIES IS EXCESSIVE MOTION BETWEEN EMISSION AND TRANSMISSION (OR CT) SCANS.** Good practice is to check the patient's position at the end of each 5-minute scan frame. The subject's position should be returned as closely as possible to the original position at the beginning of the next scan frame.

- Remind the subject not to move before the scan. It is recommended that you not talk to the subject during the scan, but be sure to let them know that you will be continually monitoring them during the acquisition. There is no need to wake a subject that appears to be sleeping as this may cause unnecessary motion.

- For PET-only scanners, acquire the transmission scan (for attenuation correction) using rod sources for 5-6 minutes immediately after the emission scan.

- Upon completion of the [^{18}F]Flutemetamol exam, the scanning bed should be returned to the home position.

	**Before releasing the patient, check that the field of view adequately captures the full brain, including the cerebellum and pons, and that the images are free of motion artifacts. If necessary, rescan the patient.**

- Upon completion of the [^{18}F]Flutemetamol scan, allow the patient to relax while the scan is checked for patient motion and correct field of view. If the scan is judged to be unacceptable due to motion or other reasons, a rescan (consisting of both a CT scan and a second 20-minute PET scan) should be performed. Once it is verified the scan is acceptable (or after the completion of a repeat scan), the subject should be removed from the scanner.

Optimizing [^{18}F]Flutemetamol irradiation

[^{18}F]Flutemetamol Injection is a radioactive drug that emits positrons, which annihilate with electrons to produce gamma rays, and must be handled with safety measures to minimize radiation exposure to clinical personnel and patients.

To minimize radiation exposure, [^{18}F]Flutemetamol Injection should be used by or under the control of physicians who are qualified by specific training and experienced in the safe use and handling of radionuclides, and whose experience and training have been approved by the appropriate government agency authorized to license the use of radiopharmaceuticals.

[^{18}F]Flutemetamol Injection will be prepared and handled according to Good Manufacturing Practice (Europe)/ USB 823 (USA) at PET manufacturing sites within a reasonable traveling distance of the PET imaging sites. Before administration, the suitability of each preparation will be assessed by a number of quality control (QC) tests including radioactivity content, radiochemical, and chemical purity by high performance liquid chromatography and pH measurement, according to approved methods developed by GEHC. A record will be provided with each delivery of [^{18}F]Flutemetamol Injection, which will contain the batch number, radioactive concentration of injection (MBq/mL) at reference date and time, and shelf-life information. The [^{18}F]Flutemetamol Injection will be released from the manufacturing site for delivery to the local radiopharmacy only after all QA testing has been completed and the lot passed all required analyses for release.

The radiopharmacist or designee at the PET imaging site will receive the shipment and process the receipt per local regulations. It is the responsibility of the site radiopharmacist or designee to ensure that the correct activity is present in the injection syringe and that the product is used within the validity period as stated on the product vial or syringe and product sheet. The site radiopharmacist or designee will manage the safe transfer of the injection syringe to the person at the PET imaging site responsible for administering the injection to the subject, as applicable. Prior to administration, the [^{18}F]Flutemetamol Injection should be checked for evidence or discoloration or particulate matter and not used if either are seen. Furthermore, in case the activity has fallen below 111 MBq (3 mCi), it is recommended to not dose the subject and expose the subject to unnecessary radiation as images of acceptable quality are unlikely to be obtained with such a dose of tracer.

To minimize radiation dose to the bladder, hydration before and after [^{18}F]Flutemetamol Injection administration should be encouraged to permit frequent voiding. The patient should be encouraged to void before and after imaging with [^{18}F]Flutemetamol Injection and frequently thereafter for the next 24 hours.

As described in the radiation safety profile, the adult Effective Dose resulting from a [^{18}F]Flutemetamol Injection administration with an administered activity of 185 MBq (5 mCi) is 5.92 mSv. This Effective Dose of 5.92 mSv to be used in subjects over 50 years of age (55-85 years of age) is well within the Effective dose range 1-10 mSv of Category IIB as defined in EU Radiation protection 99 guidance (Directorate-General Environment, Nuclear Safety and Civil Protection, 1998).

INVESTIGATOR SIGNATURE PAGE

Abbreviated Title	An Efficacy and Safety Trial of MK-8931 in Mild to Moderate AD (EPOCH)
Title	A Randomized, Placebo Controlled, Parallel-Group, Double Blind Efficacy and Safety Trial of MK-8931 with a Long Term Double-Blind Extension in Subjects with Mild to Moderate Alzheimer's Disease. (Phase 2/3; Protocol No. MK-8931-017-13) (also known as SCH 900931, P07738)
Sponsor	Merck Sharp & Dohme Corp., a subsidiary of Merck & Co., Inc.<br>2000 Galloping Hill Road<br>Kenilworth, New Jersey 07033, U.S.A.
Trial Physician/Director	PPD [redacted]<br>[redacted] Merck Research Laboratories<br>PPD [redacted]
Date of Finalization of This Current Version of the Protocol	28-JUN-2016 – Amendment 017-17
Previous Versions of the Protocol	**11-SEP-2015**--Amendment 017-16 (Country Specific)<br>**26-JUN-2015**--Amendment 017-15 (Country Specific)<br>**24-APR-2015**--Amendment 017-14 (Country Specific)<br>**09-APR-2015** – Amendment 017-13<br>**05-MAR-2015** – Amendment 017-12 (Country Specific)<br>**26-FEB2015** – Amendment 017-11 (Country Specific)<br>**18 DEC 2014** – Amendment 017-10<br>**08 AUG 2014** - Amendment 017-09<br>**11 FEB 2014** – Amendment #5 (017-08)<br>**14 AUG 2013** – Amendment #4 (017-07)<br>**08 NOV 2012** – Amendment #3 Brazil Version 1 (017-06)<br>**26 SEP 2012** – Amendment #3 (017-05)<br>**23 JUL 2012** – Amendment #2 Brazil Version 2 (017-04)<br>**23 JUL 2012** – Amendment #2 Brazil Version 1 (017-03)<br>**20 JUN 2012** - Amendment #2 (017-02)<br>**31 MAY 2012** - Amendment #1 (017-01)<br>**19 JAN 2012** - Initial Protocol (017-00)

THIS CONFIDENTIAL INFORMATION ABOUT AN INVESTIGATIONAL DRUG OR PRODUCT IS PROVIDED FOR THE EXCLUSIVE USE OF INVESTIGATORS OF THIS DRUG OR PRODUCT AND IS SUBJECT TO RECALL AT ANY TIME. THE INFORMATION IN THIS DOCUMENT MAY NOT BE DISCLOSED UNLESS SUCH DISCLOSURE IS REQUIRED BY APPLICABLE LAWS OR REGULATIONS. SUBJECT TO THE FOREGOING, THIS INFORMATION MAY BE DISCLOSED ONLY TO THOSE PERSONS INVOLVED IN THE TRIAL WHO HAVE A NEED TO KNOW, WITH THE OBLIGATION NOT TO FURTHER DISSEMINATE THIS INFORMATION. THESE RESTRICTIONS ON DISCLOSURE WILL APPLY EQUALLY TO ALL FUTURE ORAL OR WRITTEN INFORMATION, SUPPLIED TO YOU BY THE SPONSOR OR ITS AFFILIATES OR REPRESENTATIVES THAT IS DESIGNATED AS "PRIVILEGED" OR "CONFIDENTIAL".

THIS PROTOCOL AMENDMENT AND ALL OF THE INFORMATION RELATING TO IT ARE CONFIDENTIAL AND PROPRIETARY PROPERTY OF MERCK SHARP & DOHME CORP., A SUBSIDIARY OF MERCK & CO., INC., WHITEHOUSE STATION, NJ, U.S.A.

.

Name and Degree of Sponsor Representative
Department of Sponsor Representative

dd MMM yyyy

I have read Protocol No. MK-8931-017-17 (also known as P07738) dated **28 June 2016**, including all appendices, and agree to conduct the trial in accordance with the protocol. The protocol and trial documents must also be approved by the IRBs/IECs and regulatory authorities as appropriate, before implementation at the site. I agree to implement the protocol and trial documents only after all necessary approvals have been obtained and the sponsor has confirmed that it is acceptable to do so.

Name, Degree, full mailing address of Investigator

Site Number

dd MMM yyyy
