# Supplementary material for: Further analyses of the safety of verubecestat in the phase 3 EPOCH trial of mild-to-moderate Alzheimer’s disease
Source: Alzheimers Res Ther. 2019 Aug 7;11:68. doi: 10.1186/s13195-019-0520-1 (PMC6685277; doi:10.1186/s13195-019-0520-1)
Supplement: Supplementary file 2 — Table S1. Number (%) of Participants Exceeding the Predefined Limits of Change in Vital Signs and ECG Measurements. (DOCX 21 kb) [file 13195_2019_520_MOESM2_ESM.docx]

**Table S1.** Number (%) of Participants Exceeding the Predefined Limits of Change in Vital Signs and ECG Measurements

|  |  | **MK-8931 12 mg** | **MK-8931 40 mg** | **Placebo** |
| --- | --- | --- | --- | --- |
|  | **Predefined Limit of Change** | **n/m (%)** | **n/m (%)** | **n/m (%)** |
| **Vital Sign Parameter** |  |  |  |  |
| Diastolic Blood Pressure (mmHg) | DBP ≤ 50 mm Hg and decrease ≥ 15 mm Hg | 14/650 (2.2) | 15/651 (2.3) | 16/651 (2.5) |
|  | DBP ≥ 105 mm Hg and increase ≥ 15 mm Hg | 3/650 (0.5) | 5/651 (0.8) | 6/651 (0.9) |
| Systolic Blood Pressure (mmHg) | SBP ≤ 90 mm Hg and decrease ≥ 20 mm Hg | 9/650 (1.4) | 17/651 (2.6) | 9/651 (1.4) |
|  | SBP ≥ 180 mm Hg and increase ≥ 20 mm Hg | 10/650 (1.5) | 11/651 (1.7) | 13/651 (2.0) |
| Pulse Rate (beats/min) | PULSE ≤ 50 bpm and decrease ≥ 15 bpm | 16/650 (2.5) | 14/651 (2.2) | 17/651 (2.6) |
|  | PULSE ≥ 120 bpm and increase ≥ 15 bpm | 1/650 (0.2) | 1/651 (0.2) | 1/651 (0.2) |
| Respiratory Rate (breaths/min) | RESP < 5 bpm or decrease ≥ 10 bpm | 9/650 (1.4) | 4/651 (0.6) | 7/651 (1.1) |
|  | RESP > 25 bpm or increase ≥ 10 bpm | 8/650 (1.2) | 17/651 (2.6) | 20/651 (3.1) |
| Temperature (C) | ≥ 38.3 C and increase ≥ 1 C | 2/650 (0.3) | 3/651 (0.5) | 2/650 (0.3) |
| Weight (kg) | ≥ 7% decrease from baseline | 148/624 (23.7) | 182/620 (29.4) | 84/639 (13.1) |
|  | ≥ 7% increase from baseline | 50/624 (8.0) | 44/620 (7.1) | 99/639 (15.5) |
| **ECG Parameter** |  |  |  |  |
| QTc Interval Fridericia (msec) | Prolongation compared to baseline ≥ 30 to ≤ 60 msec | 17/649 (2.6) | 23/646 (3.6) | 17/646 (2.6) |
|  | Prolongation compared to baseline >60 msec | 2/649 (0.3) | 2/646 (0.3) | 2/646 (0.3) |
|  | Post-baseline Value ≥ 500 msec | 1/649 (0.2) | 0/648 (0.0) | 0/646 (0.0) |
| Abbreviation: n/m: Number of participants in the population with valid predose and postdose values of the given parameter meeting the predefined limit criteria / Number of participants in the population with valid predose and postdose values of the given parameter. | | | | |
