# Supplementary material for: Further analyses of the safety of verubecestat in the phase 3 EPOCH trial of mild-to-moderate Alzheimer’s disease
Source: Alzheimers Res Ther. 2019 Aug 7;11:68. doi: 10.1186/s13195-019-0520-1 (PMC6685277; doi:10.1186/s13195-019-0520-1)
Supplement: Supplementary file 3 — Table S2. Number of Participants with Liver Function Laboratory Findings that Met Predetermined Criteria. (DOCX 21 kb) [file 13195_2019_520_MOESM3_ESM.docx]

**Table S2.** Number of Participants with Liver Function Laboratory Findings that Met Predetermined Criteria

|  | **12 mg** | | **40 mg** | | **Placebo** | |
| --- | --- | --- | --- | --- | --- | --- |
| **Criteria** | **n/m** | **(%)** | **n/m** | **(%)** | **n/m** | **(%)** |
| **Alanine Aminotransferase** | | | | | | |
| ≥3 x ULN | 7/650 | (1.1) | 5/650 | (0.8) | 7/651 | (1.1) |
| ≥5 x ULN | 5/650 | (0.8) | 2/650 | (0.3) | 2/651 | (0.3) |
| ≥10 x ULN | 1/650 | (0.2) | 1/650 | (0.2) | 1/651 | (0.2) |
| ≥20 x ULN | 0/650 | (0.0) | 1/650 | (0.2) | 1/651 | (0.2) |
| **Aspartate Aminotransferase** | | | | | | |
| ≥3 x ULN | 7/650 | (1.1) | 2/650 | (0.3) | 9/651 | (1.4) |
| ≥5 x ULN | 7/650 | (1.1) | 2/650 | (0.3) | 4/651 | (0.6) |
| ≥10 x ULN | 1/650 | (0.2) | 2/650 | (0.3) | 1/651 | (0.2) |
| ≥20 x ULN | 0/650 | (0.0) | 1/650 | (0.2) | 1/651 | (0.2) |
| **Aminotransferase (ALT or AST)** | | | | | | |
| ≥3 x ULN | 8/650 | (1.2) | 5/650 | (0.8) | 10/651 | (1.5) |
| ≥5 x ULN | 7/650 | (1.1) | 2/650 | (0.3) | 4/651 | (0.6) |
| ≥10 x ULN | 1/650 | (0.2) | 2/650 | (0.3) | 1/651 | (0.2) |
| ≥20 x ULN | 0/650 | (0.0) | 1/650 | (0.2) | 1/651 | (0.2) |
| **Bilirubin** | | | | | | |
| ≥2 x ULN | 3/650 | (0.5) | 2/650 | (0.3) | 1/651 | (0.2) |
| **Alkaline Phosphatase** | | | | | | |
| ≥1.5 x ULN | 6/650 | (0.9) | 5/650 | (0.8) | 7/650 | (1.1) |
| **Aminotransferase (ALT or AST) and Bilirubin** | | | | | | |
| AT ≥3 x ULN and BILI ≥1.5 x ULN | 3/650 | (0.5) | 2/650 | (0.3) | 1/651 | (0.2) |
| AT ≥3 x ULN and BILI ≥2 x ULN | 2/650 | (0.3) | 2/650 | (0.3) | 1/651 | (0.2) |
| **Aminotransferase (ALT or AST) and Bilirubin and Alkaline Phosphatase** | | | | | | |
| AT ≥3 x ULN and BILI ≥2 x ULN and ALP <2 x ULN | 0/650 | (0.0) | 1/650 | (0.2) | 0/651 | (0.0) |
| Abbreviations: n / m: Number of treated participants with valid postdose values of the laboratory parameters meeting the predefined limit criteria / Number of treated participants with valid postdose values of the laboratory parameters.  ALP: Alkaline phosphatase; ALT: Alanine aminotransferase; AST: Aspartate aminotransferase; AT: Aminotransferase (ALT or AST); BILI: Bilirubin; ULN: Upper limit of normal range. | | | | | | |
