# Supplementary material for: Further analyses of the safety of verubecestat in the phase 3 EPOCH trial of mild-to-moderate Alzheimer’s disease
Source: Alzheimers Res Ther. 2019 Aug 7;11:68. doi: 10.1186/s13195-019-0520-1 (PMC6685277; doi:10.1186/s13195-019-0520-1)
Supplement: Supplementary file 4 — Table S3. Number (%) of Participants Exceeding the Predefined Limits of Change in Laboratory Parameters. (DOCX 36 kb) [file 13195_2019_520_MOESM4_ESM.docx]

**Table S3.** Number (%) of Participants Exceeding the Predefined Limits of Change in Laboratory Parameters

|  |  | **12 mg** | **40 mg** | **Placebo** |
| --- | --- | --- | --- | --- |
| **Laboratory Parameter** | **Predefined Limit of Change** | **n/m (%)** | **n/m (%)** | **n/m (%)** |
| **Hematology** | | | | |
| Hematocrit (%)^‡^ | ≤94.9% LLN | 20/300 (6.7) | 15/272 (5.5) | 20/298 (6.7) |
| Hematocrit (%)^§^ | ≤94.1% LLN | 16/350 (4.6) | 9/378 (2.4) | 8/353 (2.3) |
| Hemoglobin (gm/dL)^‡^ | ≤90.5% LLN | 15/300 (5.0) | 12/272 (4.4) | 16/298 (5.4) |
| Hemoglobin (gm/dL)^§^ | ≤81.9% LLN | 5/350 (1.4) | 5/378 (1.3) | 5/353 (1.4) |
| Leukocytes (10[3]/microL) | ≤64.2% LLN | 0/650 (0.0) | 0/650 (0.0) | 2/651 (0.3) |
|  | ≥149% ULN | 7/650 (1.1) | 8/650 (1.2) | 5/651 (0.8) |
| Neutrophils (10[3]/microL) | ≤37.0% LLN | 3/650 (0.5) | 0/649 (0.0) | 2/650 (0.3) |
| Eosinophils (10[3]/microL) | ≥147.0% ULN | 5/650 (0.8) | 13/649 (2.0) | 8/651 (1.2) |
| Platelet (10[3]/microL) | ≤57.7% LLN | 1/650 (0.2) | 2/650 (0.3) | 3/651 (0.5) |
|  | ≥177.7% ULN | 1/650 (0.2) | 1/650 (0.2) | 2/651 (0.3) |
| **Hepatic Function** | | | | |
| Bilirubin (mg/dL) | ≥166.7% ULN | 6/650 (0.9) | 2/650 (0.3) | 2/651 (0.3) |
| Alkaline Phosphatase (IU/L) | ≥300% ULN | 0/650 (0.0) | 0/650 (0.0) | 1/650 (0.2) |
| AST (IU/L) | ≥300% ULN | 7/650 (1.1) | 2/650 (0.3) | 9/65 (1.4) |
| ALT (IU/L) | ≥300% ULN | 7/650 (1.1) | 5/650 (0.8) | 7/651 (1.1) |
| **Clinical Chemistry** | | | | |
| Sodium (mEq/L) | ≤94.7% LLN | 5/650 (0.8) | 2/650 (0.3) | 0/651 (0.0) |
|  | ≥105.4% ULN | 0/650 (0.0) | 0/650 (0.0) | 0/651 (0.0) |
| Potassium (mEq/L) | ≤88.2% LLN | 6/650 (0.9) | 3/650 (0.5) | 5/651 (0.8) |
|  | ≥111.1% ULN | 8/650 (1.2) | 6/650 (0.9) | 4/651 (0.6) |
| **Renal Function** | | | | |
| Creatinine (mg/dL) | ≥142.9% ULN | 6/650 (0.9) | 6/650 (0.9) | 7/651 (1.1) |
| ^‡^ For male. ^§^ For female.  Abbreviations: n / m: Number of participants in the population with valid postdose values of the laboratory parameter meeting the predefined limit criteria / Number of subjects in the population with valid postdose values of the given laboratory parameter.  LLN: Lower limit of normal range; ULN: Upper limit of normal range; ALT: Alanine aminotransferase; AST: Aspartate aminotransferase. | | | | |
