# Supplementary material for: Further analyses of the safety of verubecestat in the phase 3 EPOCH trial of mild-to-moderate Alzheimer’s disease
Source: Alzheimers Res Ther. 2019 Aug 7;11:68. doi: 10.1186/s13195-019-0520-1 (PMC6685277; doi:10.1186/s13195-019-0520-1)
Supplement: Supplementary file 5 — Table S4. Mixed-Model Analyses for Neuropsychiatric Inventory Total Score. (DOCX 19 kb) [file 13195_2019_520_MOESM5_ESM.docx]

**Table S4.** Mixed-Model Analyses for Neuropsychiatric Inventory Total Score

|  | **Change from Baseline** | | | **Change from Baseline Comparison vs. Placebo** | |
| --- | --- | --- | --- | --- | --- |
|  | **Least Squares Mean [n]** | | | **LSM Difference (95% CI) [*P*-value]** | |
|  | **12 mg** | **40 mg** | **Placebo** | **12 mg** | **4 0mg** |
| Timepoint | | | | | |
| Week 13 | 0.2 [628] | 0.5 [623] | 0.1 [632] | 0.2 (-0.8, 1.2) [0.7382] | 0.4 (-0.5, 1.4) [0.3995] |
| Week 26 | 1.6 [604] | 2.1 [590] | 1.1 [610] | 0.5 (-0.6, 1.6) [0.3883] | 1.0 (-0.2, 2.1) [0.0936] |
| Week 52 | 2.6 [559] | 2.5 [548] | 1.5 [570] | 1.1 (-0.1, 2.3) [0.0847] | 1.0 (-0.3, 2.2) [0.1267] |
| Week 78 | 3.4 [446] | 3.8 [432] | 2.7 [457] | 0.7 (-0.6, 2.1) [0.2949] | 1.1 (-0.4, 2.6) [0.1372] |
| Based on longitudinal Analysis of Covariance with categorical factors of geographic region, treatment, gender, APOE4 genotype, baseline use of Vitamin E, baseline AD medication, study cohort from the initial 78-week trial, and the interaction of time by treatment, with baseline NPI, the interaction of baseline NPI and time, the baseline value of MMSE and the baseline value of age included as continuous covariates.  Higher values indicate higher impairment,with a negative treatment difference (verubecestat-placebo) indicating favorable efficacy for verubecestat.  Abbreviations: n: Number of observations in the primary full-analysis-set population included in the analysis model at the stated timepoint. (Subjects missing any covariate are removed from the model).  SD: Standard Deviation; LS Mean=Least Squares Mean; SE: Standard Error of the LS Mean; CI: Confidence Interval; AD: Alzheimer’s disease; NPI: Neuropsychiatric Inventory; MMSE: Mini-Mental State Examination. | | | | | |
